# Comparative Genomic Analysis of Soybean Flowering Genes

**DOI:** 10.1371/journal.pone.0038250

**Published:** 2012-06-05

**Authors:** Chol-Hee Jung, Chui E. Wong, Mohan B. Singh, Prem L. Bhalla

**Affiliations:** Plant Molecular Biology and Biotechnology Laboratory, ARC Centre of Excellence for Integrative Legume Research, Melbourne School of Land and Environment, The University of Melbourne, Parkville, Victoria, Australia; Nanjing Forestry University, China

## Abstract

Flowering is an important agronomic trait that determines crop yield. Soybean is a major oilseed legume crop used for human and animal feed. Legumes have unique vegetative and floral complexities. Our understanding of the molecular basis of flower initiation and development in legumes is limited. Here, we address this by using a computational approach to examine flowering regulatory genes in the soybean genome in comparison to the most studied model plant, Arabidopsis. For this comparison, a genome-wide analysis of orthologue groups was performed, followed by an *in silico* gene expression analysis of the identified soybean flowering genes. Phylogenetic analyses of the gene families highlighted the evolutionary relationships among these candidates. Our study identified key flowering genes in soybean and indicates that the vernalisation and the ambient-temperature pathways seem to be the most variant in soybean. A comparison of the orthologue groups containing flowering genes indicated that, on average, each Arabidopsis flowering gene has 2-3 orthologous copies in soybean. Our analysis highlighted that the *CDF3*, *VRN1*, *SVP*, *AP3* and *PIF3* genes are paralogue-rich genes in soybean. Furthermore, the genome mapping of the soybean flowering genes showed that these genes are scattered randomly across the genome. A paralogue comparison indicated that the soybean genes comprising the largest orthologue group are clustered in a 1.4 Mb region on chromosome 16 of soybean. Furthermore, a comparison with the undomesticated soybean (*Glycine soja*) revealed that there are hundreds of SNPs that are associated with putative soybean flowering genes and that there are structural variants that may affect the genes of the light-signalling and ambient-temperature pathways in soybean. Our study provides a framework for the soybean flowering pathway and insights into the relationship and evolution of flowering genes between a short-day soybean and the long-day plant, Arabidopsis.

## Introduction

Plants switch to the reproductive phase of development when environmental and endogenous factors are the most favourable for reproductive success and seed production. This proper timing is the result of elaborate regulatory networks that coordinate the external stimuli with endogenous cues, inducing the expression of genes that initiate the floral transition at the shoot apical meristem (SAM).

Much of our current understanding of the floral initiation process is derived from studies using *Arabidopsis thaliana* as the model system. More than 180 Arabidopsis genes have been identified that play a role in regulating flowering time, and these genes have been organised into six major pathways (reviewed by Fornara *et al*. [1]). Although the photoperiod and vernalisation pathways monitor seasonal changes in day length or temperature and, hence, initiate flowering in response to exposure to long days or prolonged cold temperatures, the ambient temperature pathway coordinates the response to daily growth temperatures. The autonomous pathway together with those involving age or gibberellin constitutes the rest of the floral pathways, which function more independently of external stimuli. These pathways are integrated by downstream target genes including *LEAFY* (*LFY*), *FLOWERING LOCUS T* (*FT*) and *SUPPRESSOR OF CONSTANS1* (*SOC1*), with their resulting outcomes conveyed to floral meristem identity genes such as *APETALA1* (*AP1*) at the SAM that triggers the flowering process [2], [3].

Flowering is one of the most important agronomic traits influencing crop yield. There is thus a great necessity for research that examines the molecular control of this fundamental process in important crop species. This knowledge is critical for the breeding of climate change resilient crop varieties. Soybean, a major food crop, is also a member of the large and diverse legume family, which has the unique capability of forming nitrogen-fixing symbioses with soil microorganisms and has thus been used as part of sustainable agricultural practices for thousands of years. Soybean is distributed broadly across latitudes and is cultivated as different maturity groups, with each having a narrow range of latitudinal adaptation. Unlike Arabidopsis, soybean can undergo a reversion of flowering when plants are shifted from flowering inductive to non-inductive conditions [4]. In addition, soybean also follows a floral developmental plan that is distinct from that of Arabidopsis [5]. Therefore, an understanding of the molecular mechanisms underlying these soybean traits is of fundamental and practical interest.

Recent studies have begun to shed light on the molecular adaptation of different soybean cultivars to a wide range of photoperiodic conditions [6], [7]. These studies have highlighted similarities as well as differences in the roles of flowering time genes between soybean and Arabidopsis. The blue light receptor *CRYPTOCHROME2* (*CRY2*) regulates photoperiodic flowering in Arabidopsis; however, in soybean, *GmCRY1a* but not *GmCRY2a* is the major regulator of photoperiodic flowering. On the other hand, Kong *et al.* (2010) revealed that, although soybean contains several *FT* homologues, the dynamic expression of only two of them is responsible for the adaptation of soybean to diverse photoperiodic environments [6]. Nevertheless, it is still unclear if similar downstream target genes are activated in soybean as in Arabidopsis or how the floral pathway is modified to generate the outputs that reconcile the differences in floral development between the two species.

In view of the recent availability of the soybean genome sequence, we have undertaken a genome-wide analysis for the identification of all soybean orthologues for the corresponding Arabidopsis genes, particularly those involved in flowering. As a paleopolyploid, soybean contains duplicate copies of most genes, and these duplicates may have undergone sub- or neo-functionalisation. We identified 491 putative soybean flowering regulatory genes that are scattered randomly throughout the genome, and then we performed phylogenetic analyses of these gene families to acquire an understanding of the evolutionary relationships among these candidate genes. The identified putative soybean flowering genes were further subjected to an *in silico* gene expression analysis using two independent transcriptome datasets [8], [9]. Although the distributions of the soybean genes in the paralogue-rich groups are not correlated with the recently duplicated regions in the genome, soybean chromosome 16, especially in the ∼1.4 Mb region around 34–35 Mb, is highly enriched for genes within paralogue-rich groups. Our study provides an essential genomic resource for functional analyses of the soybean flowering pathway, facilitating future research and efforts into breeding robust high-yielding crop varieties.

## Results

### Identification of Soybean Homologues of Arabidopsis Genes

The most recent genome annotation of soybean lists 46,367 genes with high confidence from the current draft genome sequence of soybean [10]. The current soybean annotation (G.max 1.09) identifies the closest Arabidopsis homologue of nearly all of the predicted soybean genes. However, soybean genes are associated with only 55% of the total Arabidopsis genes in the TAIR9 annotation. Thus, we combined the information from the TAIR9 annotation together with the orthologue-based method, which clusters soybean genes and Arabidopsis genes independently into pre-defined orthologue groups (OGs) in the OrthoMCL database (release 5.0) [11]. Then, we matched soybean and Arabidopsis genes under the same OGs as putative orthologues (see Methods). This combined analysis for homologue identification connected 20,730 Arabidopsis genes in 11,344 OGs to 45,175 soybean genes (Dataset S1).

### Soybean Flowering-related Genes

**Figure 1:**
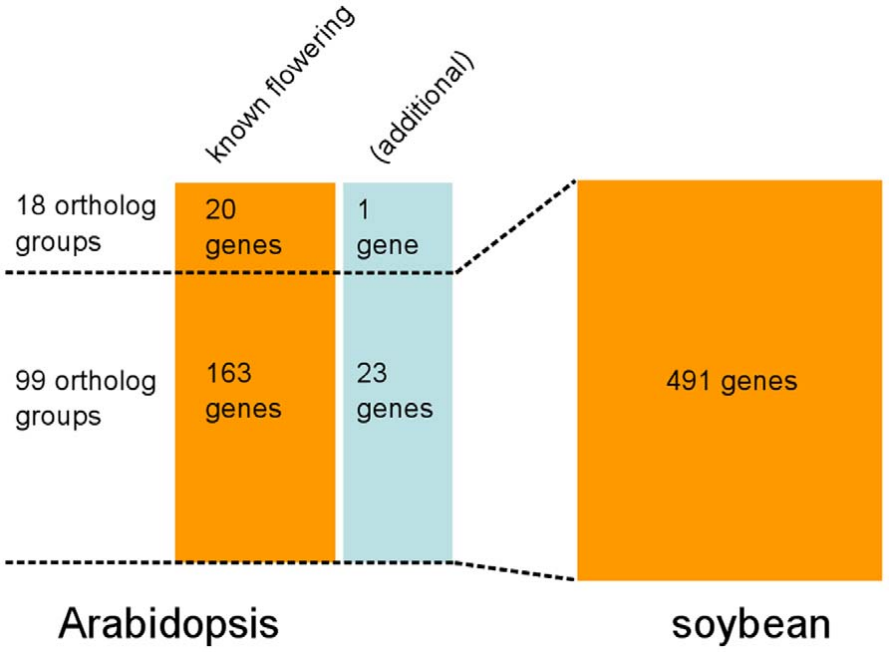
Number of orthologue groups and genes related to flowering pathways. 183 Arabidopsis flowering genes are classified into 117 orthologue groups along with 24 additional genes. Function of these additional genes has not been investigated so far. Out of the 117 OGs, 99 OGs contain 491 soybean genes that are putative soybean flowering genes. The numbers of genes are indicated within each box.

In this study, we focus on the 183 Arabidopsis genes that are known to take part in flowering regulatory pathways from previous studies [9], [12], [13], [14], [15], [16], [17], [18], [19], [20], [21], [22]. The orthologue identification analysis found 491 soybean genes that are putative flowering genes (Figure 1 and Table S1). The majority of the Arabidopsis flowering genes have putative soybean orthologues (163 out of 183). However, the soybean orthologues for 20 Arabidopsis flowering genes are not identified by the orthologue-based method used in this study. These Arabidopsis genes include *TARGET OF EARLY ACTIVATION TAGGED 2 (TOE2)*, *TOE3* and the vernalisation-insensitive genes *VERNALIZATION5/VIN3-LIKE 2* (*VEL2*), and *VEL3* (Table S1). Nevertheless, this lack of orthologue identification does not necessarily mean that these 20 Arabidopsis flowering genes are absent in soybean, as they still have similar soybean genes based on a direct BLAST analysis. In addition, the orthologue-based method identified 24 additional Arabidopsis genes that are grouped into the same OGs as known flowering genes (Table S1) but have not been investigated for their role in floral initiation.

**Figure 2:**
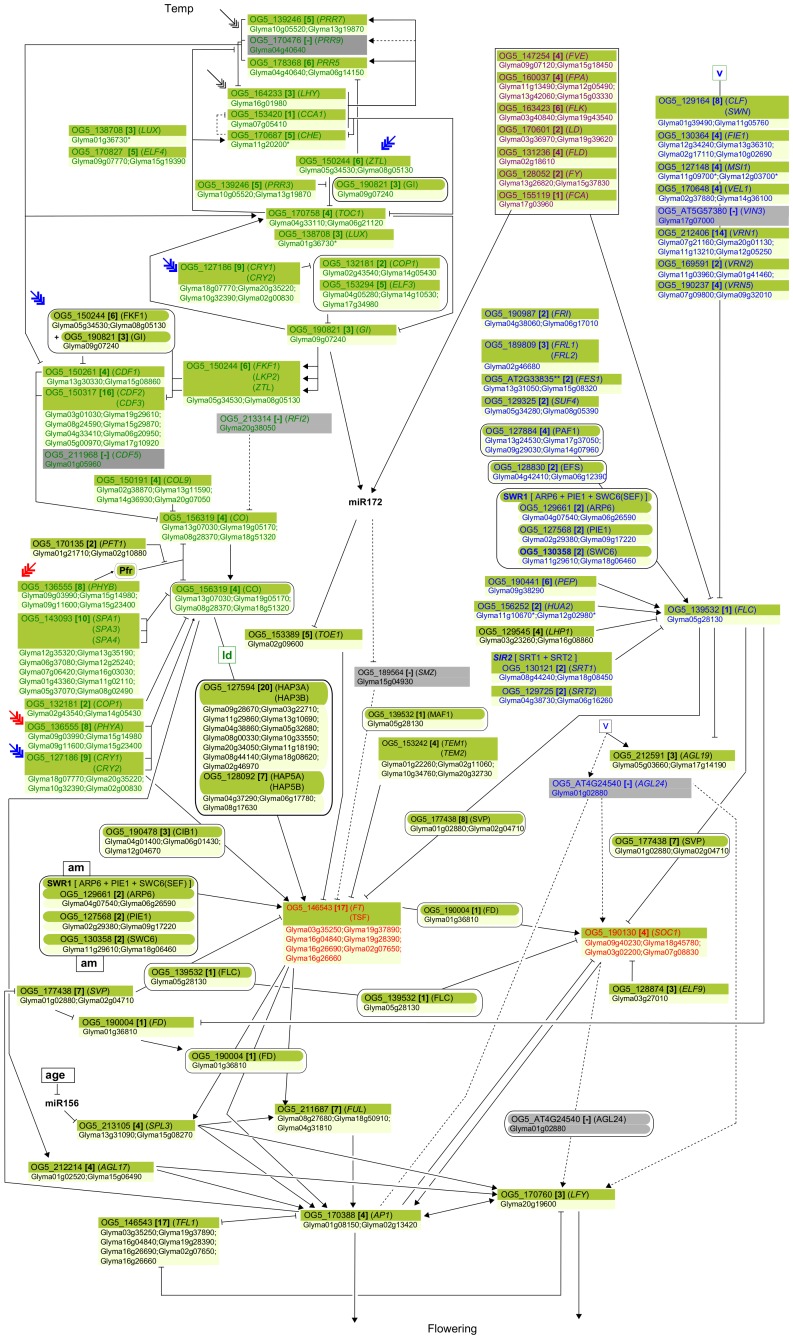
An outline of flowering pathway showing soybean orthologues for Arabidopsis flowering genes. Arabidopsis flowering pathway depicted by Higgins *et al*. (2010) [12] was adapted for this study. Arabidopsis gene symbols are shown in upper box of each node along with the corresponding OG ID and the total number of soybean genes in the same OG. Only the soybean genes that are closer to the Arabidopsis flowering genes in each OG are listed in each node. Arabidopsis genes in grey shades are those that are not assigned with putative soybean orthologues. However, based on BLAST analysis these genes still have homologous soybean genes, the best matching soybean gene in BLAST analysis is shown below these genes. Dashed-lines were used for the arrows or T-bars that involve grey shades. Soybean genes marked with * are those that share the same clade with Arabidopsis genes but have not been investigated so far as flowering genes. OG IDs starting with ‘OG5_AT’ are arbitrarily generated in this study and do not exist in OrthoMCL 5.0 database. Other conventions are same as those used in the Figure 1A by Higgins *et al*. (2010) [12]. Arrows show promoting effects, T-bars show repressing effects. Environmental cues are shown as lower case letters in square boxes; ‘v’ is extended cold (vernalization); ‘ld’ is long days; ‘sd’ is short days; ‘am’ is ambient (non-vernalizing) temperature. Genes are shown in italics and proteins in non-italics in ovals. ‘Pfr’ indicates P_fr_ phytochrome signaling. Arabidopsis genes assigned to specific pathways are color-coded (photoperiod pathway in green, vernalization in blue and autonomous pathway in purple). Flowering pathway integrators are shown in red. Triple headed arrows indicate activation by red or blue light.

Subsequent analyses of phylogenetic trees generated from the multiple sequence alignments of the soybean and Arabidopsis genes within each OG estimated that 322 genes are located in the same clades as the Arabidopsis genes known to be involved in flowering pathways (see Materials and Methods), indicating that they are likely the true orthologues of their corresponding Arabidopsis flowering genes. The simplified pathway diagram, which contains most of the Arabidopsis flowering-time genes and their putative soybean orthologues, is shown in Figure 2.

### Expression of Soybean Homologues of Arabidopsis Flowering Genes

The transcriptional activities of the putative soybean floral regulatory genes were examined to gather further evidence for their involvement in flowering. To this end, we utilised the soybean gene expression data from two recent transcriptome analyses [8], [9]. We found that the expression of most of the putative soybean floral genes (449 out of 491 genes; 91.4%) is supported by these two datasets (Figure S1 and Dataset S2). Furthermore, the vast majority of the expressed putative soybean flowering genes, 403 out of 449, exhibited transcriptional activities in flowers (Dataset S2), among which 19 genes are preferentially or specifically expressed in flowers [9]. These 19 genes are spread across 10 OGs, which contain the Arabidopsis MADS box genes, including *AP1*, *PISTILLATA* (*PI*) and *SEPALLATA1* (*SEP1*) (Table S2).

### Key Pathways and Gene Families for Flowering

**Figure 3:**
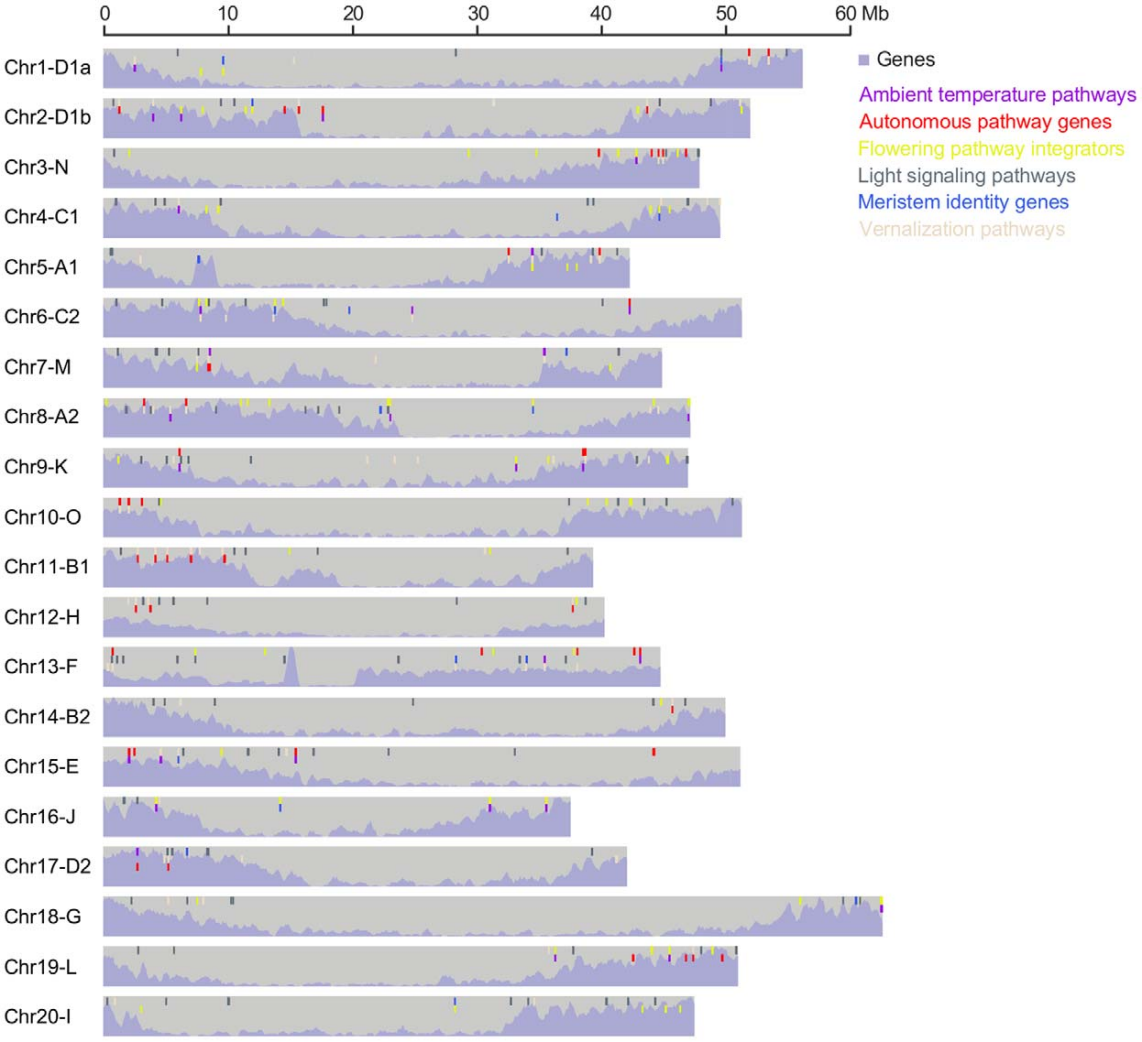
Genomic loci of soybean genes homologous to Arabidopsis floral regulatory genes. Soybean genes in Table 1 that are homologous to Arabidopsis floral regulatory genes are indicated with solid bars of different colours while the purple shade represents gene density. These soybean genes are randomly spread over the whole genome. Gene density depiction is adapted and modified from Figure 1 by Schmutz *et al*. (2010) [10].

Among the genes for flowering pathways, the key players are those that are involved in the light-signalling pathway, the vernalisation pathway, the autonomous pathway and the ambient temperature pathway, along with genes for meristem identity and flowering pathway integrators [12]. In Arabidopsis, 120 genes, which are grouped into 69 OGs, are known to be key players in flowering [12]. Among these 120 Arabidopsis genes, 112 of them are found in 62 OGs having 314 putative soybean orthologues, (Table 1). Table 1 shows the number of OGs associated with the key pathways or groups of genes for flowering. As one gene can take part in two or more different pathways, one OG can participate in multiple pathways or groups. The 237 soybean genes that are orthologous to key flowering genes of Arabidopsis are scattered throughout the genome rather than clustered within certain regions (Figure 3).

**Table 1:** Key flowering pathways and the numbers of associated genes in Arabidopsis, soybean, Medicago, *A. lyrata* and Brachypodium.

Pathway	OGs	Arabidopsis+	Soybean*	Medicago	*A lyrata*	Brachypodium
Light signaling	25	48/(53)	121/(115)	47	59	44
Vernalization	23	32/(36)	81/(71)	46	33	25
Autonomous	16	17/(23)	49/(46)	33	24	23
Ambient temperature	8	16/(19)	38/(30)	28	18	9
Meristem identity	5	7/(7)	19/(18)	6	7	3
Flowering pathway integrators	11	36/(39)	82/(69)	32	40	33

As one gene can take part in two or more different pathways, one OG can participate in multiple pathways or groups.

+ Numbers within parentheses indicate the total number of Arabidopsis genes belonging to the OGs associated with the given pathways.

* Numbers within parentheses indicate the number of soybean genes showing transcriptional activity detected by using either of the two recent transcriptome datasets [8], [9].

#### Light signalling pathways

Light is one of the main environmental regulators of flowering in plants. Plants sense the time of day and season of year by monitoring the light environment through light signalling pathways [23]. Soybean is a facultative short-day crop, but soybean cultivars also belong to different maturity groups depending upon their photoperiod sensitivity. This strong latitudinal cline is also observed in its undomesticated wild relative, *Glycine soja* (*G. soja*). In Arabidopsis, photoperiod pathway genes together with photoreceptor genes and circadian clock components take part in light signalling pathways. The number of known Arabidopsis flowering genes involved in these pathways is 48, which are clustered into 25 OGs. However, these OGs contain 53 Arabidopsis genes in total, suggesting that the additional 5 genes may also be involved in floral initiation (Table 1). In total, 121 soybean genes are identified as putative orthologues of 48 Arabidopsis flowering genes in 25 OGs (Table 1). The multiple sequence alignments followed by phylogenetic tree analyses for the Arabidopsis and soybean gene sequences in each of the 25 OGs revealed that 66 of the soybean genes are more closely located to their corresponding Arabidopsis genes than other soybean genes in the same OGs (Dataset S3). Furthermore, an *in silico* gene expression analysis of the identified soybean flowering genes determined that 115 of the 121 soybean orthologues are expressed, including 109 genes expressed in flowers [8], [9] (Figure S1 and Dataset S2).

**Figure 4:**
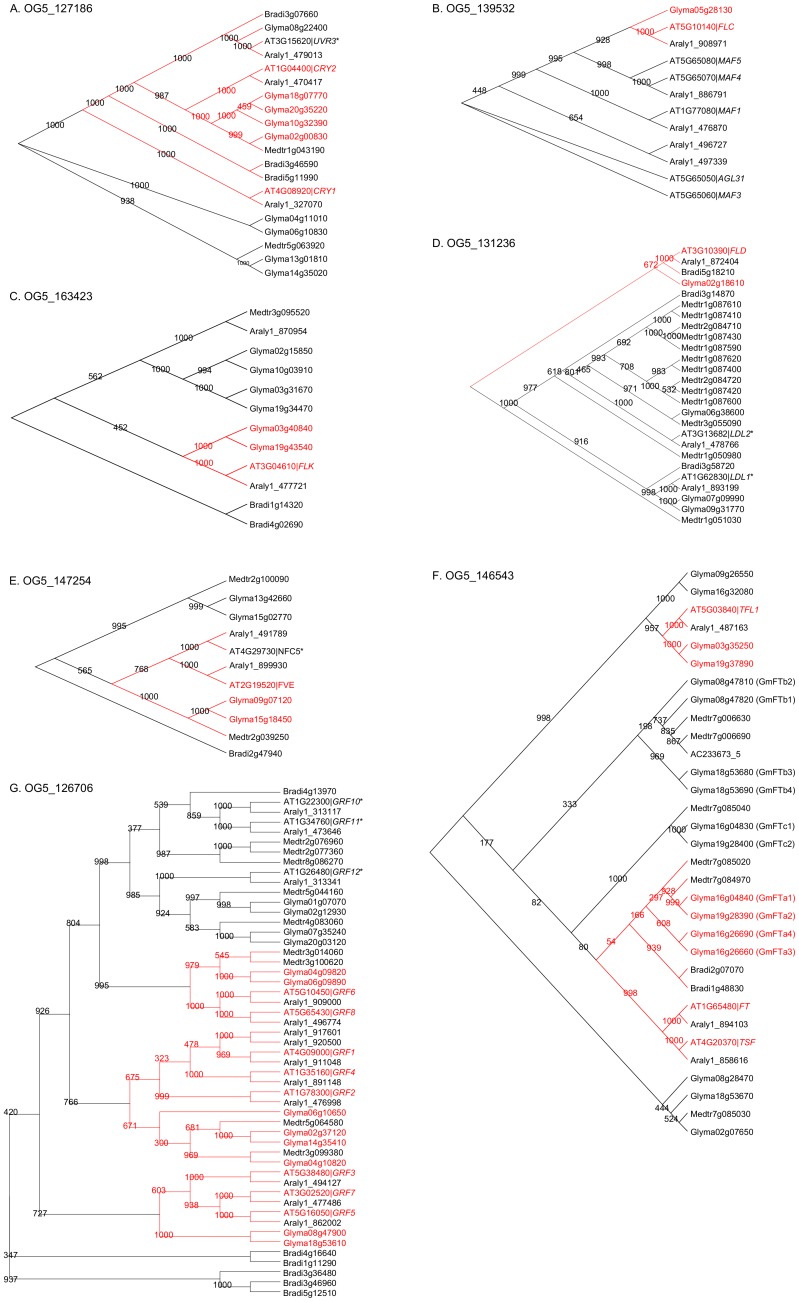
Phylogenetic relationship between soybean and Arabidopsis genes in each orthologue group. Arabidopsis genes that are known as flowering genes are shown in red along with their closest soybean homologues in the corresponding phylogenetic trees, which are most likely to be orthologues of Arabidopsis flowering genes. Numbers in each branch indicate the confidence value calculated from 1000 times of bootstrapping. Putative orthologues of *A. lyrata*, Medicago and Brachypodium are also included in the phylogenetic trees. Gene names starting with ‘AT’, ‘Glyma’, ‘Alyr_’ and ‘Bradi’ are for genes of *A. thaliana*, soybean, *A. lyrata* and Brachypodium, respectively. Medicago gene names start with either ‘Medtr’ or ‘AC’. To clarify relationships between different nodes diverse tree formats for OGs are used. The Arabidopsis genes marked with ‘*’ have not been identified and investigated as flowering genes. Phylogenetic tree for OG5_127186 (A), OG5_139532 (B), OG5_163423 (C), OG5_131236 (D), OG5_147254 (E), OG5_146543 and OG5_126706 (G) is shown.

The key Arabidopsis genes involved in the light signalling pathway include the *CONSTANS* (*CO*), *PHYTOCHROME* (*PHY*) and *CRYPTOCHROME* (*CRY*), *CIRCADIAN CLOCK ASSOCIATED 1* (*CCA1*), *LATE ELONGATED HYPOCOTYL* (*LHY*) and *PSEUDO-RESPONSE REGULATOR 1* [*PRR1*, also called *TIMING OF CAB EXPRESSION 1* (*TOC1*)] genes. *CO*, along with *CONSTANS-LIKE 1* (*COL1*) and *CONSTANS-LIKE 2* (*COL2*), are contained in OG5_156319, which also contains four soybean genes as soybean orthologues (Glyma08g28370, Glyma13g07030, Glyma18g51320 and Glyma19g05170) (See Table S1). All four soybean-orthologue candidates of Arabidopsis *CO* are expressed in tested tissues/developmental stages in the two recent transcriptome datasets [8], [9], but only two candidates are expressed in flowers (Dataset S2). The *CRY* genes *CRY1* and *CRY2* are grouped into OG5_127186, which contains nine soybean genes (Table S1). The *UV REPAIR DEFECTIVE 3* (*UVR3*) gene is also grouped into OG5_127186. In the phylogenetic tree of genes contained in OG5_127186, *CRY1*, *CRY2* and *UVR3* are all located in the same clade, along with 5 soybean genes (Figure 4A). Among these soybean genes, Glyma08g22400 is the closest orthologue of Arabidopsis *UVR3*, while Glyma18g07770, Glyma20g35220, Glyma10g32390 and Glyma02g00830 are closer to *CRY2* (Figure 4A). Phylogenetic trees in Figure 4 include putative orthologues in *Arabidopsis lyrata* (*A. lyrata*), *Medicago truncatula* (Medicago) as well as a monocot *Brachypodium distachyon* (Brachypodium). All three Brachypodium genes and one Medicago genes clustered in the same OG are also found in the *CRY1* clade, leaving four soybean genes and one Medicago gene in separate clades, indicating that these may have diverged functions (Figure 4A). Five *PHY* genes of Arabidopsis (*PHYA*, *PHYB*, *PHYC*, *PHYD* and *PHYE*) have eight soybean orthologue candidates, which are contained within OG5_136555 (Table S1). All of these soybean genes, except for Glyma15g23400, are expressed in flowers in one or both of the two transcriptome gene expression analyses integrated in this study [8], [9]. The MYB-transcription factor genes *CCA1* and *LHY* are among the key circadian clock components in Arabidopsis and are regulated by *TOC1* (also known as *PRR1*) [24]. *CCA1* has a single soybean gene orthologue candidate (Glyma07g05410), while *LHY* and *TOC1* have three and four soybean orthologue candidates, respectively (Table S1). All of the putative soybean orthologues of *CCA1*, *LHY* and *TOC1* are expressed in the samples tested, including flowers, when analysed for their *in silico* gene expression [8], [9]. The *GIGANTEA* (*GI*) gene in OG5_190821 is a part of the evening loop in Arabidopsis and performs different functions through its interactions with other genes, including the *FLAVIN-BINDING*, *KELCH REPEAT*, *F BOX 1* (*FKF1*), *LOV KELCH PROTEIN 2* (*LKP2*) and *ZEITLUPE* (*ZTL*) genes contained within OG5_150244, which contains six soybean genes in total (Table S1) [12]. Higgins *et al*. (2011) reported that *GI* is a highly conserved single copy gene in Arabidopsis, rice, *Brachypodium* and barley [12], but it has three orthologous soybean genes (Glyma09g07240, Glyma10g36600, Glyma20g30980) (Table S1).

#### Vernalisation pathway

Vernalisation involves plants that require prolonged periods of low temperature to initiate flowering. The vernalisation pathway in Arabidopsis involves 32 genes clustered into 23 OGs (Table 1). Among these, 30 Arabidopsis genes in 21 OGs have 81 soybean orthologue candidates (Table 1), of these 81 genes, 71 show evidence of transcription (Dataset S2). However, the orthologous counterparts of the Arabidopsis *VERNALISATION INSENSITIVE 3* (*VIN3*) gene in OG5_AT5G57380 and *AGAMOUS-LIKE24* (*AGL24*) gene in OG5_AT4G24540 were not identified in soybean (Table S1) by this method. Nonetheless, a BLAST analysis suggests the potential existence of their soybean orthologues (see below and the Discussion). Among the OGs containing Arabidopsis genes associated with the vernalisation pathway, the ratio of the number of soybean genes to that of Arabidopsis genes is highest in OG5_212406, in which the ratio is 14 soybean genes to 1 Arabidopsis gene, *REDUCED VERNALISATION RESPONSE 1* (*VRN1*) (Table S1). In contrast, the six Arabidopsis genes in OG5_139532, which includes a MADS-box transcription factor gene, *FLOWERING LOCUS C* (*FLC*), that negatively regulates flowering [25], share only one soybean gene as a putative orthologue (Glyma05g28130), resulting in the lowest soybean-to-Arabidopsis gene count ratio among the vernalisation-related OGs (Table S1). In the phylogenetic tree of OG5_139532, Glyma05g28130 is most closely related to *FLC* (Figure 4B). Interestingly, no Medicago and Brachypodium genes are found in this OG. As mentioned above, *VIN3* (in OG5_AT5G57380), which is a repressor of *FLC* in cold temperatures [26], and a flowering promoter gene, *AGL24* [27], [28], [29] [reviewed by Alexandre and Hennig (2008) [30]] in OG5_AT4G24540, are not assigned with putative soybean orthologues (Table S1), but share closely related soybean genes with other flowering genes (see below and Discussion).

#### Autonomous pathway

Autonomous pathways in plants are activated in response to endogenous changes that are independent from the environmental cues leading to flowering [31]. There are 17 genes, grouped into 16 OGs, involved in the Arabidopsis autonomous pathway (Table 1). Each OG has a single Arabidopsis gene that is known to be functional during floral initiation, except for OG5_129164, which contains two Arabidopsis flowering genes: *CURLY LEAF* (*CLF*) and *SWINGER* (*SWN*) (Table S1). Three other OGs (OG5_147254, OG5_127148 and OG5_131236) also include one or two additional Arabidopsis genes, raising the total number of Arabidopsis genes in the autonomous pathway-related OGs to 23 (Table 1). The total number of orthologous soybean genes to the 17 Arabidopsis genes (or 23 if the additional genes are included) is 49, of which 46 genes are transcriptionally active (Table 1). OG5_163423 has six soybean genes that are orthologous to AT3G04610 [*FLOWERING LOCUS KH DOMAIN* (*FLK*)], a repressor of *FLC* expression [32], which is the highest soybean-to-Arabidopsis gene count ratio among the OGs for autonomous pathways. The subsequent phylogenetic tree analyses revealed that only two soybean genes (Glyma03g40840 and Glyma19g43540) are located in the same clade with Arabidopsis *FLK*, indicating that they are likely true orthologues of *FLK* (Figure 4C). Similarly, in OG5_131236 and OG5_147254, only one (Glyma02g18610) and two (Glyma15g18450 and Glyma09g07120) soybean genes, respectively, are found and thus are also likely to be true orthologues of their Arabidopsis counterparts involved in autonomous pathways (Figure 4D,E). OG5_131236 has three Arabidopsis genes, including *FLOWERING LOCUS D* (*FLD*), which down-regulates *FLC* and has Glyma02g18610 as its closest orthologue according to the phylogenetic tree (Figure 4D), and *FVE* [also known as *MULTICOPY SUPPRESSOR OF IRA1 4* (*MSI4*)] in OG5_147254, which also down-regulates *FLC* and has Glyma15g18450 and Glyma09g07120 as its closest orthologues (Figure 4E). In comparison, four soybean orthologue candidates of Arabidopsis *FPA*, which has a redundant role with *FLD*, *FVE*, and *LD* [33], are equally distant from their Arabidopsis counterpart (data not shown). Because the minimum number of sequences for the generation of a phylogenetic tree is four, we are unable to generate phylogenetic trees for four OGs (OG5_128052, OG5_155119, OG5_169591 and OG5_170601) (Table S1). Therefore, all of the soybean genes in these OGs are regarded as the closest homologues of the Arabidopsis genes contained in the corresponding groups. Each of *VEL2*, *VEL3* and *VIN3* are grouped into a singleton OG and are not assigned orthologous counterparts in soybean (Table S1) but do have homologous genes in soybean according to the direct BLAST analysis (see below and Discussion).

#### Ambient temperature pathway

Plants respond to ambient temperature changes to modulate their flowering times [34]. The ambient temperature pathway in Arabidopsis involves 16 genes that are clustered into 8 OGs that have 38 soybean genes in total (Table 1). Three OGs (OG5_131236, OG5_147254 and OG5_155119) are also involved in autonomous pathways, and the Arabidopsis genes contained in OG5_139532, OG5_129661 and OG5_177438 are also involved in its vernalisation pathway (Table S1). In most of the OGs related to the ambient temperature pathway, the numbers of soybean genes are greater than those of Arabidopsis genes; however, the opposite findings are observed in the cases for OG5_139532 and OG5_190004. OG5_139532 contains six Arabidopsis genes (including *FLC*) that are orthologous to only one soybean gene, Glyma05g28130 (see above and Figure 4B). Similarly, Glyma01g36810 is the only soybean orthologue of the Arabidopsis genes AT4G35900 (*FD*) and AT2G17770 (*FDP*) in OG5_190004, which encode for the basic leucine zipper (bZIP) domain protein and positively regulate flowering [35]. Arabidopsis AT4G16280 (*FCA*) in OG5_155119 has one putative soybean orthologue (Glyma17g03960) (Table S1).

#### Meristem identity genes

Meristem identity genes are activated by upstream pathways and initiate floral development by triggering the transition of the apical meristem from the vegetative phase to the reproductive phase [12]. Seven Arabidopsis genes, including *FD*, *LFY*, *SQUAMOSA PROMOTER BINDING PROTEIN-LIKE 3* (*SPL3*), *AP1* and *AGL8* [also known as *FRUITFULL* (*FUL*)], are involved in this role and are clustered in five OGs (Table 1 and Table S1). The total number of soybean genes clustered within these OGs is 19, 18 of which were expressed in either the transcriptome data [8] or [9] (Table 1). *SPL3* in OG5_213105, which positively regulates *FT*, *AP1* and *LFY* in Arabidopsis [36], has four putative soybean orthologues (Glyma07g31880, Glyma13g24590, Glyma13g31090 and Glyma15g08270). Arabidopsis *LFY* (AT5G61850) in OG5_170760 has three orthologous counterparts in soybean (Glyma04g37900, Glyma06g17170 and Glyma20g19600) (Table S1). *AP1* and *CAULIFLOWER* (*CAL*), which are important in initiating flowering, are grouped into OG5_170388, which contains four soybean genes (Table S1). *AGL8* (or *FUL*) in OG5_211687, which is also important for the initiation of flowering [37], [38], has 7 putative soybean orthologues (Table S1).

#### Flowering pathway integrators

Genes of flowering pathway integrators integrate signals from several related pathways and determine the exact timing of flowering [2], [3]. In this study, 36 Arabidopsis flowering pathway integrator genes, including *FT*, *LFY*, *FLC*, *CO* and *SOC1*, were grouped into 11 OGs (Table 1 and Table S1). Among the 82 soybean genes grouped into these same 11 OGs, 69 genes demonstrated expression in at least one of the recent transcriptome datasets of SoyBase [8] or Libault *et al*. [9] (Table 1). As *FT*, *FLC*, *LFY* and *CO* are also involved in other flowering pathways, their relationships with soybean counterparts are described above. OG5_169591, which contains the *REDUCED VERNALIZATION RESPONSE 2* (*VRN2*) gene, includes two soybean genes (Glyma01g41460 and Glyma11g03960). OG5_146543 consists of three Arabidopsis genes [*FT*, *TWIN SISTER of FT* (*TFT*) and *TERMINAL FLOWER 1* (*TFL1*)] and 17 soybean genes. These 17 soybean genes include all 10 genes identified as soybean counterparts of Arabidopsis *FT* in two recent studies [6], [39], validating our approach for the identification of orthologues. The phylogenetic tree for OG5_146543 is also in agreement with that produced by Hecht and colleagues, which sub-classed 10 soybean *FT* homologues into *FTa*, *FTb* and *FTc* (Figure 4F) [39]. Eleven members of the NUCLEAR FACTOR Y transcription factors separate into three OGs (OG5_127594, OG5_128092 and OG5_152404) and have 34 soybean homologues (Table S1). The *SUPPRESSOR OF OVEREXPRESSION OF CO 1* (*SOC1*) in OG5_190130 and *EARLY FLOWERING 9* (*ELF9*) in OG5_128874 have four and three putative soybean orthologues, respectively (Glyma03g02200, Glyma07g08830, Glyma09g40230 and Glyma18g45780 for *SOC1*; Glyma03g27010, Glyma10g31450 and Glyma20g36110 for *ELF9*) (Table S1). OG5_126706 contains 11 Arabidopsis genes that encode 14-3-3 proteins that are involved in various processes, including signal transduction [40]; 8 of these 11 genes have demonstrated proven roles as flowering pathway integrators in previous studies (Table 1). In soybean, 12 genes are found in the same OG as the putative orthologues of these 11 Arabidopsis genes. In the phylogenetic tree analysis, the *GENERAL REGULATORY FACTOR 10* (*GRF10*), *GRF11* and *GRF12* genes branch out on their own along with four soybean genes (Figure 4G). Interestingly, these have not yet been investigated for their role as flowering genes even though they group together with eight other 14-3-3 protein genes (that are known as flowering genes) in OG5_126706.

### Comparison with Other Species

As *Medicago truncatula* is another important model species of the legume family that has also been extensively sequenced and studied, we applied the same methods used for the identification of orthologues of Arabidopsis genes to the annotated protein sequences of Medicago (Mt 3.5 annotation) (Dataset S1). Along with Medicago, we also applied the orthologue identification method to *Arabidopsis lyrata* (*A. lyrata*) and *Brachypodium distachyon* (Brachypodium). The numbers of putative orthologues of Arabidopsis genes involved in key flowering pathways in each species are shown in Table 1. Although the number of Medicago genes in the Mt3.5 annotation (version 3) is similar to that of soybean genes in G.max 1.09, smaller numbers of Medicago genes were grouped into the same OGs that contain key flowering genes of Arabidopsis. In particular, the numbers of putative Medicago orthologues of Arabidopsis genes involved in the light signalling pathways, meristem identity and coding for flowering pathway integrators were less than half of the number of soybean genes in these same pathways (Table 1). The number of Brachypodium and *A. lyrata* genes in each of OGs is also comparable with that of *A. thaliana*, even though the genome size varies from ∼130 Mb (*A. thaliana*) to ∼270Mb (Brachypodium) [41]. (Table 1).

### Comparison with *Glycine soja*

Recently, the draft genome sequence of *Glycine soja* (*G. soja*), wild soybean, was released [42]. As Kim *et al*. identified structural variations, such as large deletions, inversions and insertions, between *G. max* and *G. soja* genomes by mapping short reads of *G. soja* against the *G. max* genome sequence [42], we determined how many of the *G. max* genes involved in flowering pathways harbour such structural variations. Of the 1,538 genes in *G. max* associated with large deletions when promoter regions (the 1 kb region upstream) are taken into account, 10 genes are involved in flowering, 7 of which are involved in key flowering pathways. Similarly, 11 out of the 689 genes associated with large insertions are involved in flowering, but only 4 of them are associated with key flowering pathways (Table S3).Kim *et al*. also compared the single nucleotide polymorphisms (SNPs) in *G. soja* to those in the *G. max* genome [42]. A total of 4,187 SNPs were found within the genic regions of 405 *G. soja* genes that are counterparts of putative flowering genes of *G. max*. The number of flowering genes in *G. soja* that contain SNPs increases to 458 when the 1 kb upstream is included as the promoter region. However, a substantial number of these flowering genes, 182 genes (39.8% of 458 genes), have SNPs only in non-protein coding regions and/or promoters. For example, among the 17 *G. soja* genes corresponding to *G. max* genes that are homologous to the Arabidopsis *FT*, *TSF* and *TFL1* (OG5_146543) genes, only five have SNPs in CDSs: Glyma08g47820, Glyma16g04840, Glyma03g35250, Glyma09g26550 and Glyma16g32080. Of these, Glyma08g47820 and Glyma16g04840 were named as *GmFT6* and *GmFT3a*, respectively, by Kong *et al*. [6]. However, neither *GmFT6* nor *GmFT3a* expression is detected in either of the transcriptome datasets [8], [9].

A subsequent analysis of the proportions of genes containing structural variations or SNPs indicated that these mutations are not particularly enriched or depleted in flowering genes (data not shown).

### Soybean Genes with More or Less Paralogues than in Arabidopsis

#### Soybean flowering genes with more or less paralogues than in Arabidopsis

**Figure 5:**
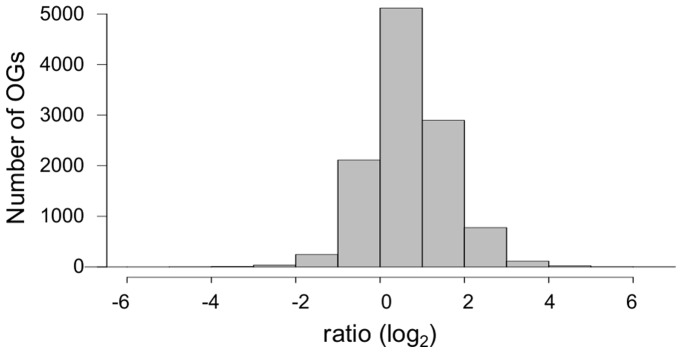
Ratio of soybean gene count to Arabidopsis gene count per OG. On average, the number of soybean genes per OG is 2-3 times of Arabidopsis genes, but 300 OGs have far more soybean genes (more than 2-standard deviation from average), hence designated as paralogue-rich OGs.

**Figure 6:**
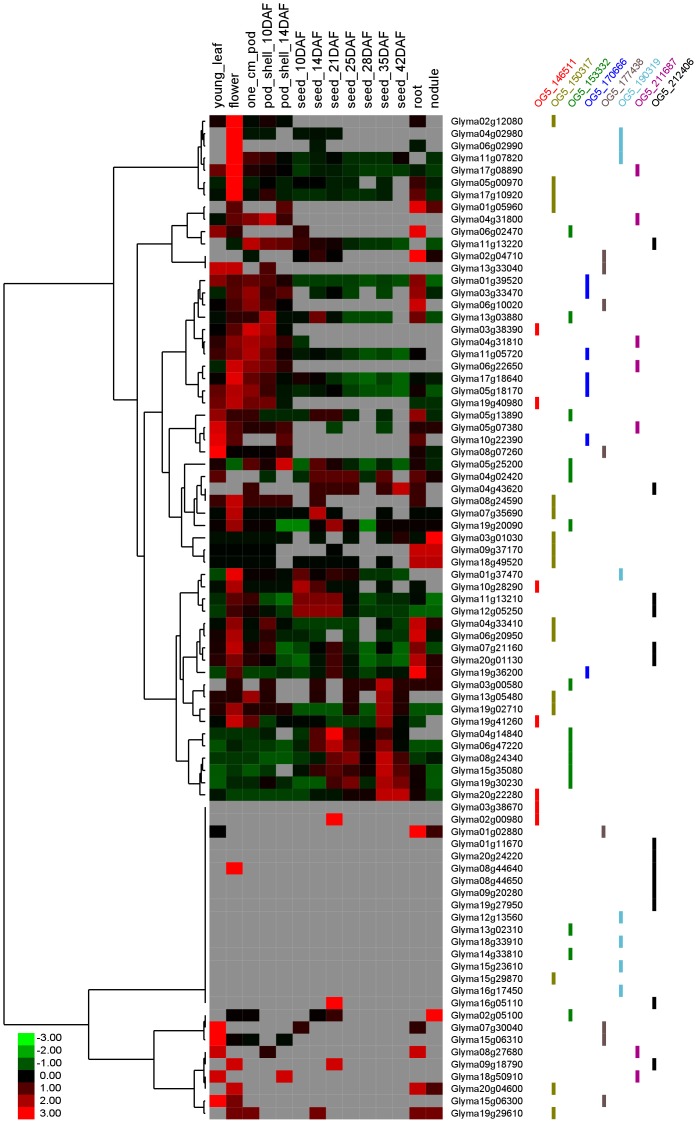
Expression profiles of soybean genes in eight paralogue-rich orthologue groups. The hierarchical clustering of the expression profiles for 82 soybean genes shows that genes in the same group do not always have similar expression patterns, indicating functional divergence among paralogues. The expression data were extracted from the soybean transcriptome data in SoyBase [8]. DAF: Days After Flowering.

We subsequently focused on the soybean OGs that are potentially involved in flowering pathways and have significantly more or less paralogues than the corresponding Arabidopsis genes (paralogue-rich and paralogue-less groups, respectively). As soybean underwent additional rounds of whole genome duplication events compared to Arabidopsis, the ratio of soybean gene counts against Arabidopsis gene counts per group is 2.5 on average, which suggests that each OG has 2-3 times more soybean genes than Arabidopsis genes (Figure 5). However, in 8 OGs, the number of soybean genes is far greater than that of Arabidopsis genes (Table 2). The Arabidopsis genes in these groups include *PIF3*, *CDF2/3*, *EEL*, *AREB3*, *AP2*, *AP3*, *SVP*, *AGL8* (or *FUL*) and *VRN1* (Table 2). The majority of the soybean genes in the paralogue-rich groups are transcriptionally active according to the two recent transcriptome datasets for soybean [8], [9] (Dataset S2). However, the hierarchical clustering of the expression profiles of the 82 soybean genes in these 8 paralogue-rich OGs is not in agreement with the gene grouping based on the sequence similarity (i.e., the grouping of OGs), suggesting that the paralogues have diverged in terms of function (Figure 6).

**Table 2:** List of paralogue-rich OGs containing Arabidopsis flowering genes.

OrthoMCL ID	Arabidopsis gene	Description	Soybean
OG5_146511	AT1G09530	*PIF3* (*PHYTOCHROME INTERACTING FACTOR 3*); DNA binding/protein binding/transcription factor/transcription regulator	**7**
OG5_150317	AT3G47500	*CDF3* (*CYCLING DOF FACTOR 3*); DNA binding/protein binding/transcription factor	**16**
	AT5G39660	*CDF2* (CYCLING DOF FACTOR 2); DNA binding/protein binding/transcription factor	
OG5_153332	AT2G41070	*EEL* (*ENHANCED EM LEVEL)*; DNA binding/transcription factor	**15**
	AT3G56850	*AREB3 (ABA-RESPONSIVE ELEMENT BINDING PROTEIN 3)*; DNA binding/transcription activator/transcription factor	
OG5_170666	AT4G36920	*AP2* (*APETALA 2*); DNA binding/transcription factor	**7**
OG5_177438	AT2G22540	*SVP* (*SHORT VEGETATIVE PHASE*); transcription factor/translation repressor, nucleic acid binding	**8**
OG5_190319	AT3G54340	*AP3* (*APETALA 3*); DNA binding/transcription factor	**8**
OG5_211687	AT5G60910	*AGL8 (AGAMOUS-LIKE8)*; transcription factor	**7**
OG5_212406	AT3G18990	*VRN1* (*REDUCED VERNALIZATION RESPONSE 1*); transcription repressor	**14**

In contrast to the results for the paralogue-rich OGs, the soybean gene counts are smaller than the Arabidopsis gene counts for the three paralogue-less OGs (OG5_139532, OG5_189849 and OG5_190004) (Table 3). Furthermore, there are 20 Arabidopsis flowering genes in 18 OGs that are not assigned putative soybean orthologues by our method (Table S1). Among these genes are Arabidopsis *VEL2*, *VEL3* and *AGL24*. *VEL2* and *VEL3* belong to a small gene family of plant homeodomain (PHD) finger-containing proteins that coordinate flowering through epigenetic regulation [43], [44], while *AGL24* is one of the MADS-box genes found to promote flowering by integrating flowering signals from several floral pathways [27], [28], [29]. However, BLAST searches of these Arabidopsis flowering genes against all of the annotated soybean genes reveal that they do have homologous soybean genes. In spite of this, the soybean genes that best match these Arabidopsis genes are putative soybean orthologues of other Arabidopsis genes (Table 4). Table 4 includes Arabidopsis genes in OGs that do not contain any other soybean gene members besides the best-matching soybean gene in the BLAST results. All of the soybean genes bearing a sequence similarity with Arabidopsis genes in OGs lacking other soybean gene members (with BLAST e-values less than 1e-10) are provided in the Dataset S4.

**Table 3:** List of OGs for flowering genes containing less number of paralogues in soybean.

OrthoMCL ID/soybean gene	Arabidopsis gene/description	PFAM	Panther
OG5_139532/Glyma05g28130	AT1G77080: *MAF1* (*MADS AFFECTING FLOWERING 1*); transcription factor	K-box region	MADS BOX PROTEIN
	AT5G10140: *FLC* (*FLOWERING LOCUS C*); specific transcriptional repressor/transcription factor	K-box region	MADS BOX PROTEIN
	AT5G65050: *AGL31* (*AGAMOUS LIKE MADS-BOX PROTEIN 31*); transcription factor	K-box region	MADS BOX PROTEIN
	AT5G65060: *MAF3* (*MADS AFFECTING FLOWERING 3*); transcription factor	K-box region	MADS BOX PROTEIN
	AT5G65070: *MAF4* (*MADS AFFECTING FLOWERING 4*); transcription factor	K-box region	MADS BOX PROTEIN
	AT5G65080: *MAF5* (*MADS AFFECTING FLOWERING 5*); transcription factor	K-box region	MADS BOX PROTEIN
OG5_189849/Glyma20g39140	AT1G50680: AP2 domain-containing transcription factor, putative	B3 DNA binding domain	-
	AT1G51120: AP2 domain-containing transcription factor, putative	B3 DNA binding domain	-
OG5_190004/Glyma01g36810	AT2G17770: *ATBZIP27*; transcription factor	-	-
	AT4G35900: *FD*; DNA binding/protein binding/transcriptionactivator/transcription factor	-	CYCLIC-AMP-DEPENDENT TRANSCRIPTION FACTOR ATF-6

**Table 4:** Arabidopsis flowering genes in OGs with no soybean genes members and their best BLAST-hit soybean genes.

Arabidopsis genes	Symbol/annotation	Best BLAST-hit soybean gene	OG ID for soybean gene	Arabidopsis genes in OG
AT5G67180	AP2 domain-containing transcription factor, putative	Glyma17g18640	OG5_170666	*AP2*
AT2G35670	*FERTILIZATION INDEPENDENT SEED 2* (*FIS2*)	Glyma01g41460	OG5_169591	*VRN2*
AT5G27220	protein transport protein-related	Glyma05g21790	OG5_170932+	AT5G48385
AT5G62040	*BFT* (*brother of FT and TFL1 protein*)	Glyma16g32080	OG5_146543	*FT;TSF;TFL1*
AT2G46790	*ARABIDOPSIS PSEUDO-RESPONSE REGULATOR 9* (*APRR9*)	Glyma04g40640	OG5_178368	*APRR5*
AT4G34000	*ABSCISIC ACID RESPONSIVE ELEMENTS-BINDING* *FACTOR 3* (*ABF3*)	Glyma02g14880	OG5_144915	*ABF1;ABF2;ABF4*
AT2G39250	*SCHNARCHZAPFEN* (*SNZ*)	Glyma15g04930	OG5_153389	*TOE1*
AT3G54990	*SCHLAFMUTZE* (*SMZ*)	Glyma15g04930	OG5_153389	*TOE1*
AT1G26790	Dof-type zinc finger domain-containing protein	Glyma18g49520	OG5_150317	*CDF3;CDF2*
AT1G69570	Dof-type zinc finger domain-containing protein	Glyma01g05960	OG5_150317	*CDF3;CDF2*
AT2G24790	*CONSTANS-LIKE 3* (*COL3*)	Glyma06g06300	OG5_144994	*ATCOL5;ATCOL4*
AT2G47700	zinc finger (C3HC4-type RING finger) family protein	Glyma20g38050	OG5_178422?	AT3G05545
AT2G18870	*VERNALIZATION5/VIN3-LIKE* (*VEL3*)	Glyma07g09800	OG5_190237	*VRN5*
AT2G18880	*VERNALIZATION5/VIN3-LIKE* (*VEL2*)	Glyma17g07000	OG5_170648	*VEL1*
AT3G30260	*AGAMOUS-LIKE 79* (*AGL79*)	Glyma16g13070	OG5_170388	*AP1;CAL*
AT4G16810	VEFS-Box of polycomb protein	Glyma01g41460	OG5_169591	*VRN2*
AT4G24540	*AGAMOUS-LIKE 24* (*AGL24*)	Glyma01g02880	OG5_177438	*SVP*
AT5G42910	basic leucine zipper transcription factor (*BZIP15*)	Glyma04g04170	OG5_144915	*ABF1;ABF2;ABF4*
AT5G57380	*VERNALIZATION INSENSITIVE 3* (*VIN3*)	Glyma17g07000	OG5_170648	*VEL1*
AT5G60120	*TARGET OF EARLY ACTIVATION TAGGED (EAT) 2* (*TOE2*)	Glyma12g07800	OG5_153389	*TOE1*

+ Not among the OGs containing Arabidopsis flowering genes.

#### Genomic distribution of genes for paralogue-rich groups and one genomic region harbouring numerous homologues of specific Arabidopsis genes

**Figure 7:**
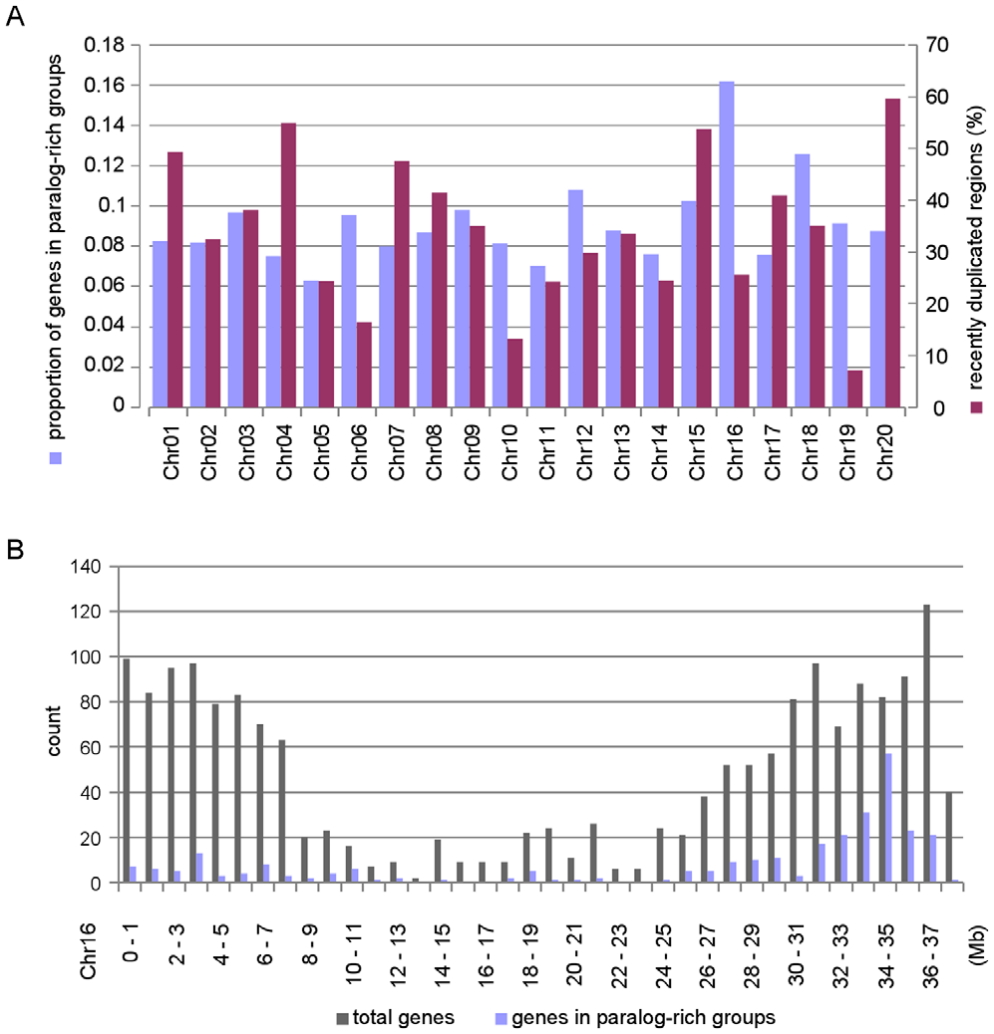
Distribution of genes in paralogue-rich groups in whole genome (A) and chromosome 16 (B). (A) Paralogue-rich orthologue groups and the percentage of recently duplicated segments per chromosome seems to have no apparent correlation. (B) In chromosome 16, a large number of the genes in paralogue-rich groups, mostly from OG5_134835 or OG5_170470, are condensed in the region around 34-35Mb (see the main text).

After we observed that some of the Arabidopsis flowering genes have more or less copies in soybean than average, we particularly expanded the investigation of paralogue-rich groups to the genomic scale. The 300 paralogue-rich OGs that have significantly more numbers of soybean genes (more than 2 standard deviations above the average) contain 4,236 soybean genes, which are spread evenly across the genome. On average, these genes comprise 6–16% of the total number of genes on each chromosome, and their fraction does not have any obvious correlation with the percentage of recently duplicated segments collected from the PHYTOZOME website (www.phytozome.net) (Pearson’s Correlation Coefficient: -0.08) (Figure 7A). However, soybean chromosome 16 (Chr16) is exceptionally enriched for genes belonging to paralogue-rich OGs, containing 292 genes, which is 16.2% of the total of genes on Chr16. In particular, 76 of these 292 genes are condensed within a 1.4 Mb region on Chr16 (Figure 7B). These 76 genes are associated with only two OGs: OG5_134835 and OG5_170470. The numbers of total paralogues of these OGs in soybean are 115 and 20, respectively, while Arabidopsis has only one (AT2G34930) and two genes (AT2G44290 and AT2G44300) in OG5_134835 and OG5_170470, respectively. AT2G34930 in OG5_134835 is known as a disease-resistance family protein and is involved in a defence response to fungus and signal transduction (TAIR, www.arabidopsis.org/servlets/TairObject?id=32293&type=locus). Two Arabidopsis genes in OG5_170470 (AT2G44290 and AT2G44300) are annotated as protease inhibitor/seed storage/lipid transfer protein (*LTP*) family proteins (AT2G44290: TAIR, www.arabidopsis.org/servlets/TairObject?id=33037&type=locus; AT2G44300: TAIR, www.arabidopsis.org/servlets/TairObject?id=33039&type=locus).

## Discussion

In this study, we identified potential soybean orthologues of most of the Arabidopsis genes with an emphasis on those that are involved in flowering pathways by associating soybean and Arabidopsis genes based on the current soybean annotation (G.max 1.09) in conjunction with the pre-defined Arabidopsis OGs in the OrthoMCL database [11]. Although the current soybean annotation assigned a closest Arabidopsis gene to nearly all of the predicted soybean genes, only 55% of the total Arabidopsis genes in the TAIR9 annotation are associated with soybean genes. However, the combined methodology used in this study, which incorporates the orthologue-group based results and the current soybean annotation, increased the number of Arabidopsis genes that are assigned with putative soybean orthologues to 20,730, which is more than 75% of the total Arabidopsis genes in the TAIR9 annotation. Nevertheless, it is difficult to determine the true soybean orthologues of Arabidopsis genes, especially when an OG contains multiple Arabidopsis genes. Thus, we resolved this by constructing phylogenetic trees for the candidates concerned. Conversely, the 25% of Arabidopsis genes that failed to have putative soybean orthologues by our analysis are not necessarily absent in soybean. In fact, all of the 20 Arabidopsis flowering genes that are not assigned with potential soybean counterparts have homologous soybean genes with significant e-values (<1e-10) when we subsequently performed BLASTP analyses with them (Table 4 and Dataset S4). However, the orthologue identification method used in this study determined that all of the top-matching soybean genes for these 20 Arabidopsis flowering genes (by BLASTP) were also candidate orthologues of other Arabidopsis genes. This finding suggests that the sequences concerned may have diverged beyond the sensitivity of our orthologue detection method; therefore, future functional analyses of these genes are necessary to confirm their orthology. In addition, it should be also noted that the false positive and false negative rates of OrthoMCL algorithm are 0.17 and 0.06, respectively, even though the OrthoMCL is among the best performing orthology identification tools [45]. Thus, failure of the orthologue identification for a subset of Arabidopsis genes may also due to the error in OrthoMCL-DB.

Using an approach that associates a group of soybean genes with a group of Arabidopsis genes that has the same OG ID also enabled us to investigate which soybean genes have a statistically higher or lower number of paralogues (or copies) in comparison to their Arabidopsis counterparts. For example, the analysis of paralogue-rich and paralogue-less soybean genes can be expanded to whole genes. Based on the near log-normal distribution of soybean-Arabidopsis gene count ratio per OG (mean: 1.03 and standard-deviation: 0.88 in log_2_), the number of soybean genes are much higher for 300 OGs (7 time or more than Arabidopsis genes) and smaller for 304 OGs (half or less than Arabidopsis genes) (Figure 5). A preliminary Gene Ontology analysis on the Arabidopsis genes in paralogue-rich and paralogue-less OGs (i.e., those that have far more and less copies in soybean than average, respectively) showed interesting features. Three hundred and ninety nine (399) Arabidopsis genes in 300 paralogue-rich OGs are enriched for ‘response to auxin stimulus (GO:0009733)’, ‘defense response (GO:0006952)’, ‘response to wounding (GO:0009611)’ and ‘lipid transport (GO:0006869)’ (Table S4). While the 1,946 Arabidopsis genes that have much less copies in soybean (paralogue-less OGs) are also enriched for ‘defense response (GO:0006952)’, they are mainly enriched for other GO terms, such as ‘intracellular signaling cascade (GO:0007242)’ (Table S4). The outcome raises questions as to whether the differential accumulation of gene copies between soybean and Arabidopsis is a possible evolutionary innovation that distinguishes the two species from one another.

**Figure 8:**
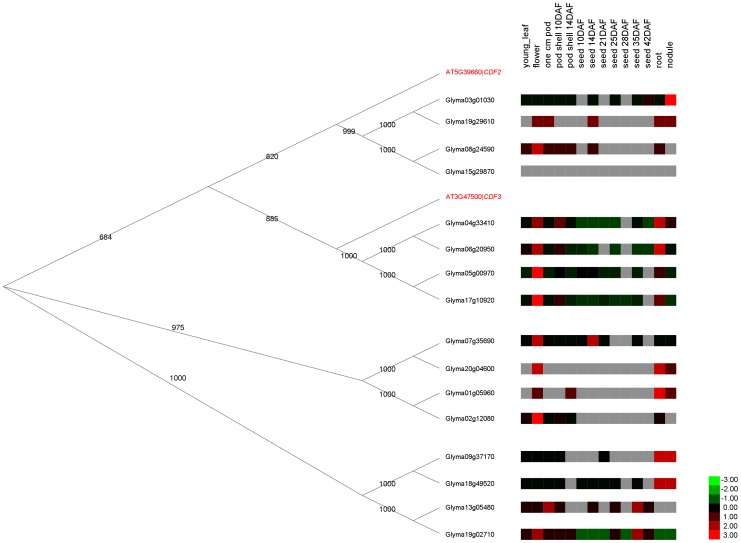
Expression patterns of soybean genes in OG5_150317. While the expression patterns of the 16 soybean genes in OG5_150317 were spread in different clusters as shown in Figure 6, but those that are in the same clade of the phylogenetic tree tend to have similar expression profiles. The expression data were extracted from the soybean transcriptome data in SoyBase [8]. DAF: Days After Flowering.

It is known that duplicated genes (i.e., genes in the same OG) do not necessarily retain the same functions. In our analysis, we found paralogues with diverged expression patterns as well as some more highly related paralogues located within the same clades in the phylogenetic tree and exhibiting similar expression patterns and, hence, more likely to retain similar functions (Figure 6 and Figure 8). Intriguingly, there exist some OGs that consist of soybean genes that are all non-tandem duplicates but with similar expression patterns (for example OG5_150317 in Figure 8). This begs the question as to how the soybean genome keeps copies of the same genes with presumably the same function in different places in the genome, especially because we observed no correlation between the fraction of genes in paralogue-rich OGs in each chromosome and the percentage of recently duplicated segments in the respective chromosomes (Figure 7A).

Soybean is a short-day species that does not require vernalisation to induce flowering [46]. It is, therefore, intriguing to observe that some of the Arabidopsis genes involved in the vernalisation pathway (*e.g.*, *FLC*, *VRN1* and *VRN2*) are represented in soybean. A previous study failed to identify any *FLC* genes, a key regulator of the vernalisation pathway, in *Medicago truncatula*, soybean, or *Lotus japonicus* [18], which can be attributed to the more incomplete genome sequence at the time; however, our analysis grouped Arabidopsis *FLC* along with 5 other Arabidopsis genes and one soybean gene (Glyma05 g28130) into the same OG (OG5_139532), in which the soybean gene and *FLC* have the closest relationship (Figure 4B). *VRN1* forms part of a chromatin-modifying polycomb that is involved in the methylation of histone 3 lysine 9 (H3K9) and histone 3 lysine 27 (H3K27) and, hence, the repression of *FLC* expression [47], [48]. *VRN1* belongs to a paralogue-rich OG that has far more soybean orthologue candidates than the majority of Arabidopsis genes, although other Arabidopsis genes that mediate vernalisation responsiveness, such as *VIN3*, are not assigned any putative soybean orthologues. *SVP* in OG5_177438, which encodes a MADS box transcription factor and is a negative regulator of flowering in Arabidopsis [49], has 8 putative soybean orthologues, while its close homologue *AGL24* is not assigned a candidate soybean orthologue. It is likely that the soybean genes in OG5_177438 may have acquired and replaced the function of *AGL24* because the most homologous soybean gene of Arabidopsis *AGL24* is one of the putative soybean orthologues of Arabidopsis *SVP* (Table 4).

Two microRNAs (miRNAs), miR156 and miR172, are involved in the regulation of *SQUAMOSA PROMOTER BINDING PROTEIN LIKE* family genes and *APETALA2-LIKE* (*AP2-like*) transcription factors in Arabidopsis [50] and are conserved in soybean [51]. Zhang *et al*. also concluded that the *SPL* family genes and *AP2-like* transcription factors are among the predicted target genes of miR156 and miR172, respectively, in soybean [51]. However, whether or not miR172 plays a similar role in soybean flowering still needs to be investigated.

The functional analysis of genes through reverse genetics approaches is more complicated for soybean than Arabidopsis. Computational analyses such as our study can therefore pinpoint the putative soybean orthologues of Arabidopsis genes with known functions. Indeed, putative soybean orthologues of *FT* and *TFL1* were first identified via computational analysis [18], [52]. This study determines all of the soybean genes that are putative orthologues of Arabidopsis genes by first grouping Arabidopsis and soybean genes into pre-defined orthologue groups and then associating genes in the same group from each species. This method determined not only the inter-species relationship of genes between soybean and Arabidopsis but also the intra-species relationships of genes in terms of their sequence similarities. Subsequently, the most probable soybean orthologues for Arabidopsis genes, especially those involved in key flowering pathways, were inferred through phylogenetic tree analyses. These inferences were also strengthened by referring to publicly available transcriptome datasets for the expression profile of the soybean genes. As more soybean transcriptome data becomes available in the future, it is expected that the putative soybean orthologues of Arabidopsis flowering genes can be more precisely compared by combining the data on their sequence similarities and expression patterns. Additionally, our methods found that 24 Arabidopsis genes, which had not been previously investigated for their roles in flowering, belong to OGs with known Arabidopsis flowering genes. Although sequence similarity does not always indicate functional similarity, these Arabidopsis genes may well be involved in the initiation of flowering.

In summary, our study has identified numerous floral regulatory candidate genes in soybean. Further studies of the genes identified here will provide a new perspective on the molecular processes underlying the floral transition process in soybean.

## Materials and Methods

### Sequence Data

The protein sequences of annotated soybean genes (G. max 1.09) were downloaded from the PHYTOZOME website (www.phytozome.net). The protein sequences of annotated Arabidopsis genes (TAIR9 release) were downloaded from The Arabidopsis Information Resource (TAIR) website (www.arabidopsis.org). The protein sequences of Medicago genes (Mt3.5 version 3) were downloaded from J. Craig Venter Institute website (www.jcvi.org). For those that have splicing variants, the longest isoforms were selected. Peptide sequences of *Brachypodium distachyon* were downloaded from PHYTOZOME website, then the longest peptide sequence for each locus was extracted for the analysis [41]. Peptide sequence of filtered *Arabidopsis lyrata* gene model which best represent each locus is downloaded from PHYTOZOME website [53].

### Soybean Homologue Identification and Grouping Orthologous Genes

The current soybean annotation information (G.max 1.09) associates 44,818 out of 46,367 soybean genes to 15,113 Arabidopsis genes. In order to identify soybean orthologues of more Arabidopsis genes, we assigned orthologue group (OG) IDs pre-defined in OrthoMCL database (release 5.0) [11] to Arabidopsis genes. OrthoMCL-DB is a list of OG IDs and the genes under same OG IDs from multiple species. OrthoMCL algorithm examines the all-versus-all BLAST search result and use the Markov Clustering algorithm to find interspecies homologues (orthologues) and intraspecies homologues (paralogues) [11]. Soybean genes that have homologous Arabidopsis genes in the current annotation information were given the same OG ID with the corresponding Arabidopsis genes, and those that do not were assigned OG IDs by OrthoMCL. An OG IDs was assigned to each gene, if possible, via the web-based tool in the OrthoMCL website (http://www.orthomcl.org), which considers the BLAST-hit quality of the input protein sequence to the protein sequences in the OrthoMCL-DB to find the OG containing the closest protein to the input. For the 317 Arabidopsis genes that have homologous soybean genes according to the current soybean annotation but do not have OG IDs assigned by OrthoMCL, arbitrary OG IDs were given, which are ‘OG5_’ followed by the Arabidopsis gene locus name (e.g., OG5_AT2G33835 for an Arabidopsis gene AT2G33835). Among these, 9 groups have Arabidopsis flowering genes, which are AT2G33835, AT2G18880, AT2G18870, AT3G30260, AT4G16810, AT4G24540, AT5G57380, AT5G42910 and AT5G60120. Then, for each OG, the soybean gene members were regarded as putative soybean orthologues of Arabidopsis gene members.

### Transcriptional Activity and Gene Expression Data Analysis

The expression profiles of the 491 putative soybean orthologues of Arabidopsis flowering genes were extracted from SoyBase [8] and the integrated transcriptome atlas of soybean generated by Libault *et al*. (2010) [9]. For the SoyBase data, a transcriptionally active gene is a gene that has at least two or more sequence reads at one or more of the tested tissues/developmental stages. For the dataset of Libault *et al*. (2010) [9] any gene that has normalized read counts greater than 0 at least in one tissue/developmental stage is regarded as a transcriptionally active gene. The hierarchical clustering of the expression patterns was performed by CLUSTER 3.0 using the Z-scores of the normalized read counts (bonsai.ims.u-tokyo.ac.jp/∼mdehoon/software/cluster), and the clustered results were visualized by Java Treeview (http://rana.lbl.gov/downloads/TreeView/TreeView_vers_1_60.exe).

### Multiple Alignment and Phylogenetic Tree Generation

Multiple sequence alignment was carried out by MUSCLE 3.8.31 [54], and phylogenetic trees were generated by CLUSTALW 2.0.12 [55] with the bootstrap option. Dendroscope 2.7.4 [56] was used for the graphical representation of the phylogenetic trees.

### Identification of Closer Soybean Orthologues of Arabidopsis Flowering Genes within OGs

The phylogenetic tree information for between all Arabidopsis and soybean genes within the same OG in Newick format was parsed to decide which soybean genes are closer homologues to Arabidopsis genes involved in flowering pathways using in-house Python scripts. Any soybean gene located in the same clade with Arabidopsis flowering genes is regarded as a soybean homologue involved in flowering pathways, unless the soybean gene is equally close or closer to another Arabidopsis gene that are not explicitly involved in flowering pathways. For the small OGs (less than 4 sequences in total), all soybean genes are regarded as close orthologues of corresponding Arabidopsis flowering genes if the OG has only Arabidopsis flowering genes.

## Supporting Information

Figure S1 **Hierarchically clustered expression profiles of 491 soybean genes homologous to Arabidopsis flowering genes.** Expression data were extracted from the soybean transcriptome data in SoyBase (A) [8] and that by Libault *et al*. (B) [9]. Z-scores for expression levels were used for the clustering. Grey indicates no expression. DAF: Days After Flowering; RH: Root Hair; HAI: Hours After Inoculation; IN: inoculation; UN: mock-inoculation; Strip: stripped.
(TIF)

Table S1 **List of OGs containing flowering pathways genes in Arabidopsis.**
(PDF)

Table S2 **Soybean flowering genes preferentially or specifically expressed in flower.**
(PDF)

Table S3 ***G. max*** **genes containing large structural variation in comparison with** ***G. soja*** **.**
(PDF)

Table S4 **Gene Ontology analysis on soybean genes of different categories.**
(PDF)

Dataset S1 **Orthologue groups in Arabidopsis, soybean, Medicago,** ***Arabidopsis lyrata*** **and Brachypodium.**
(XLS)

Dataset S2 **Transcriptional activities of the 491 putative soybean flowering genes.**
(XLS)

Dataset S3 **Phylogenetic trees for OGs that contain four or more sequences including Arabidopsis flowering genes.**
(PDF)

Dataset S4 **Arabidopsis genes that are not assigned putative soybean orthologues and their closest soybean genes from BLAST analysis.**
(XLS)
