# Supplementary figures and images for: Comparative Genomic Analysis of Soybean Flowering Genes
Source: PLoS One. 2012 Jun 5;7(6):e38250. doi: 10.1371/journal.pone.0038250 (PMC3367986; doi:10.1371/journal.pone.0038250)

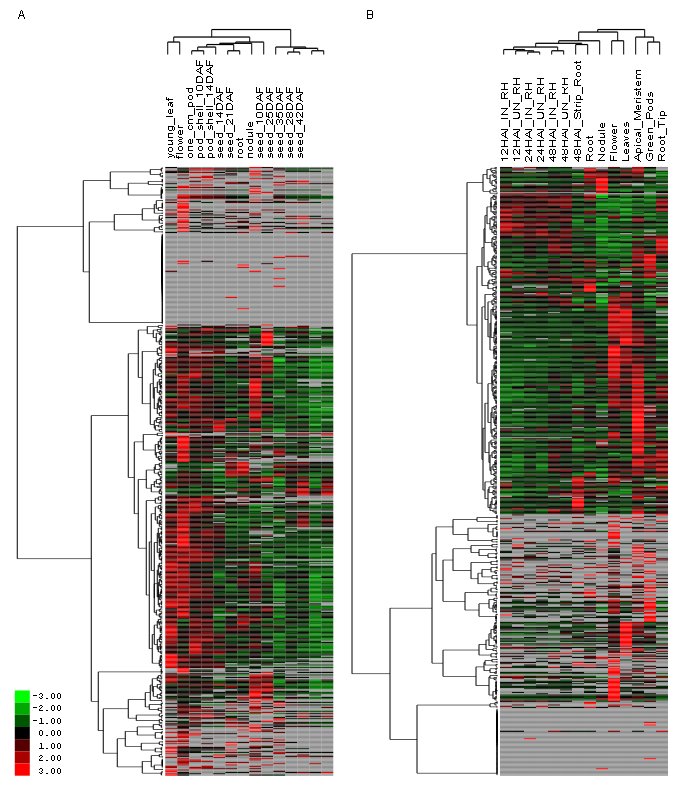

Supplement: Figure S1 — Hierarchically clustered expression profiles of 491 soybean genes homologous to Arabidopsis flowering genes. Expression data were extracted from the soybean transcriptome data in SoyBase (A) [8] and that by Libault et al. (B) [9]. Z-scores for expression levels were used for the clustering. Grey indicates no expression. DAF: Days After Flowering; RH: Root Hair; HAI: Hours After Inoculation; IN: inoculation; UN: mock-inoculation; Strip: stripped. (TIF) [file pone.0038250.s001.tif]

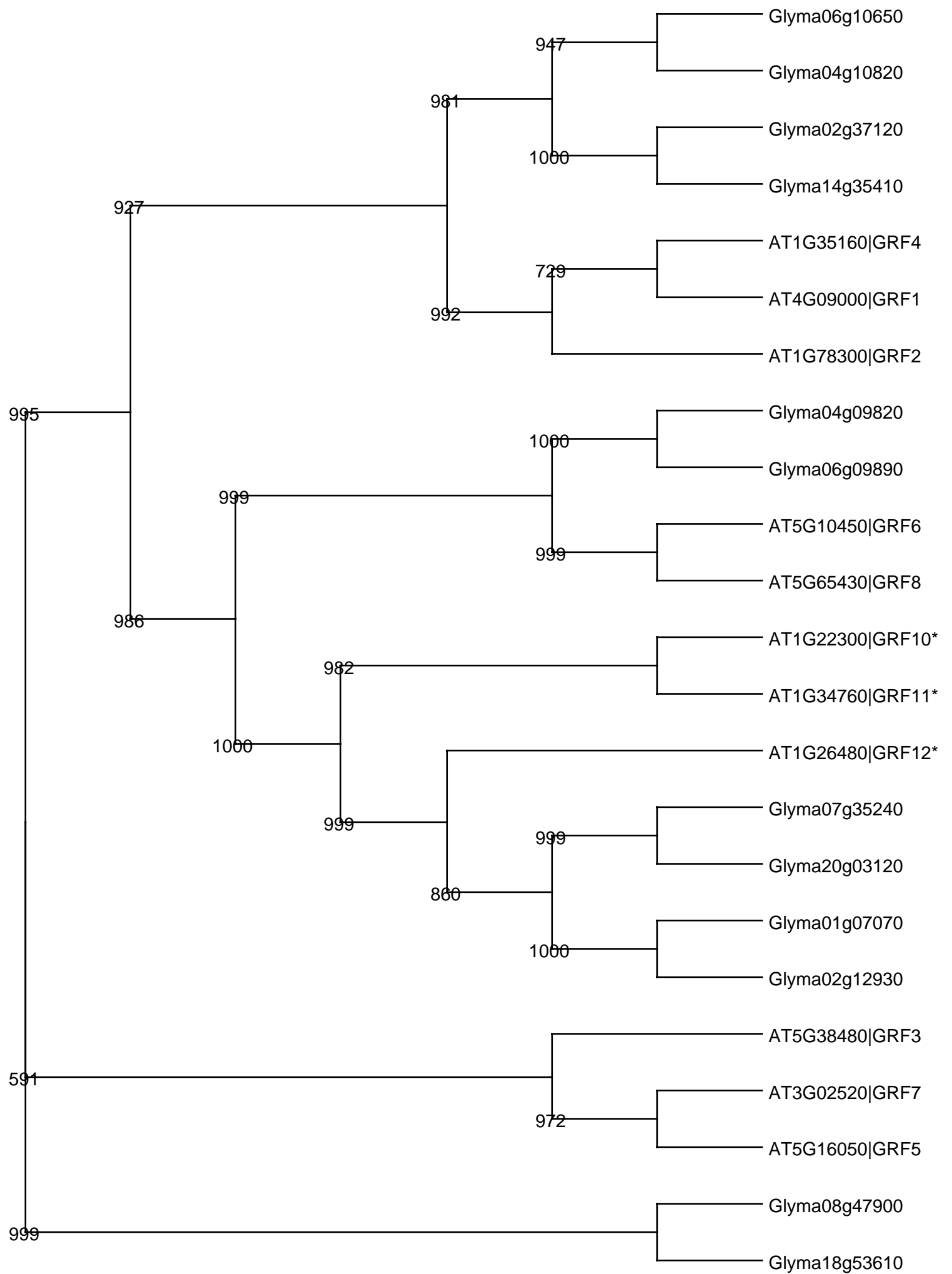

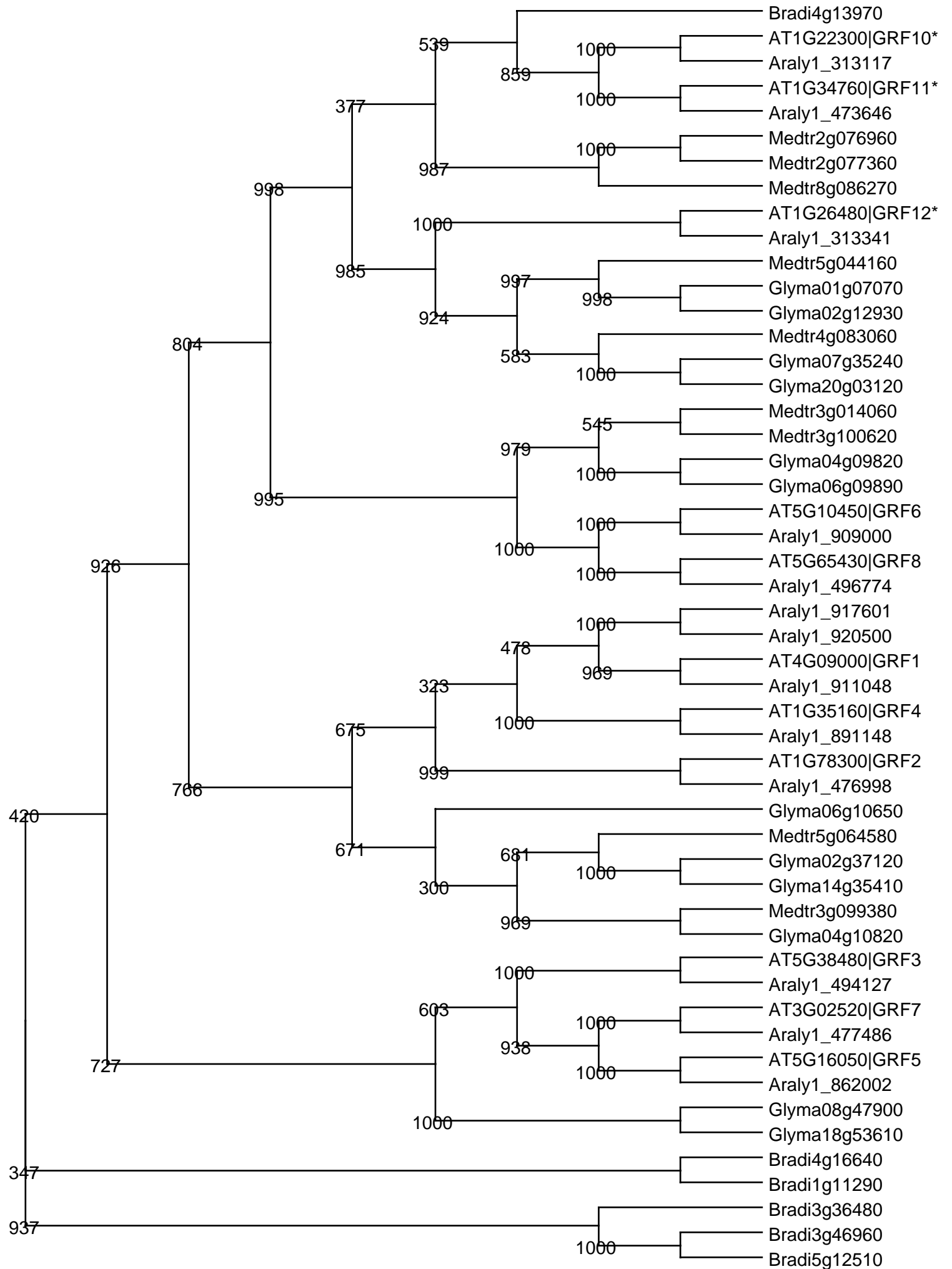

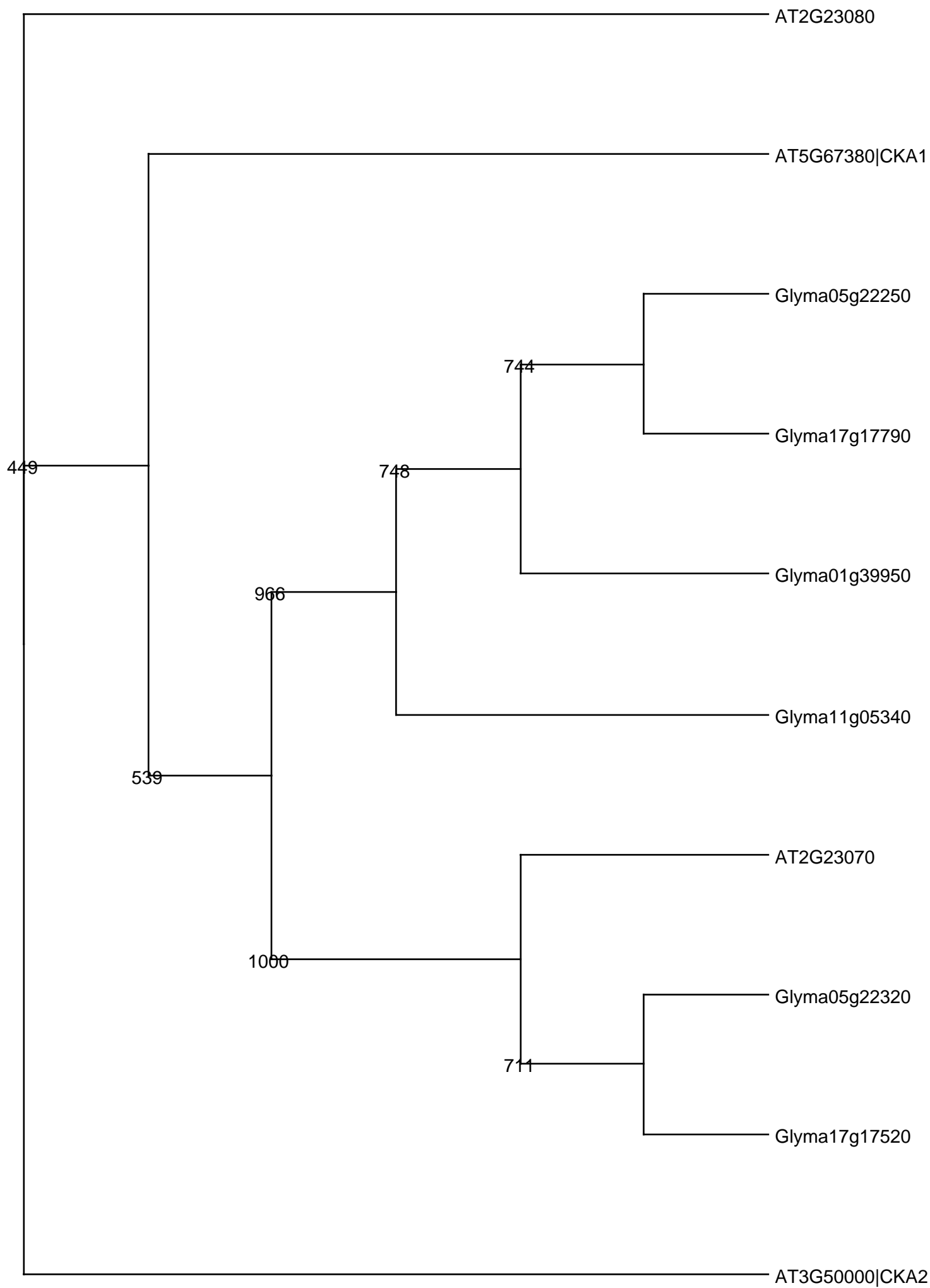

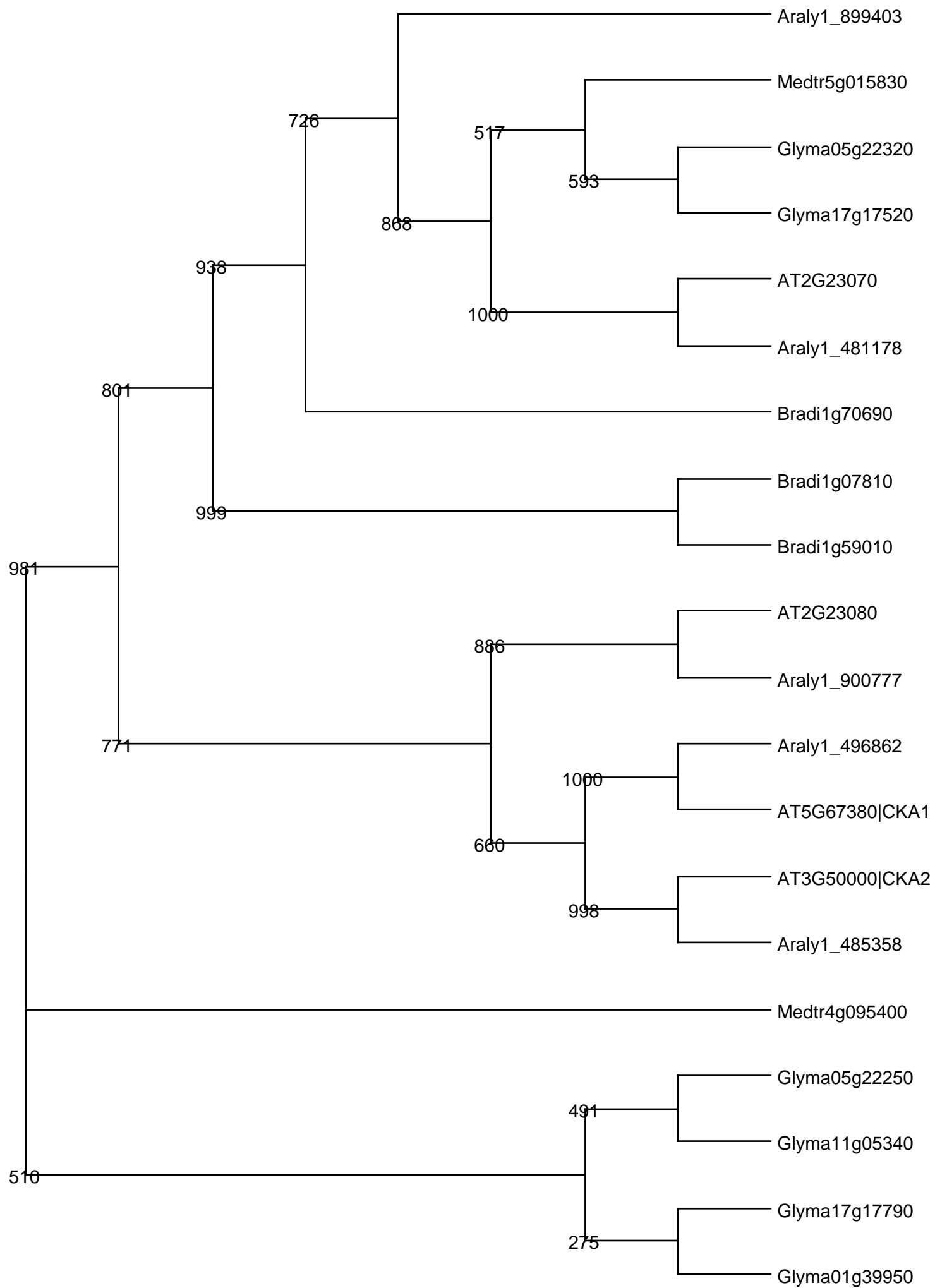

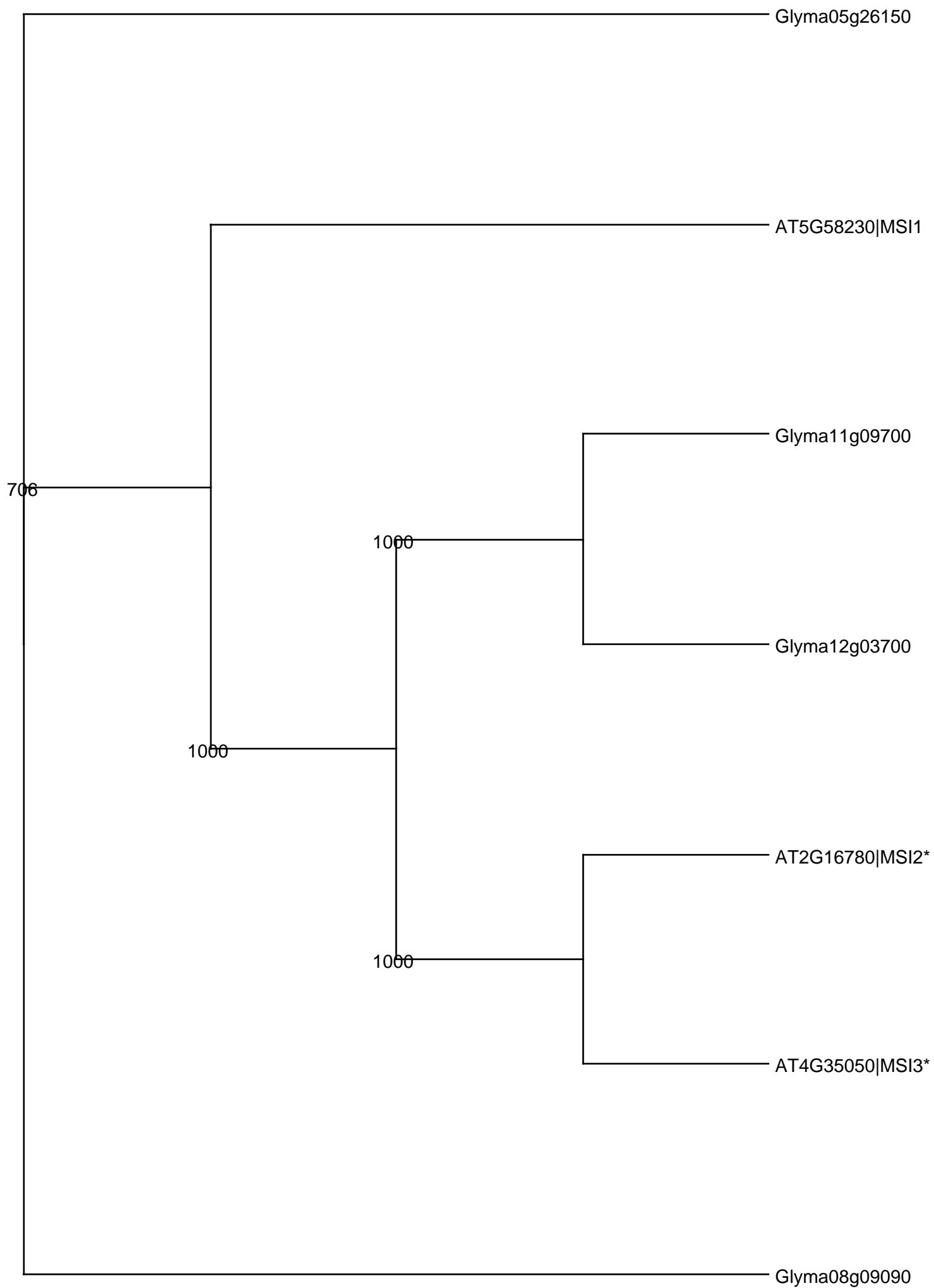

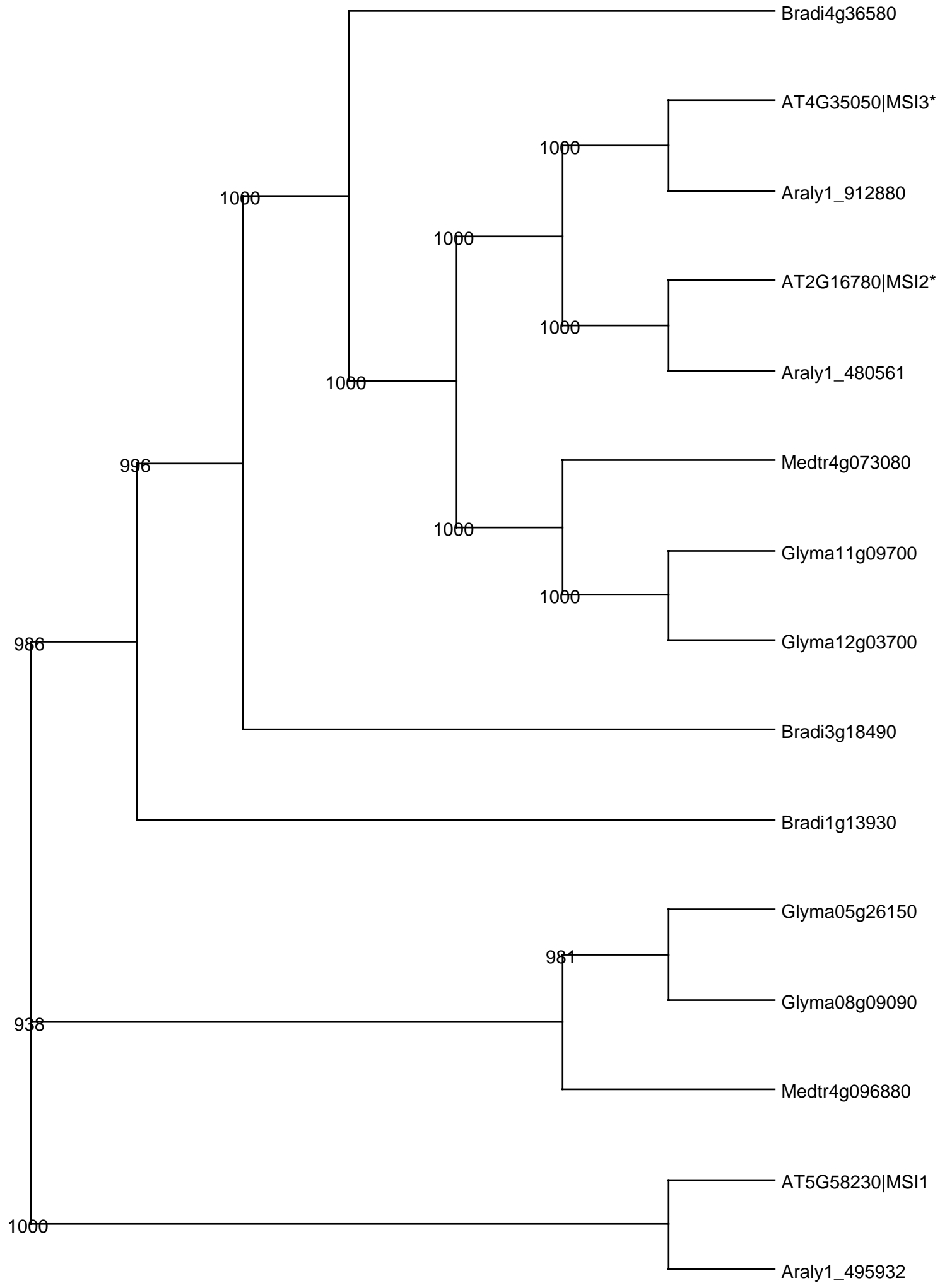

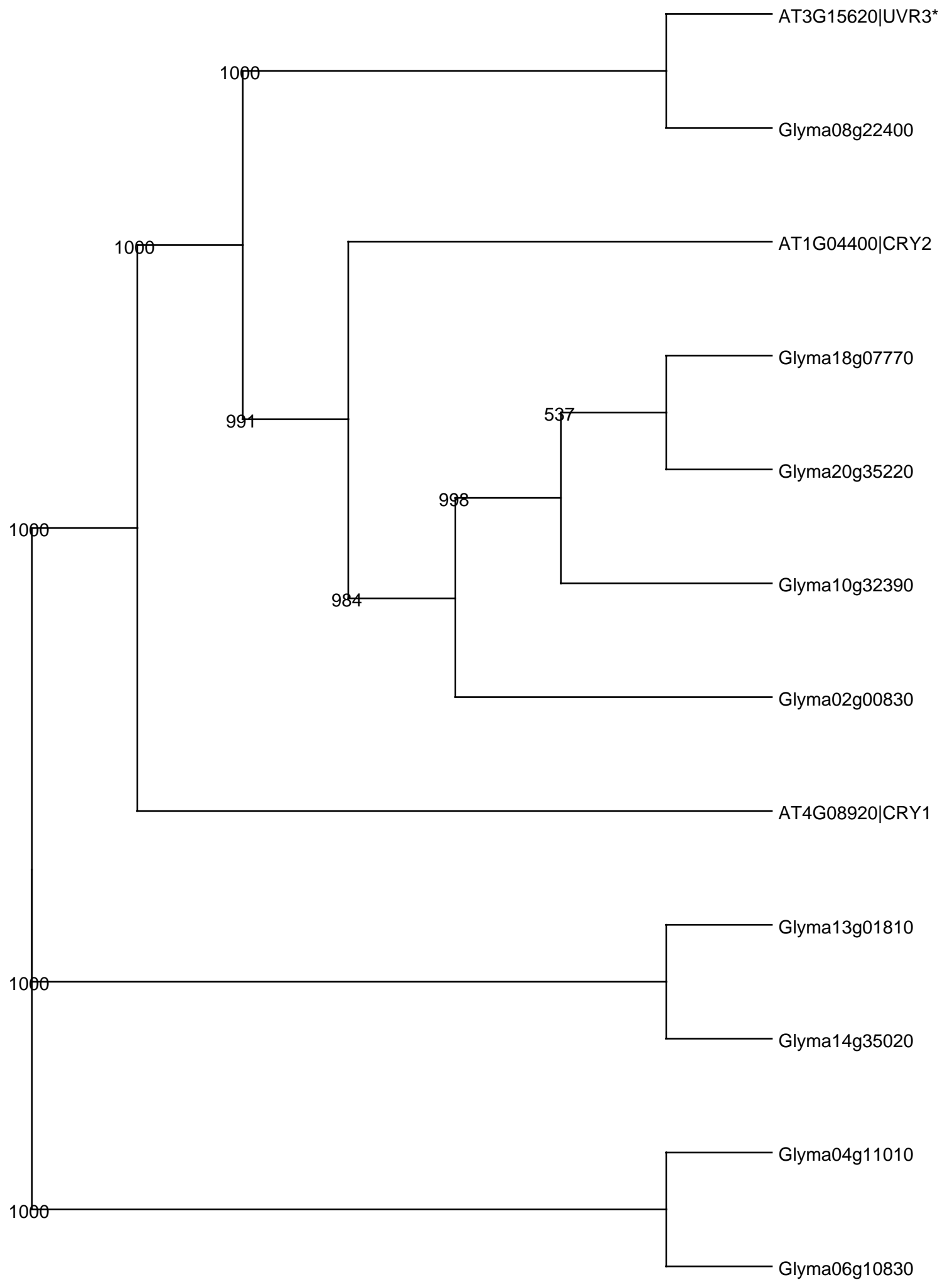

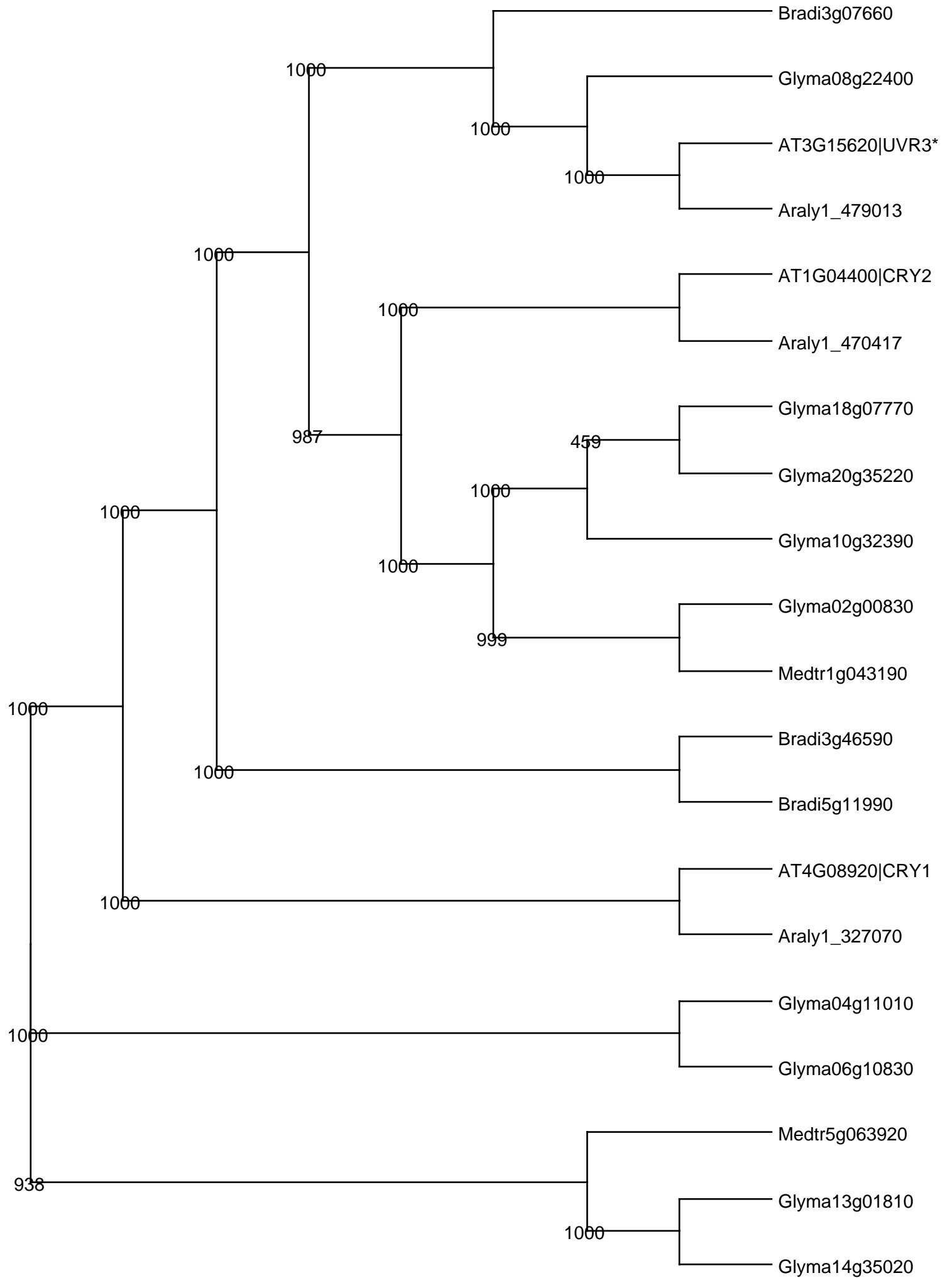

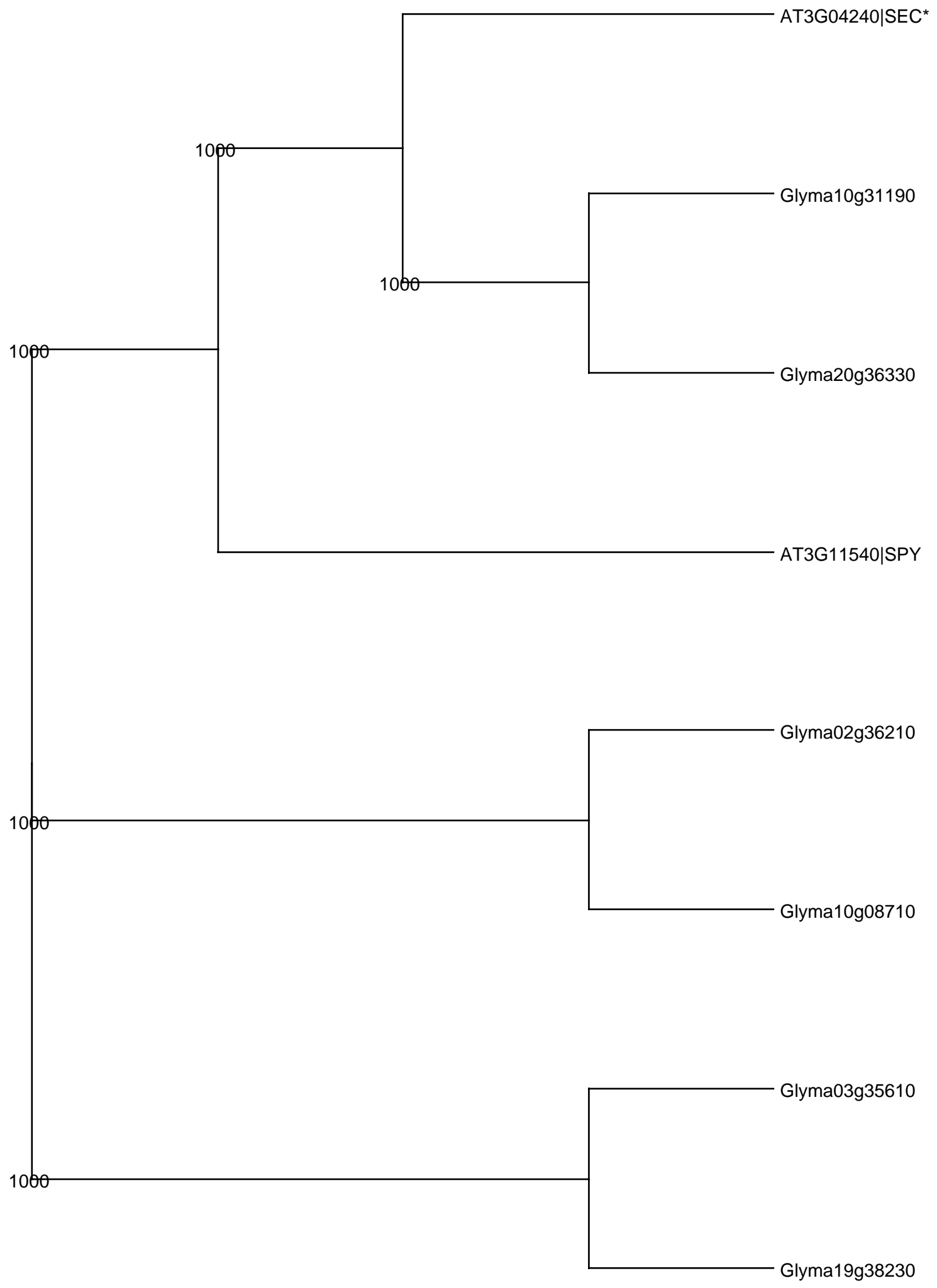

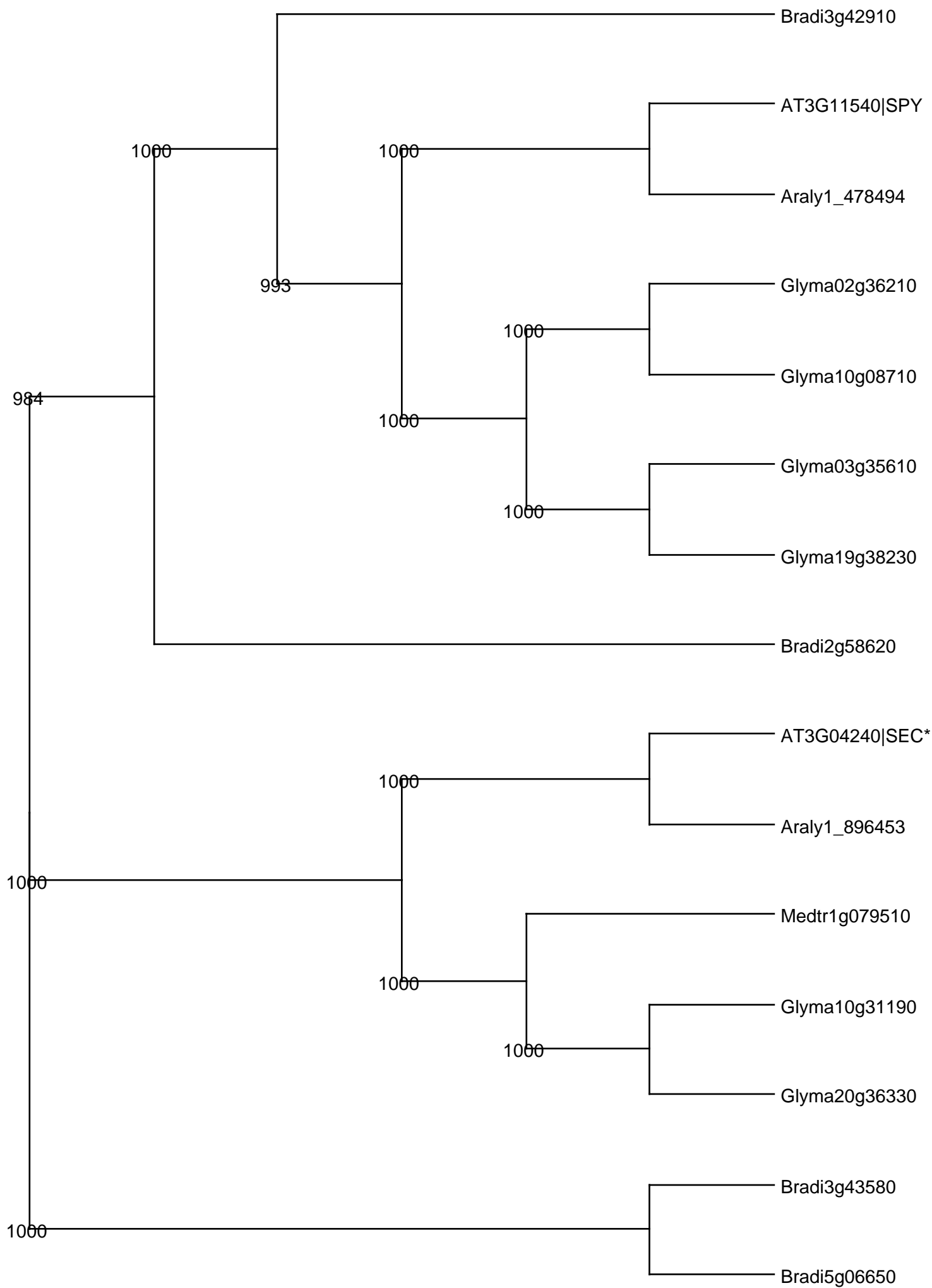

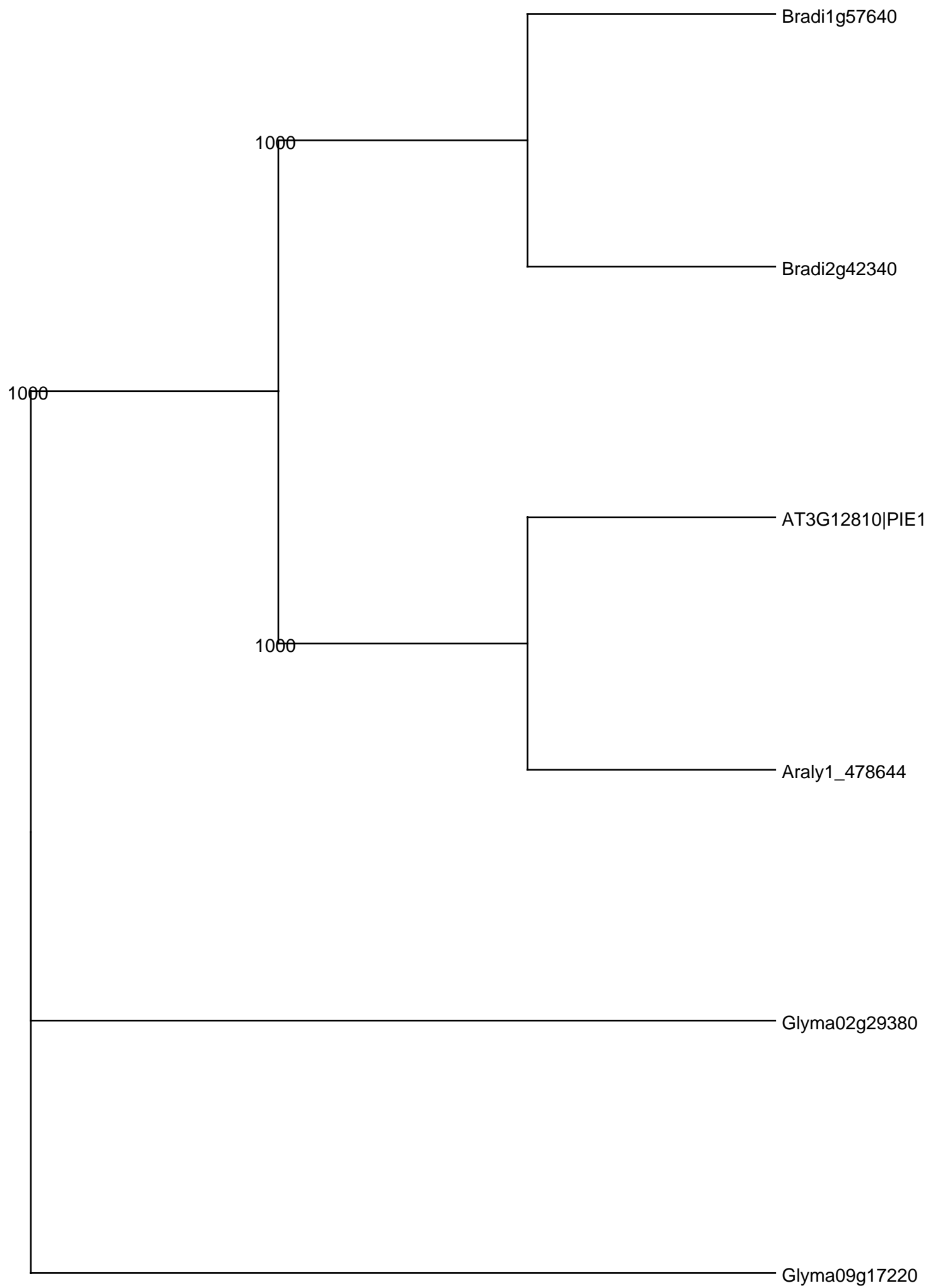

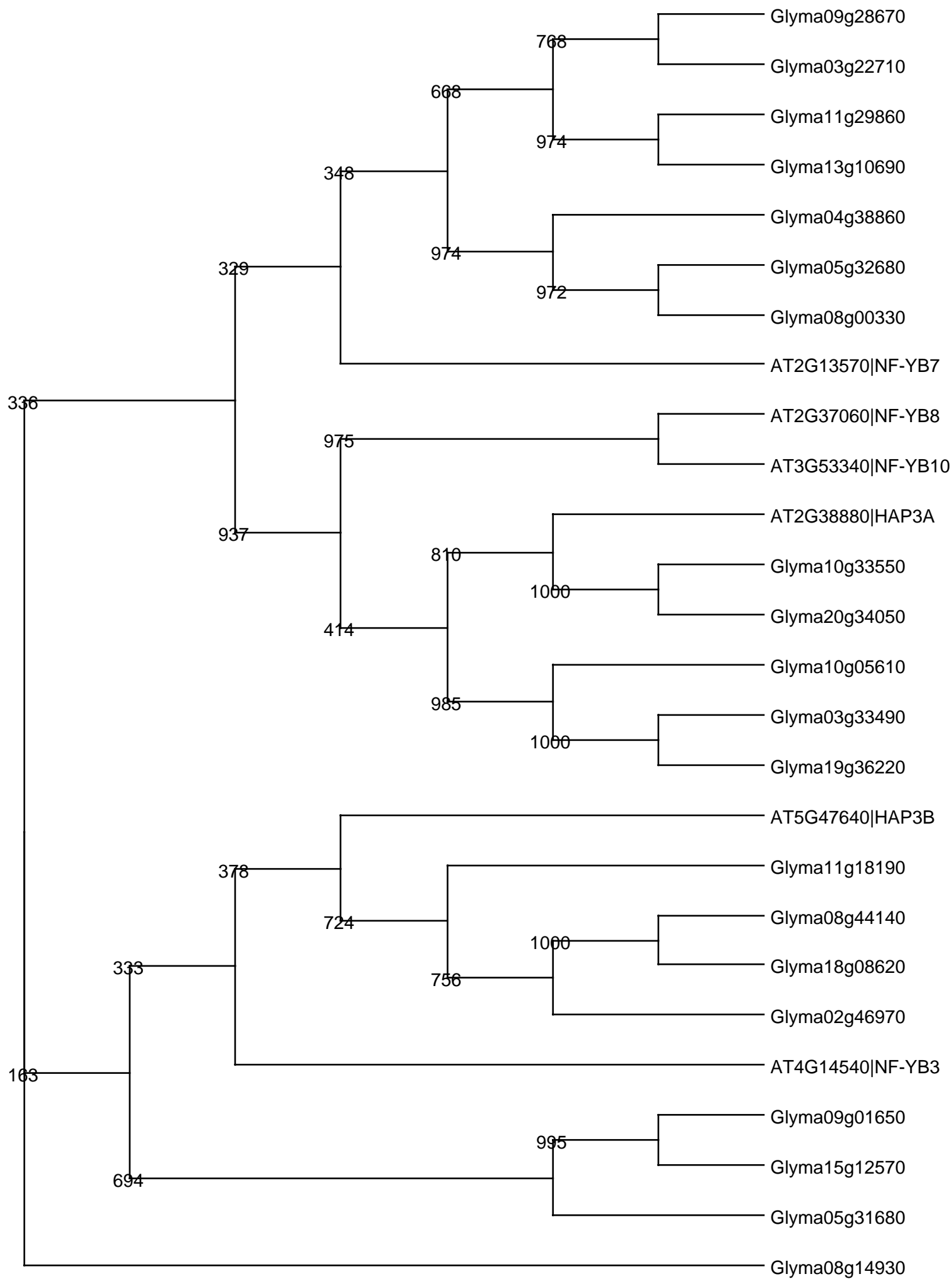

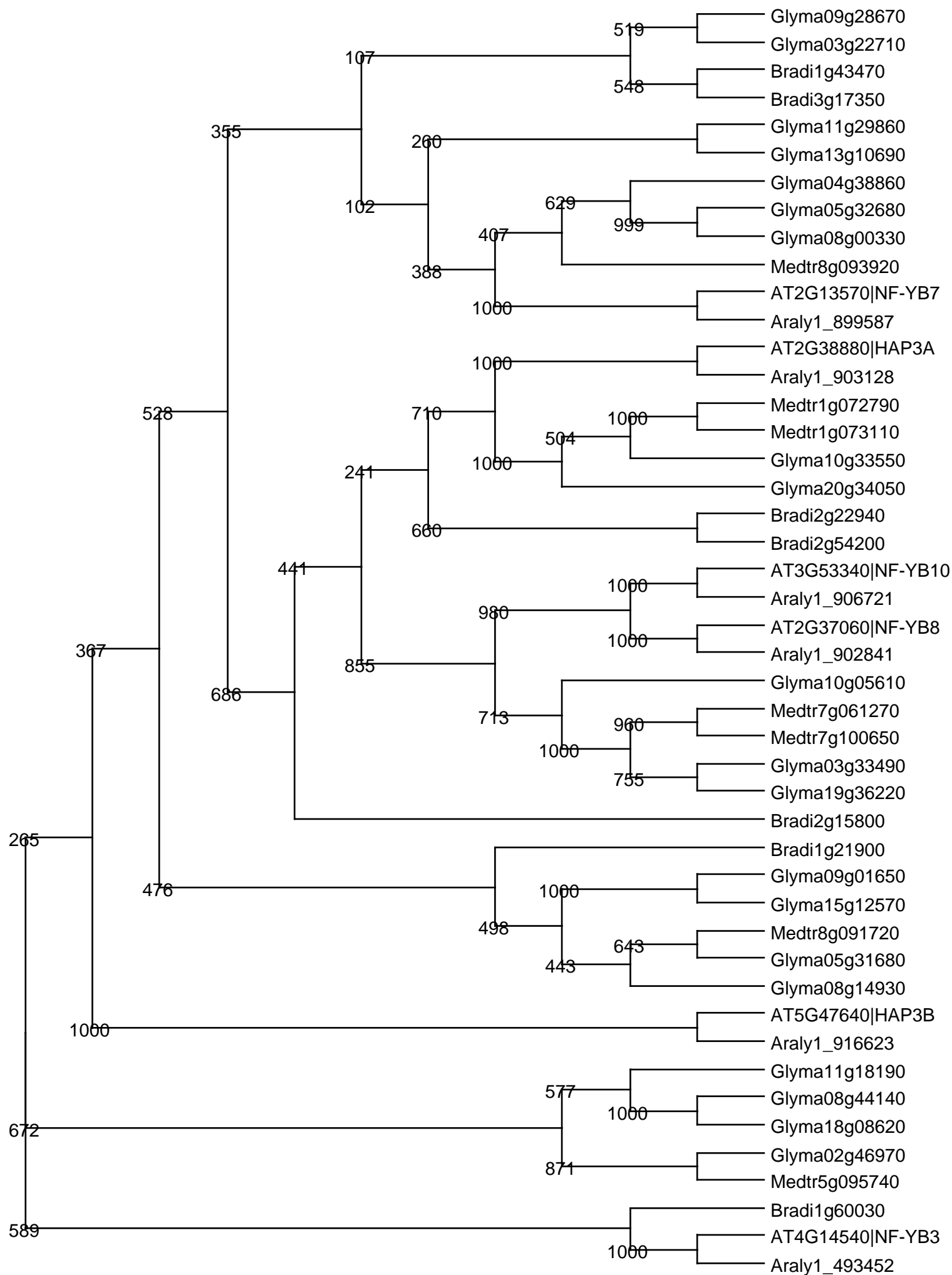

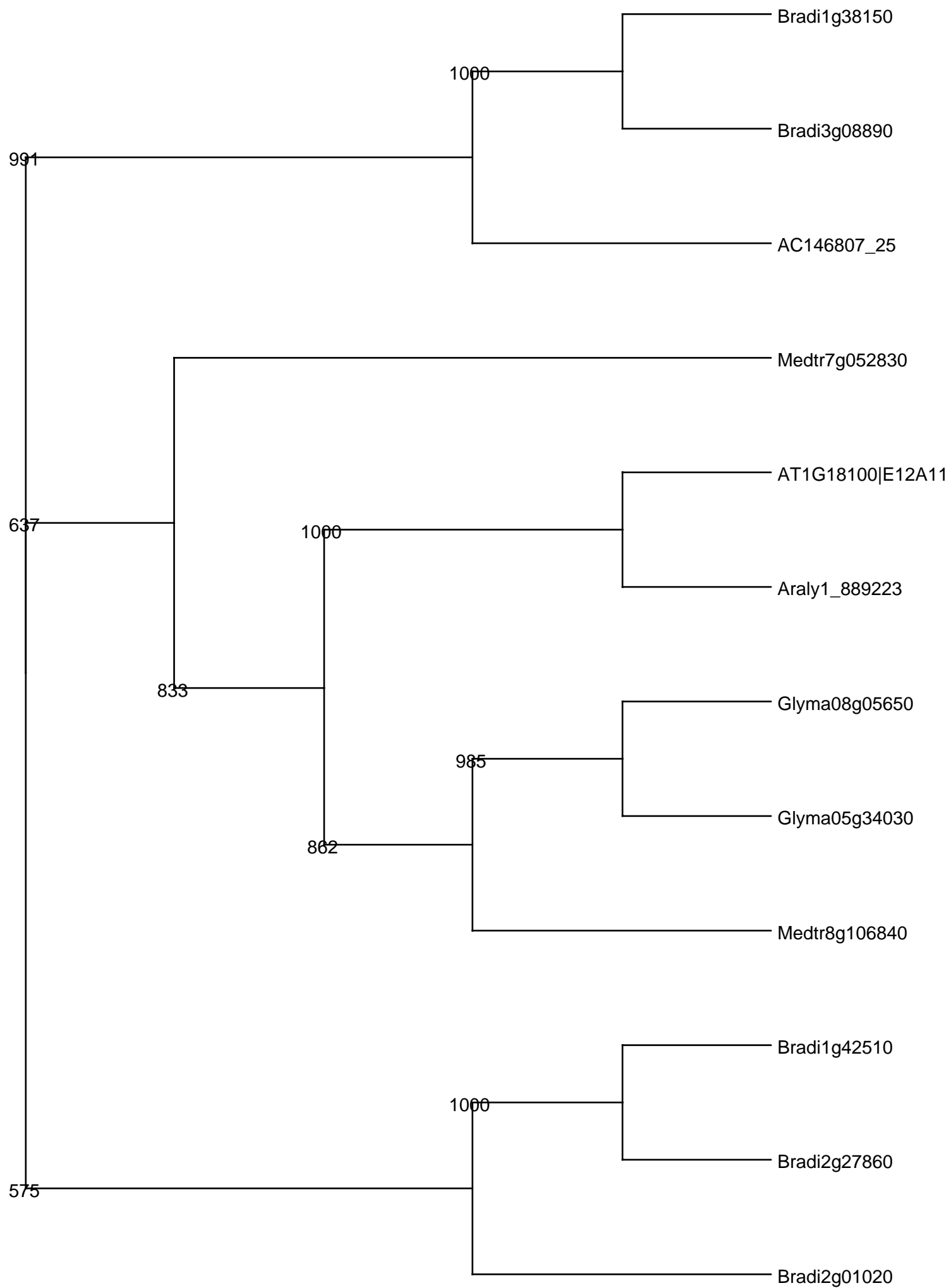

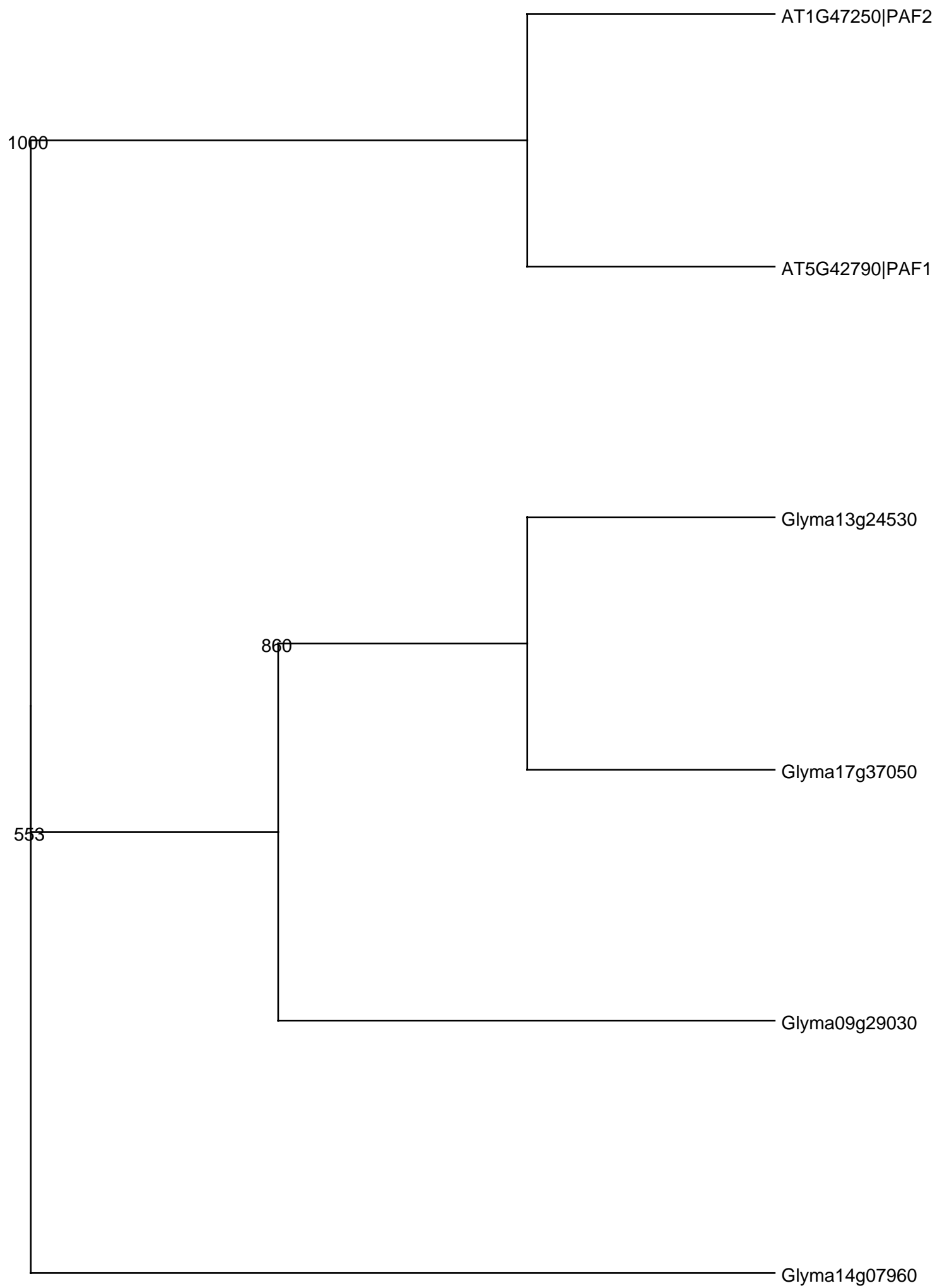

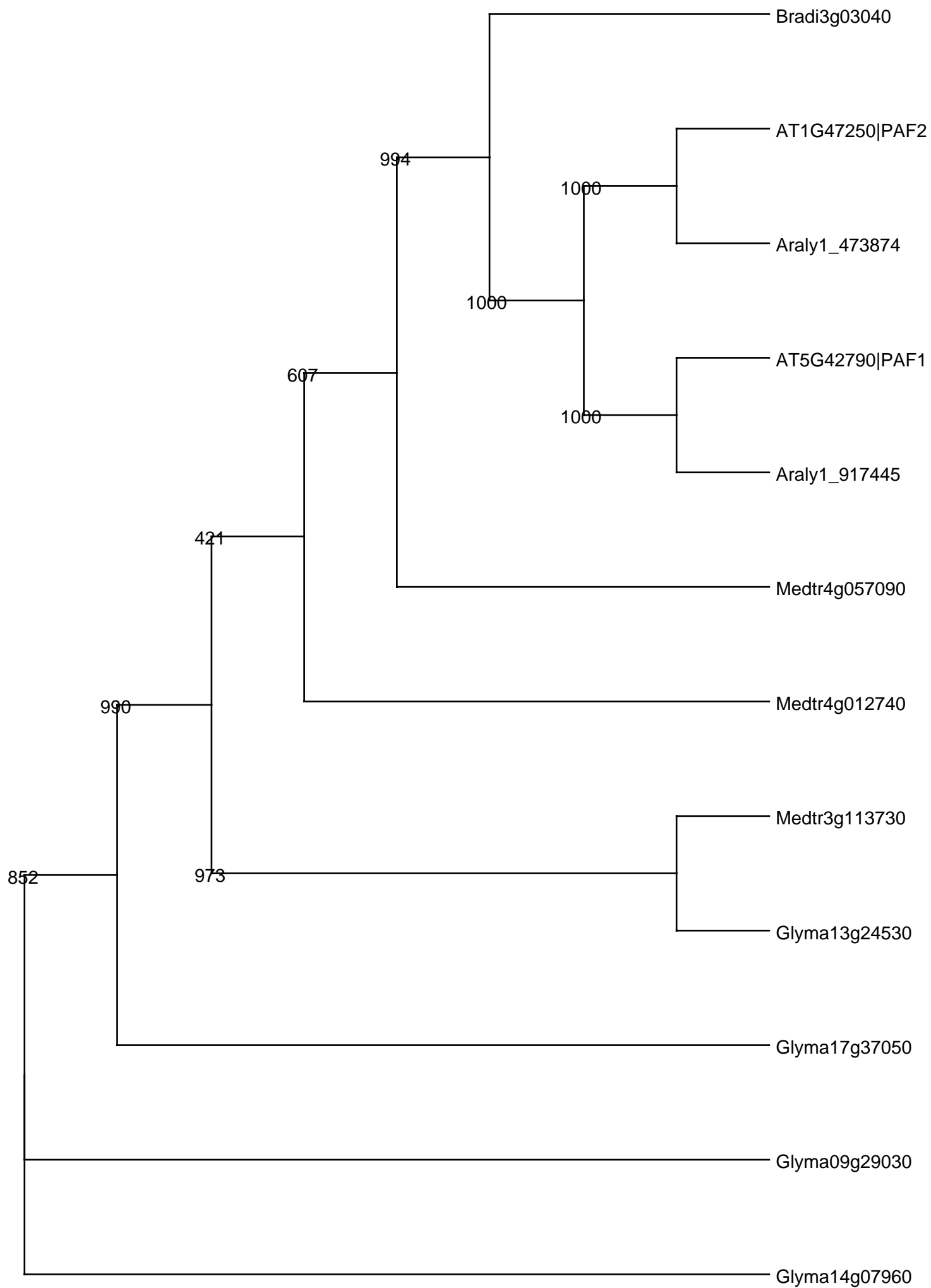

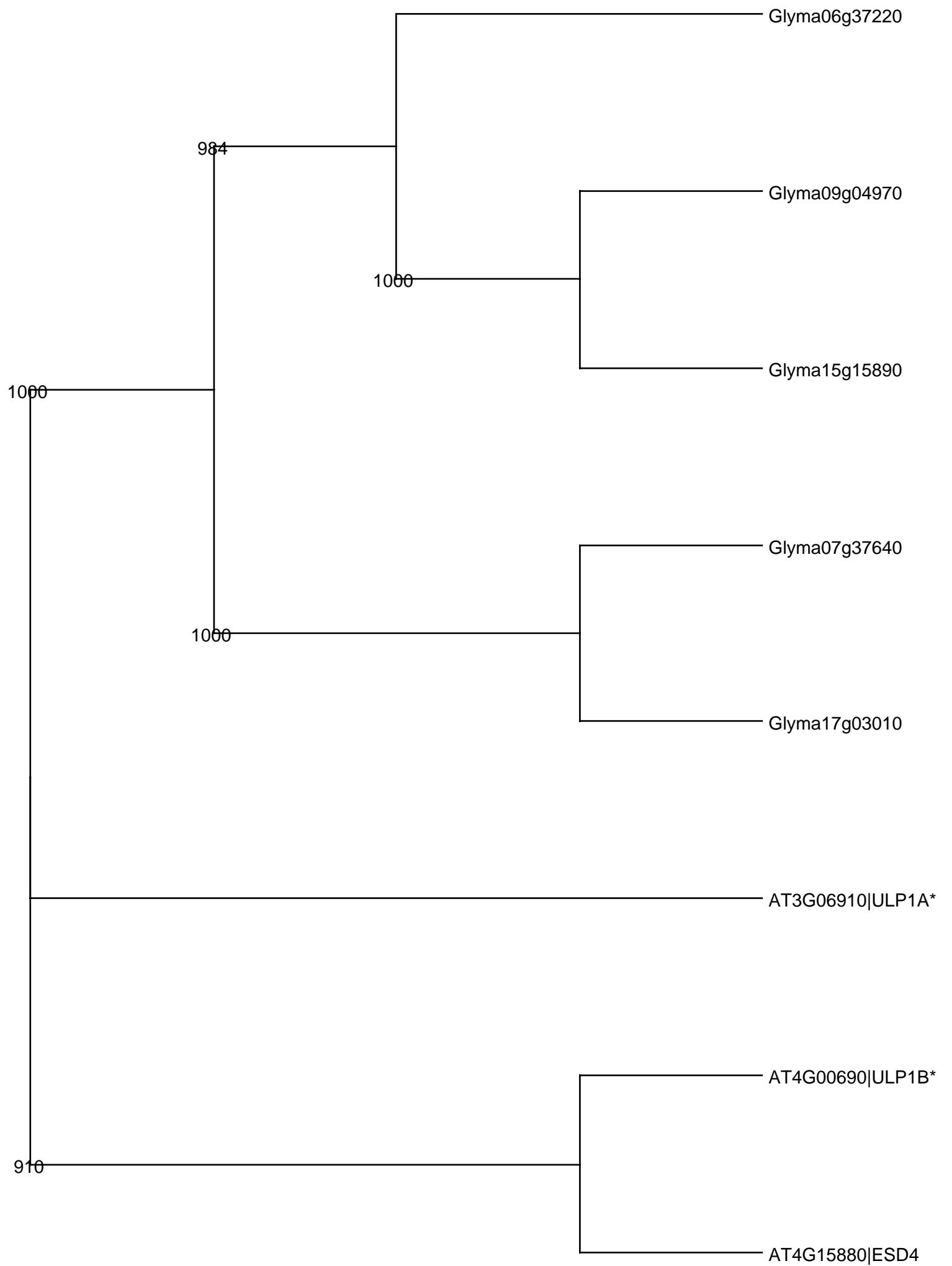

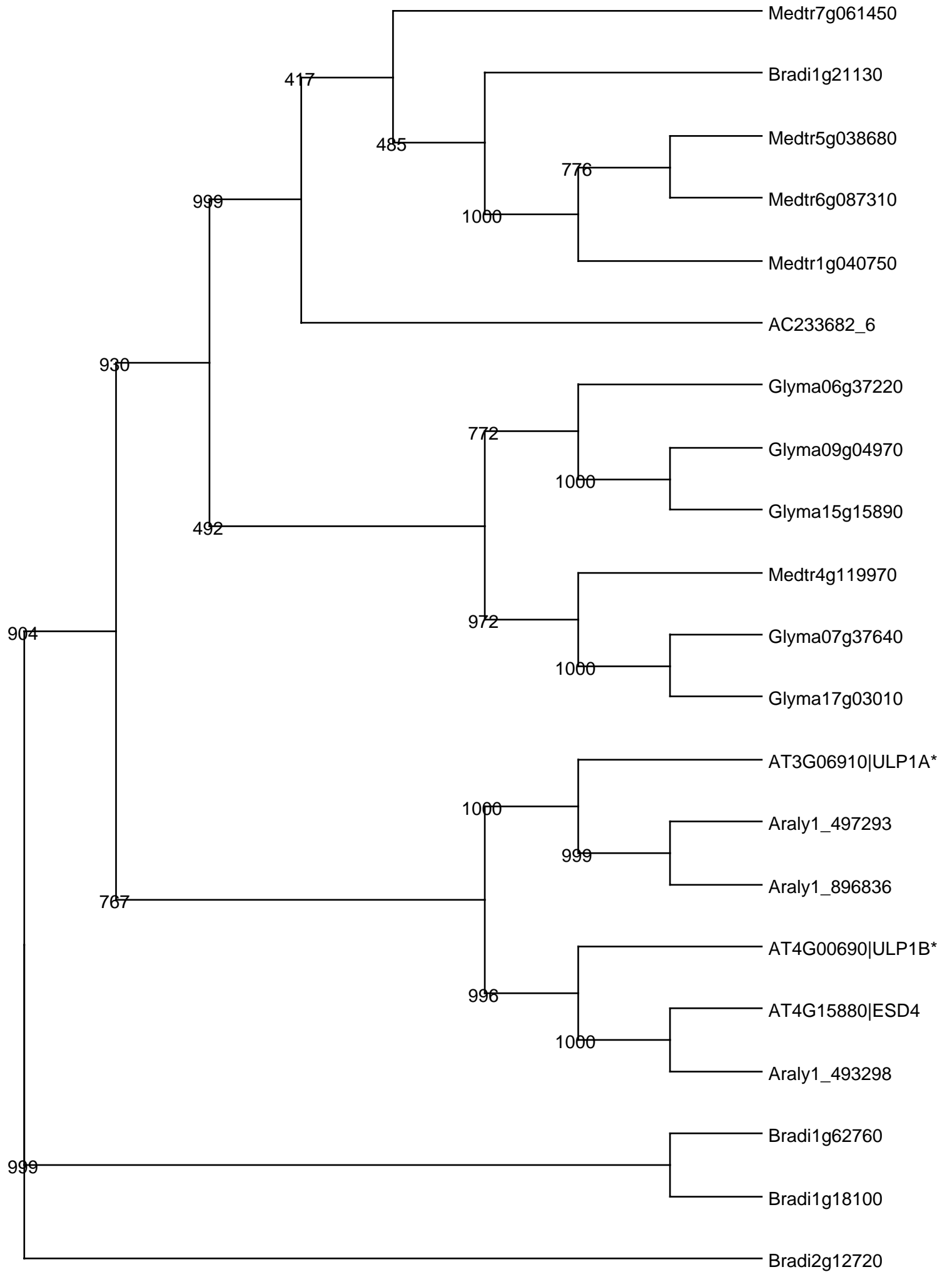

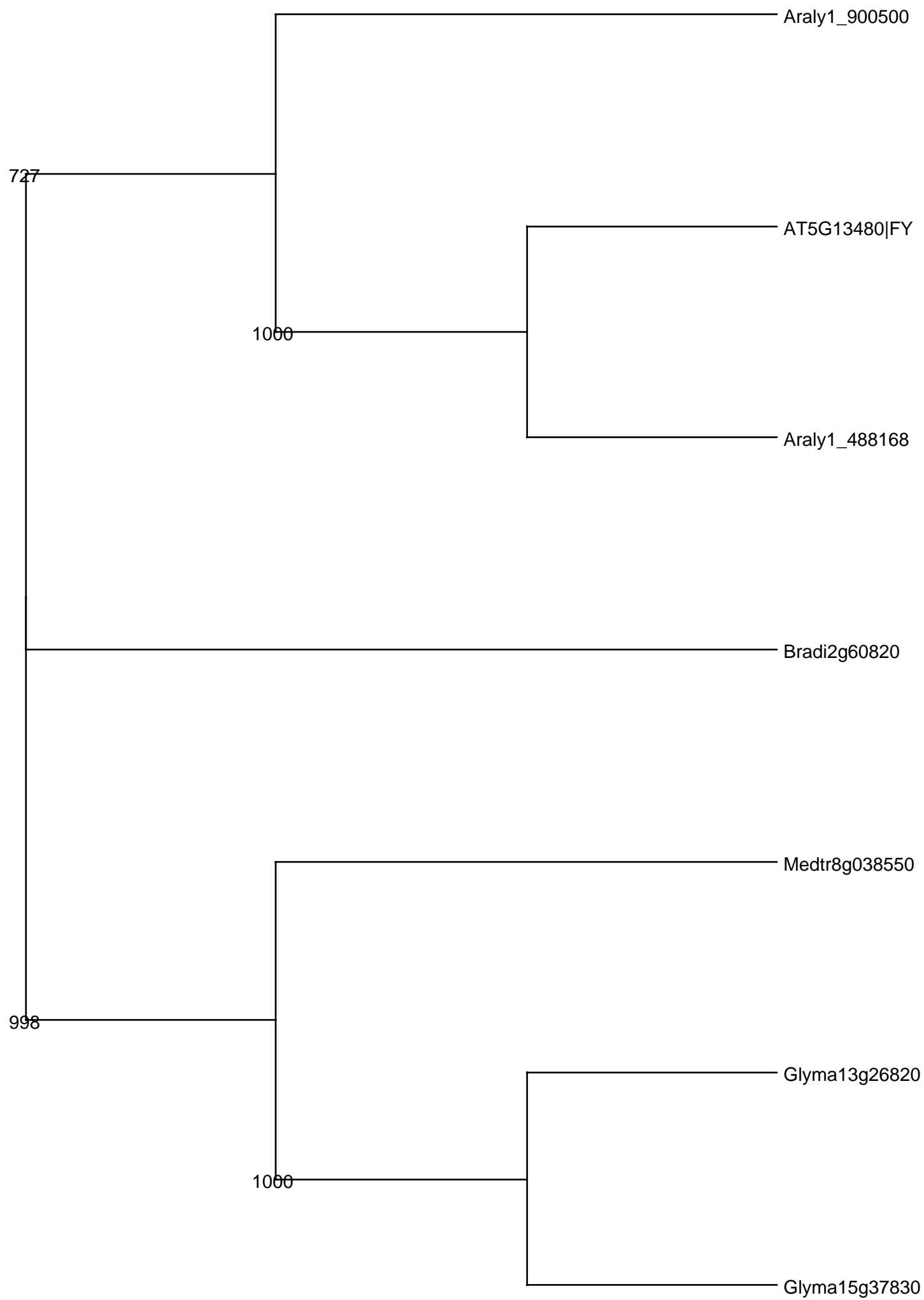

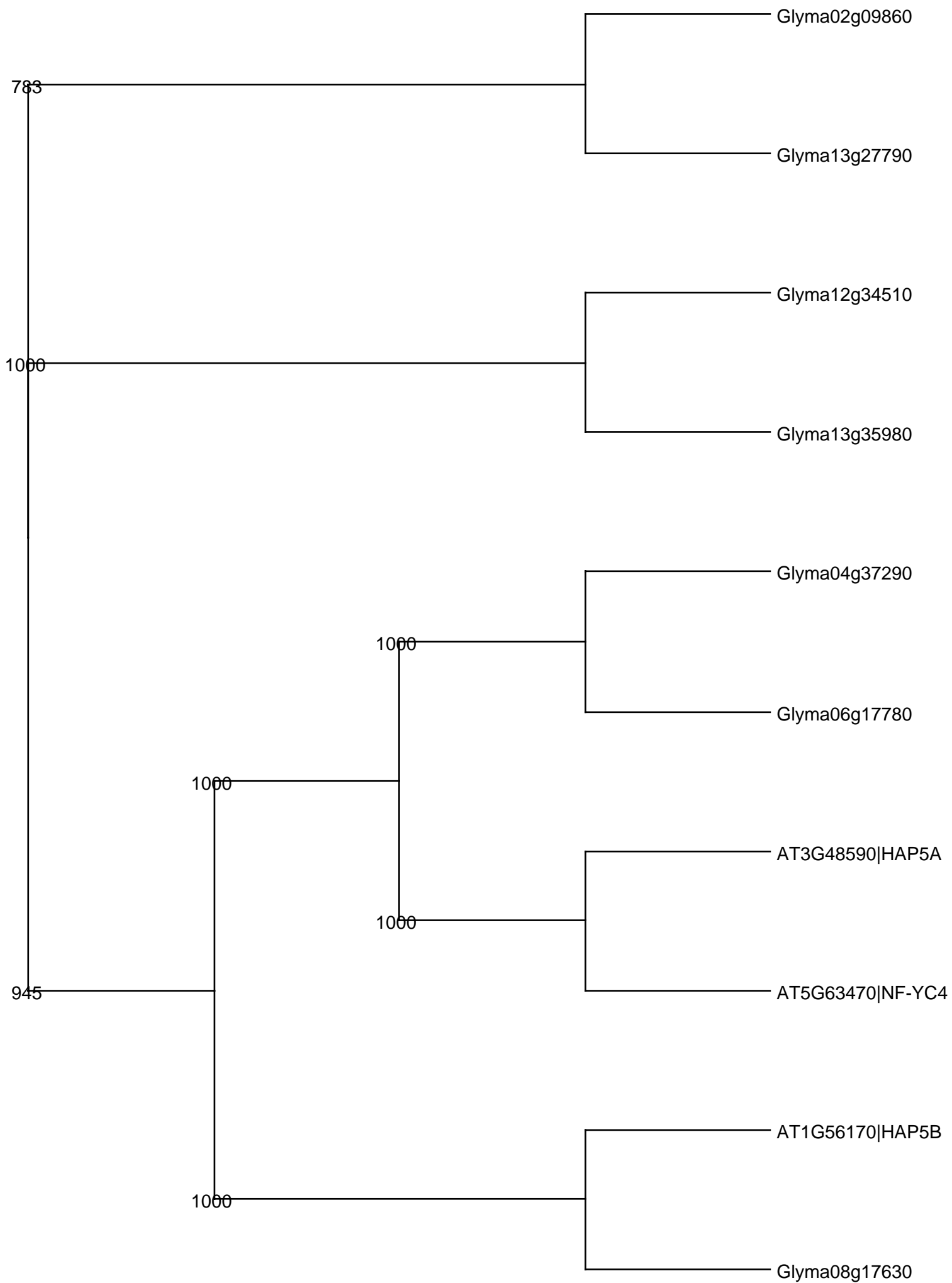

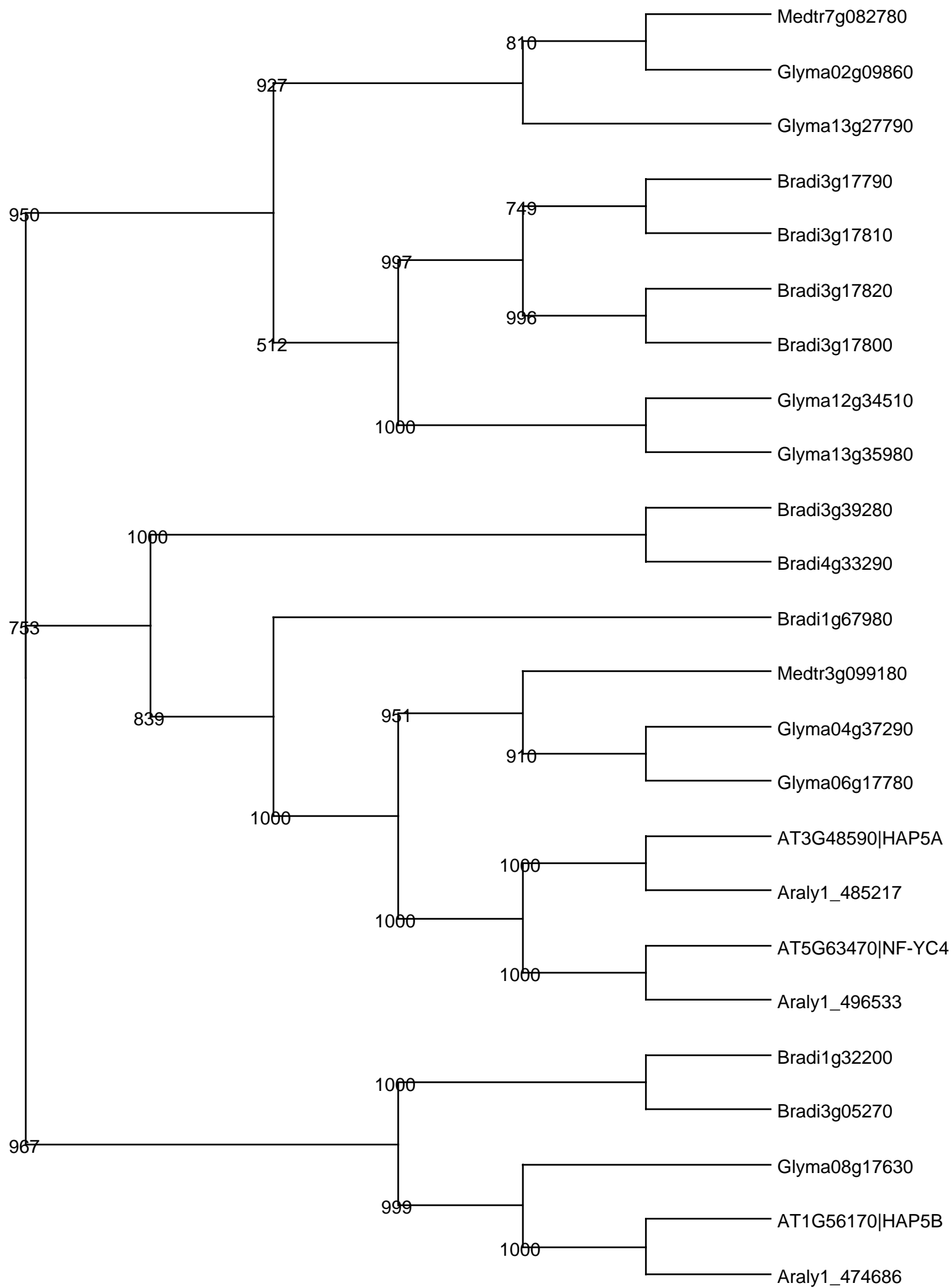

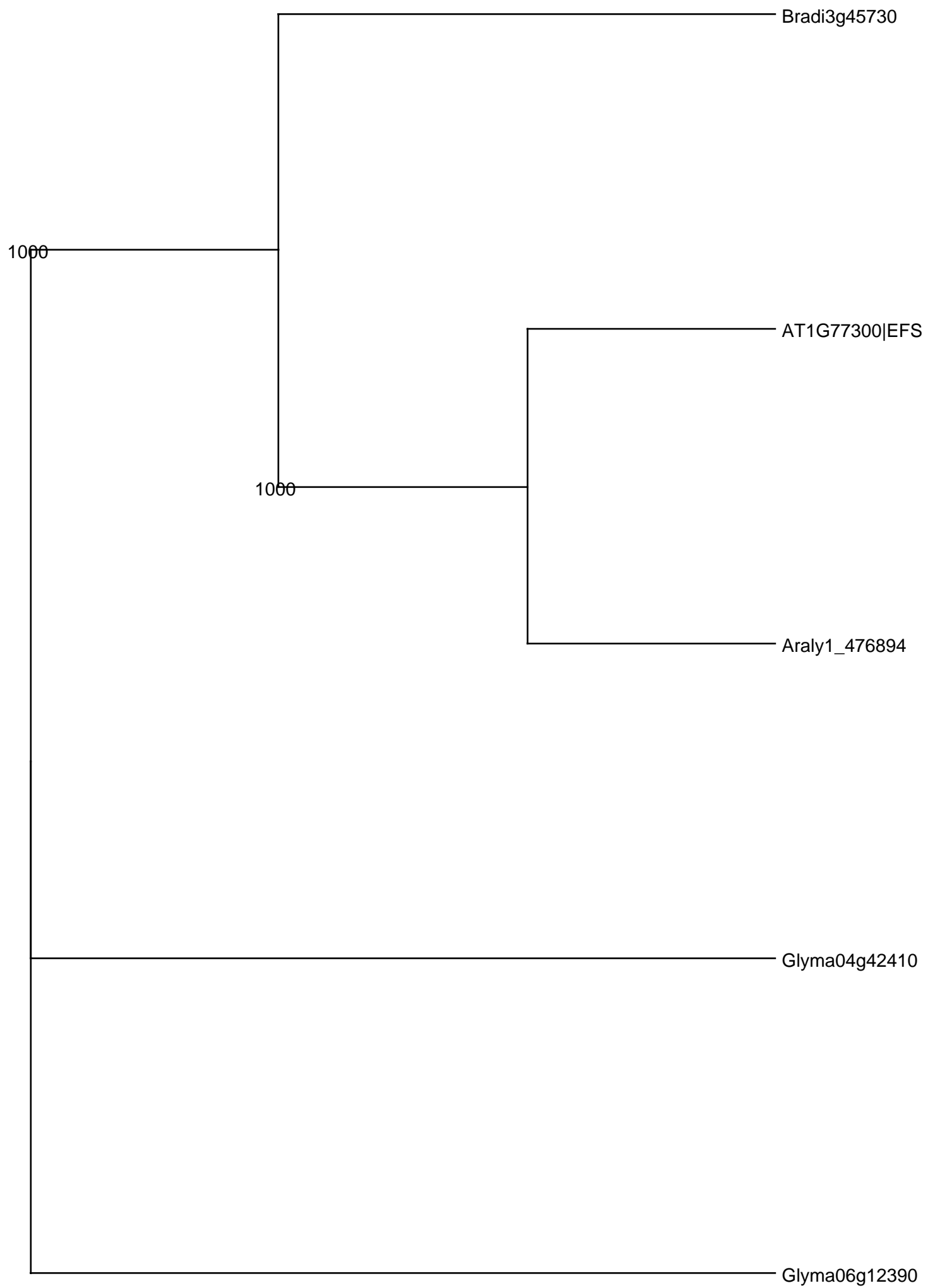

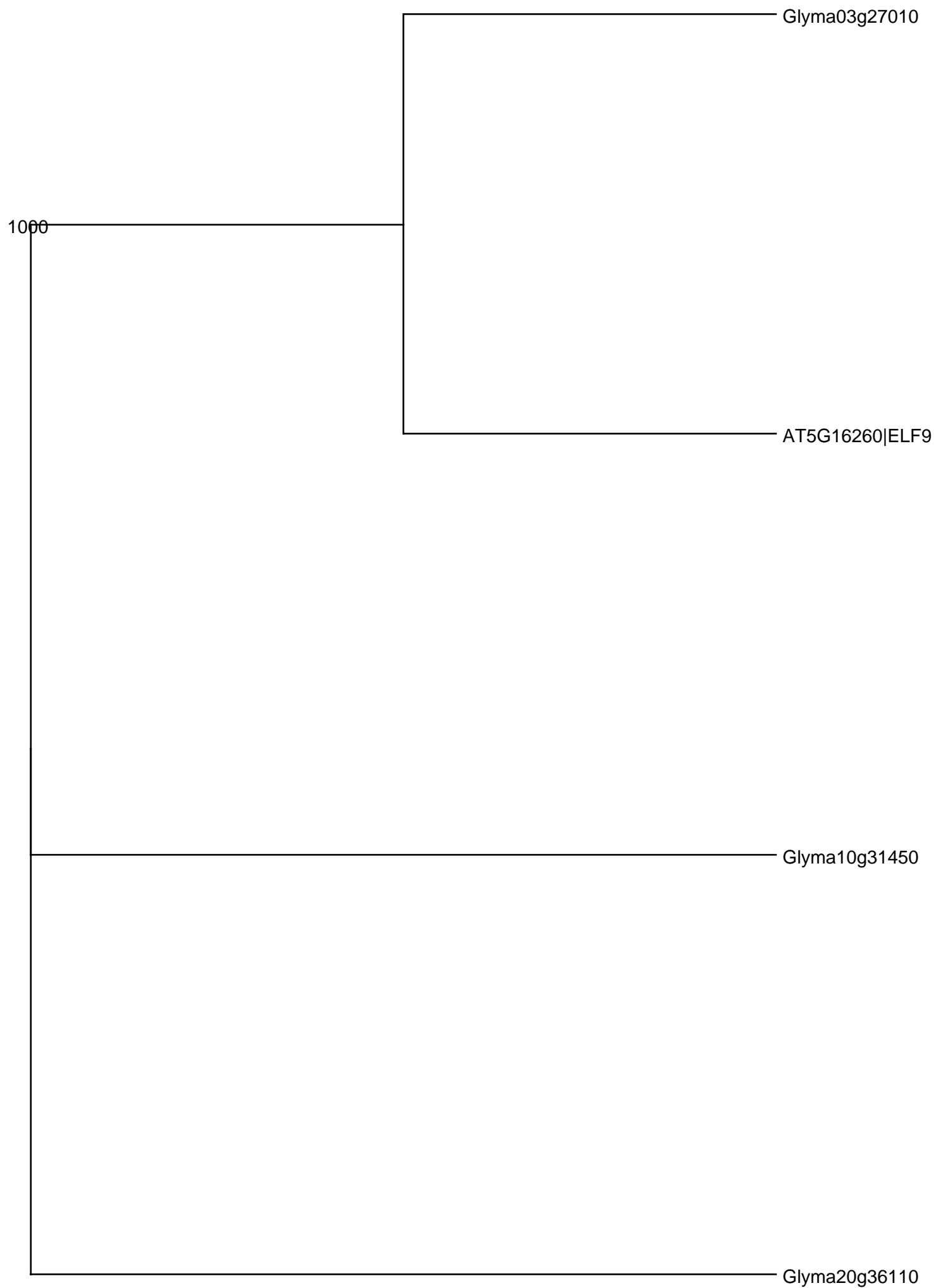

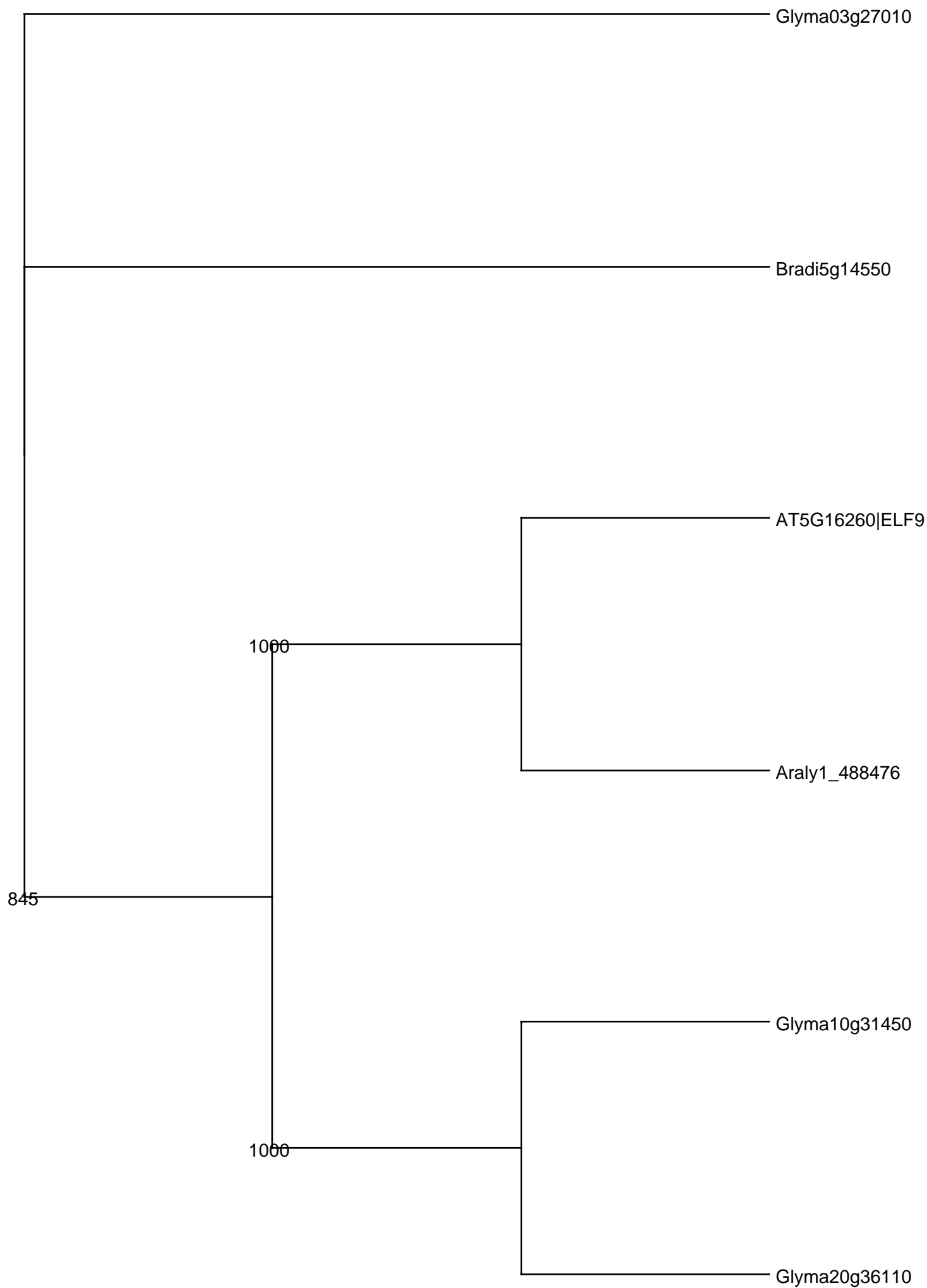

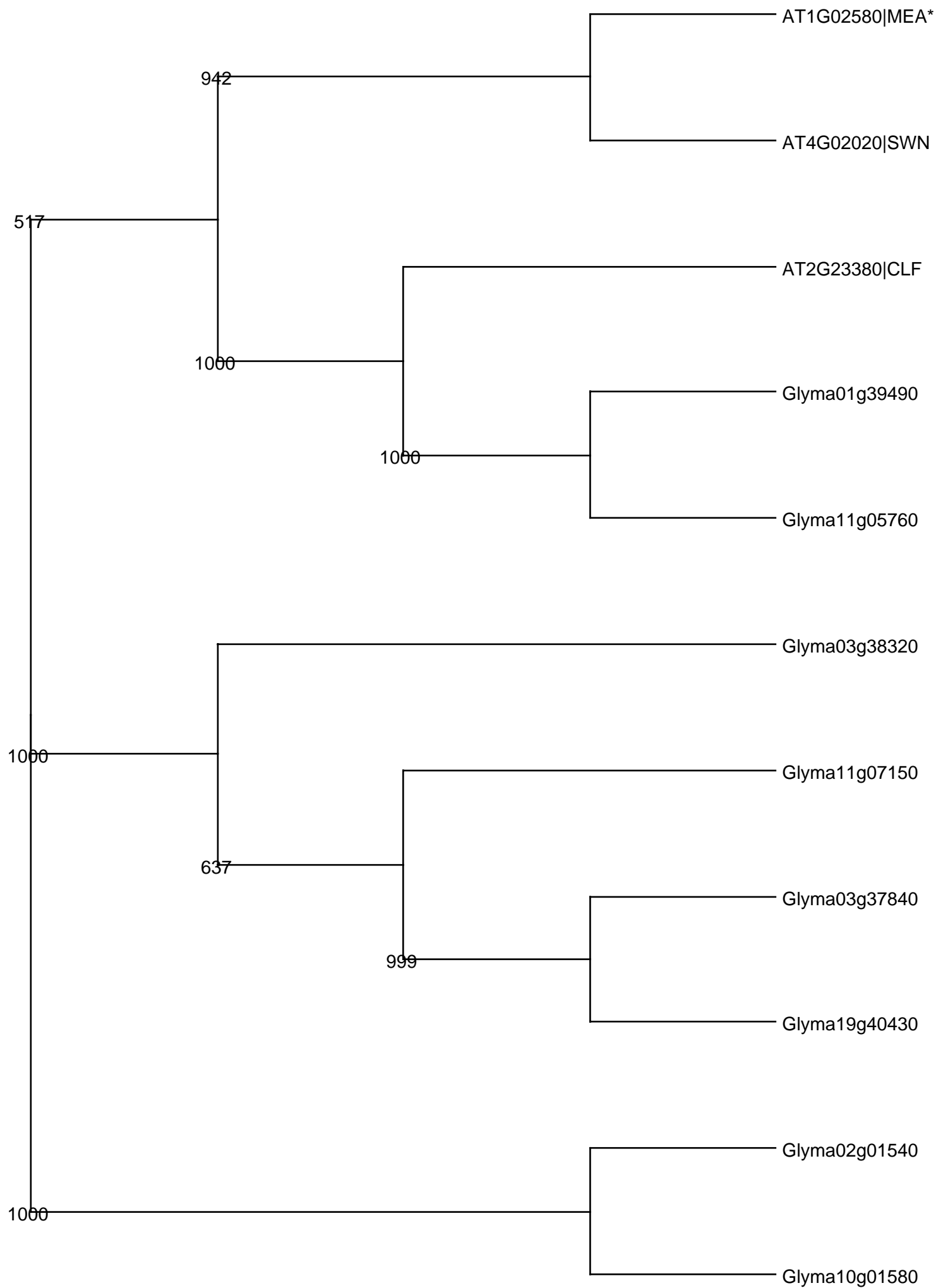

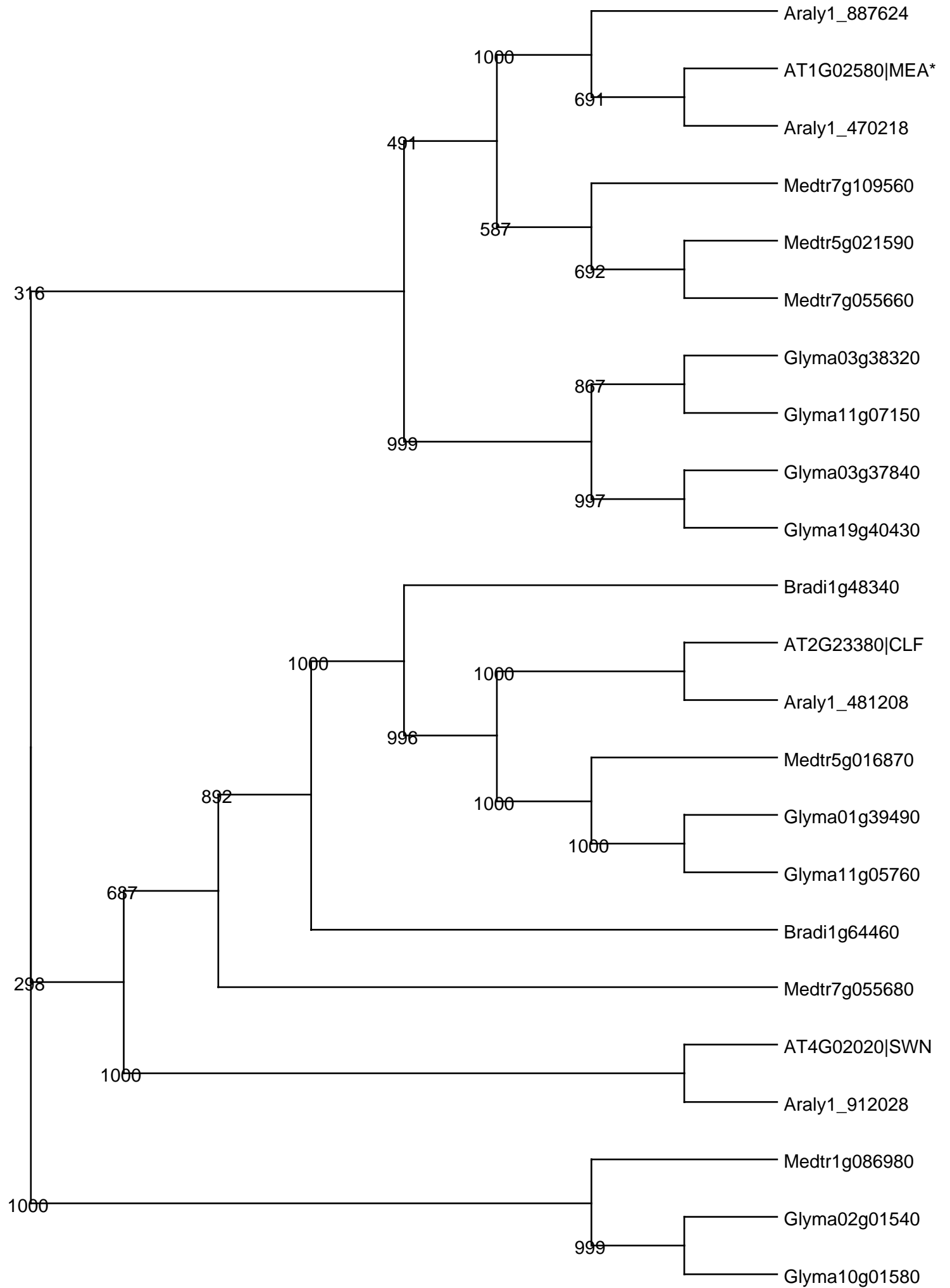

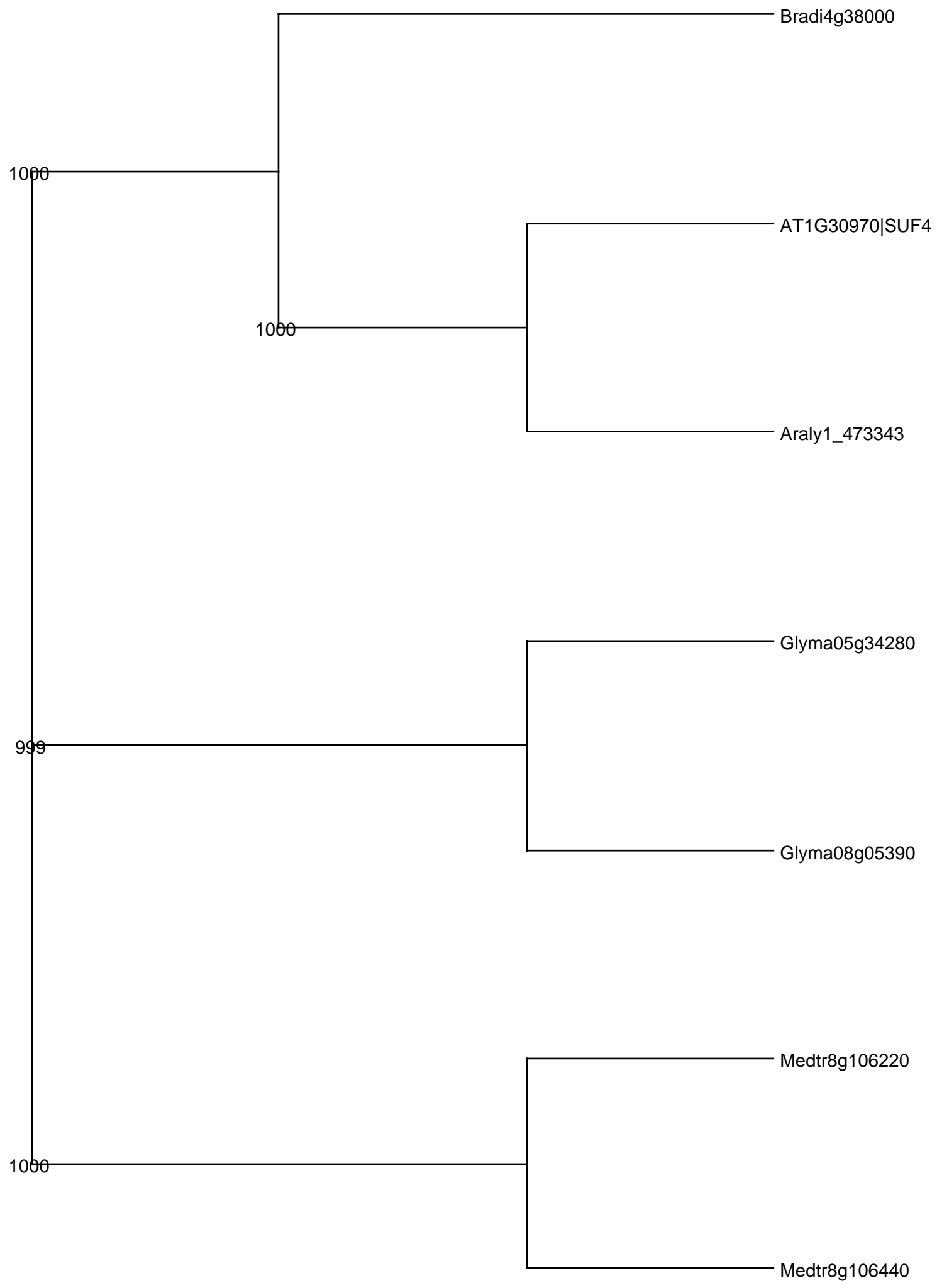

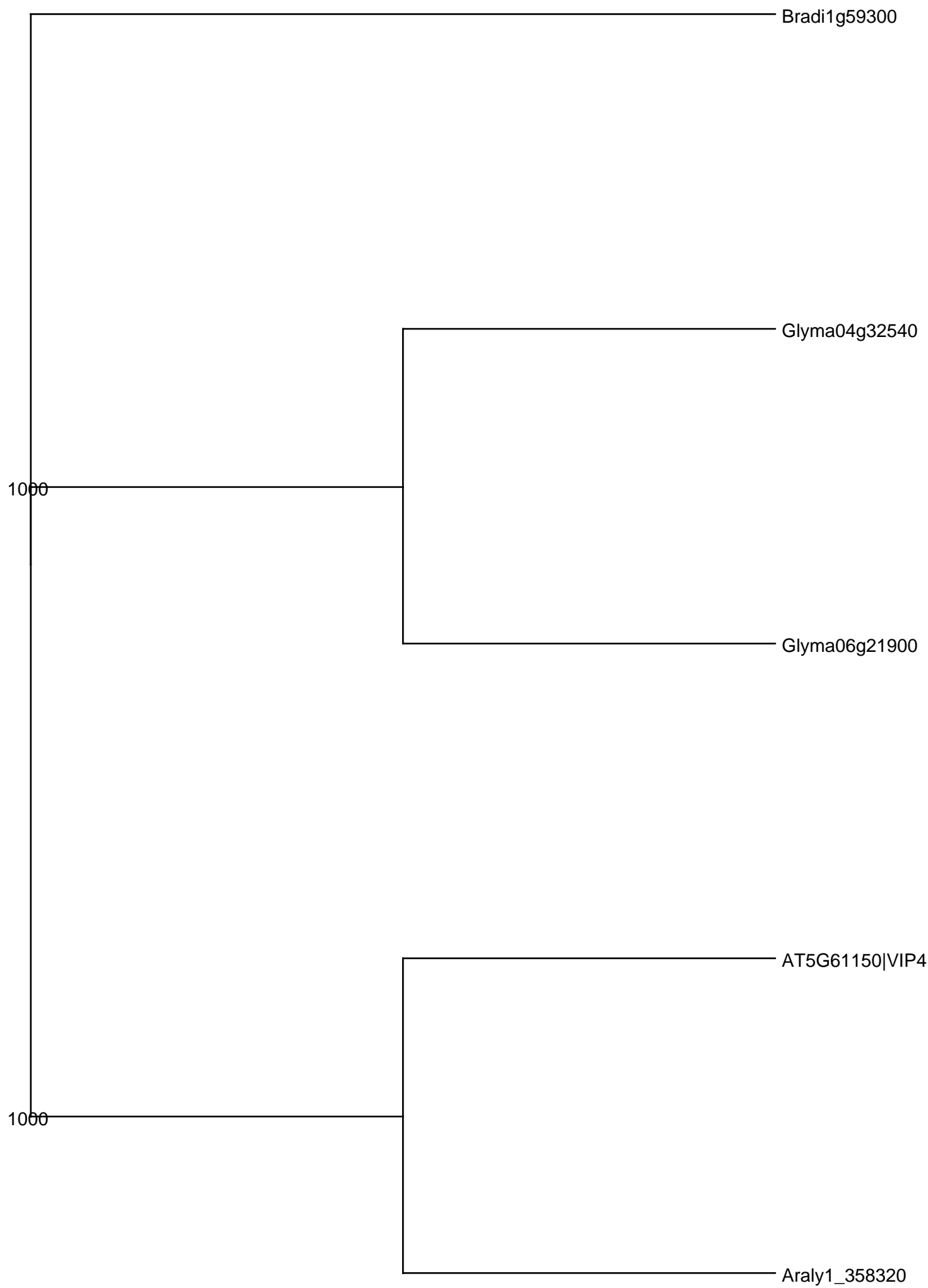

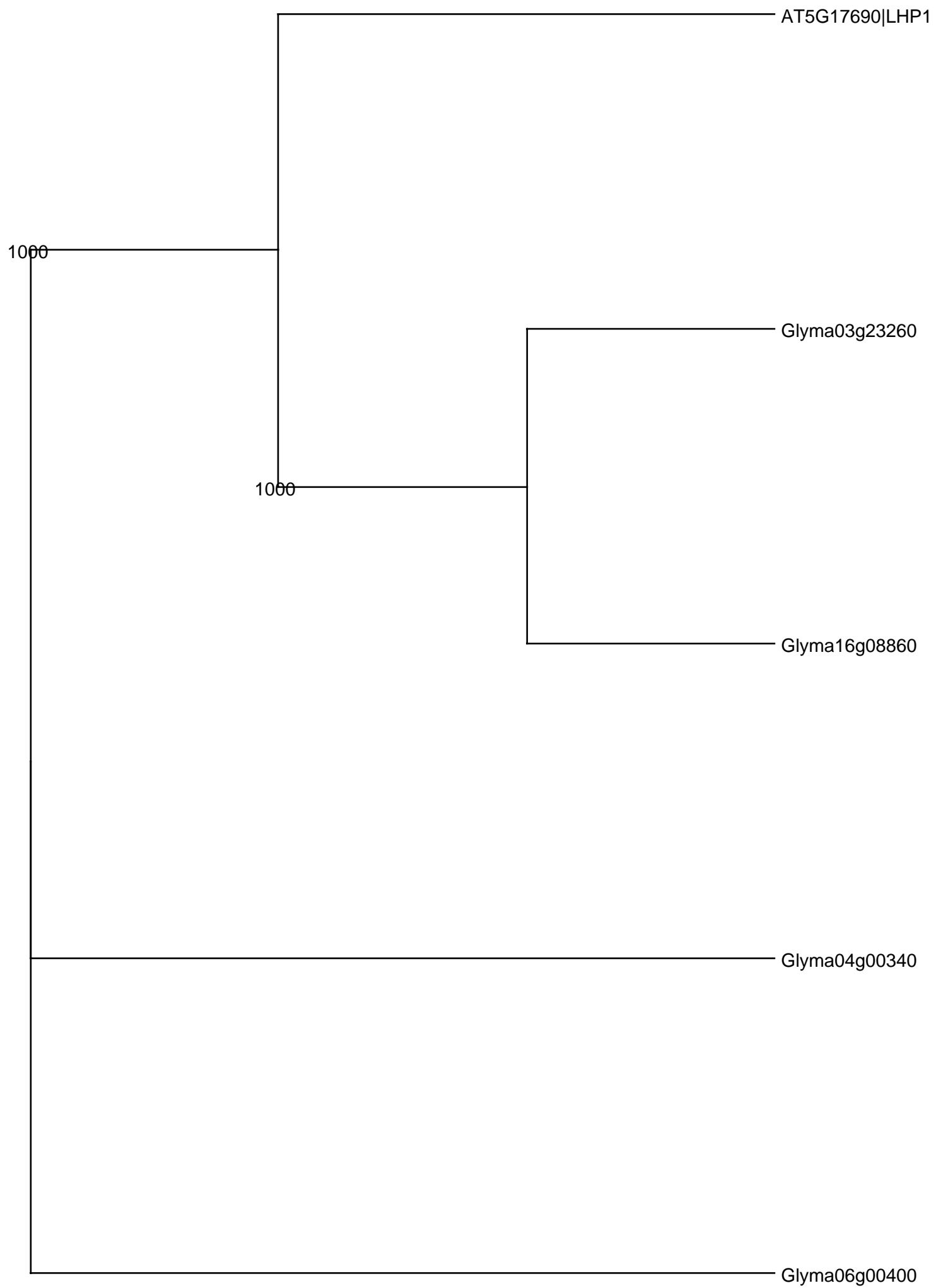

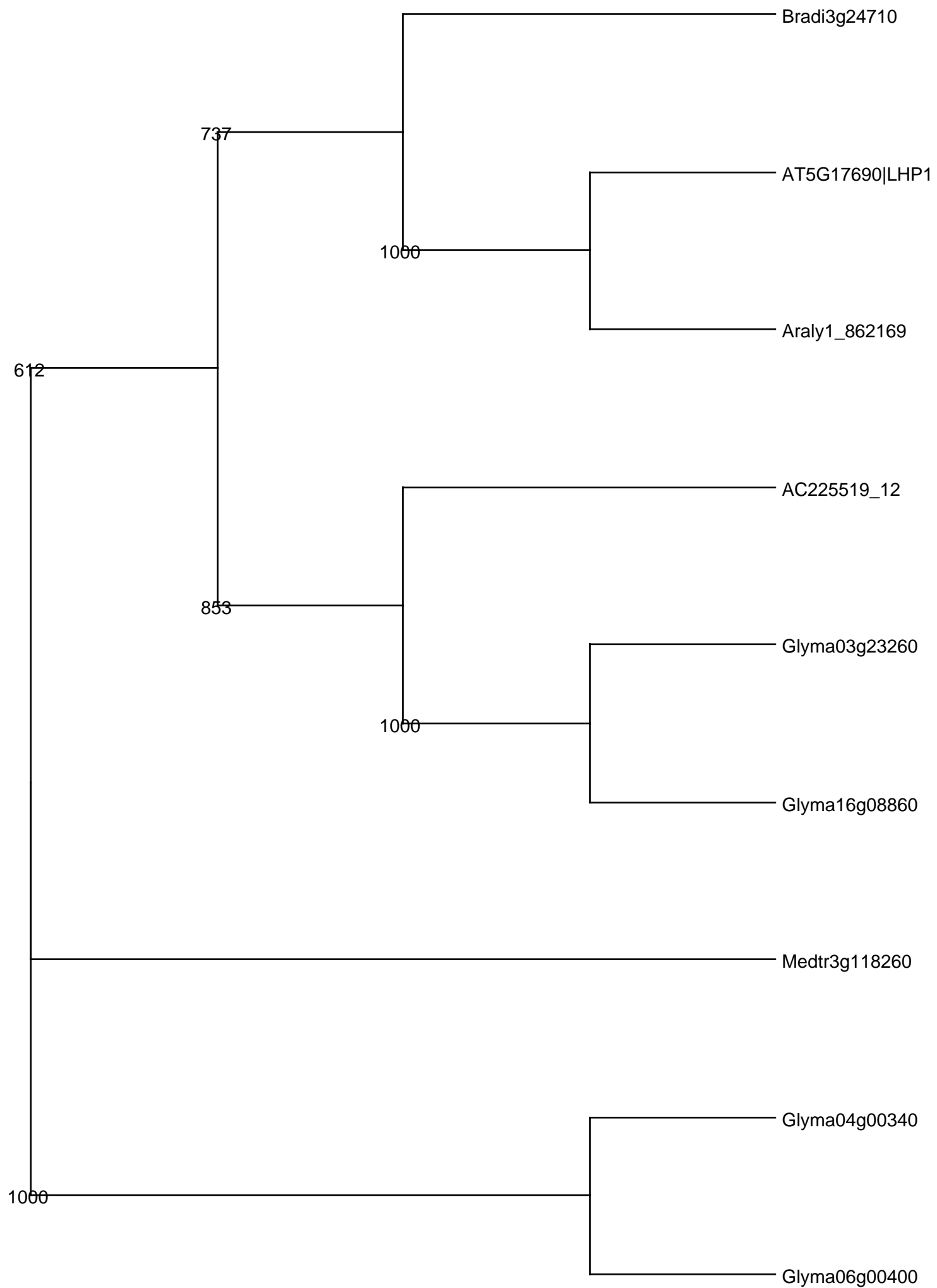

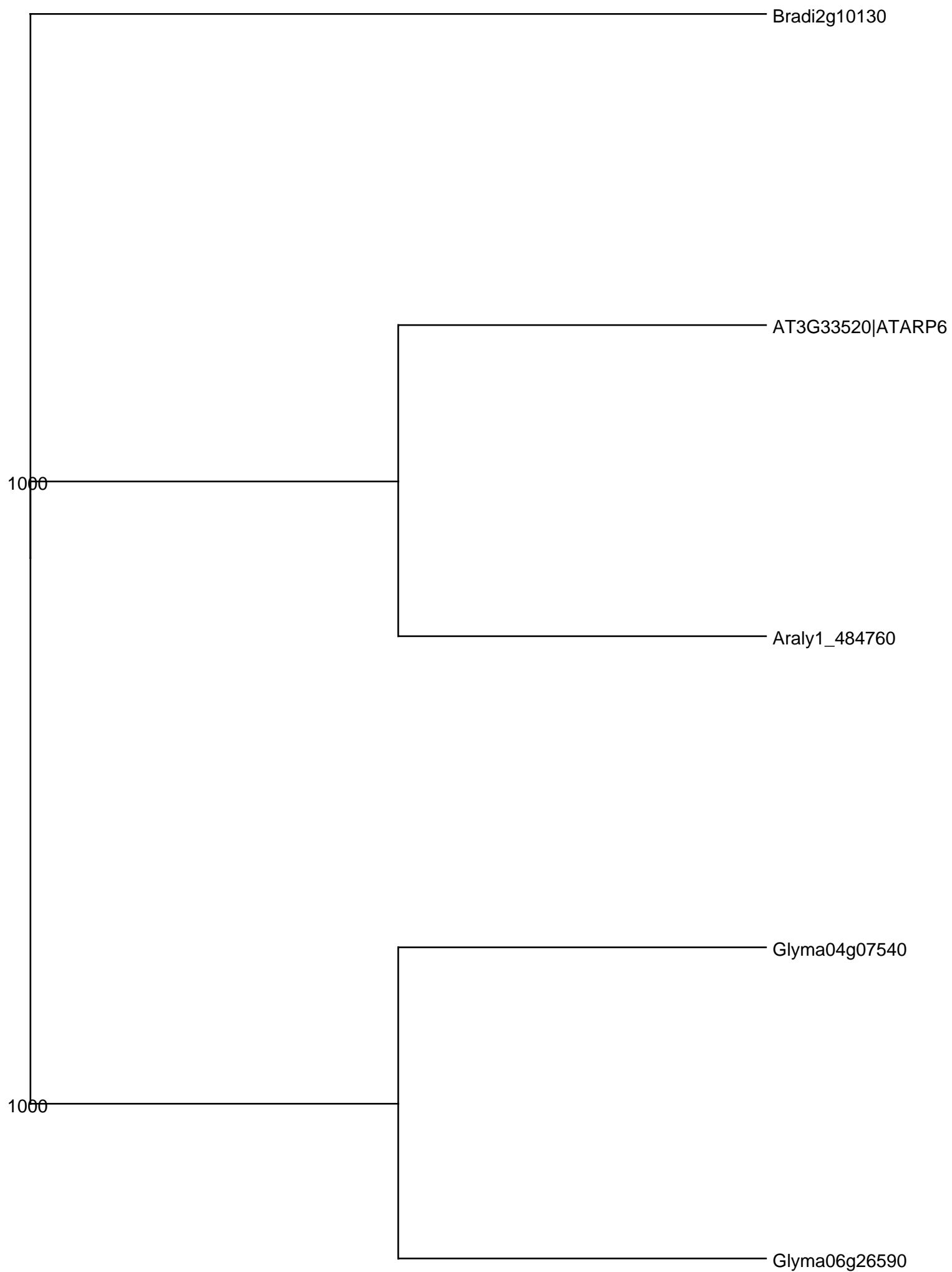

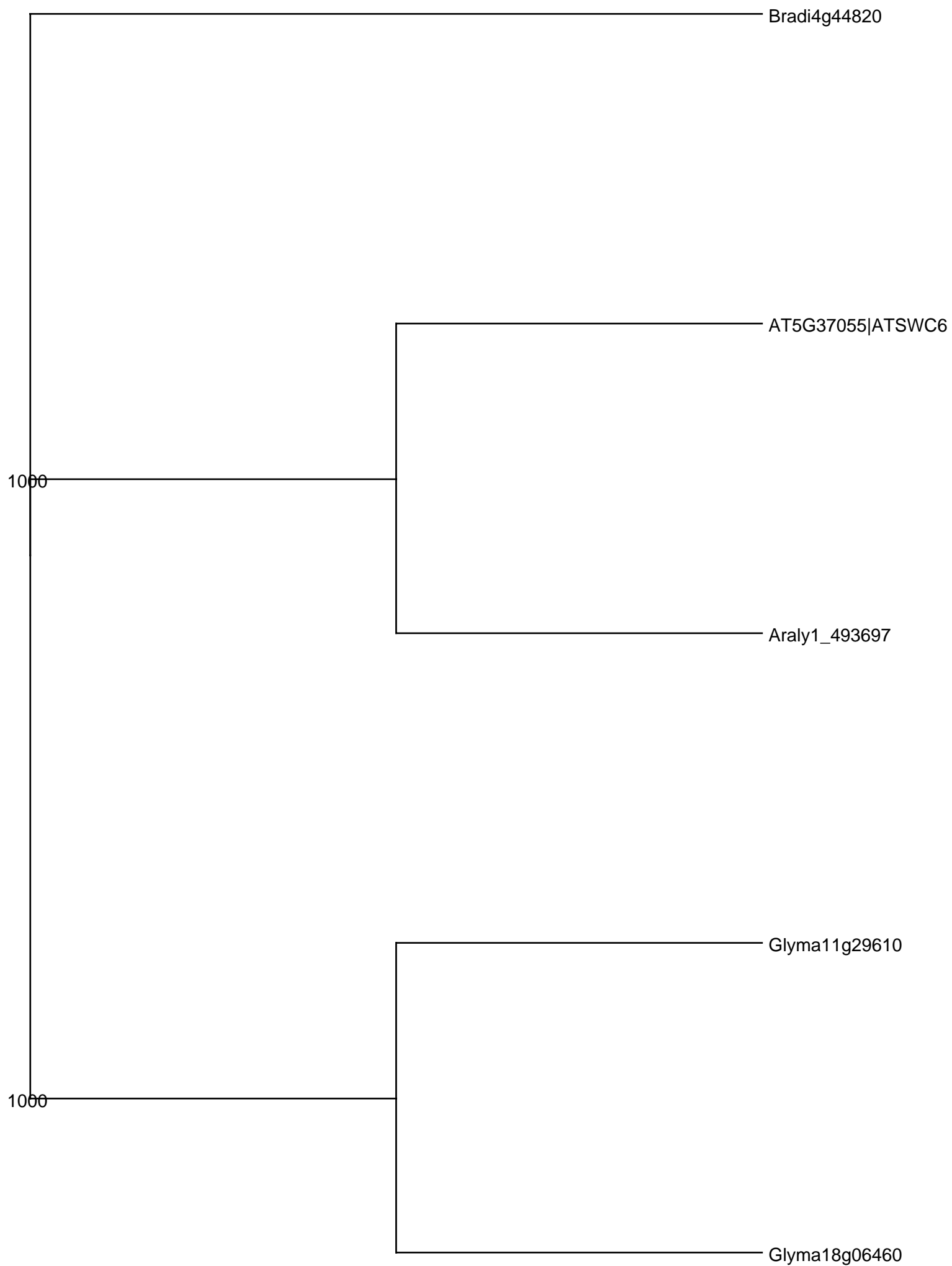

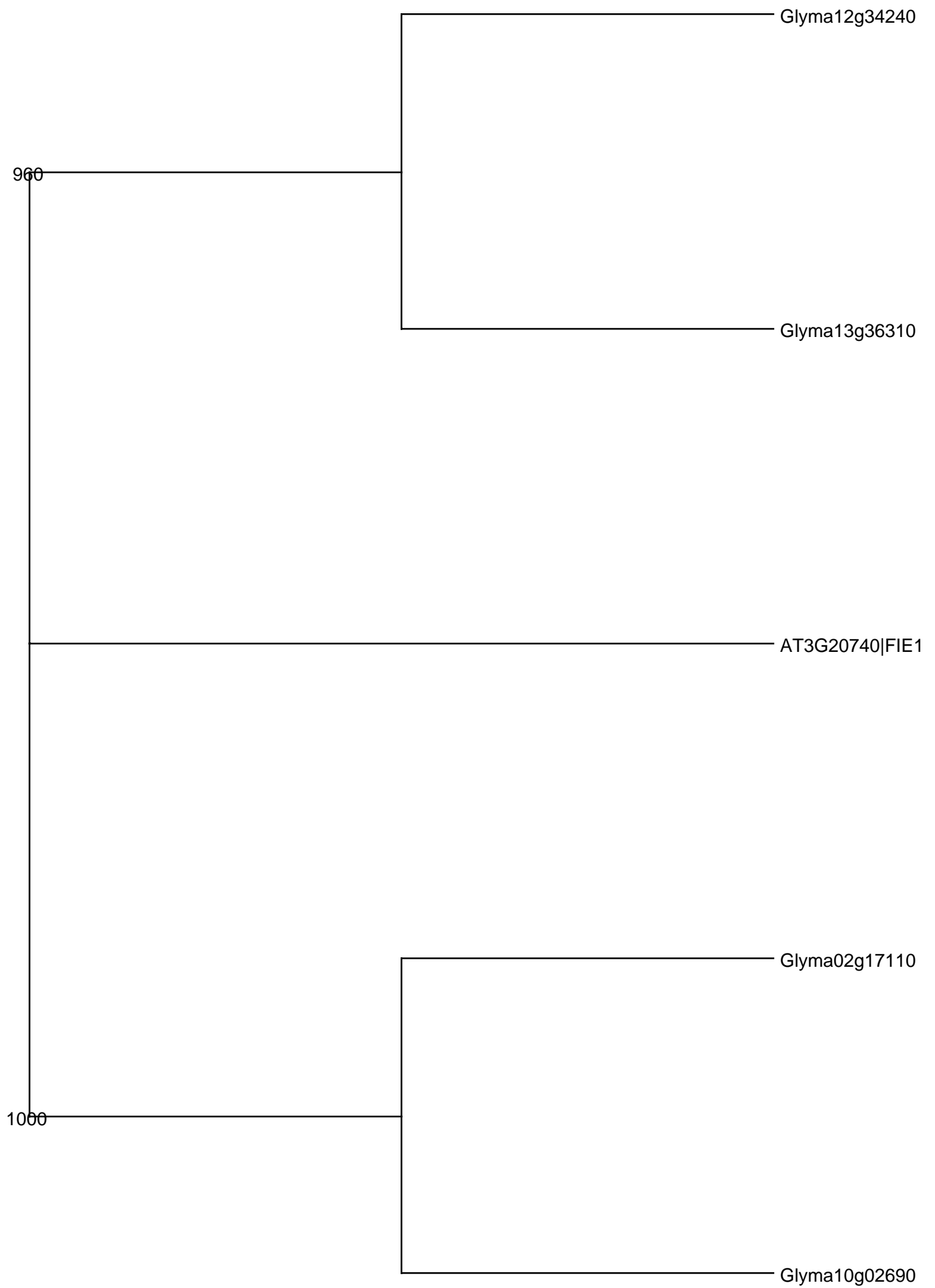

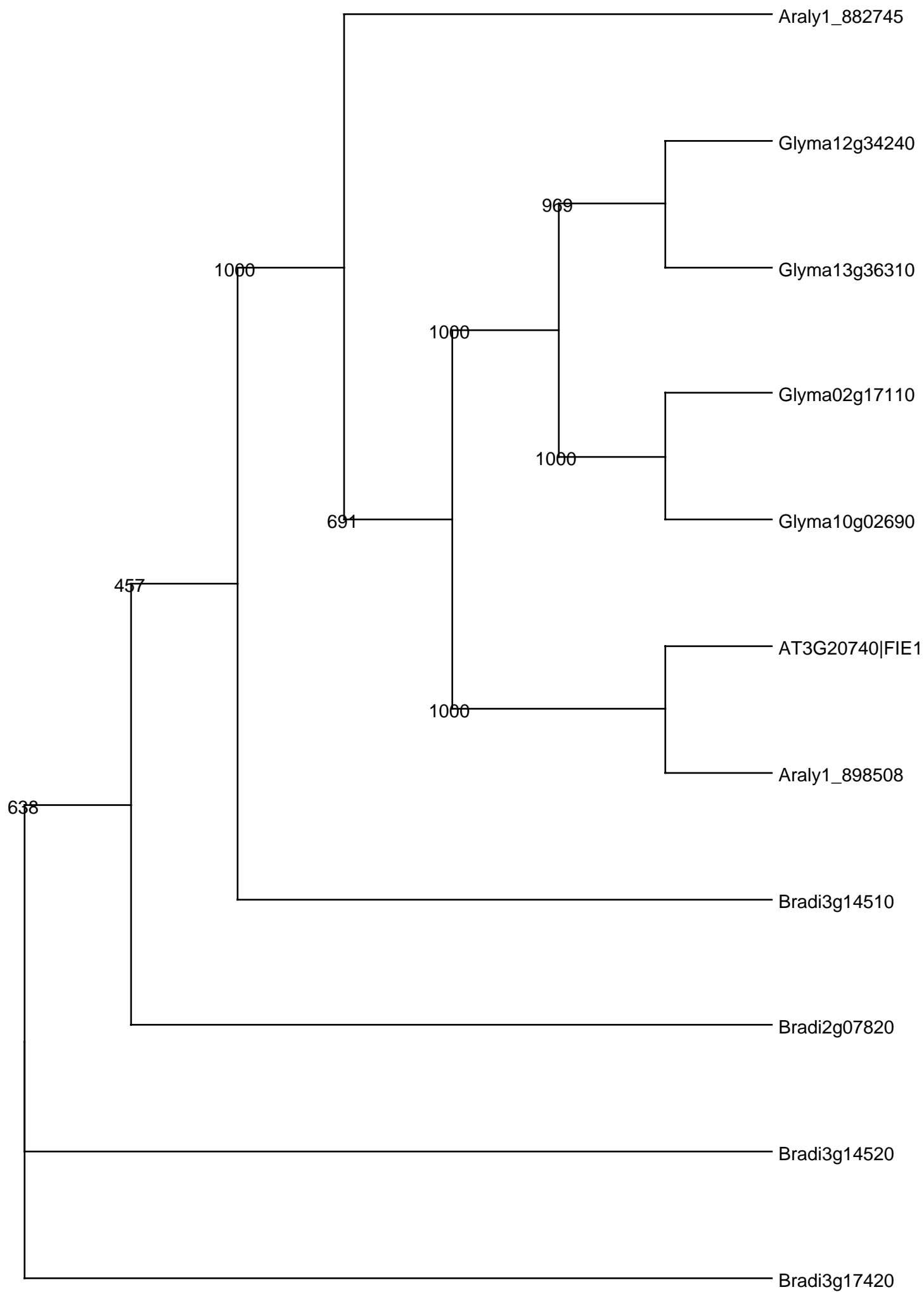

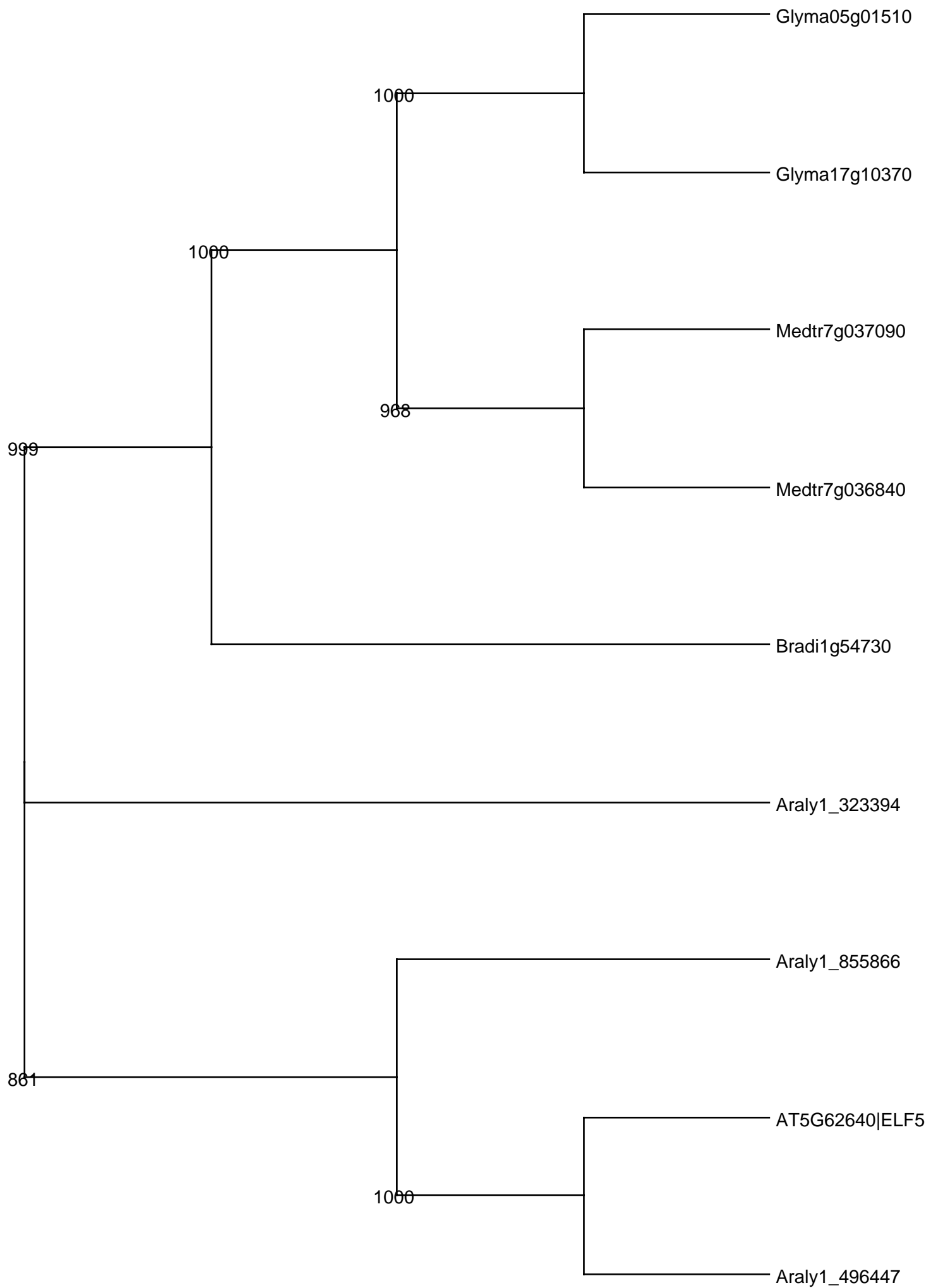

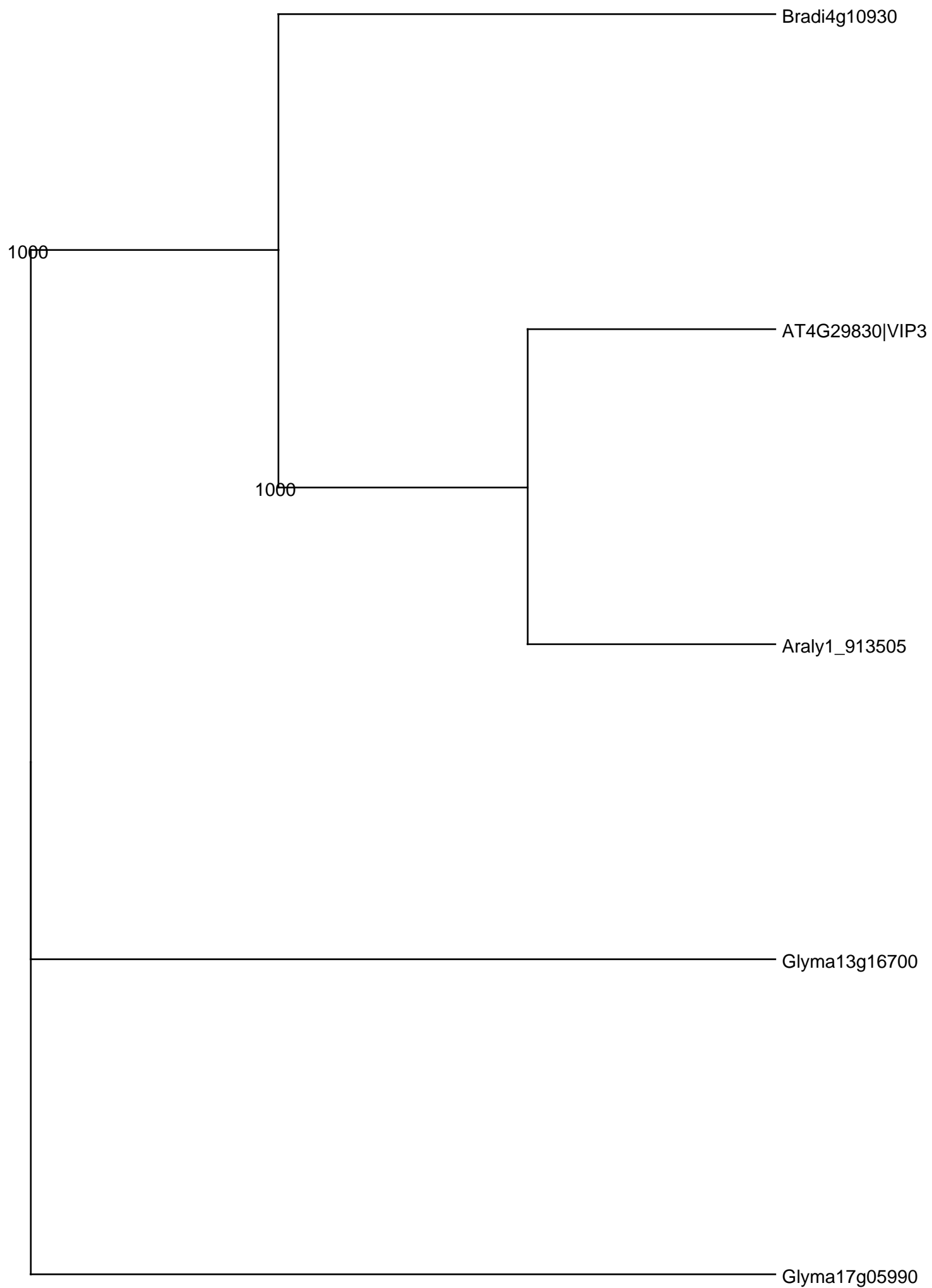

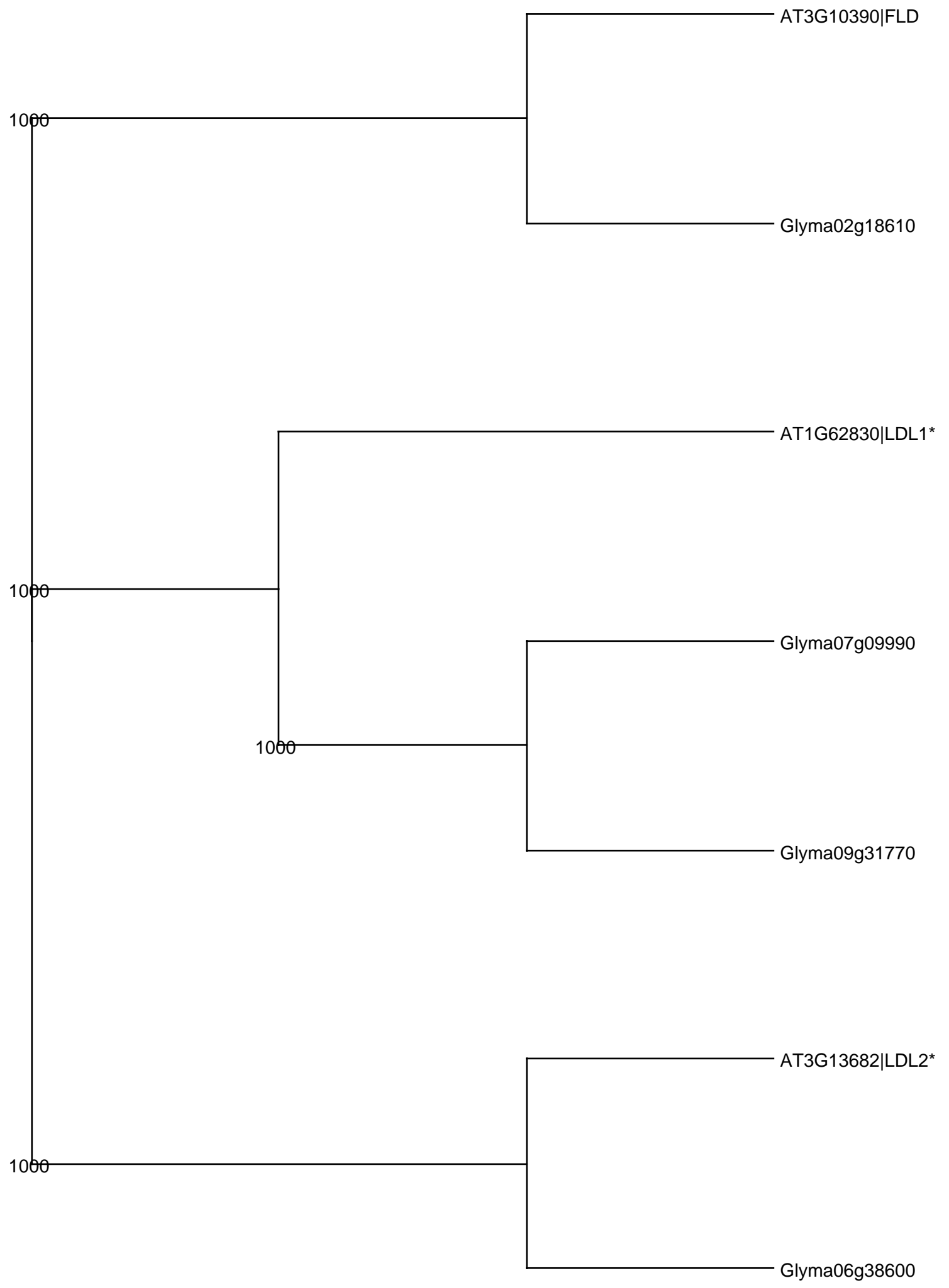

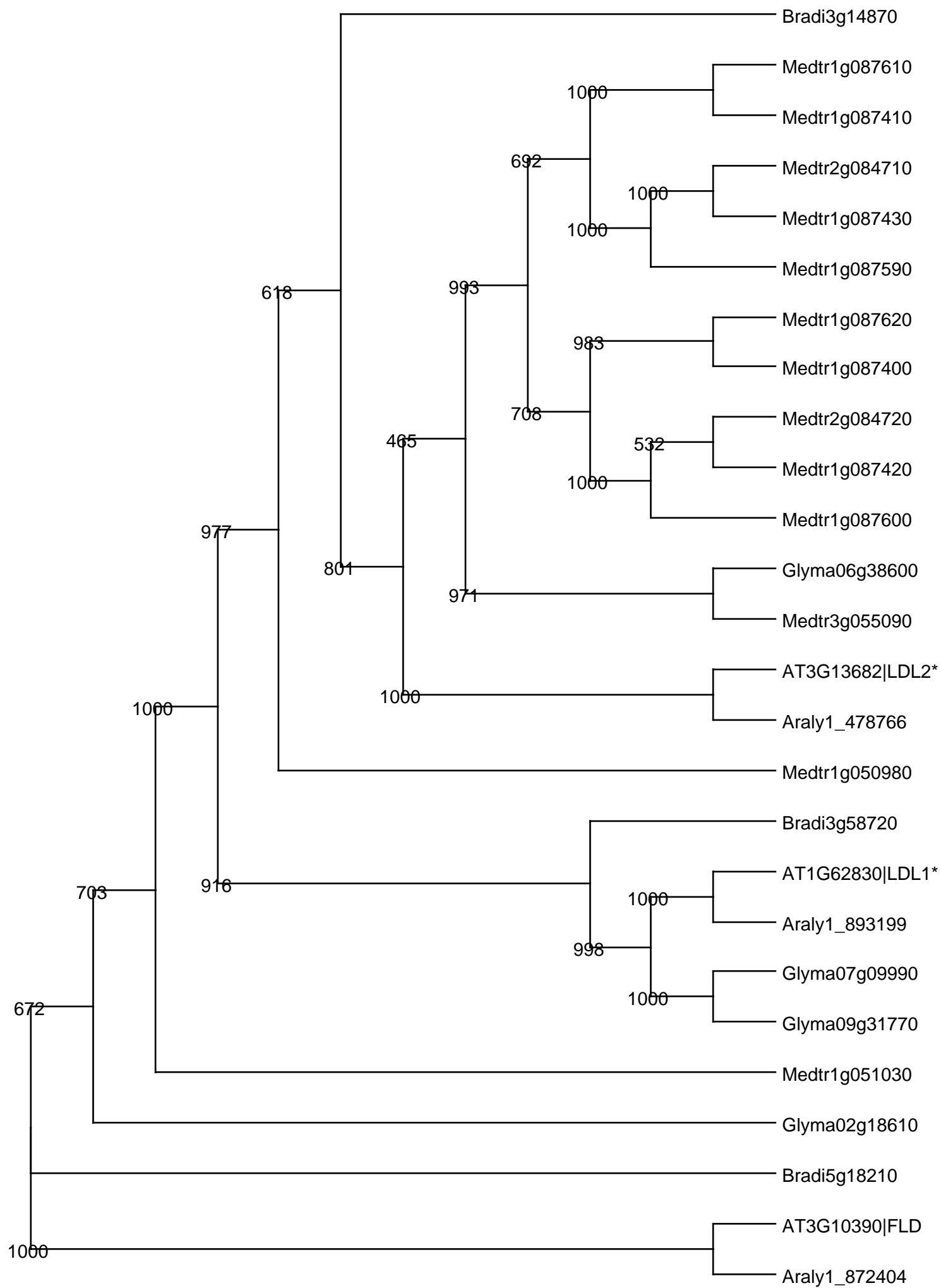

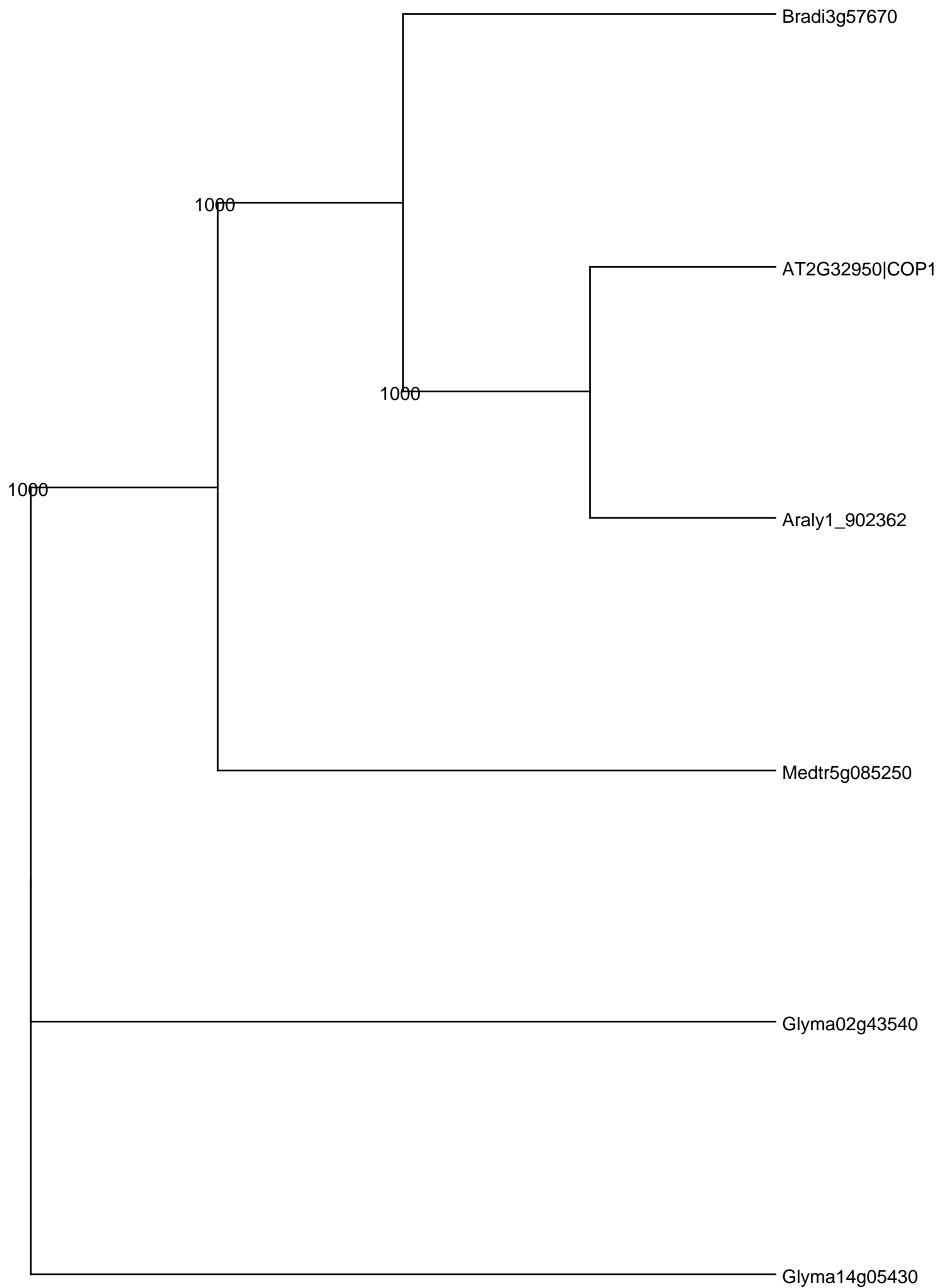

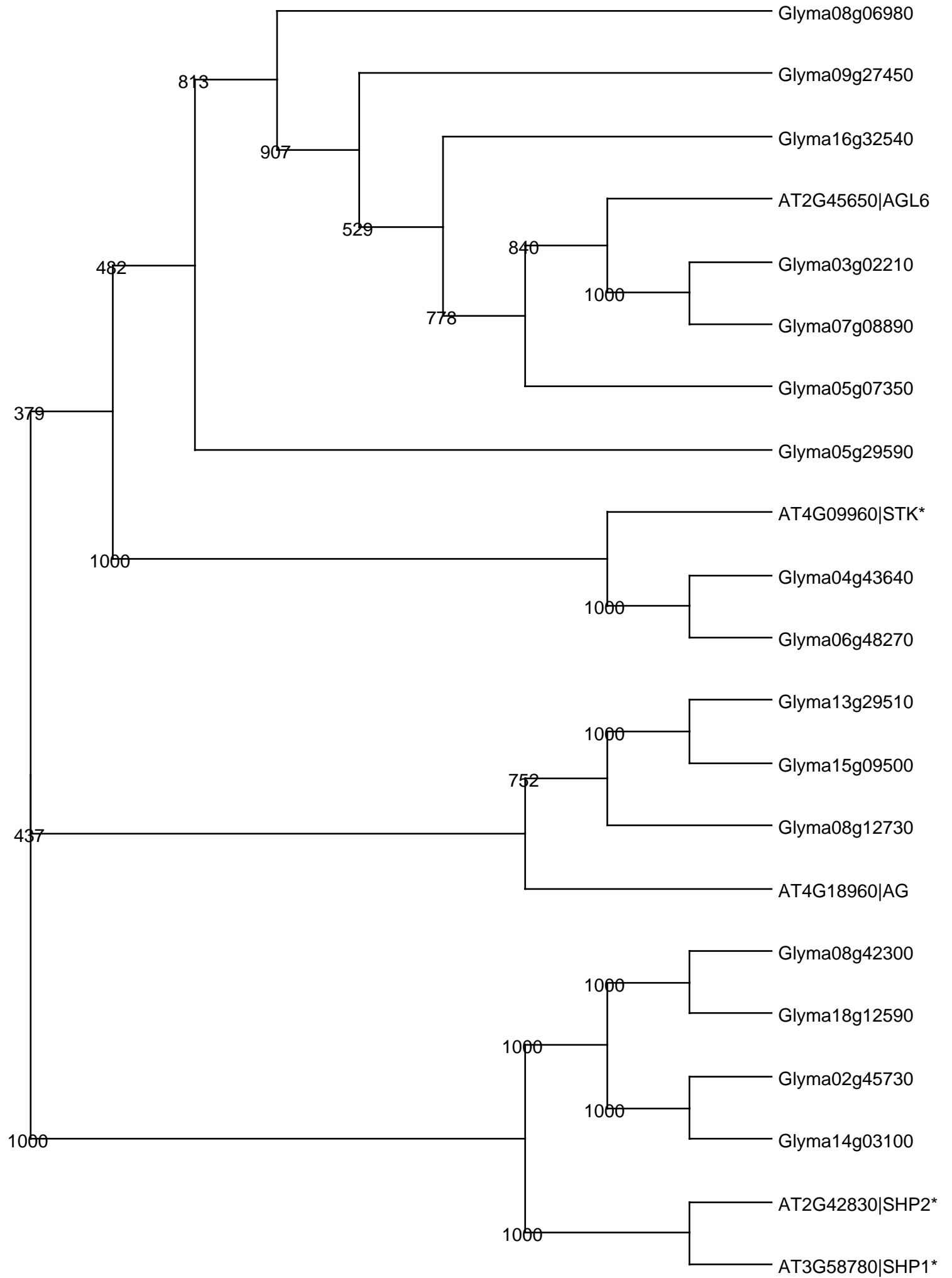





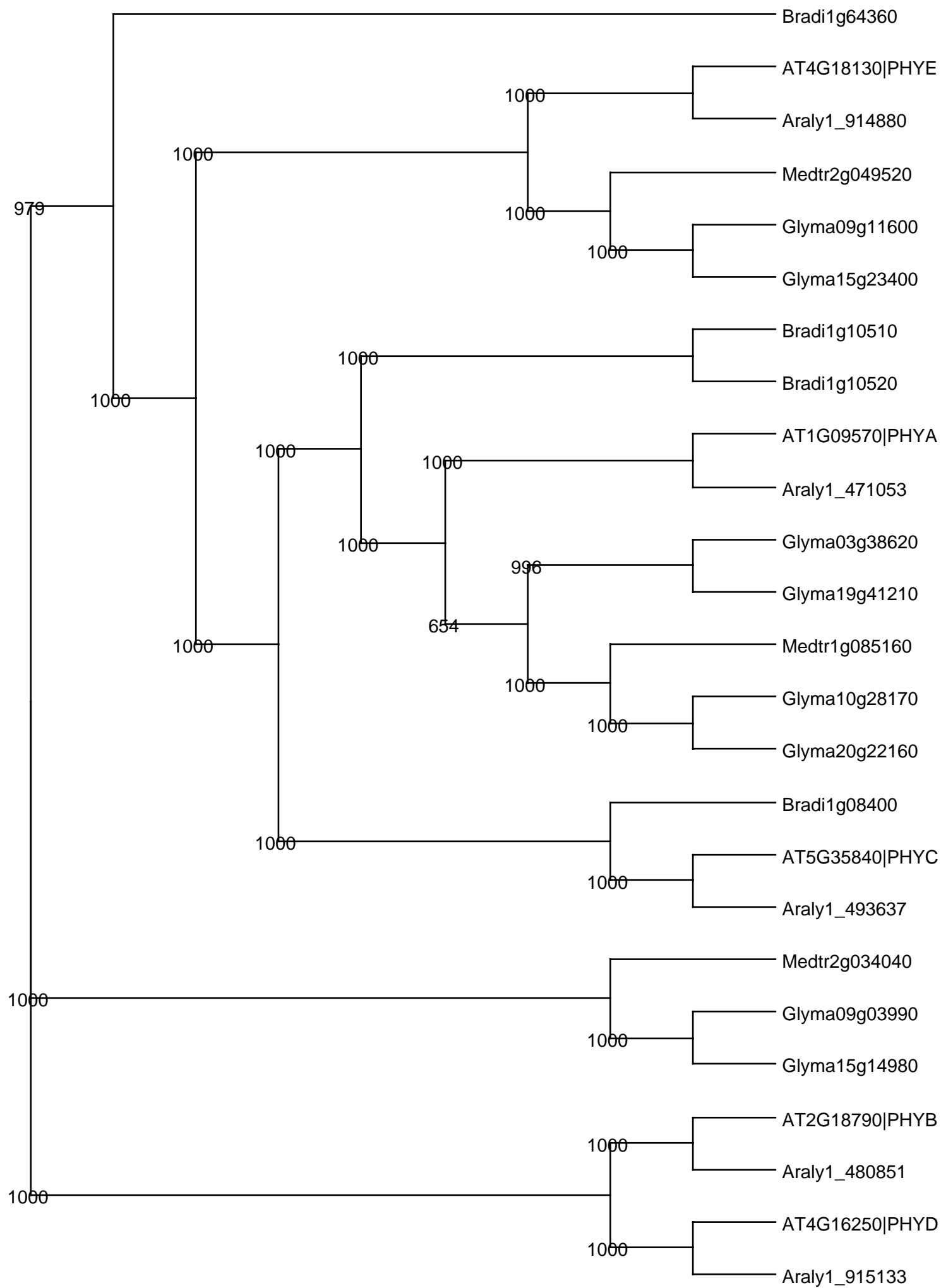

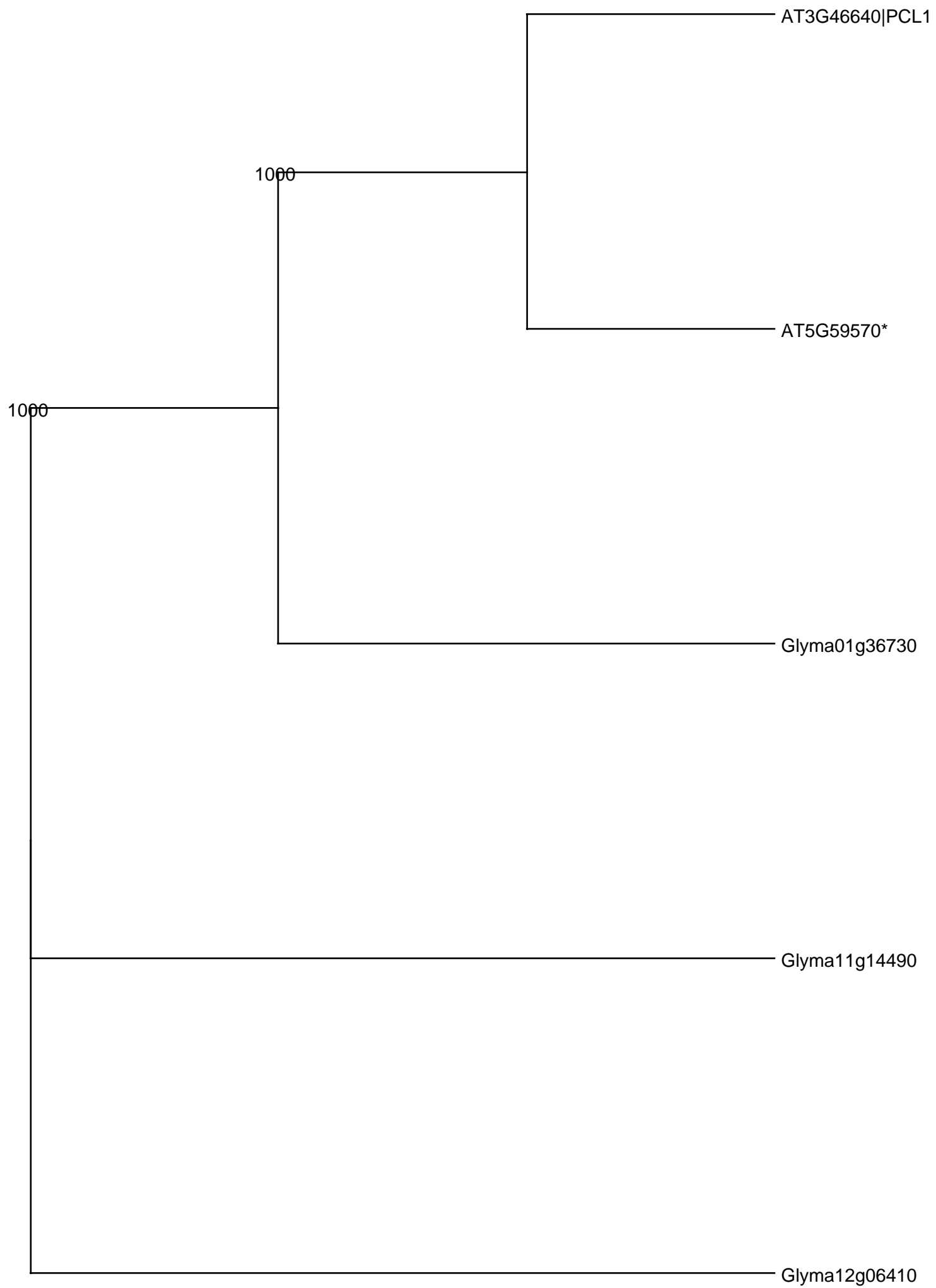

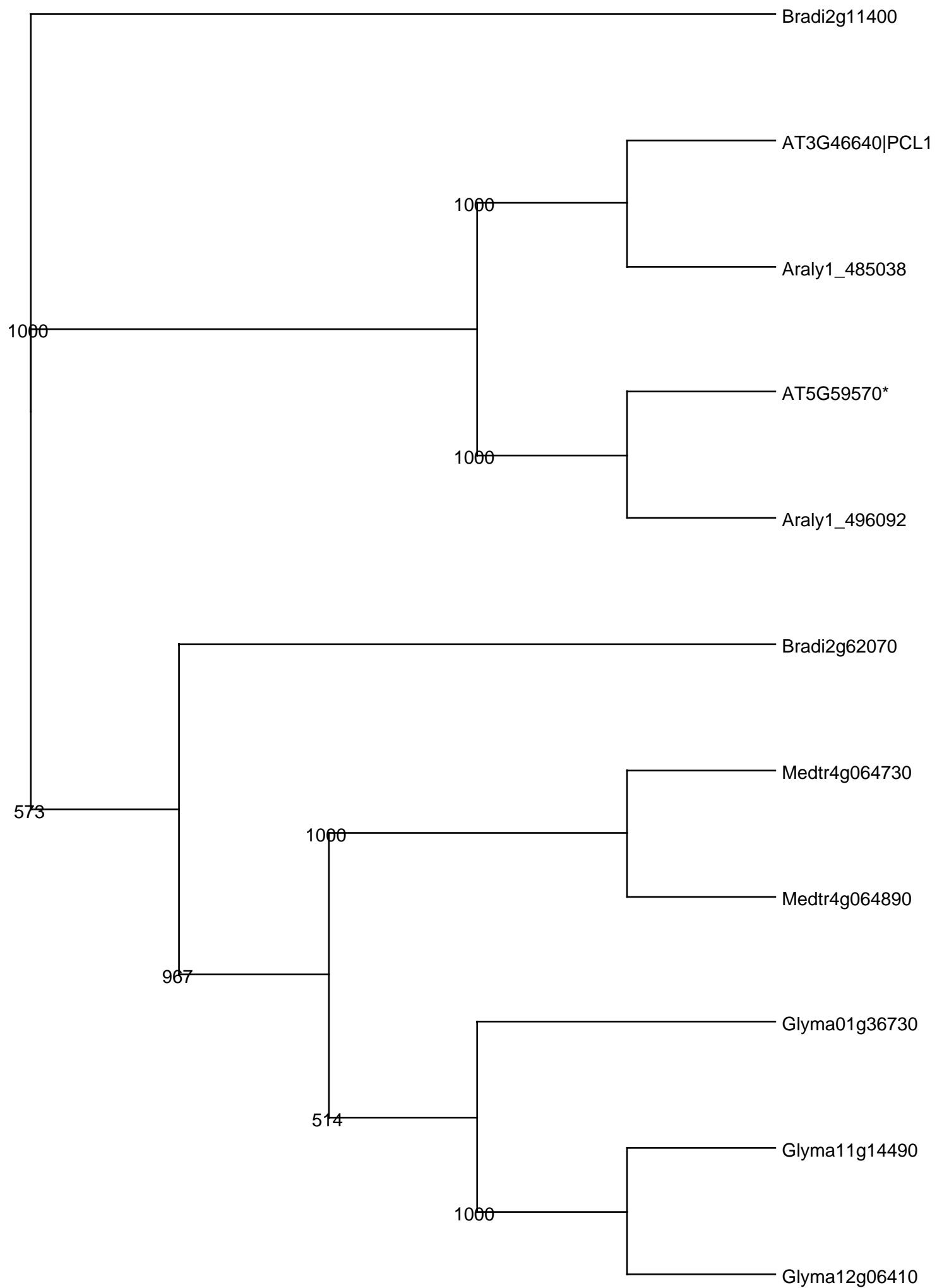

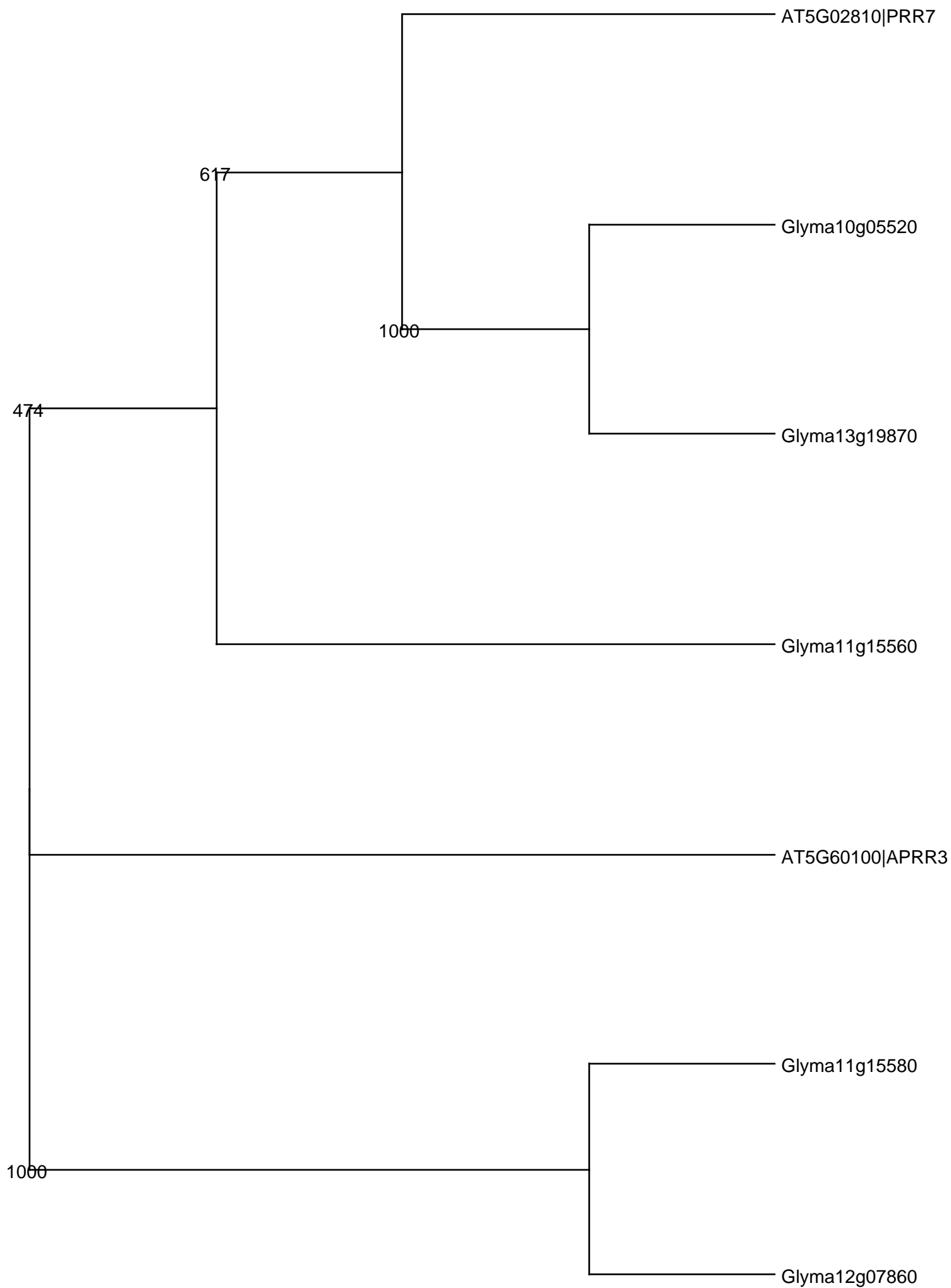

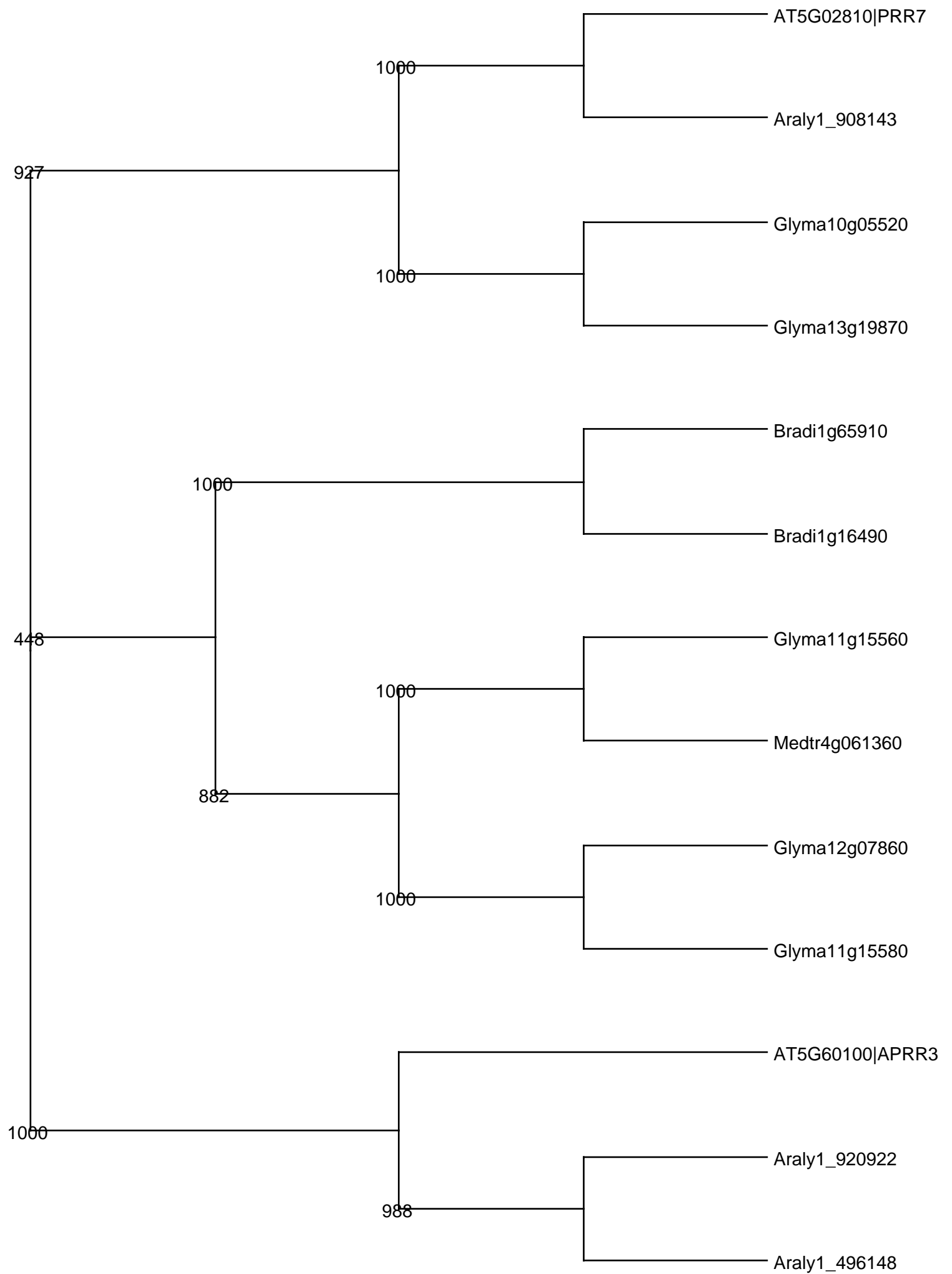

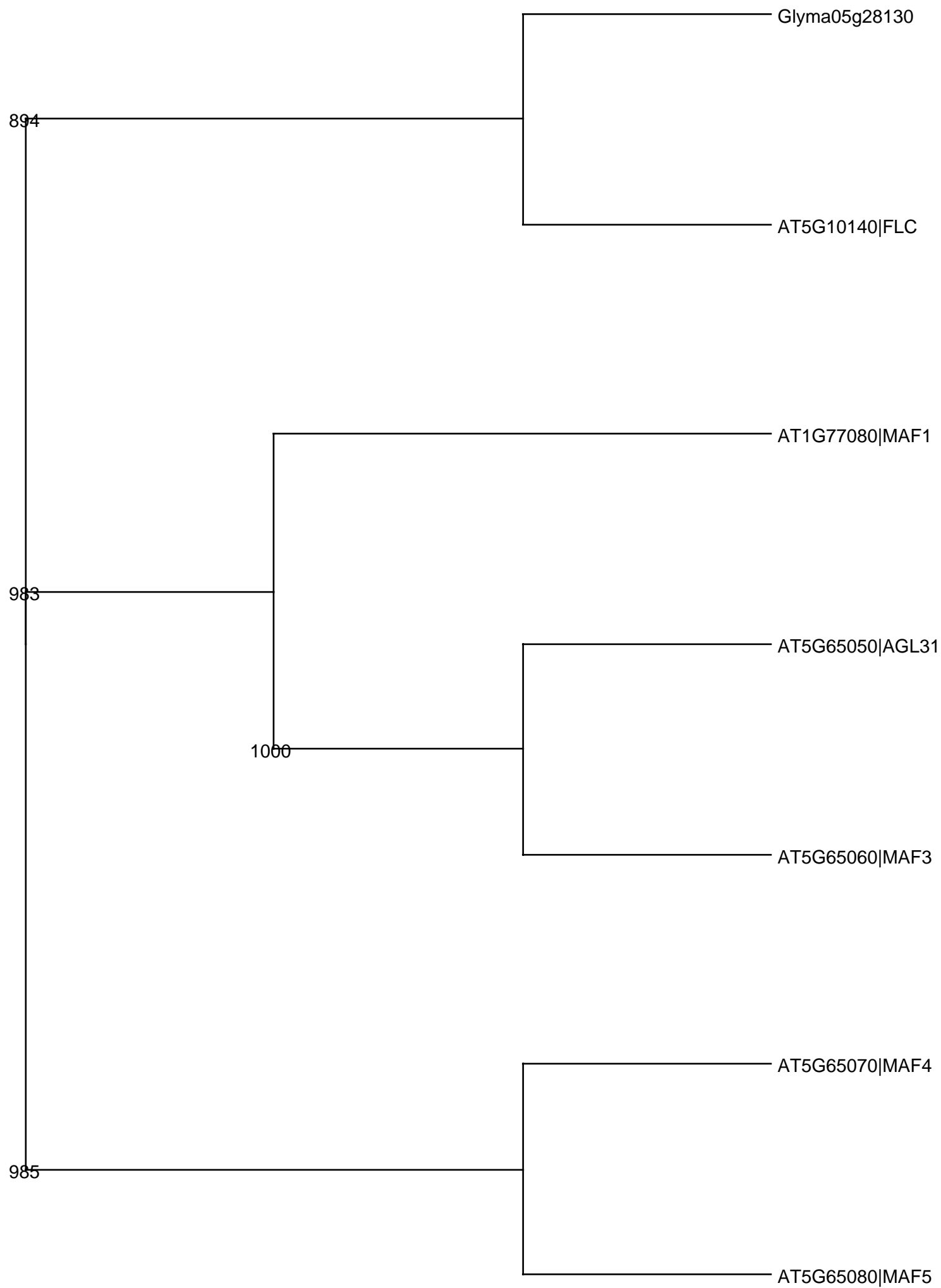

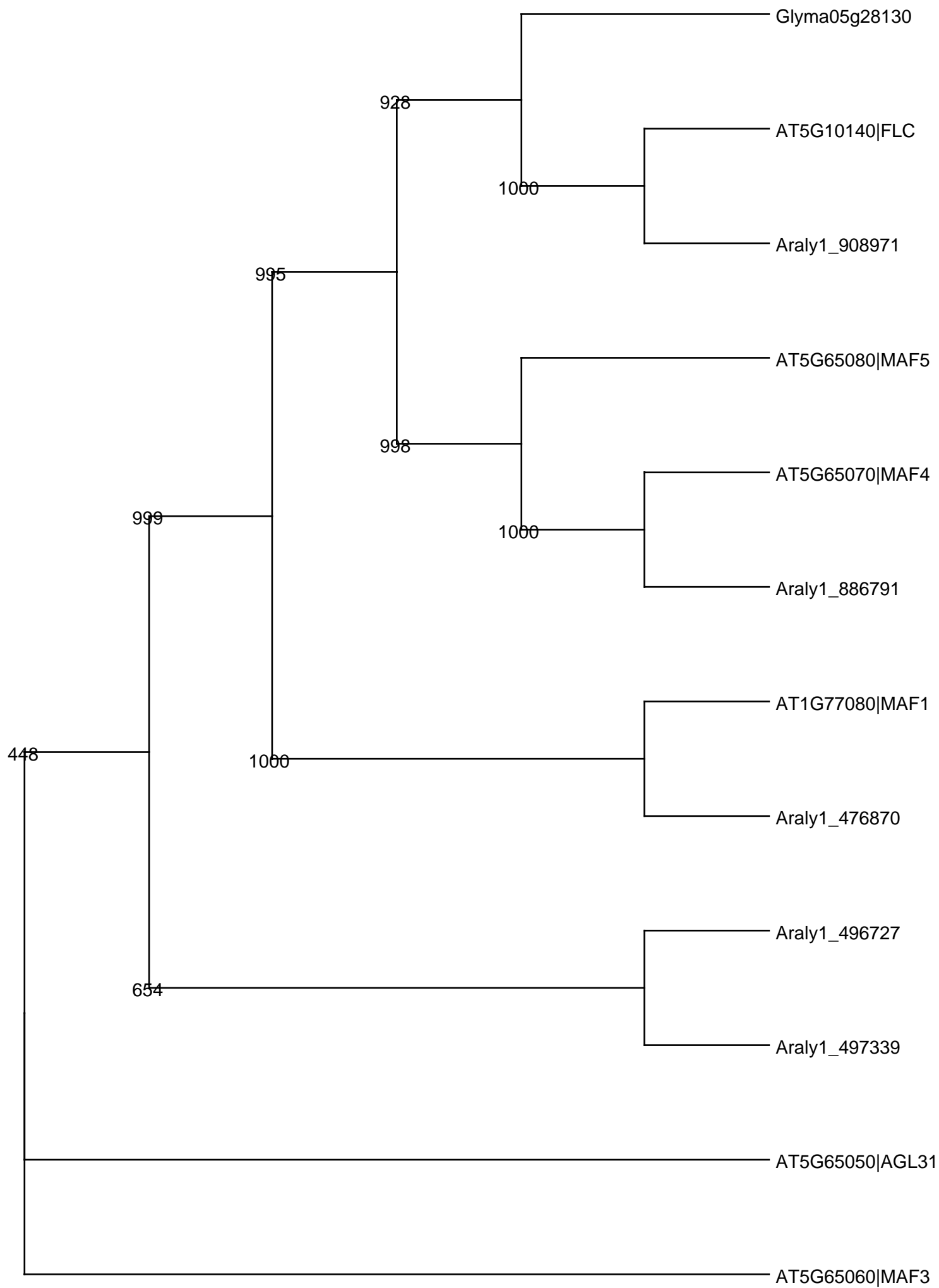

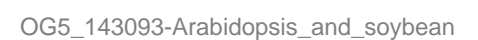



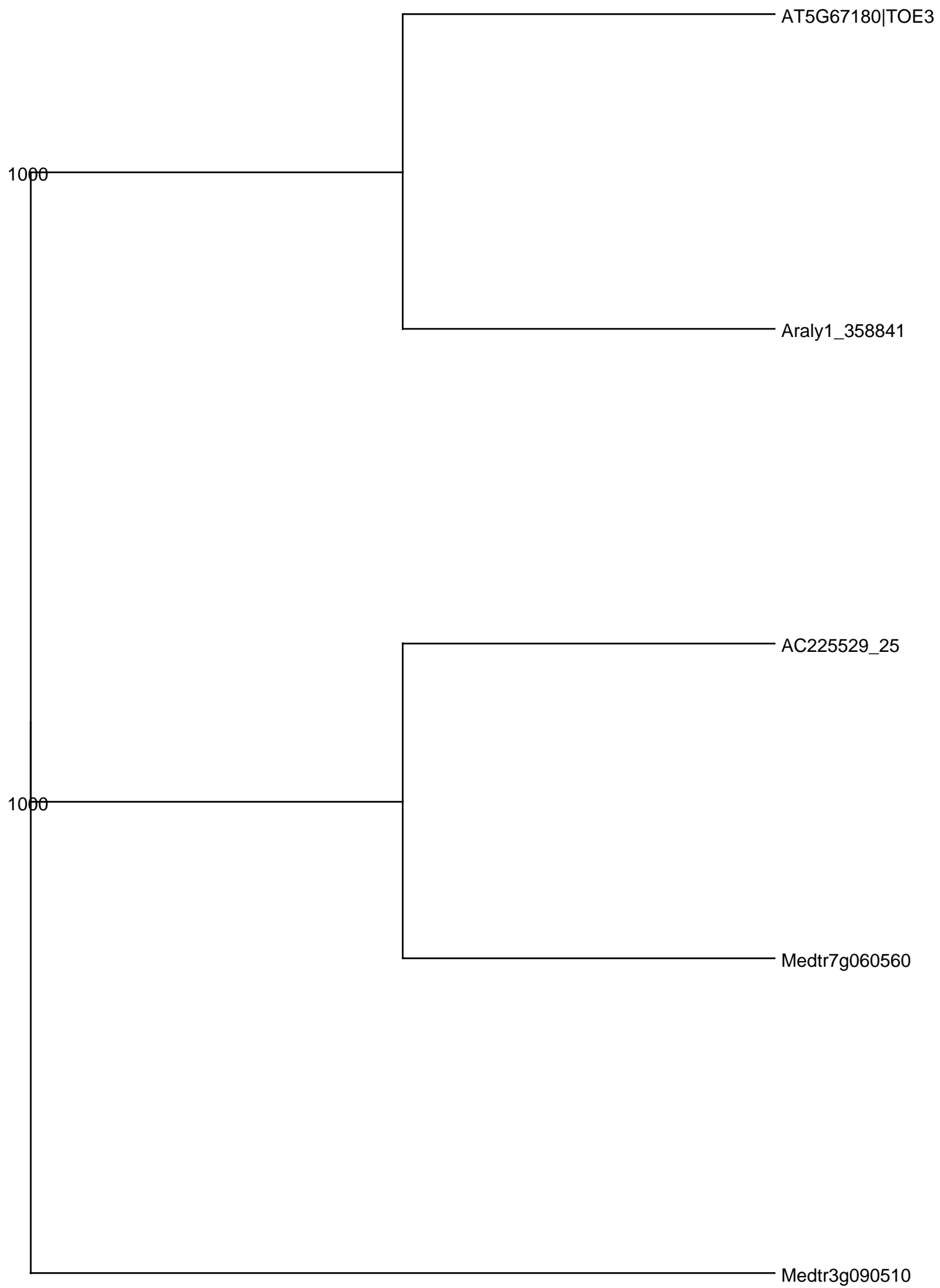

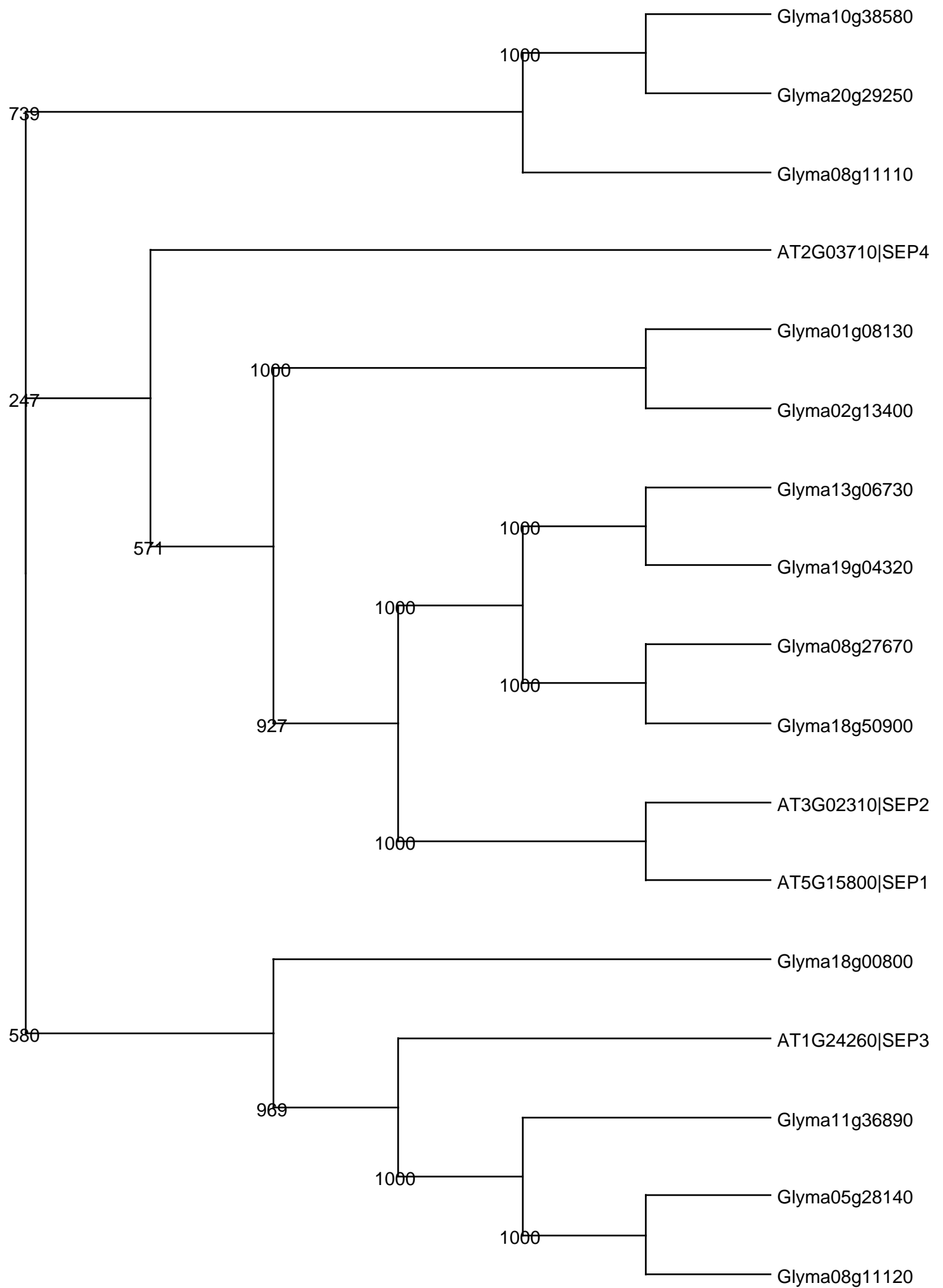



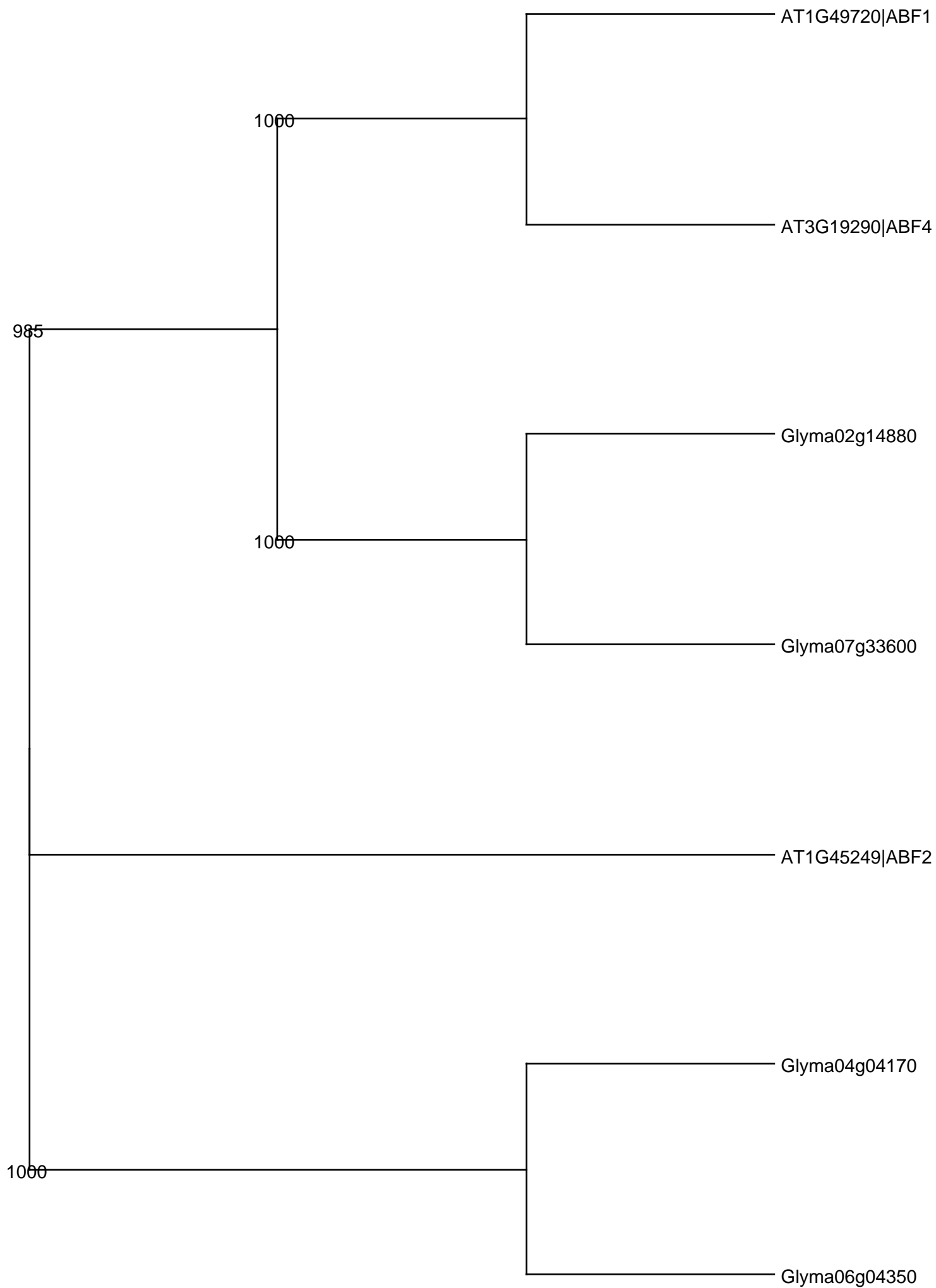

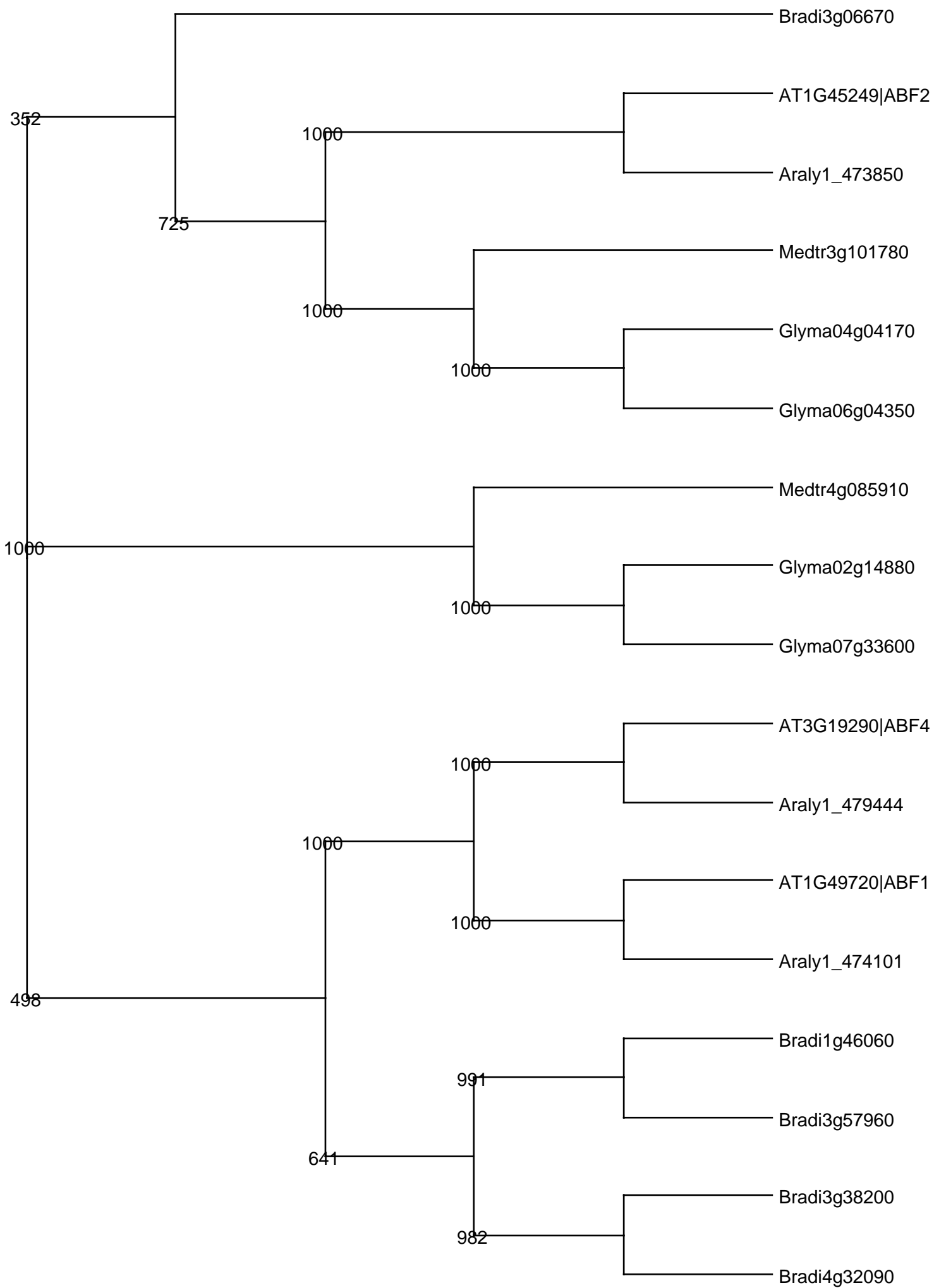

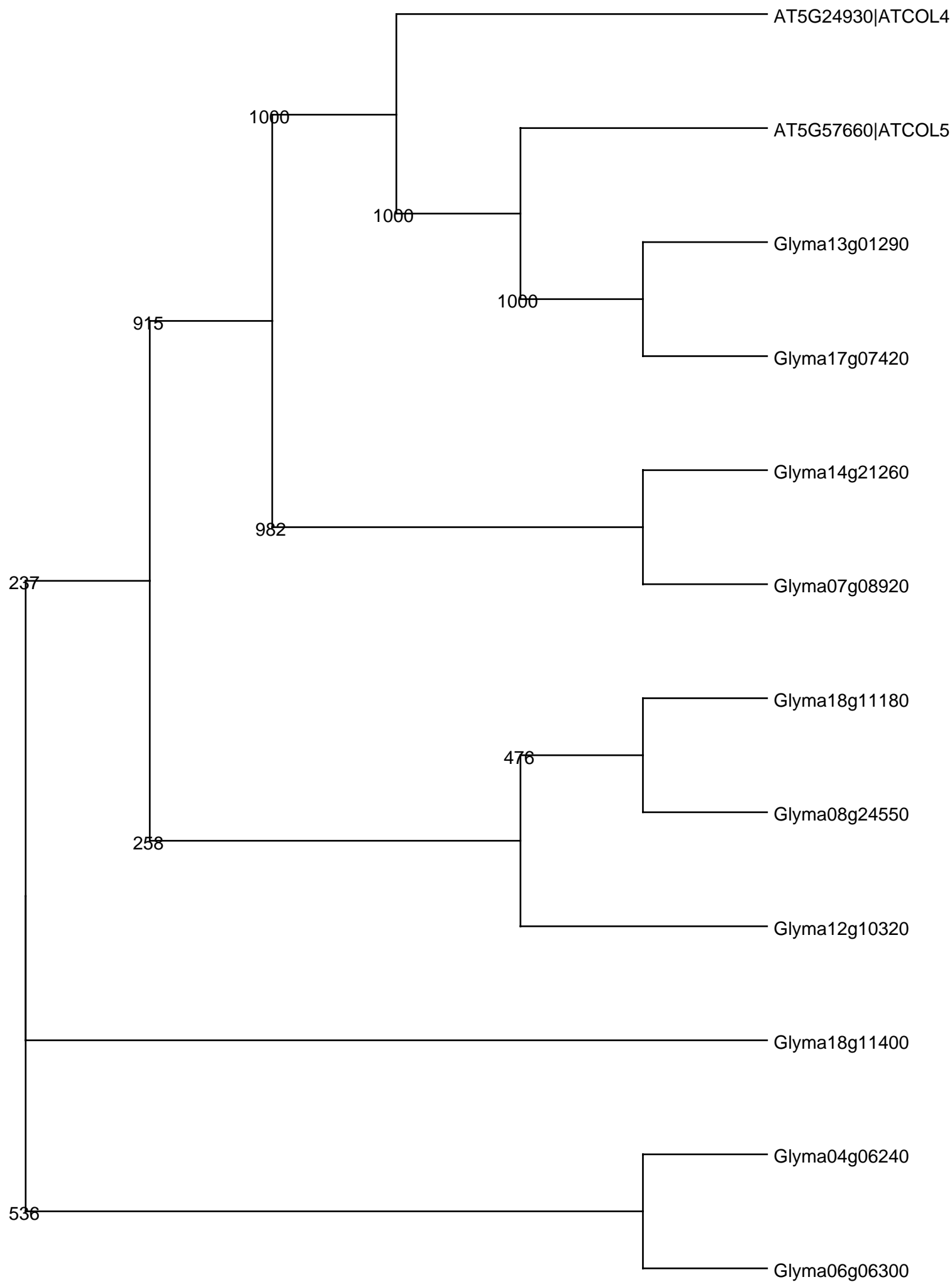

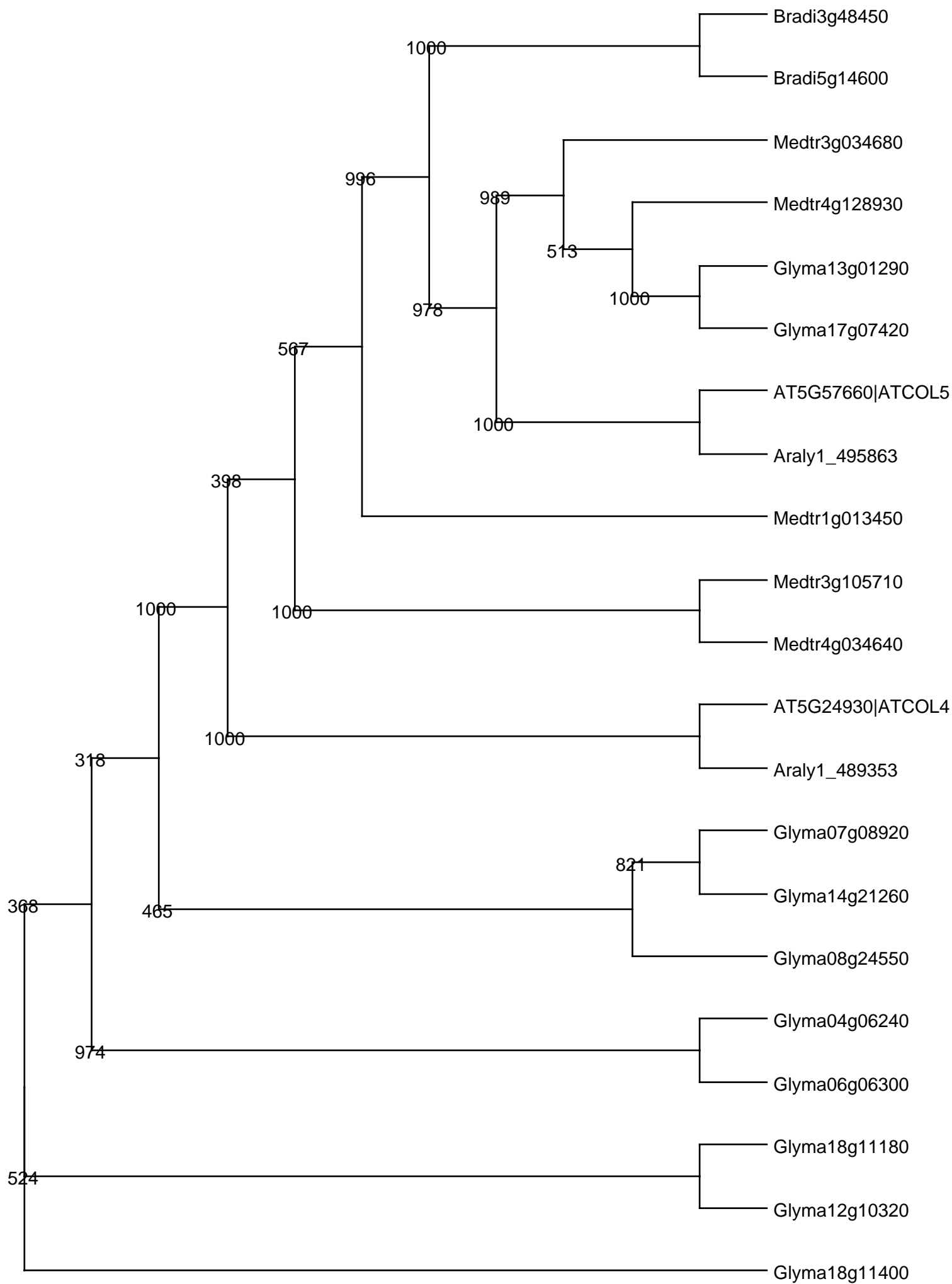

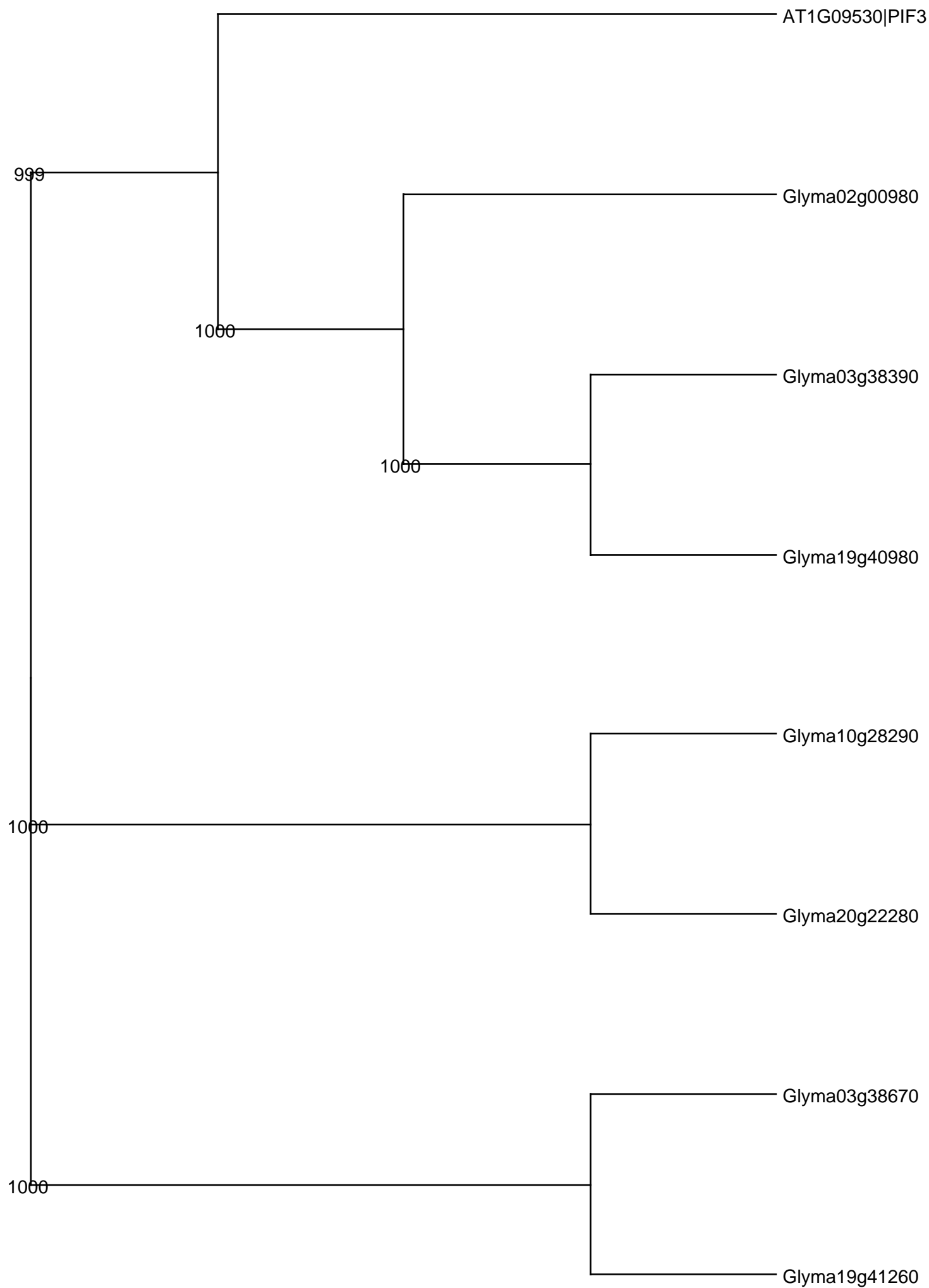

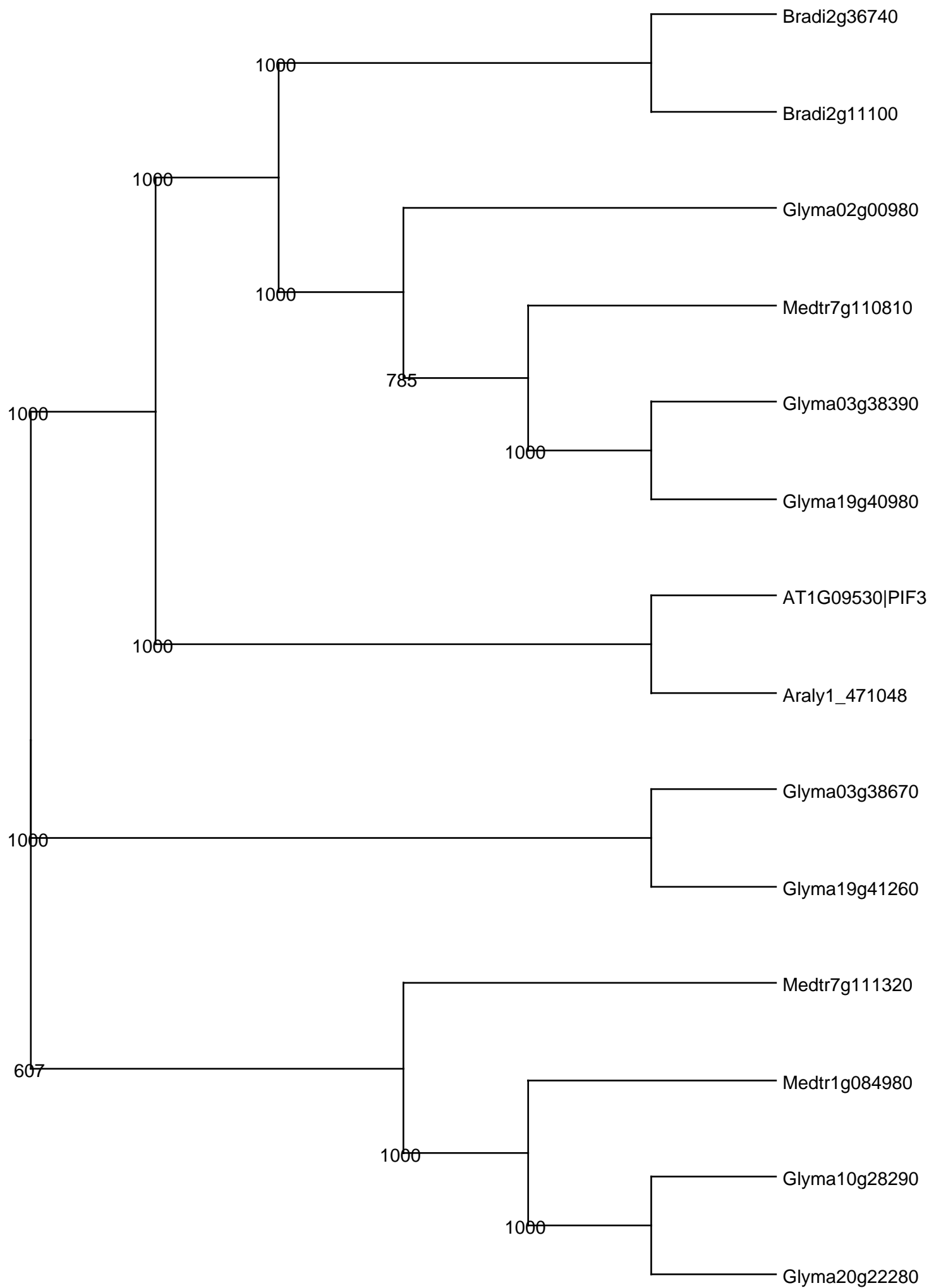

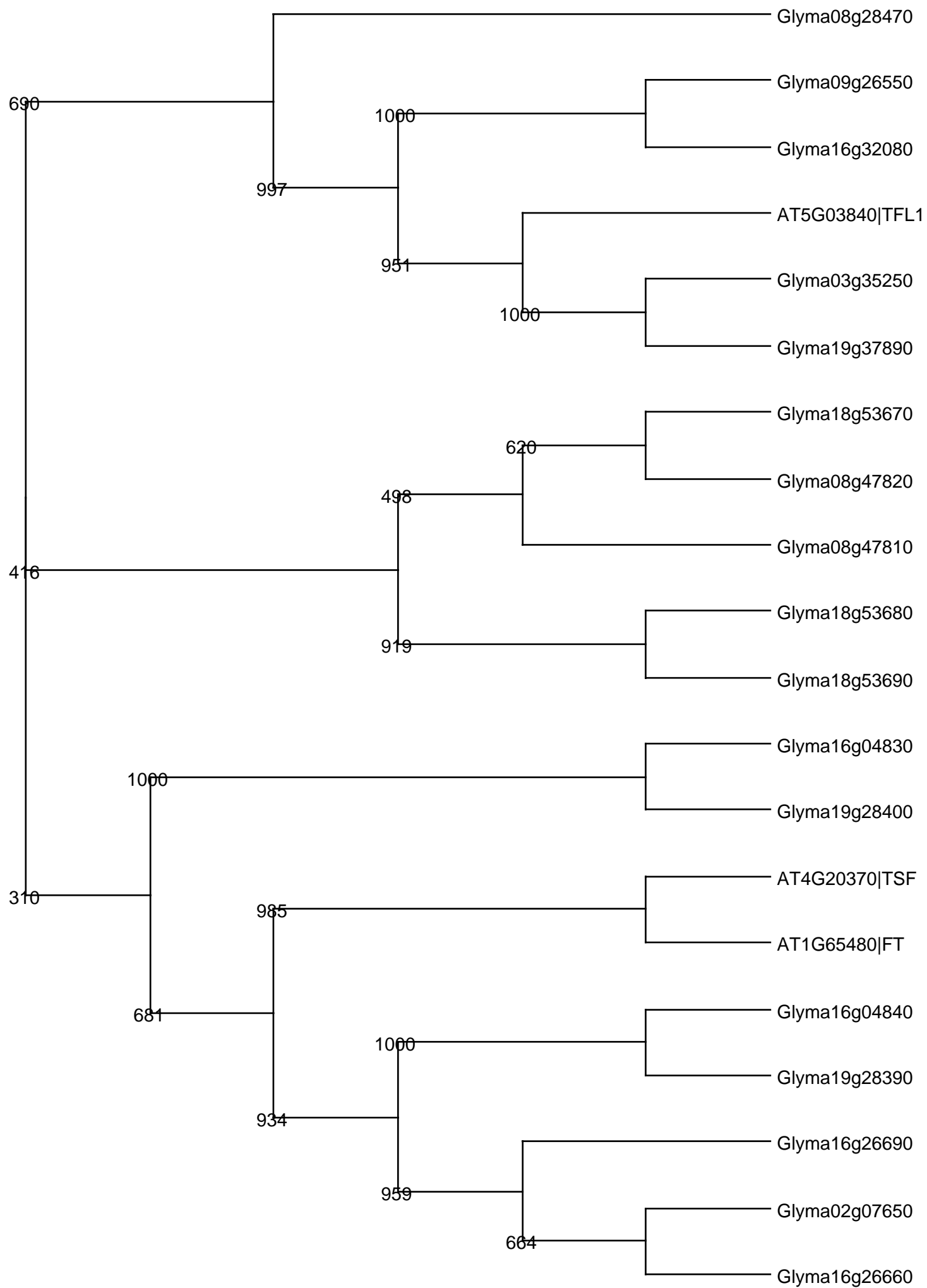

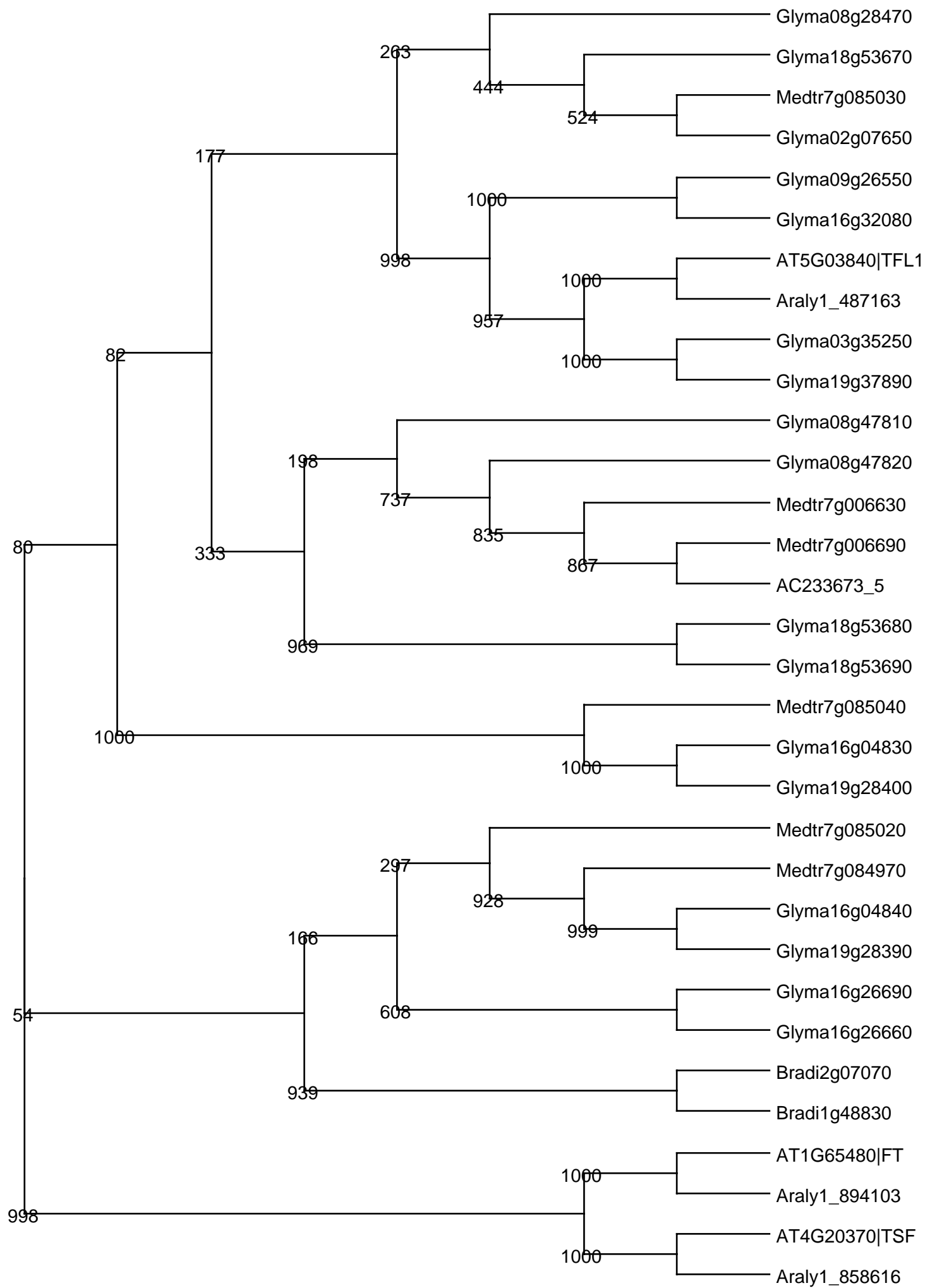

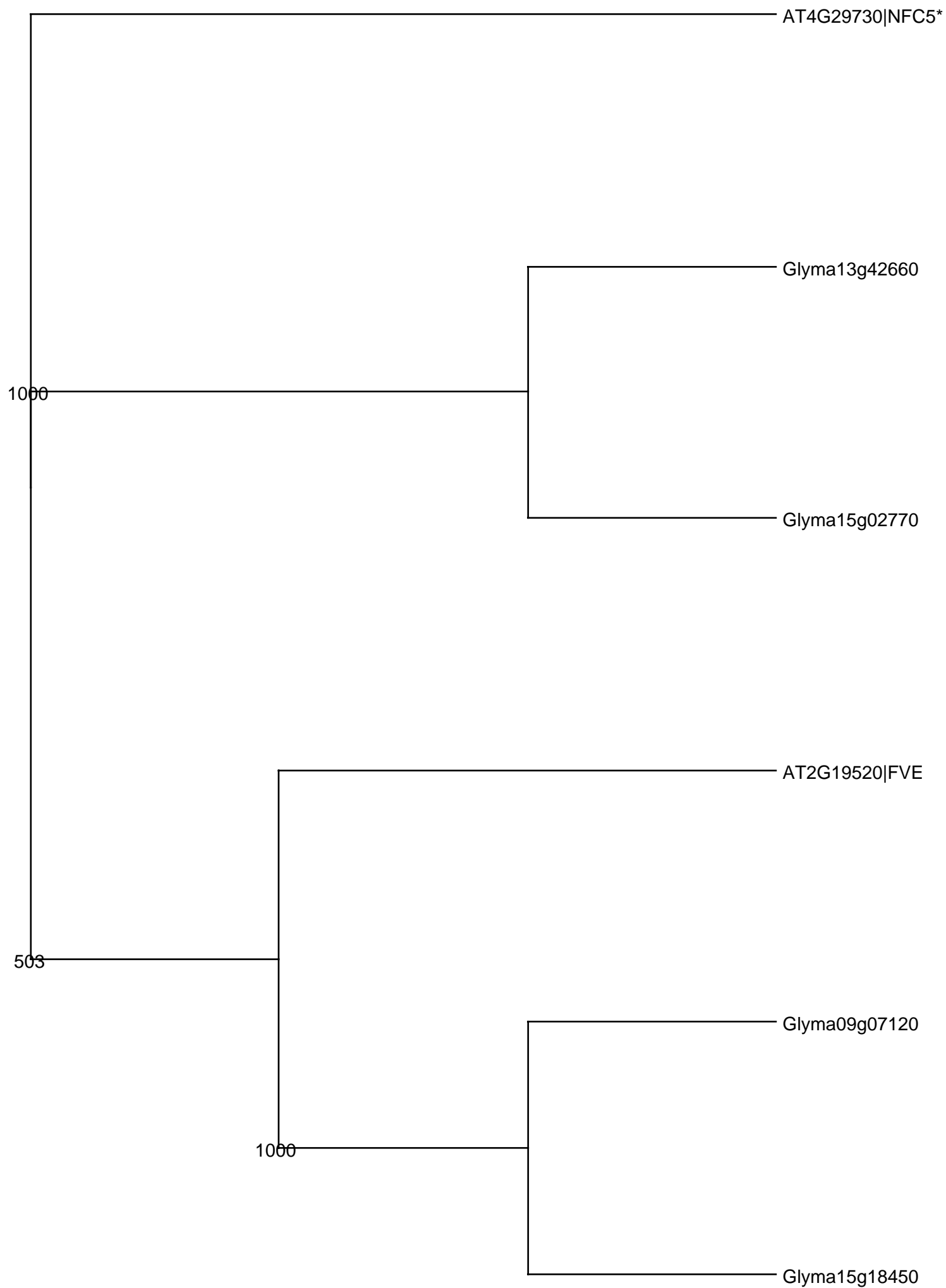

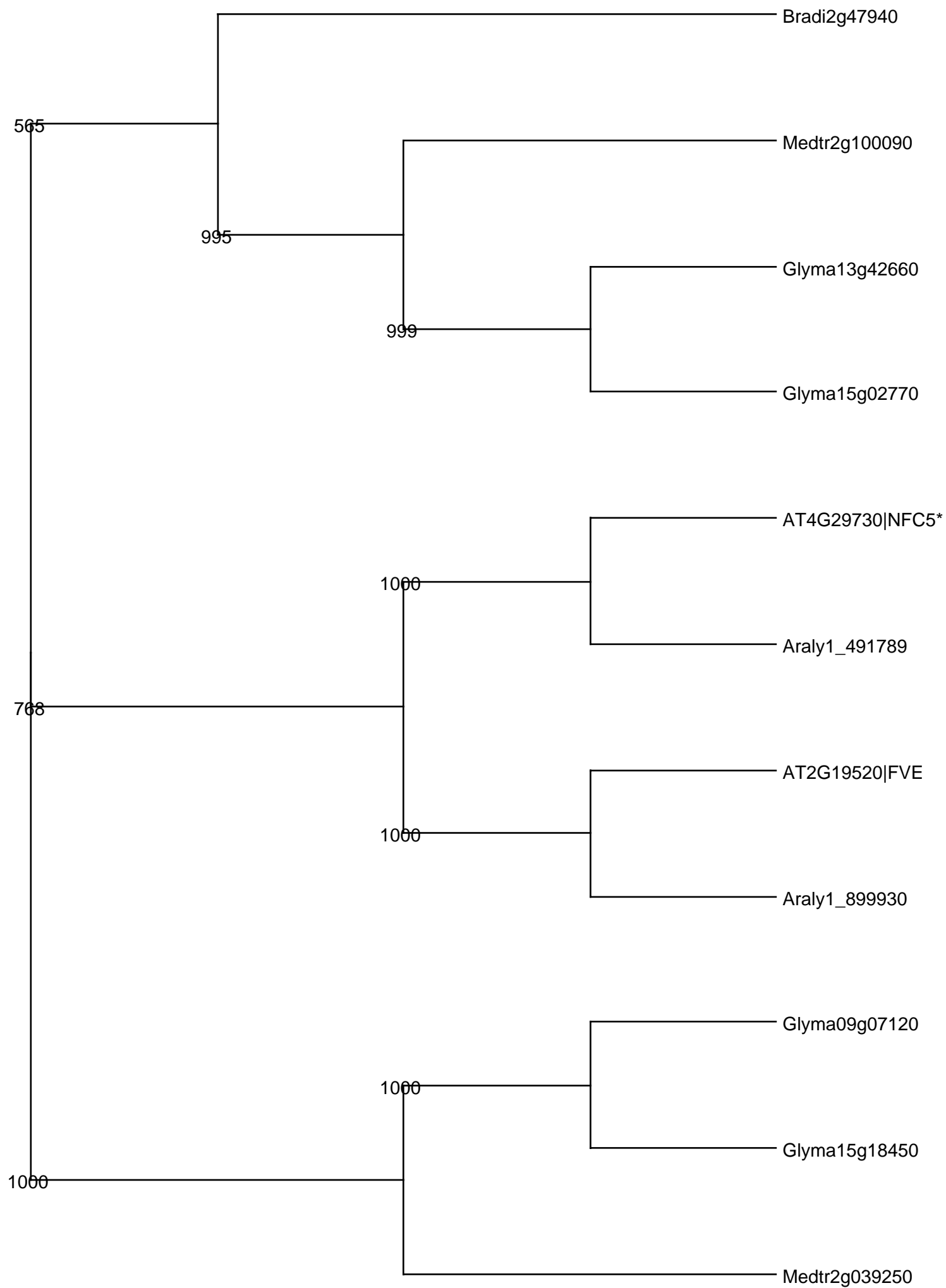

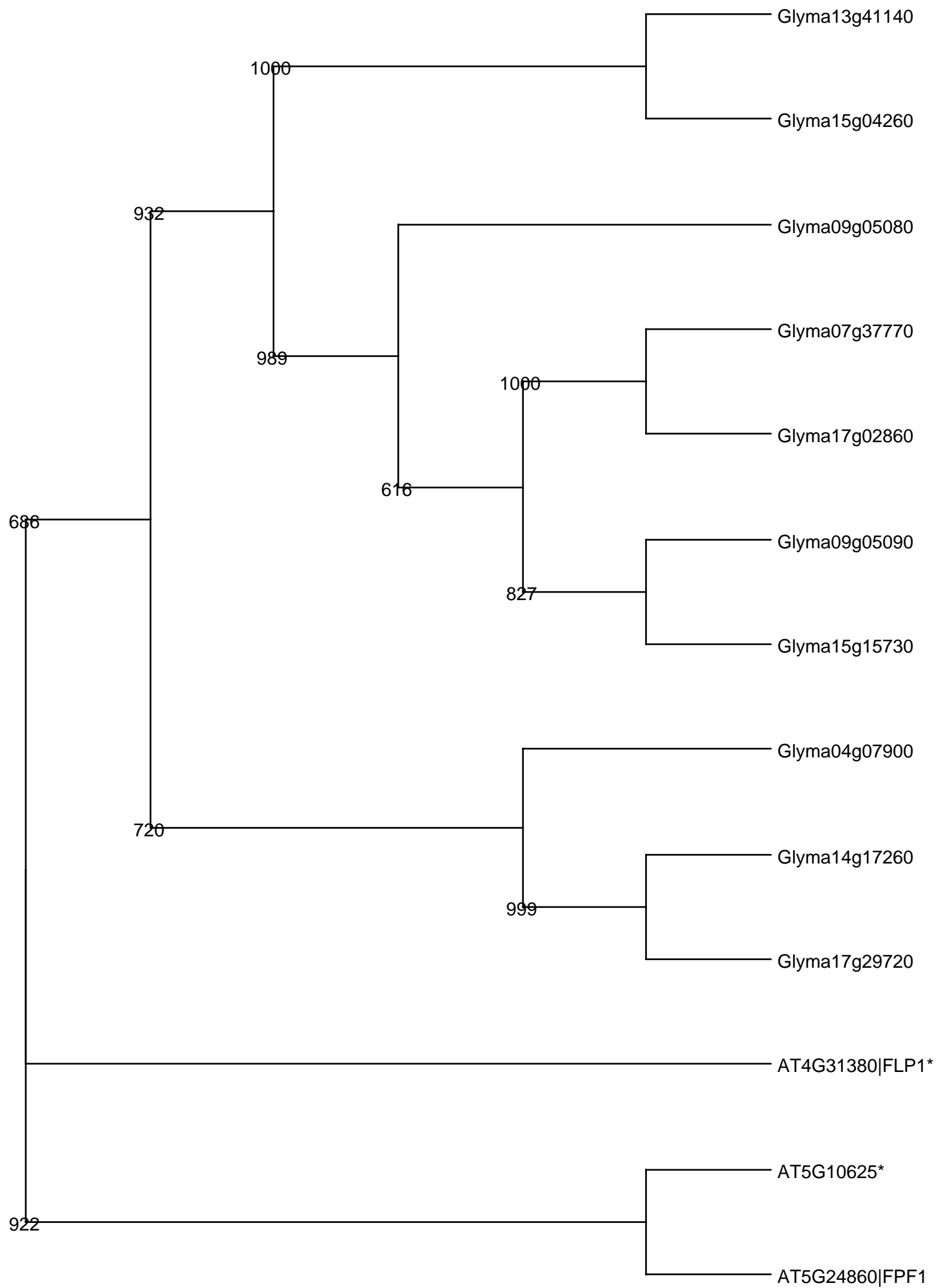

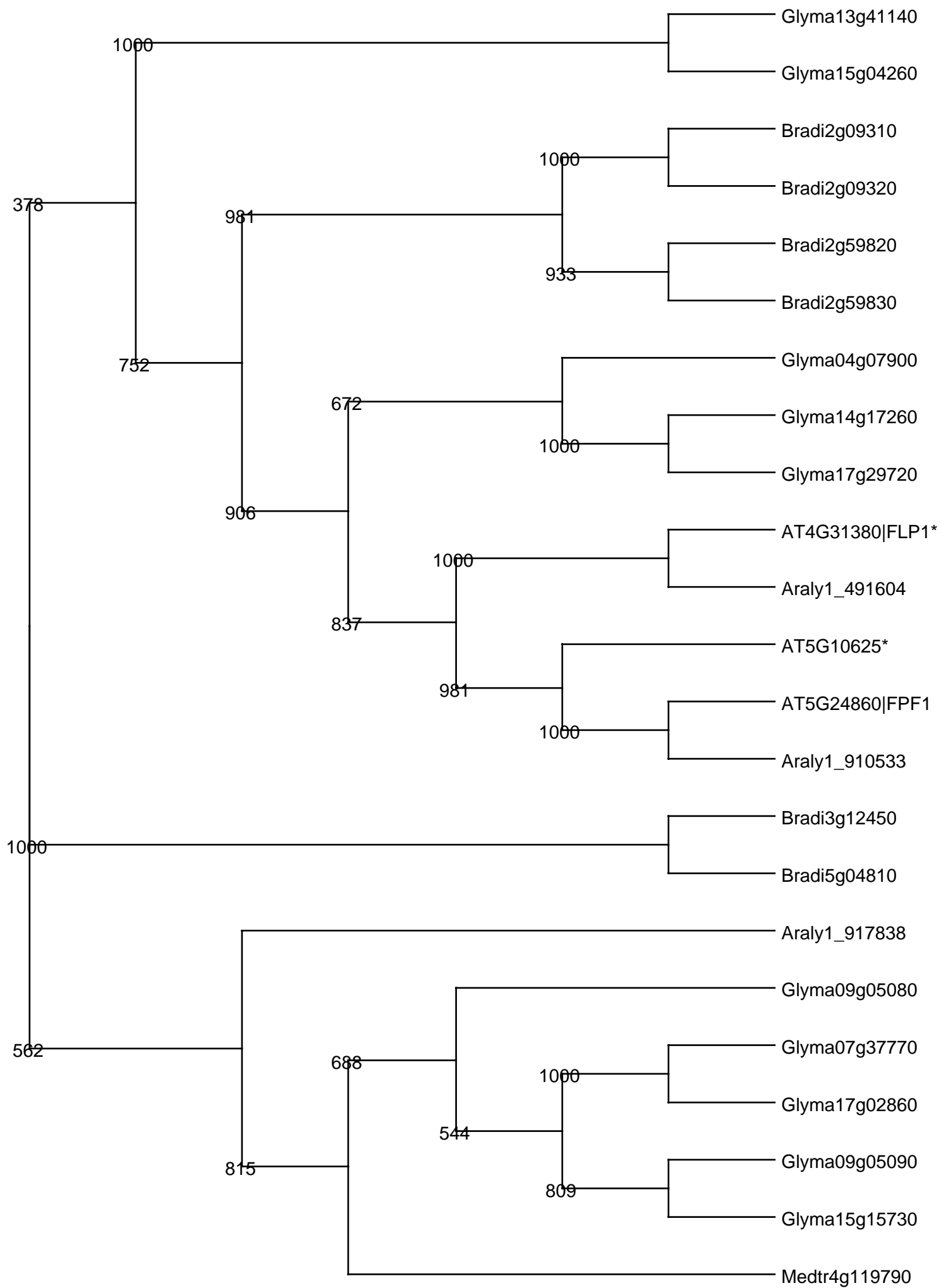

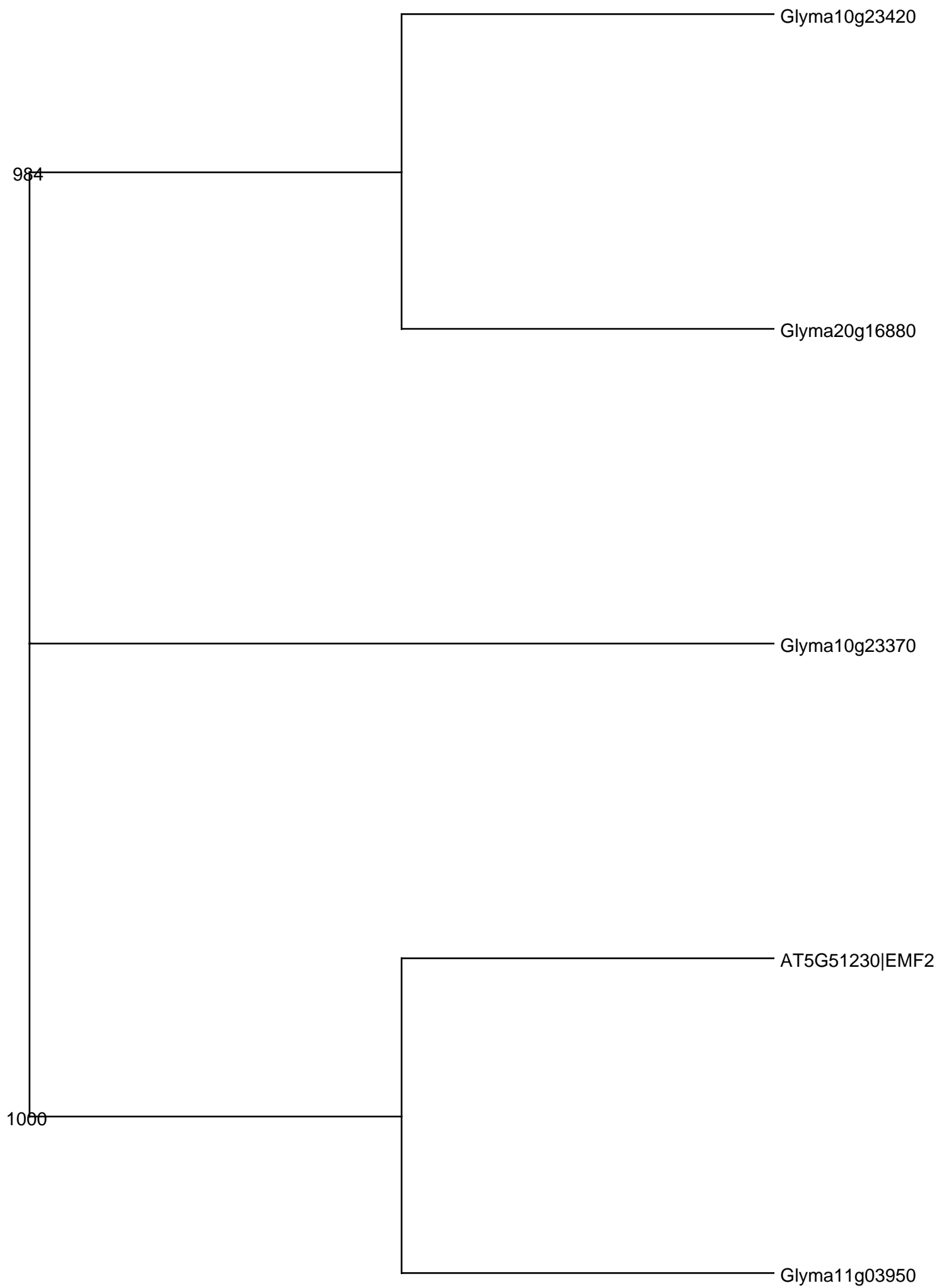

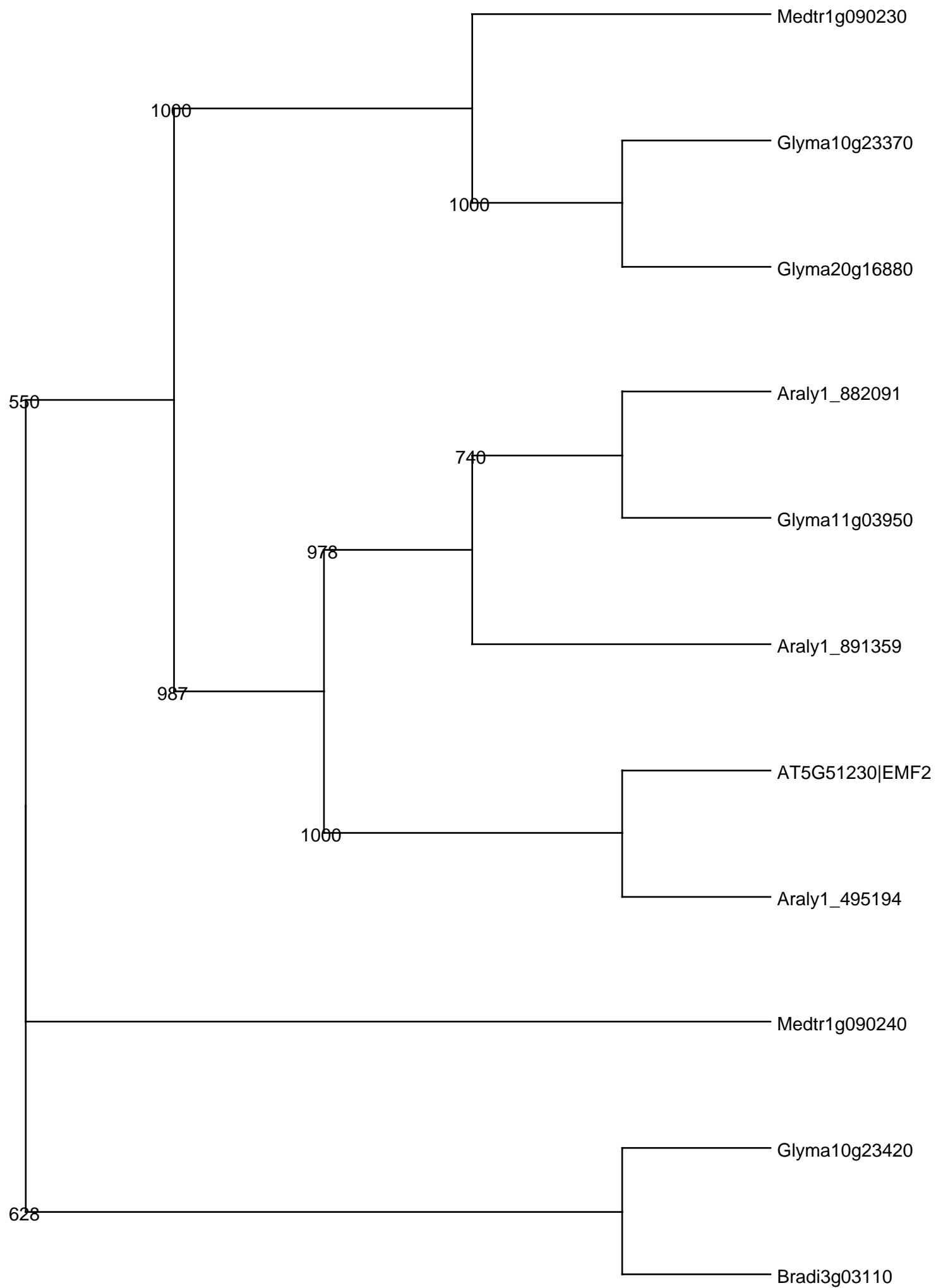

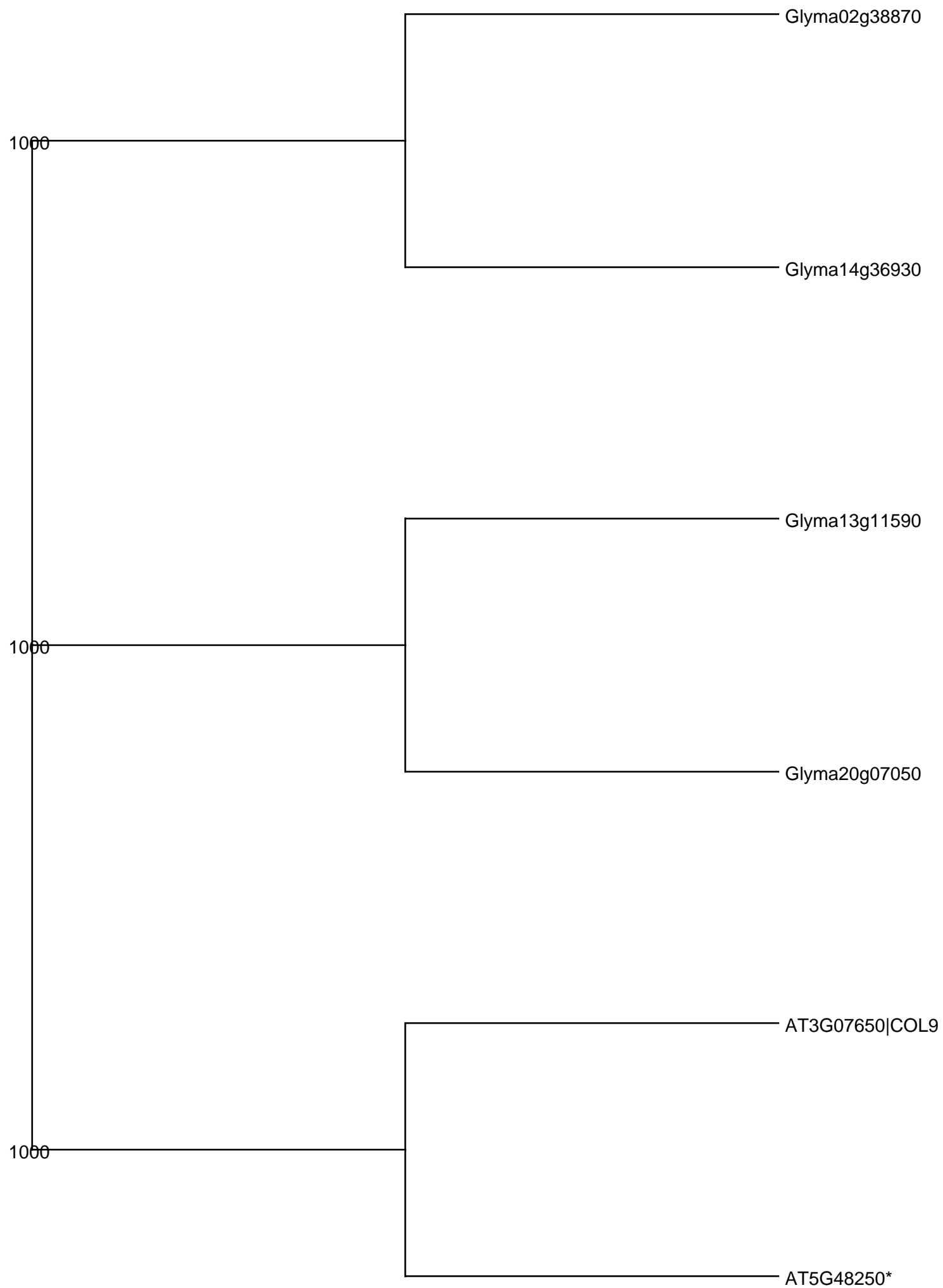

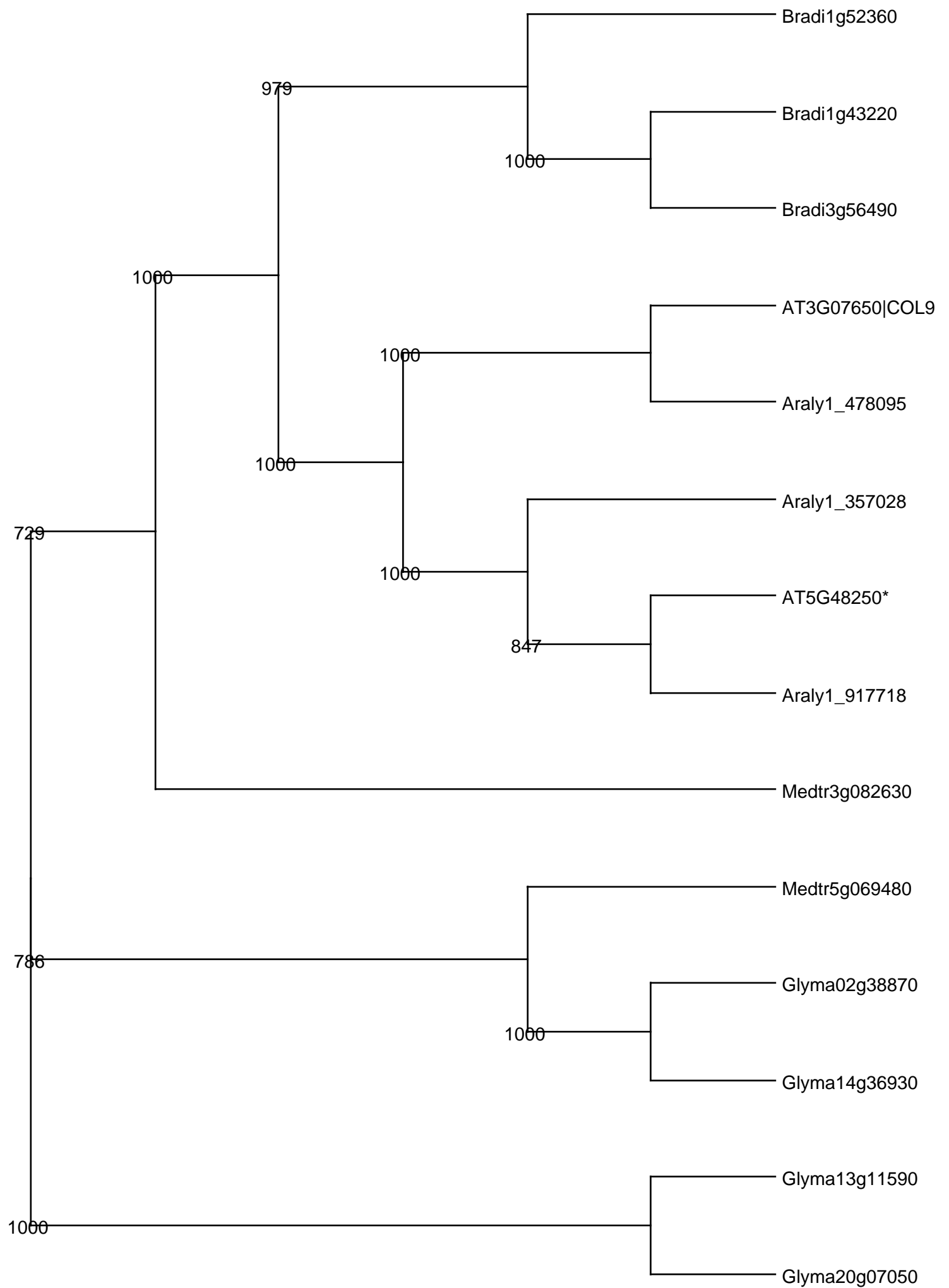

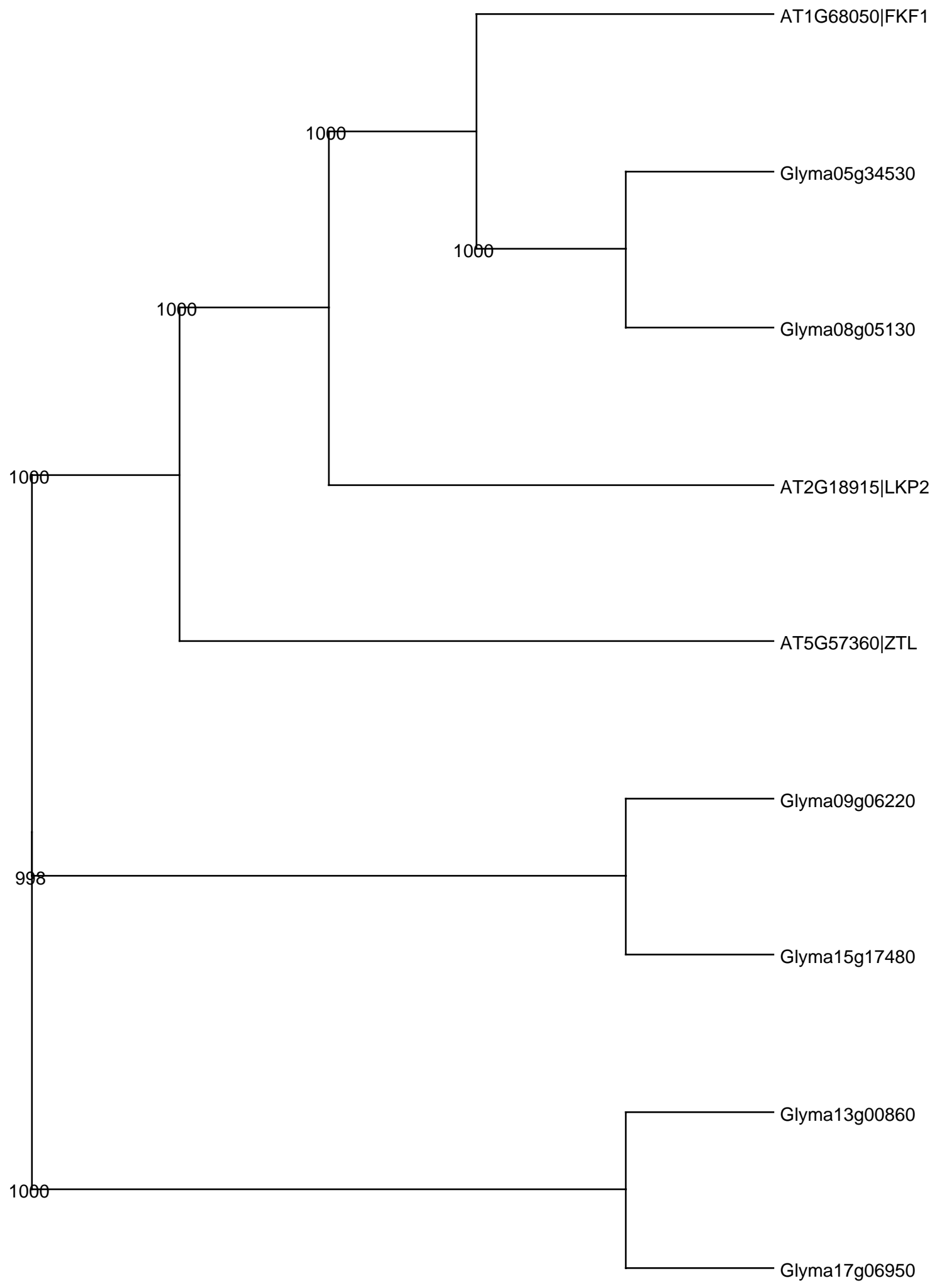

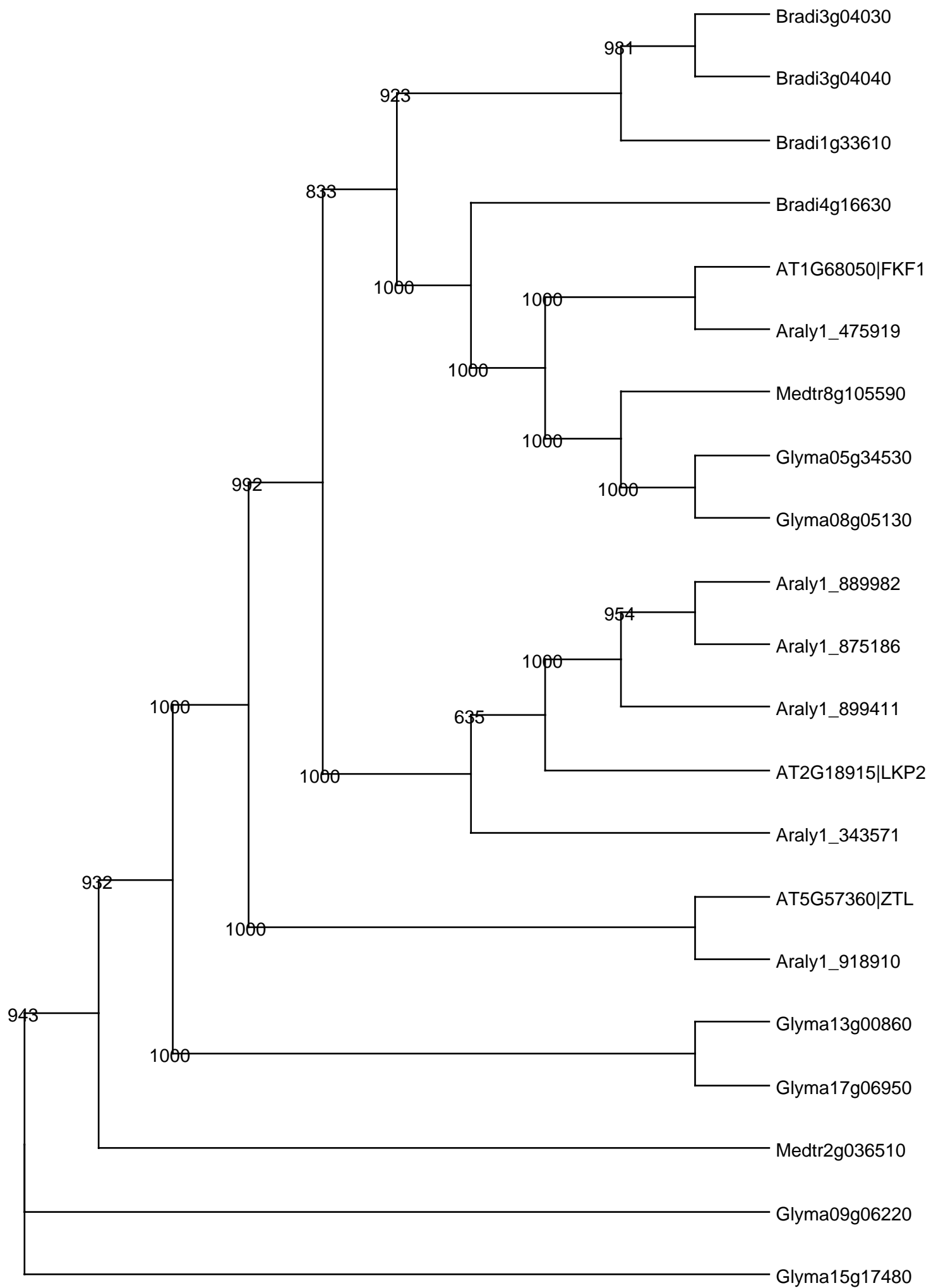

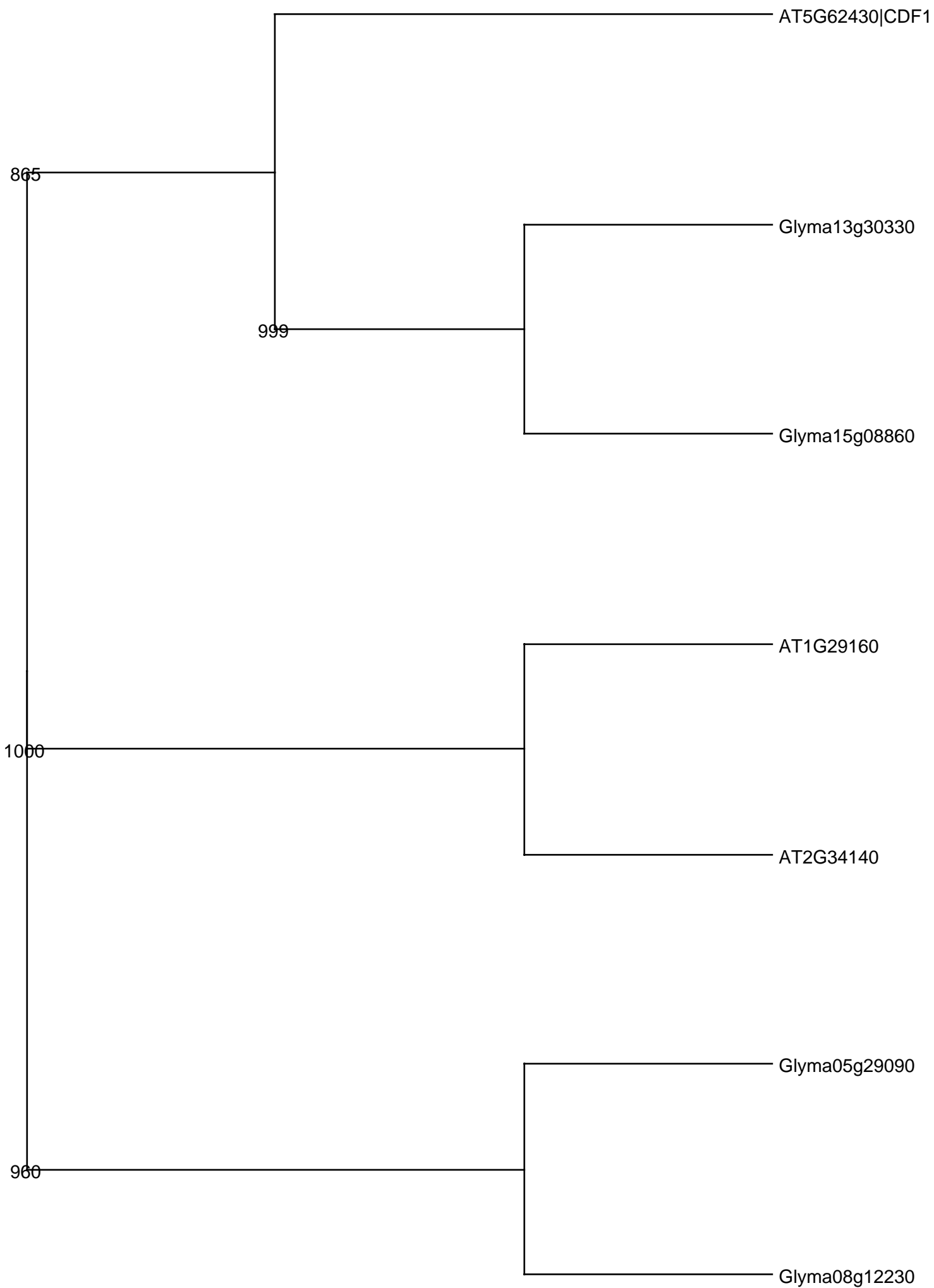

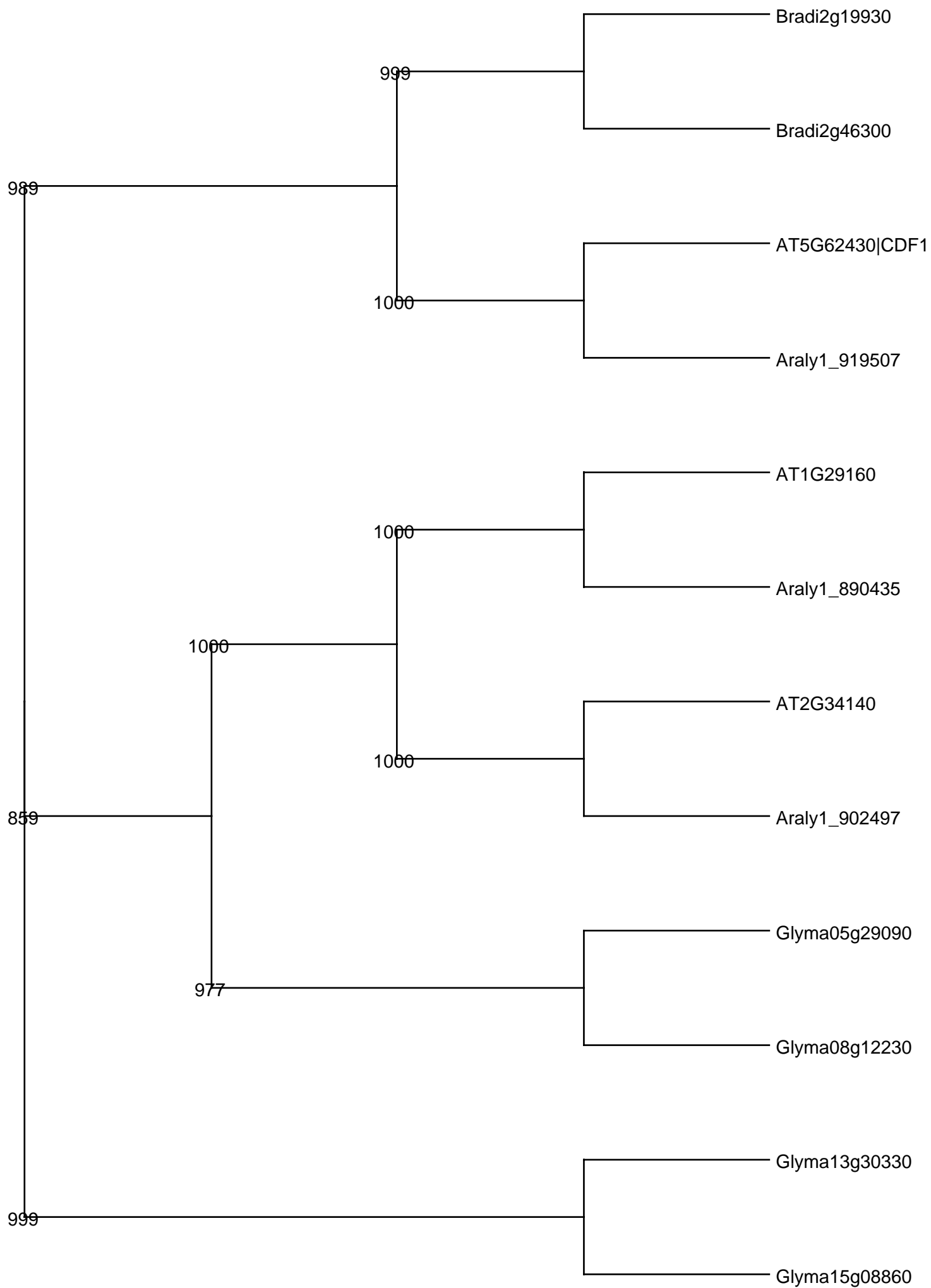

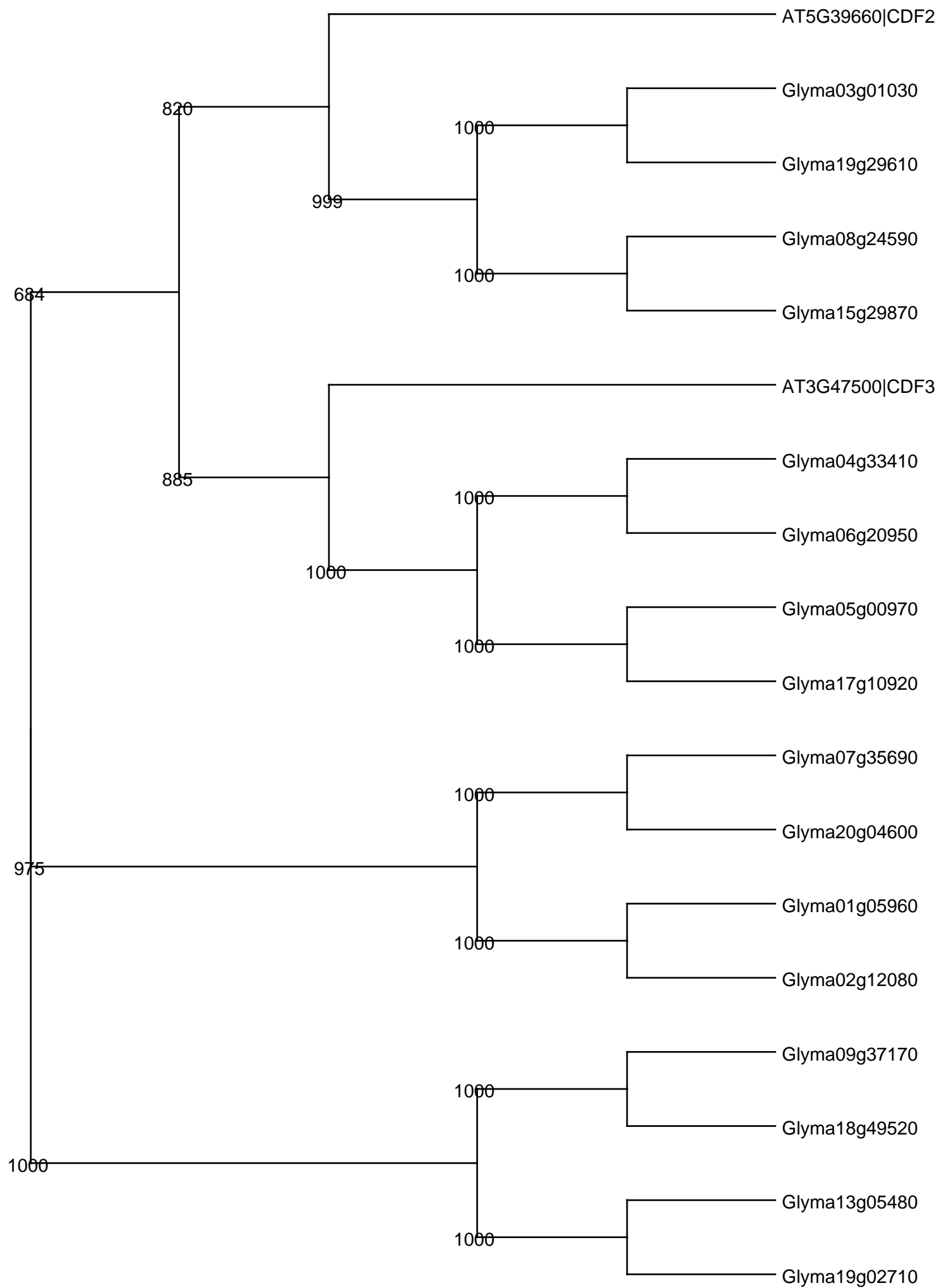

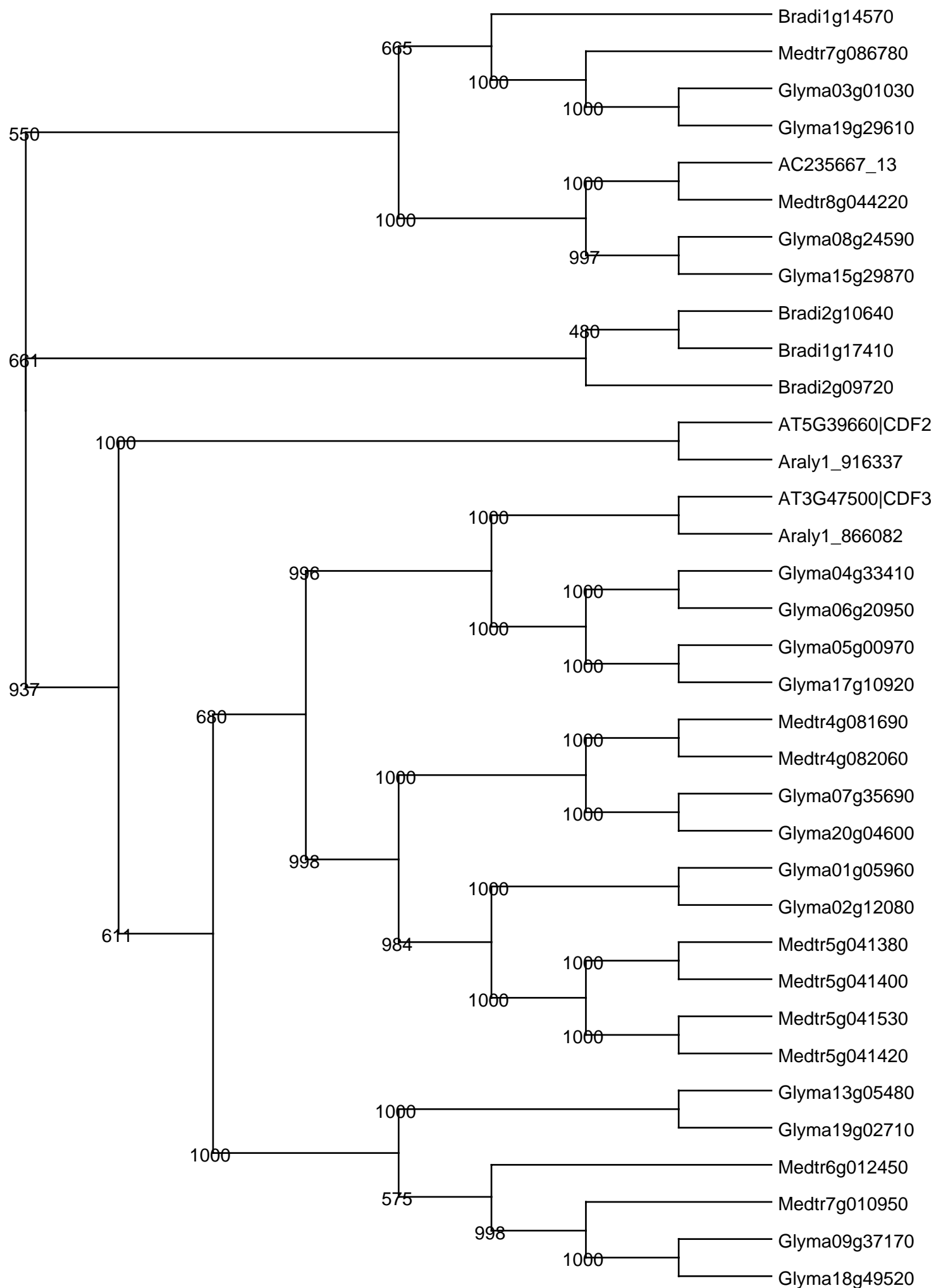

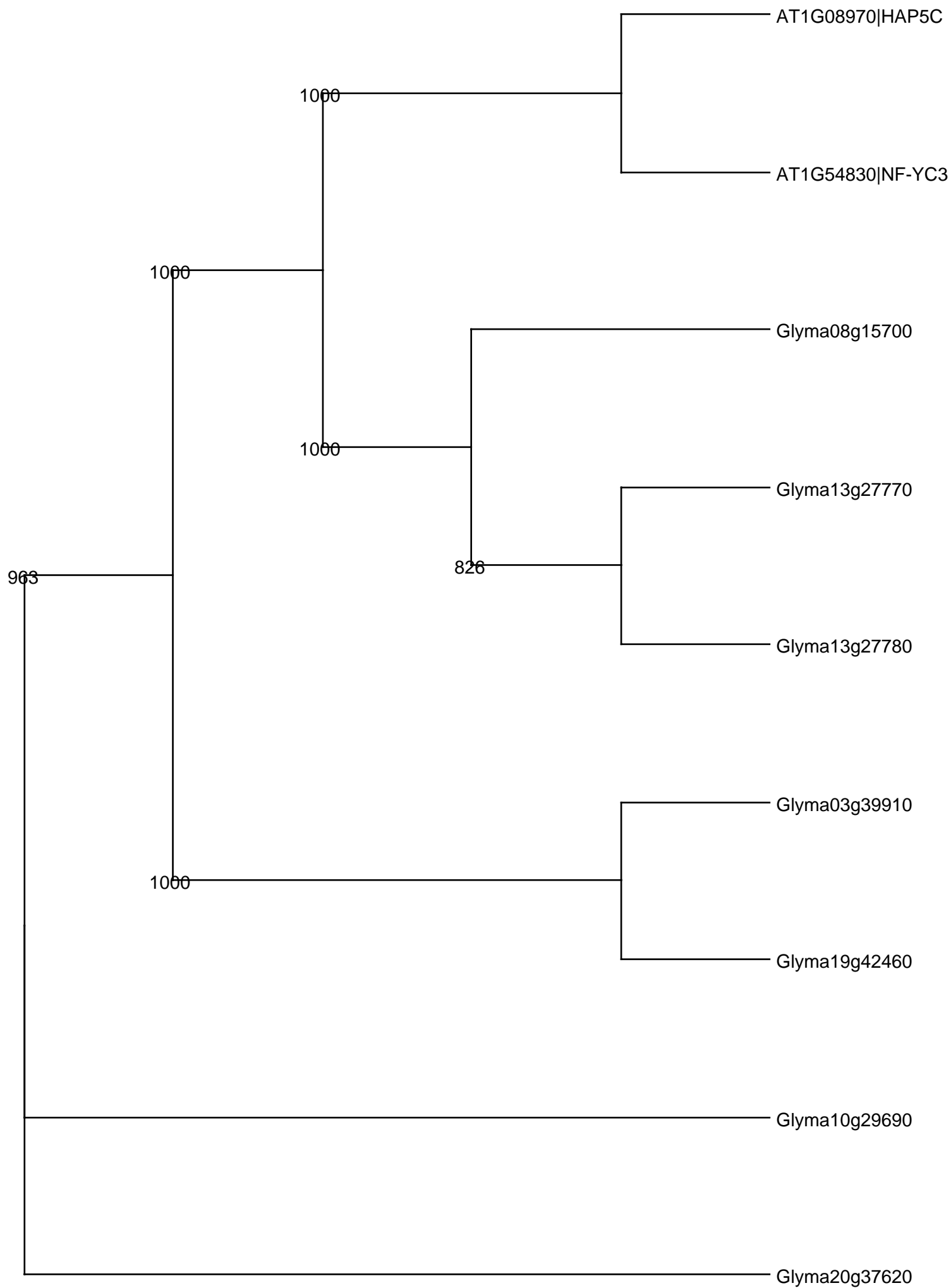

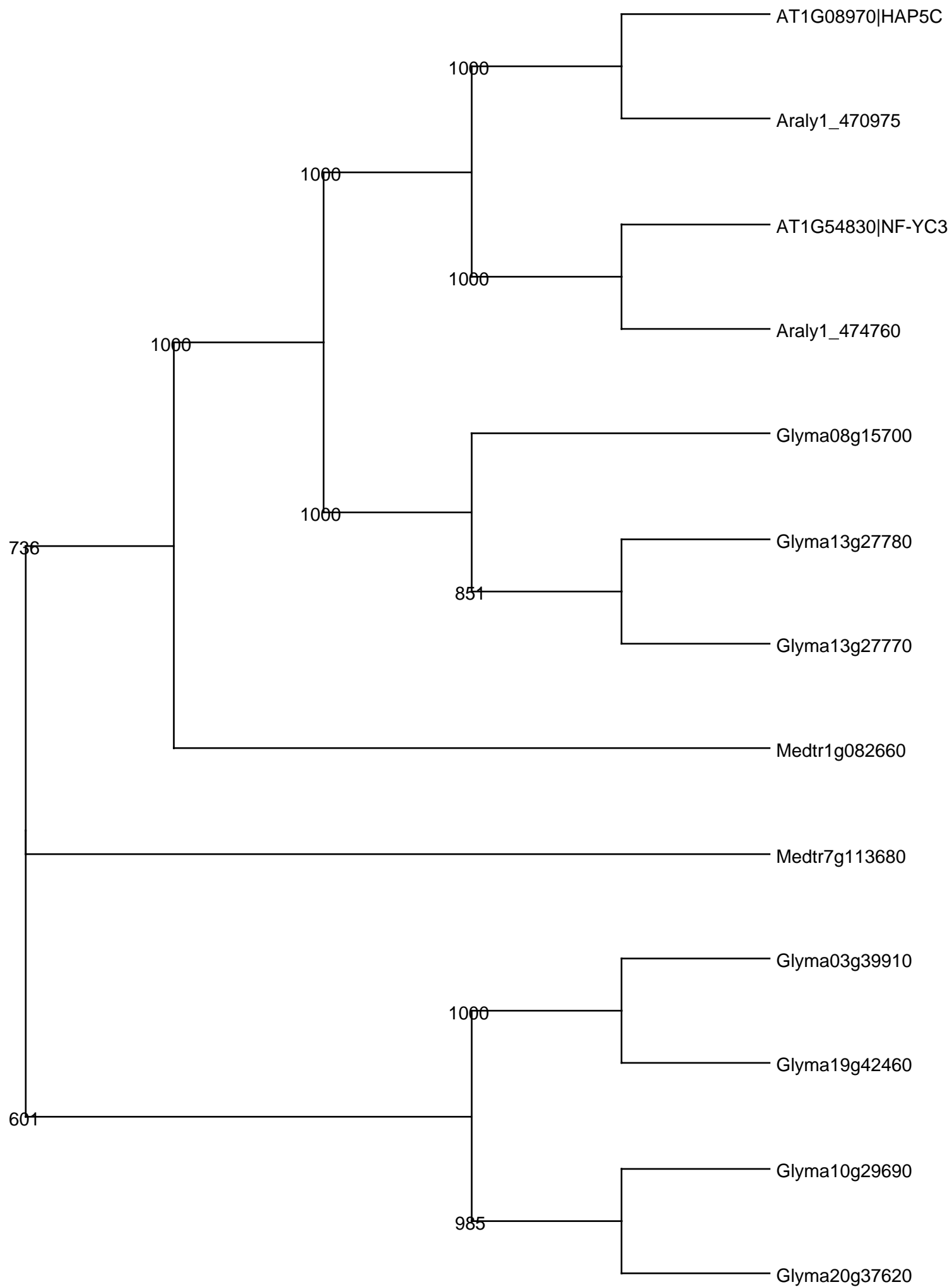

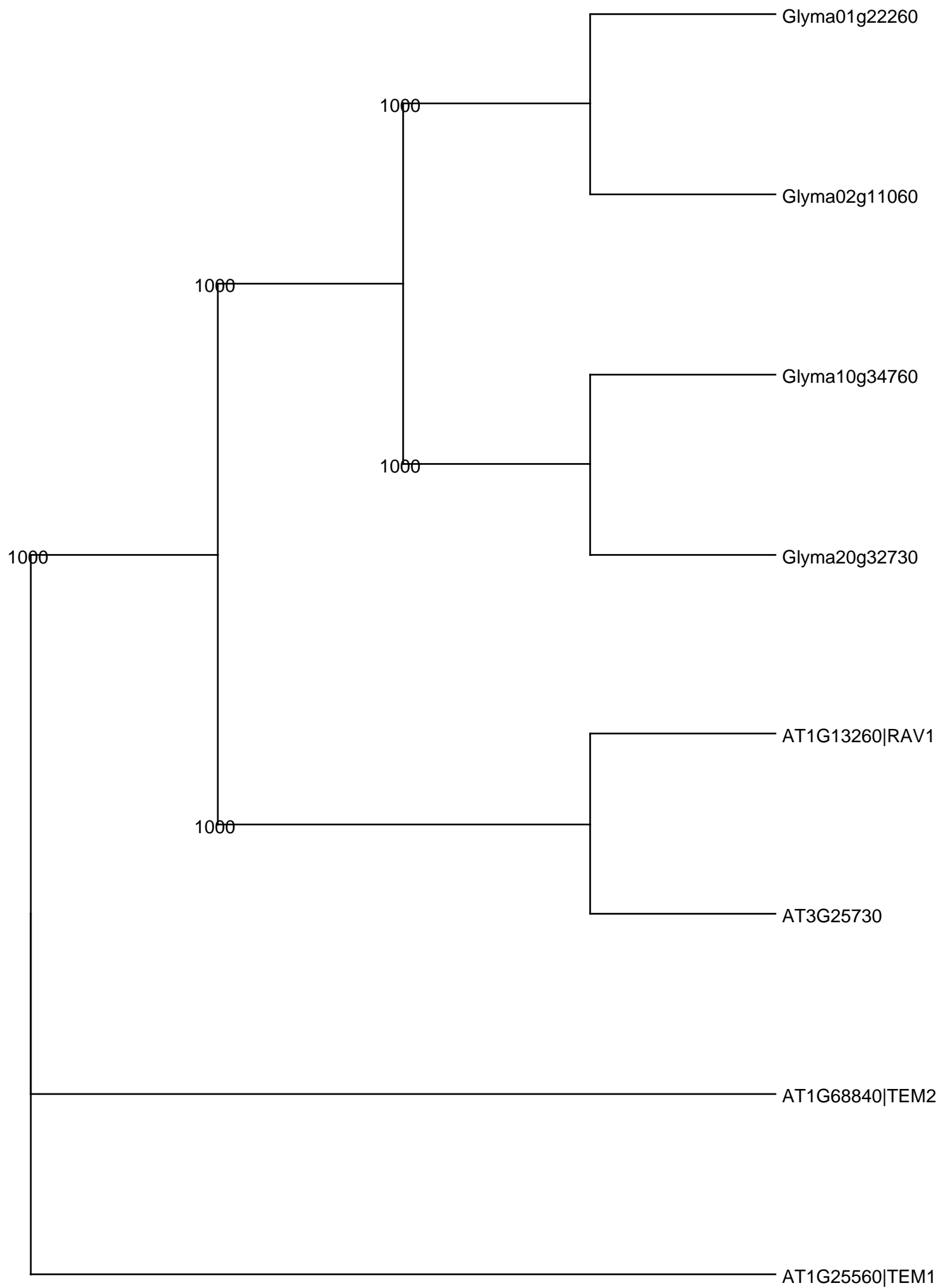



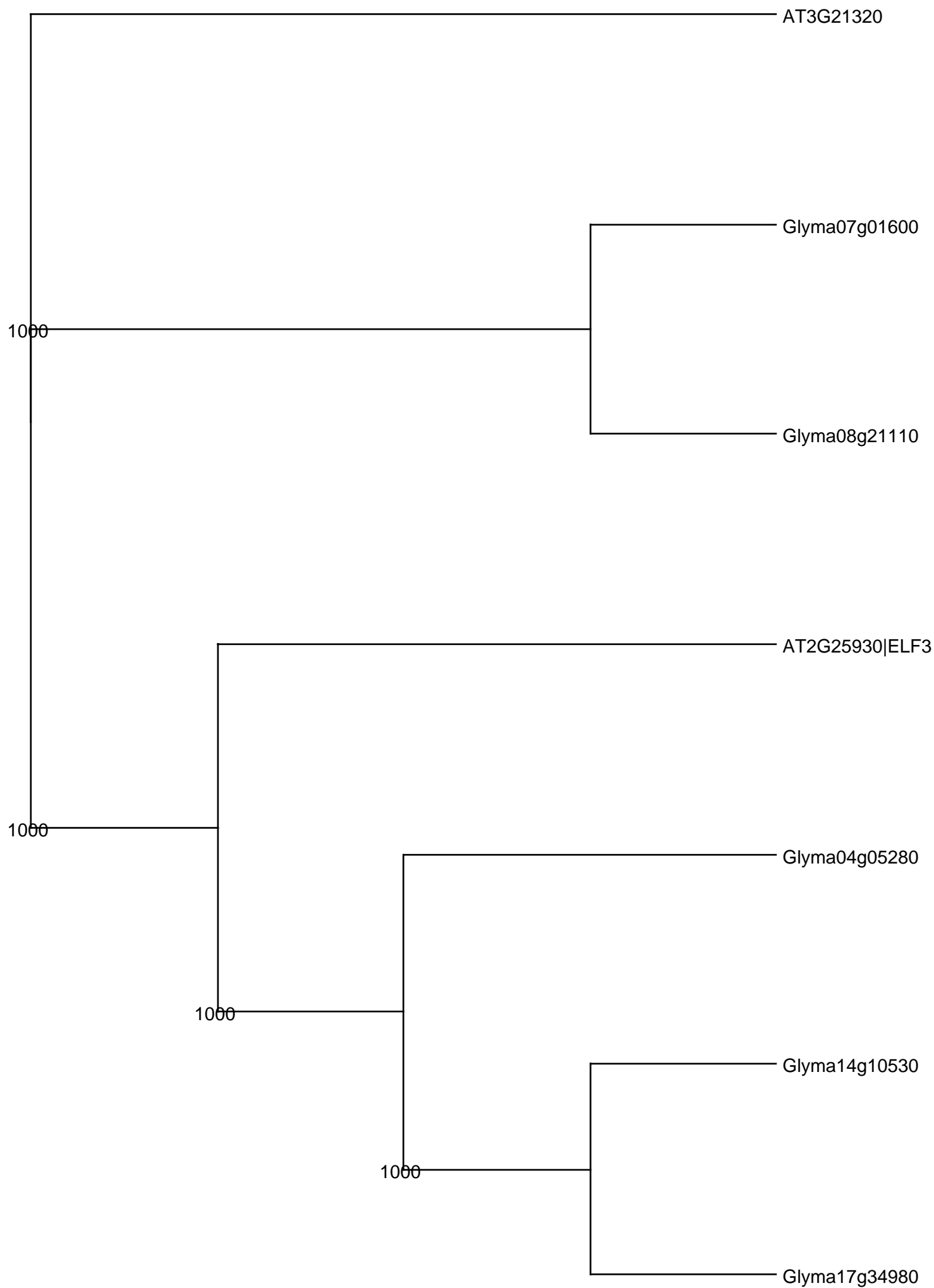

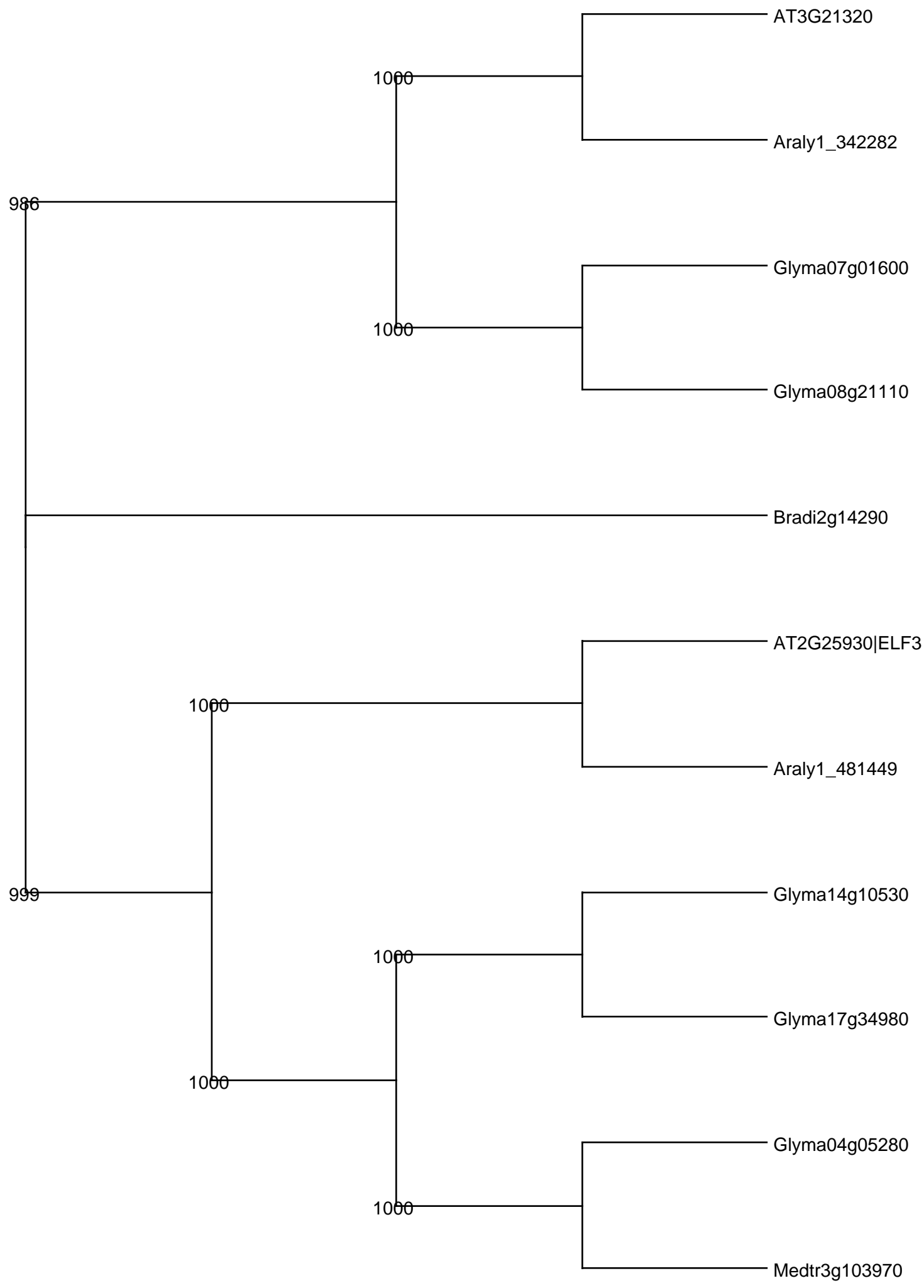

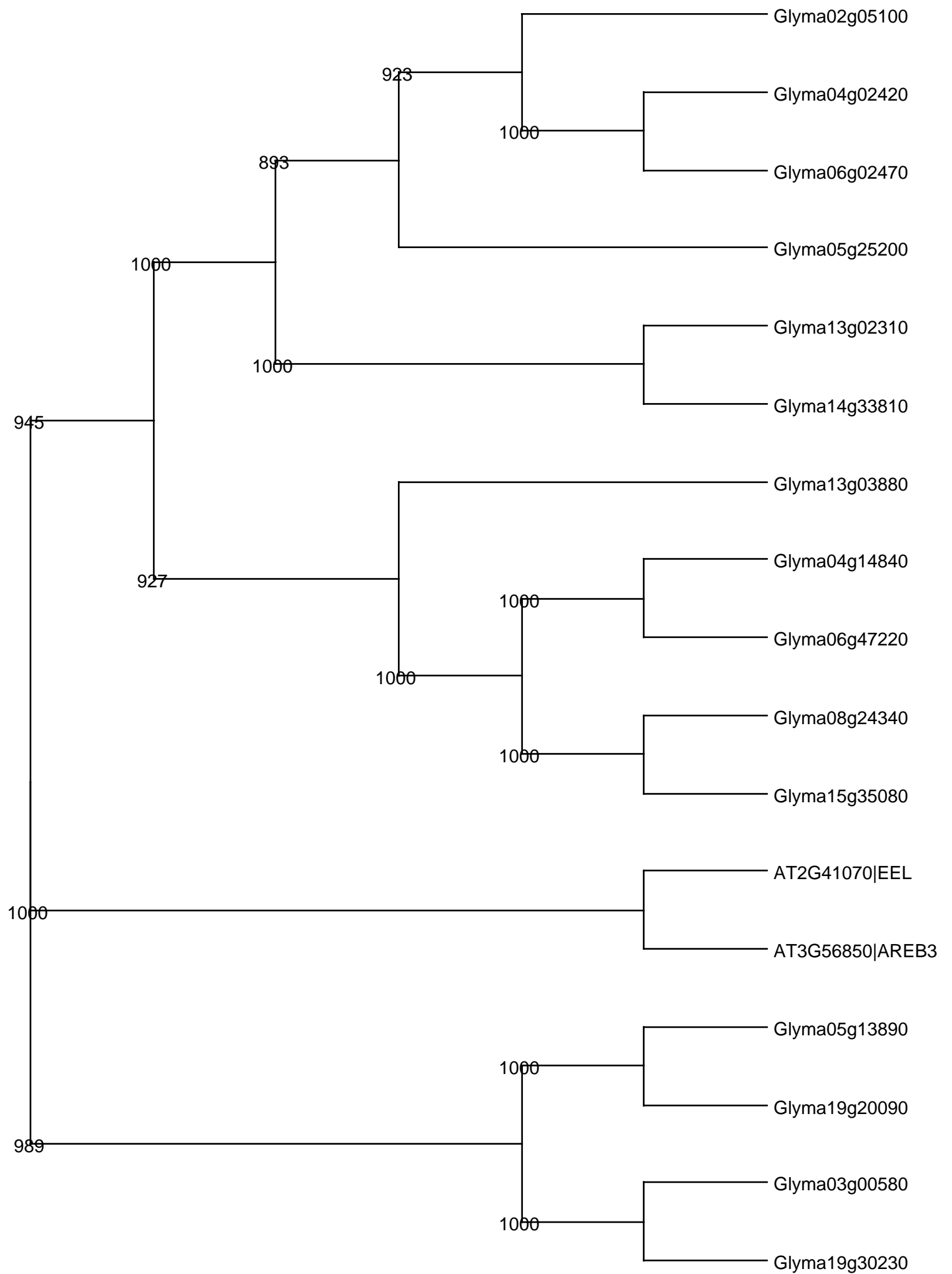

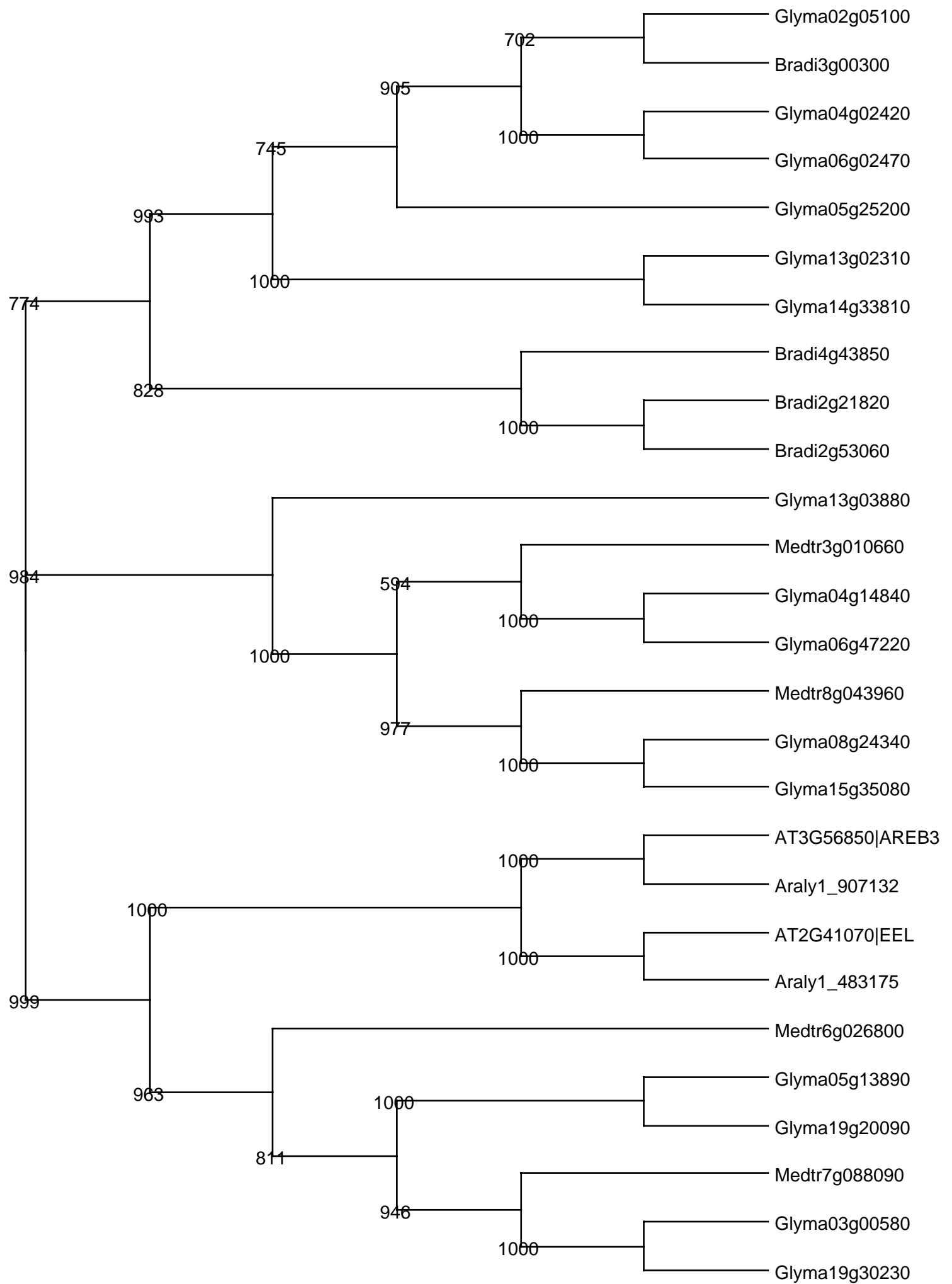

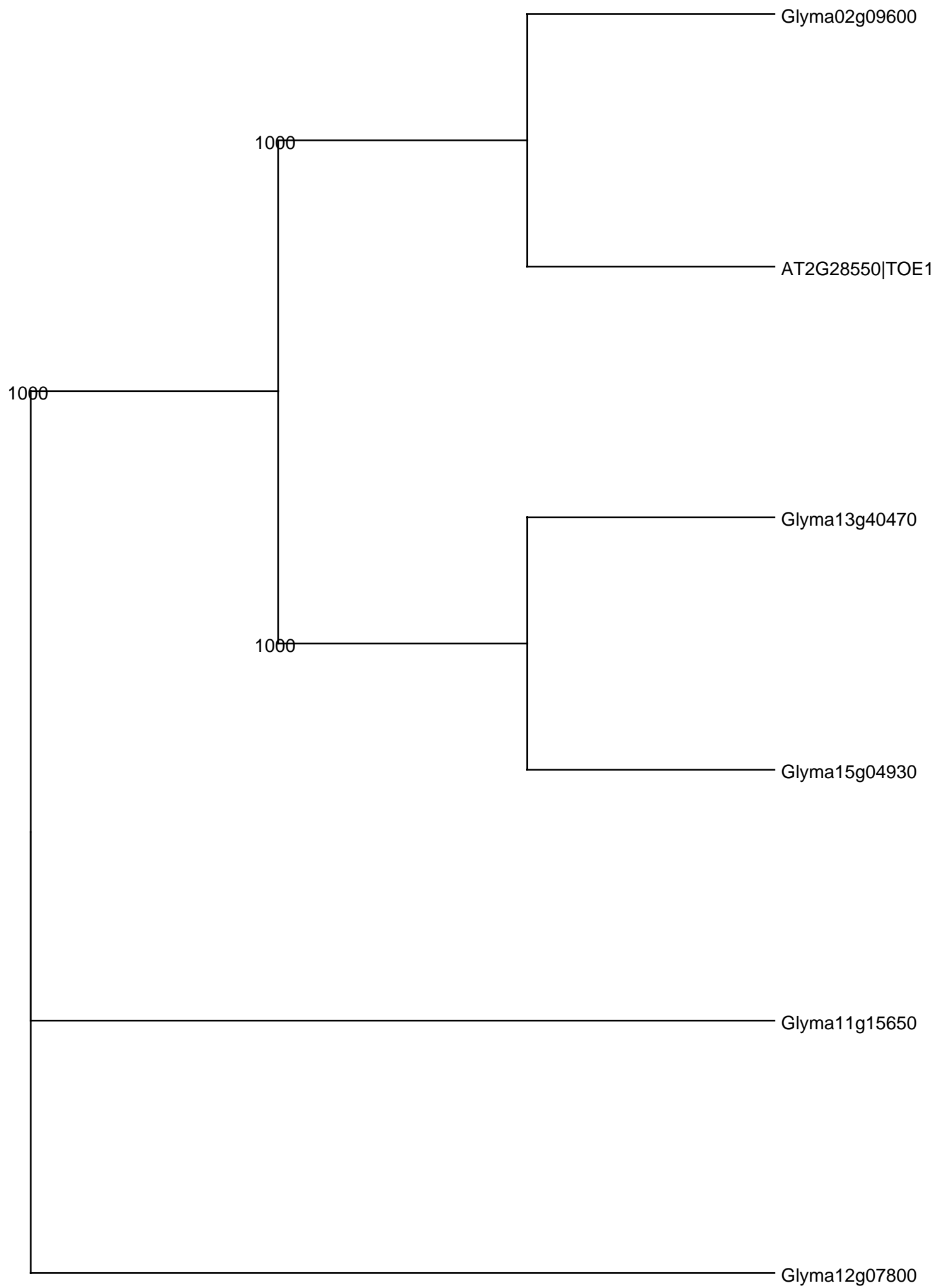



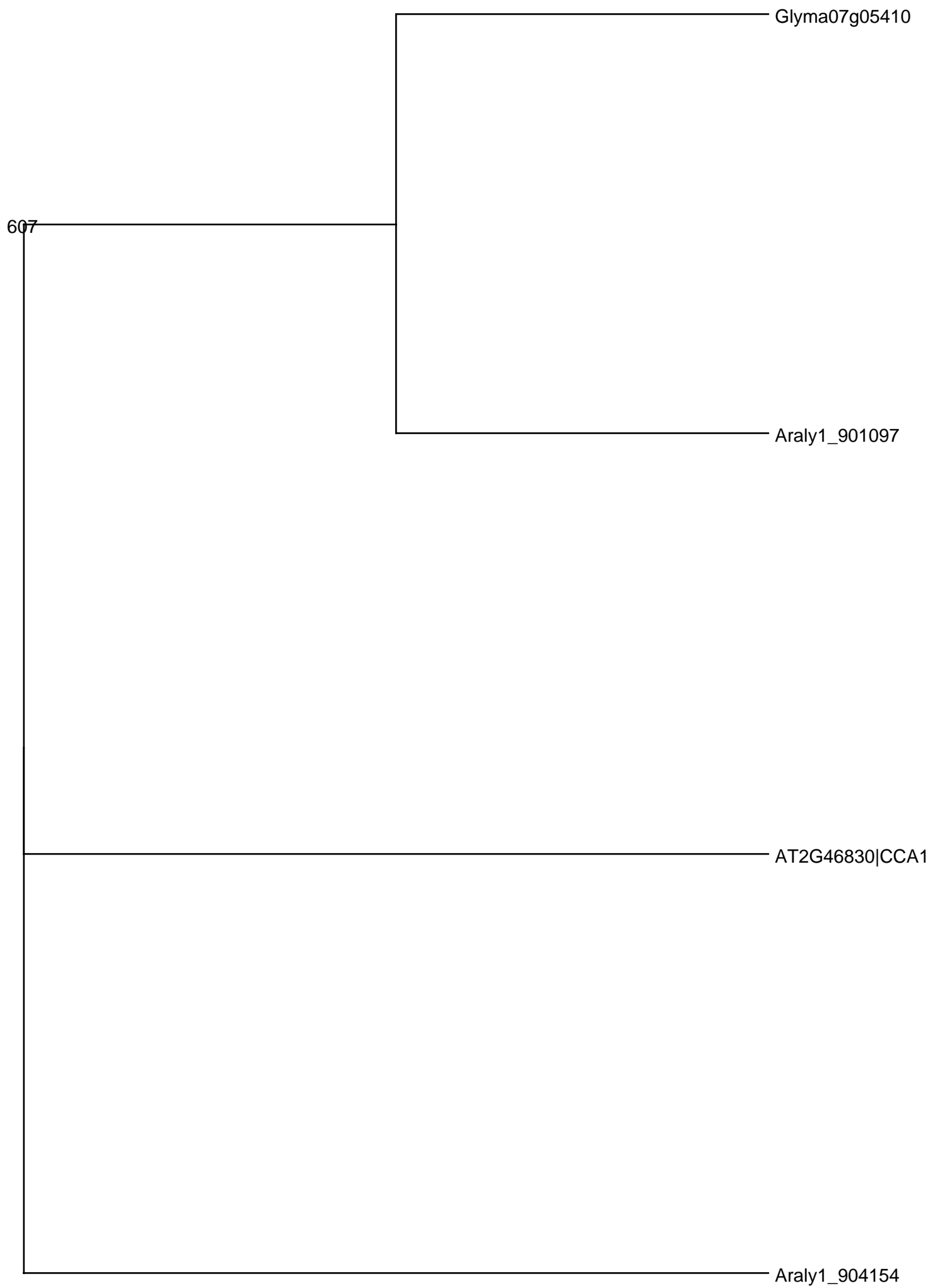

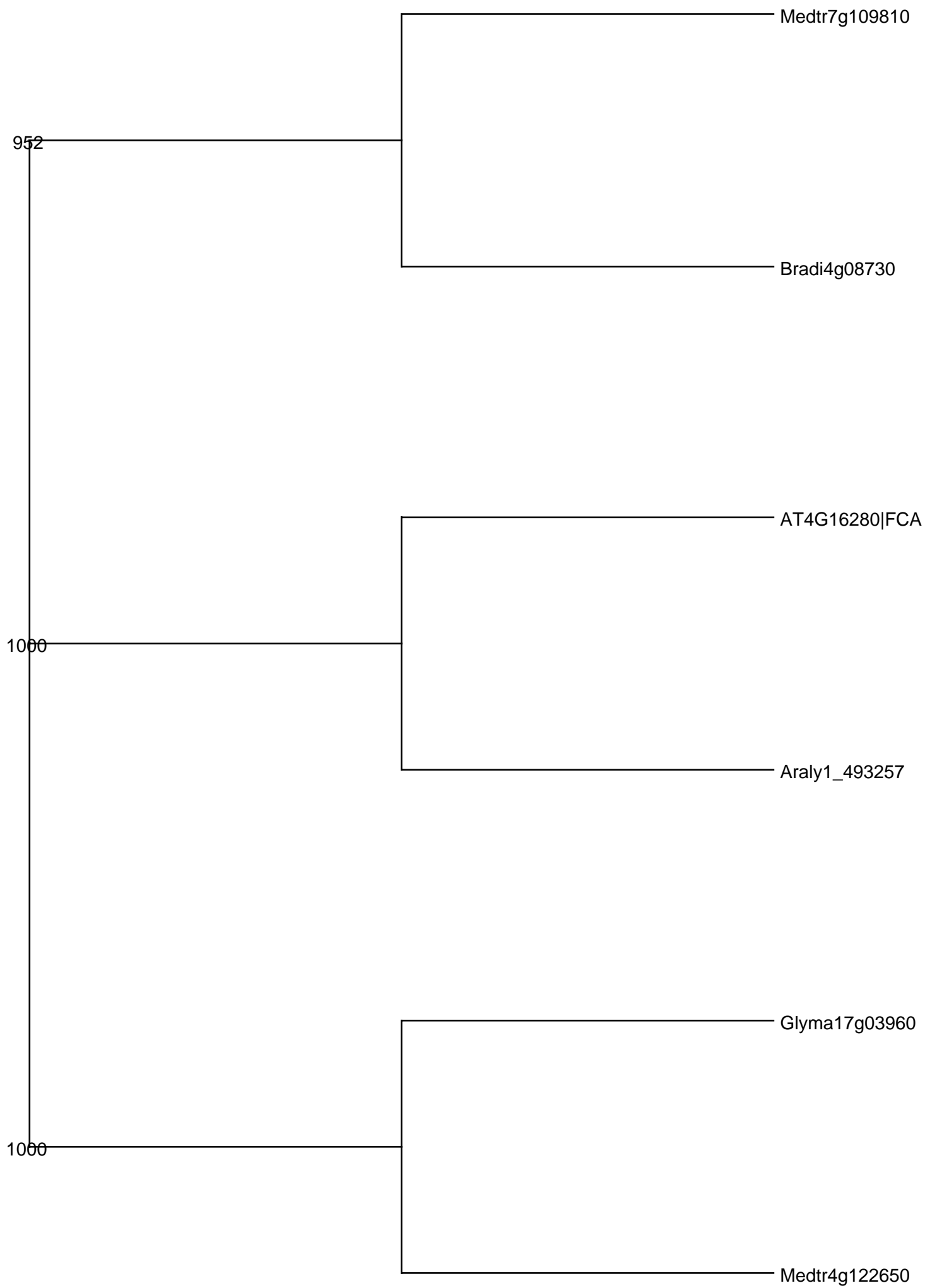

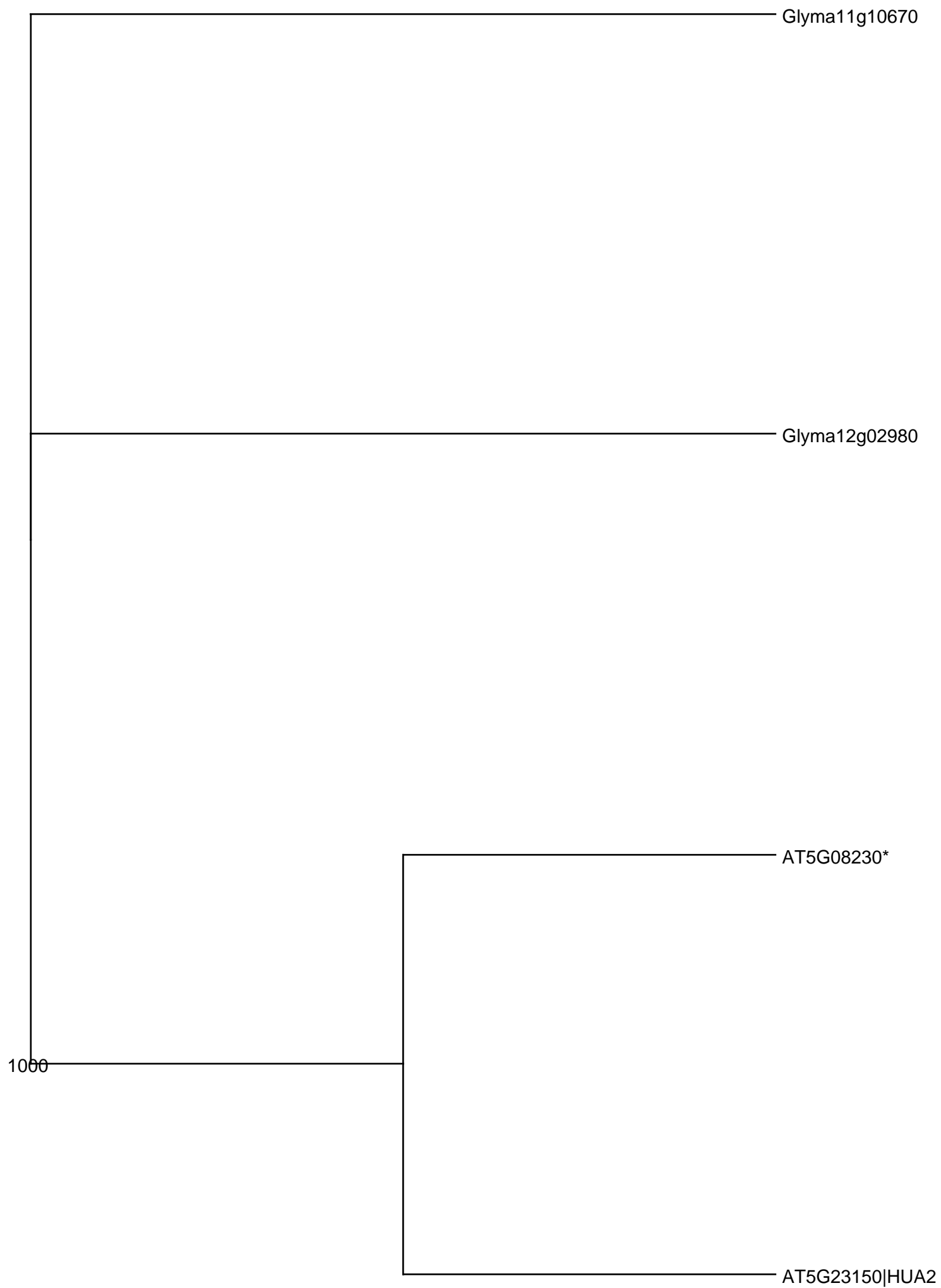

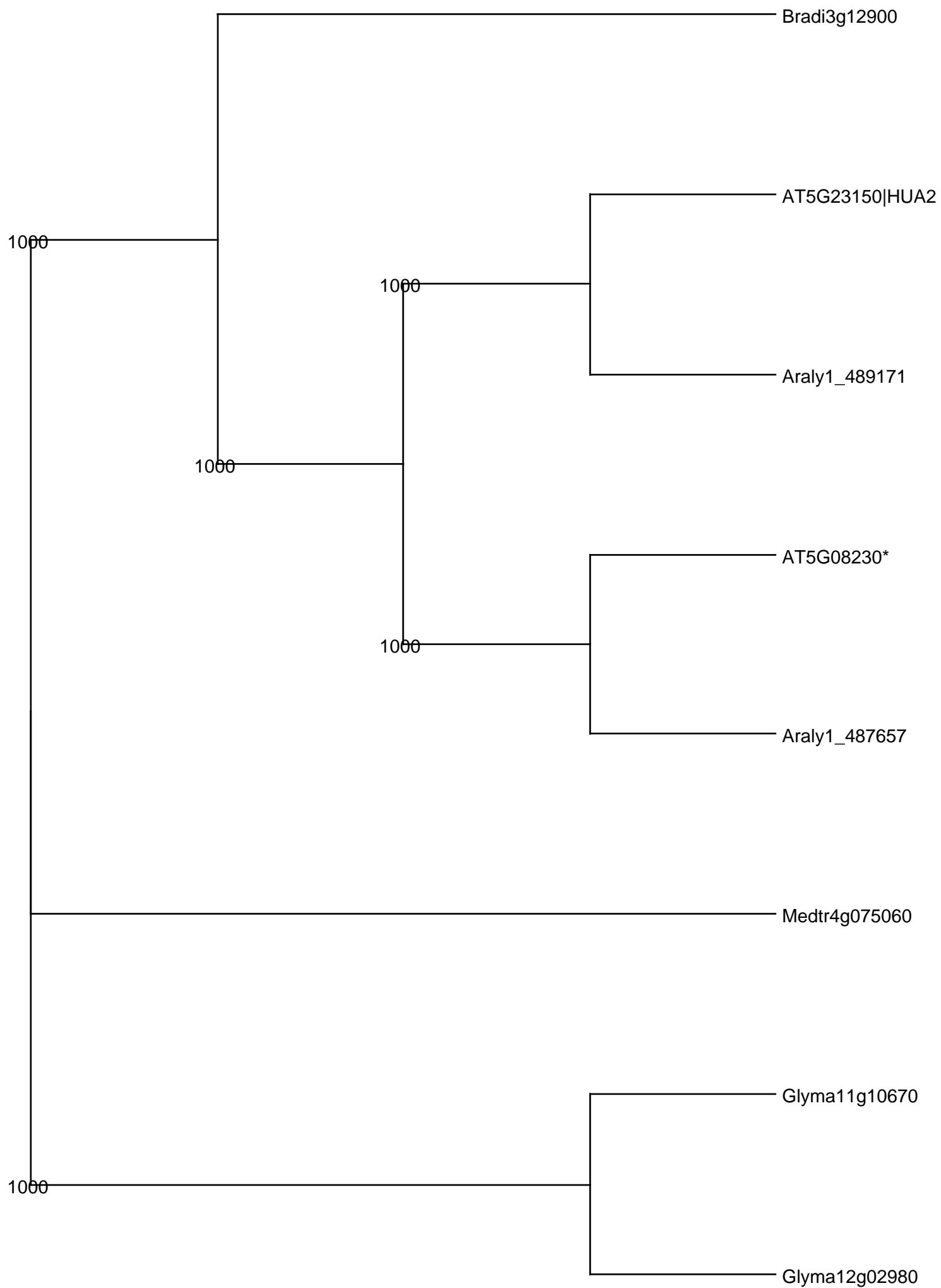

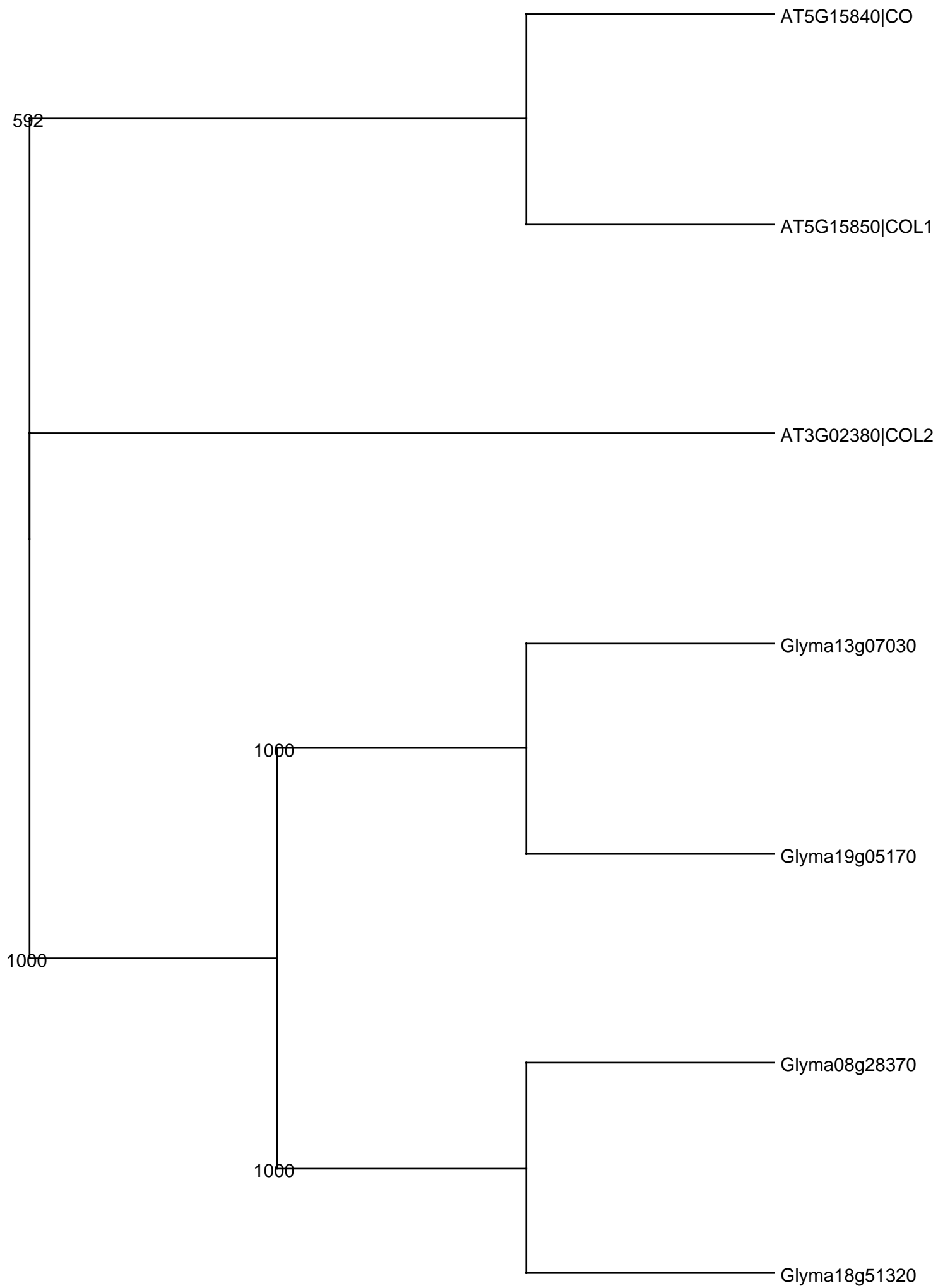

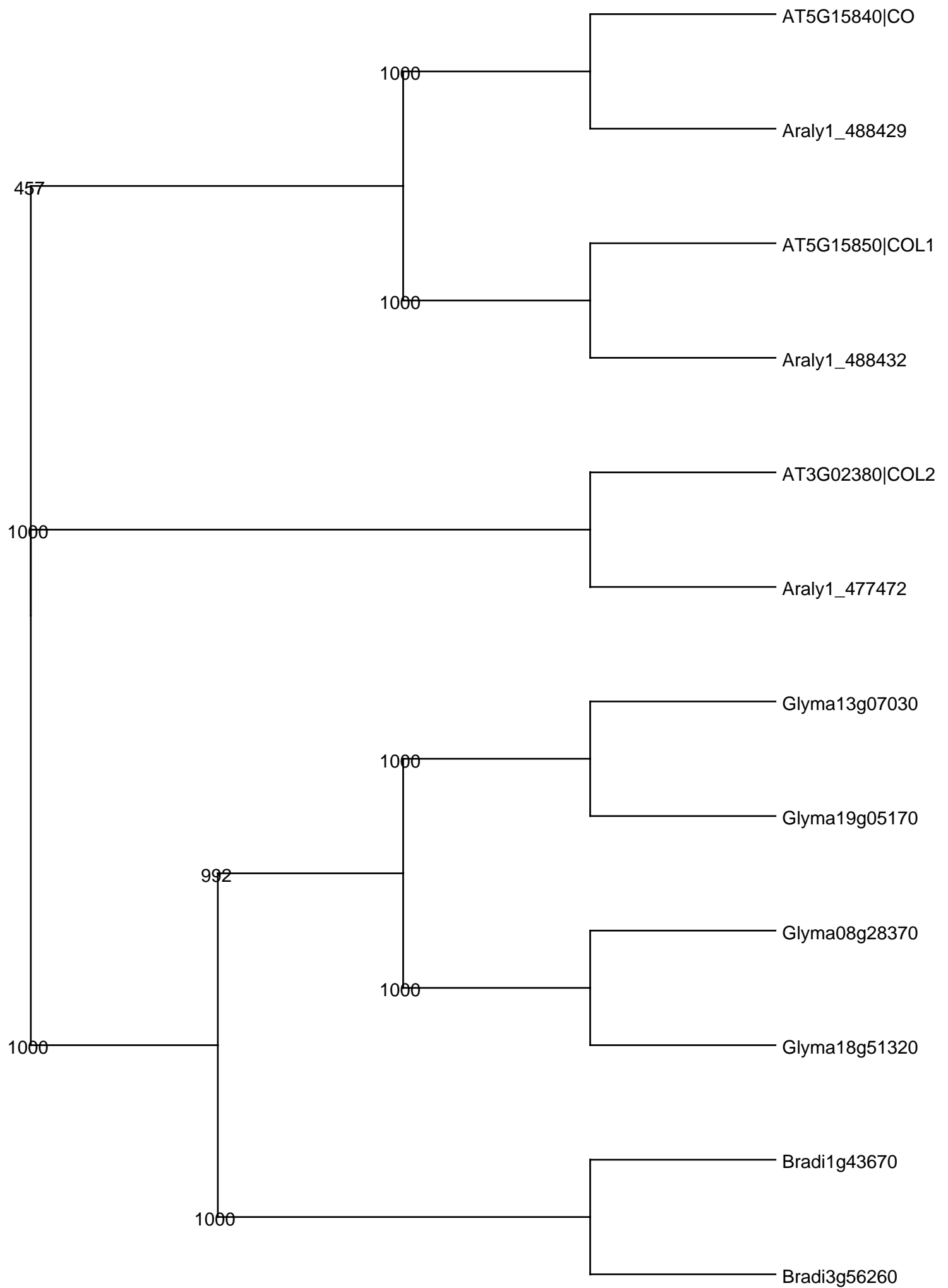

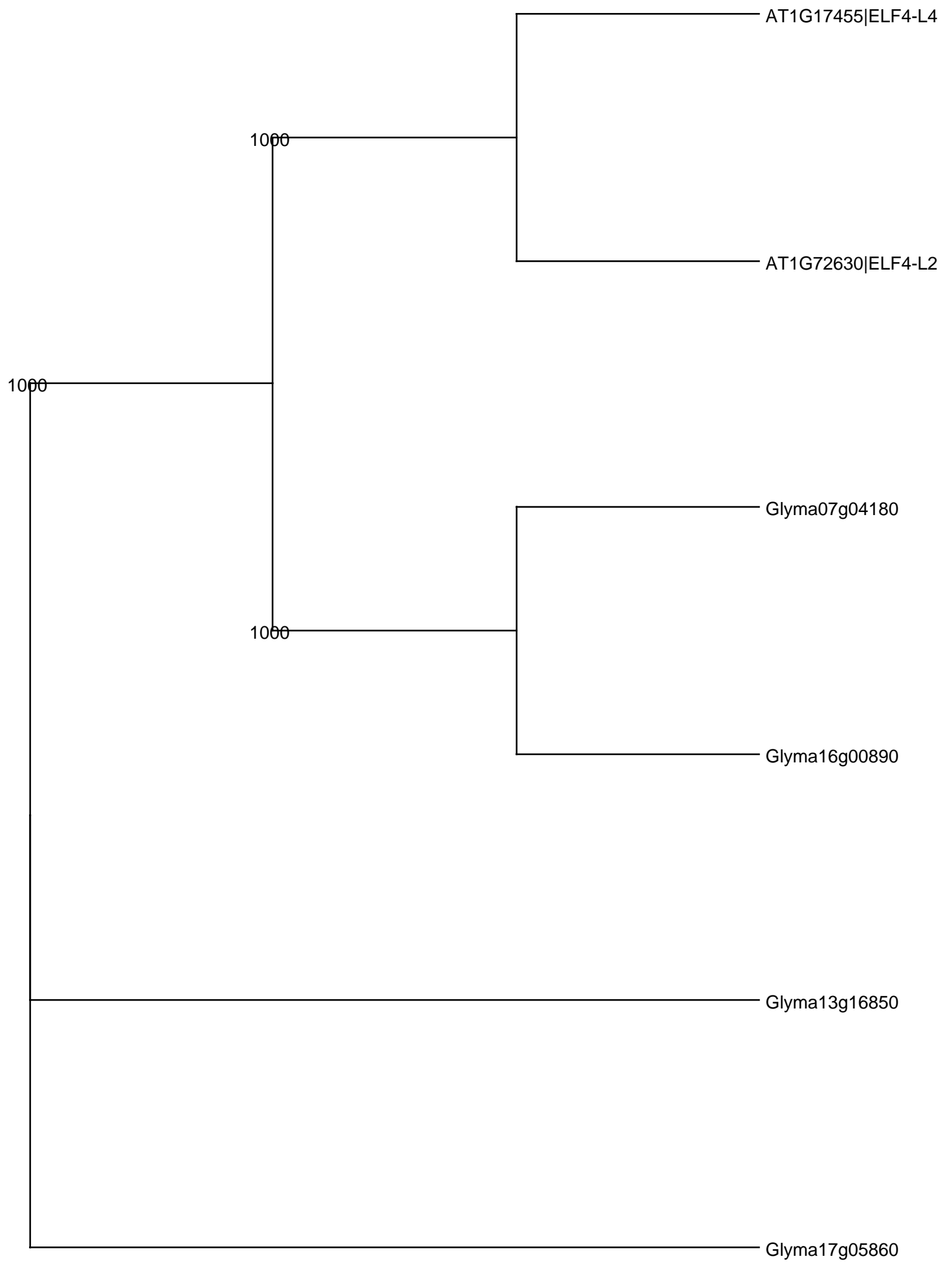

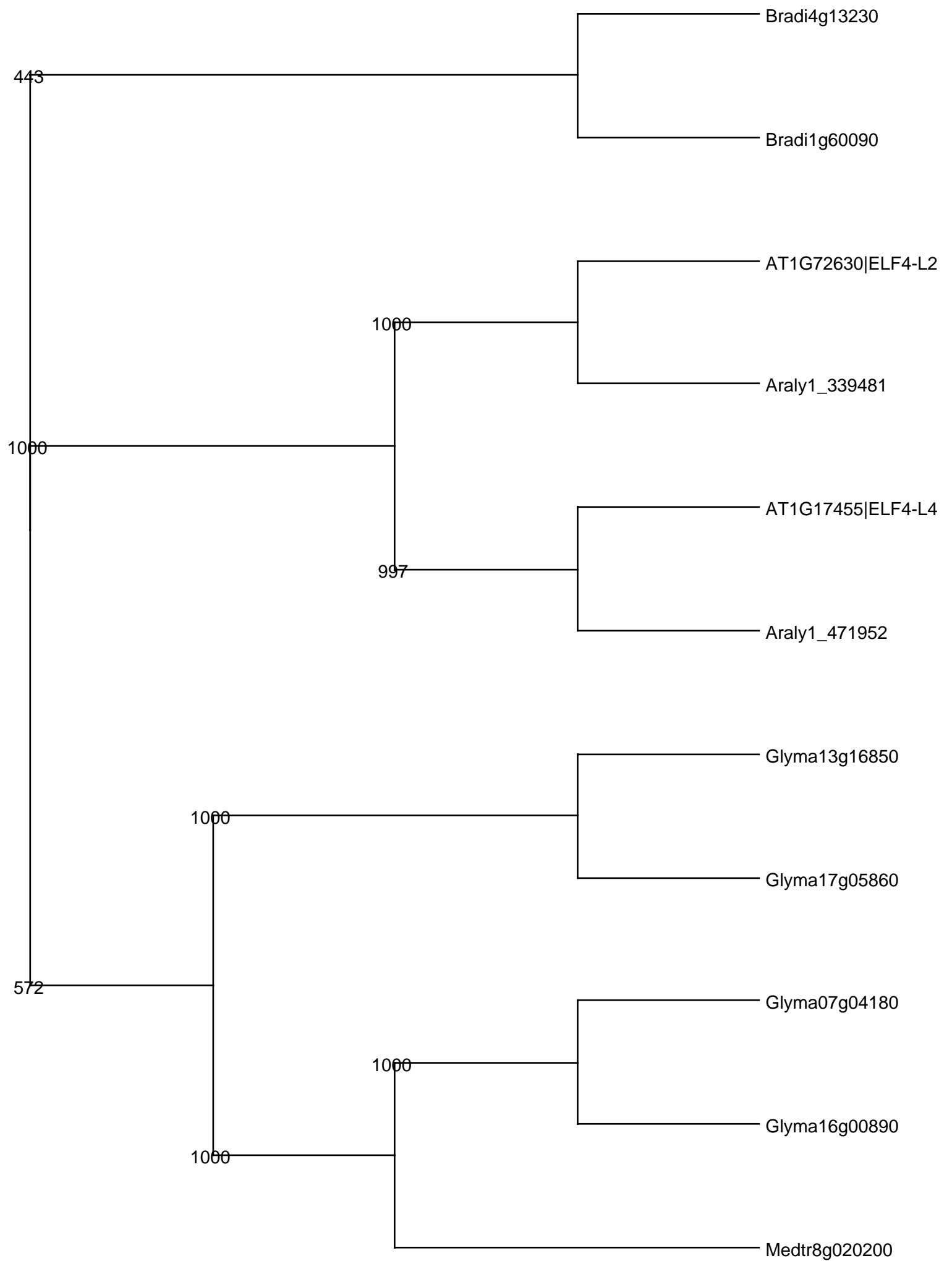

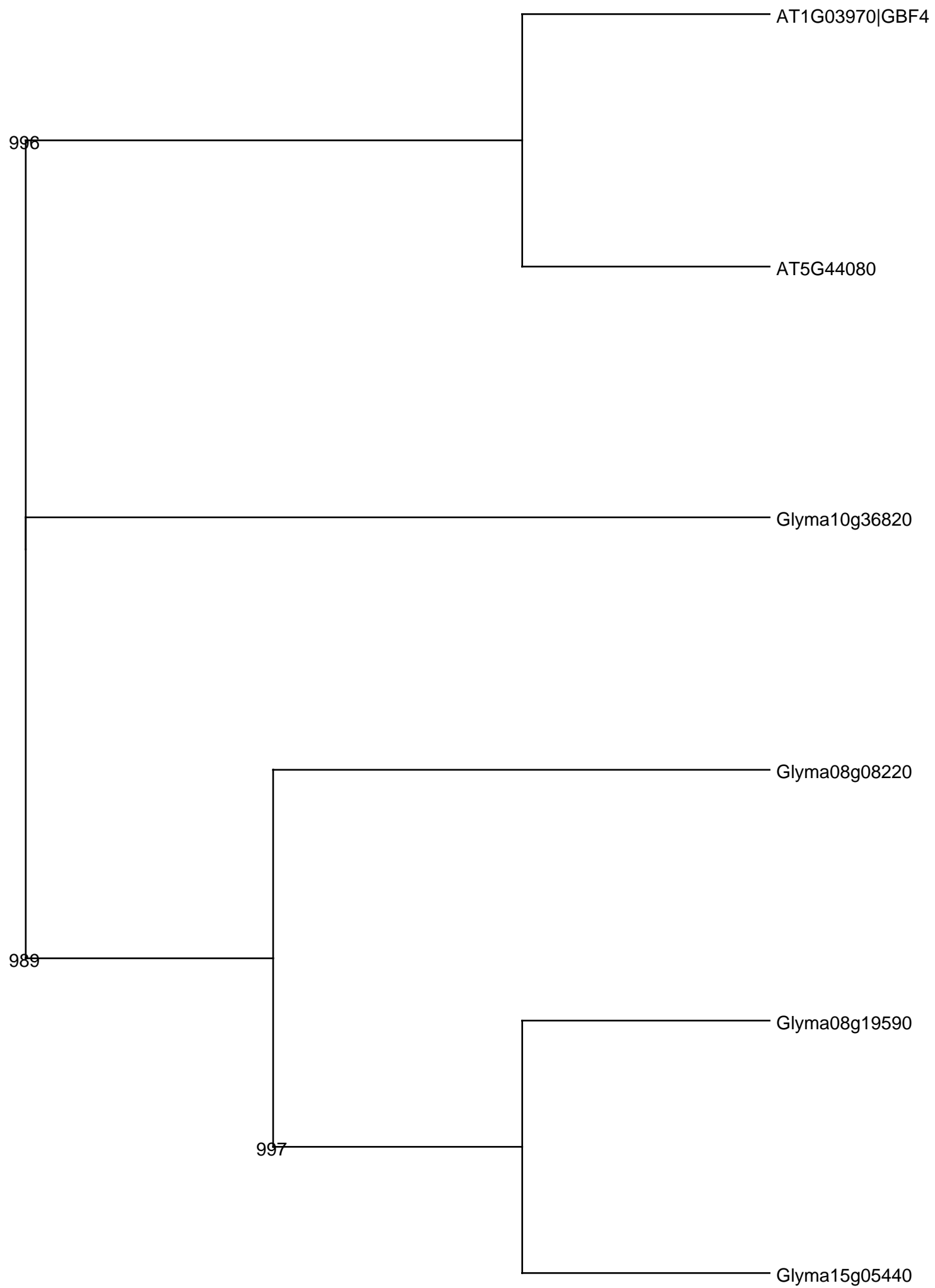

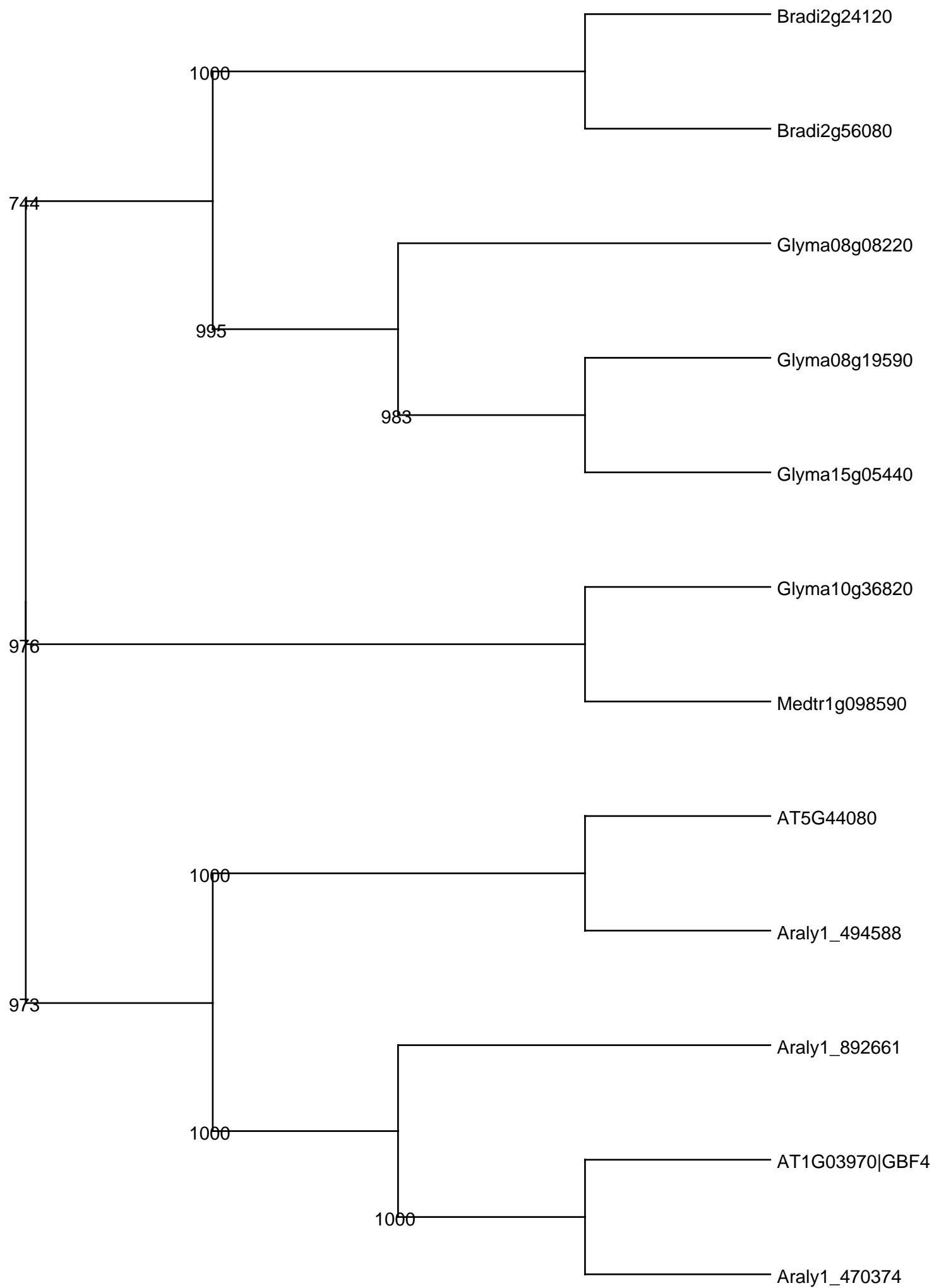

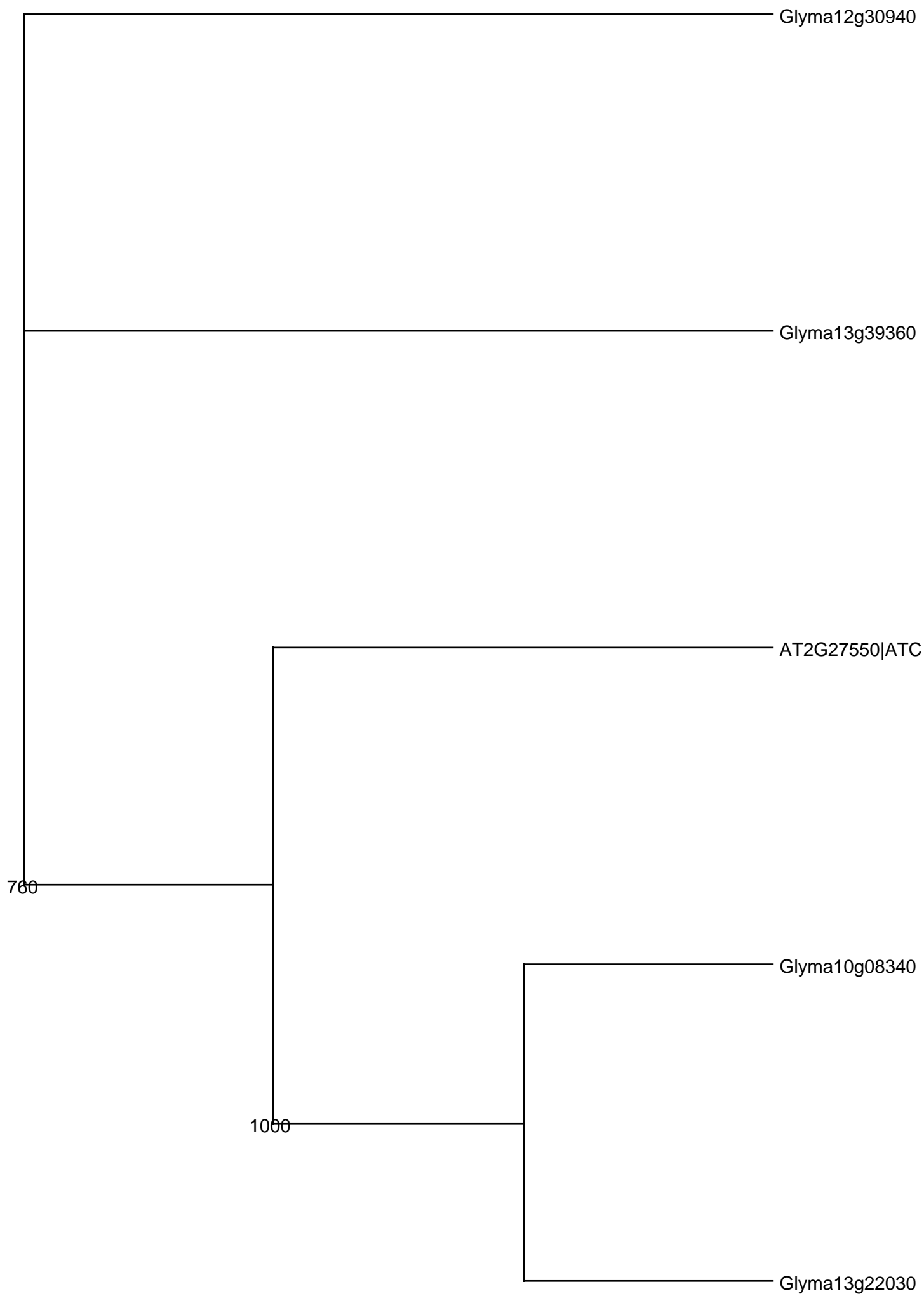

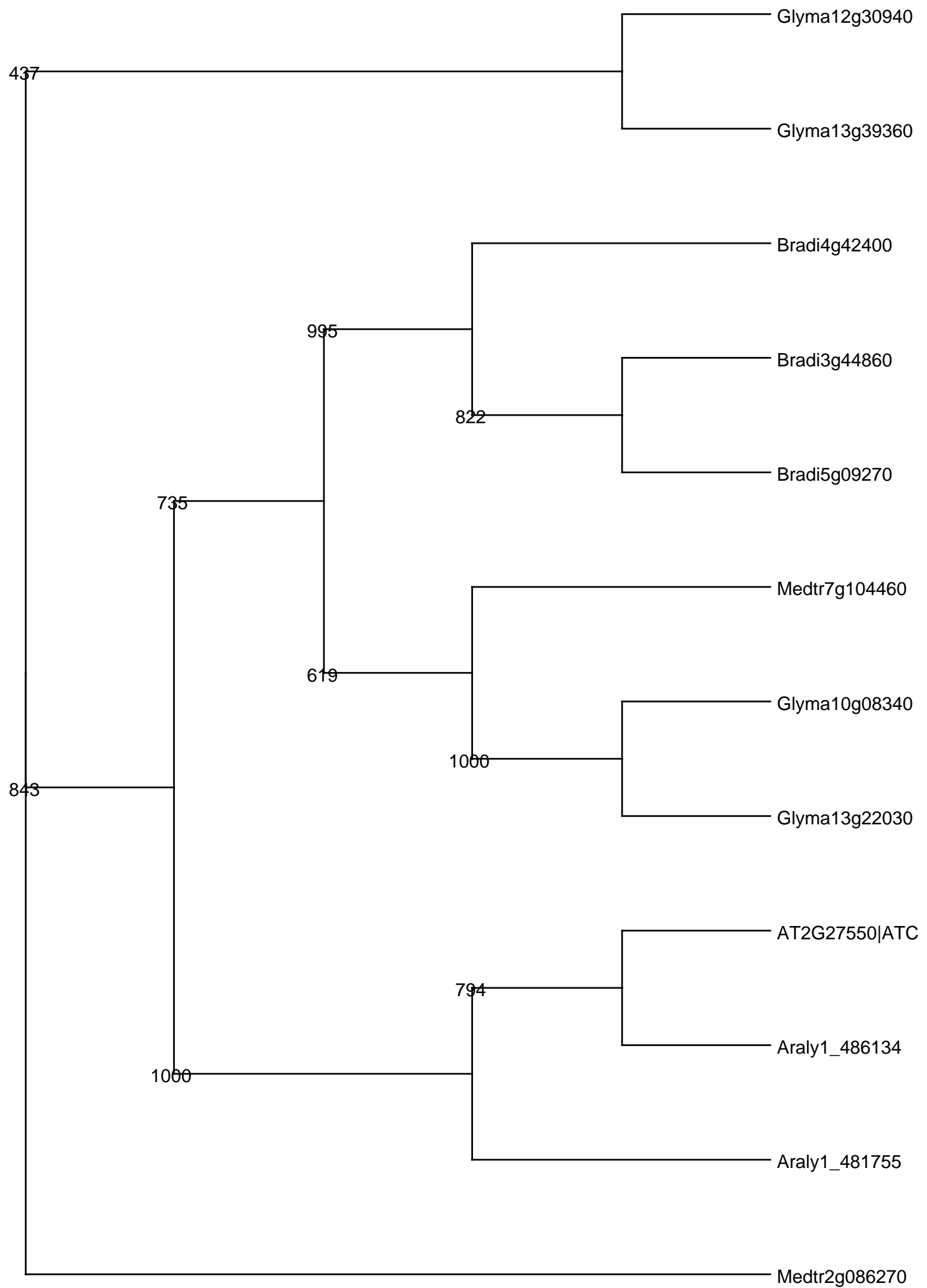

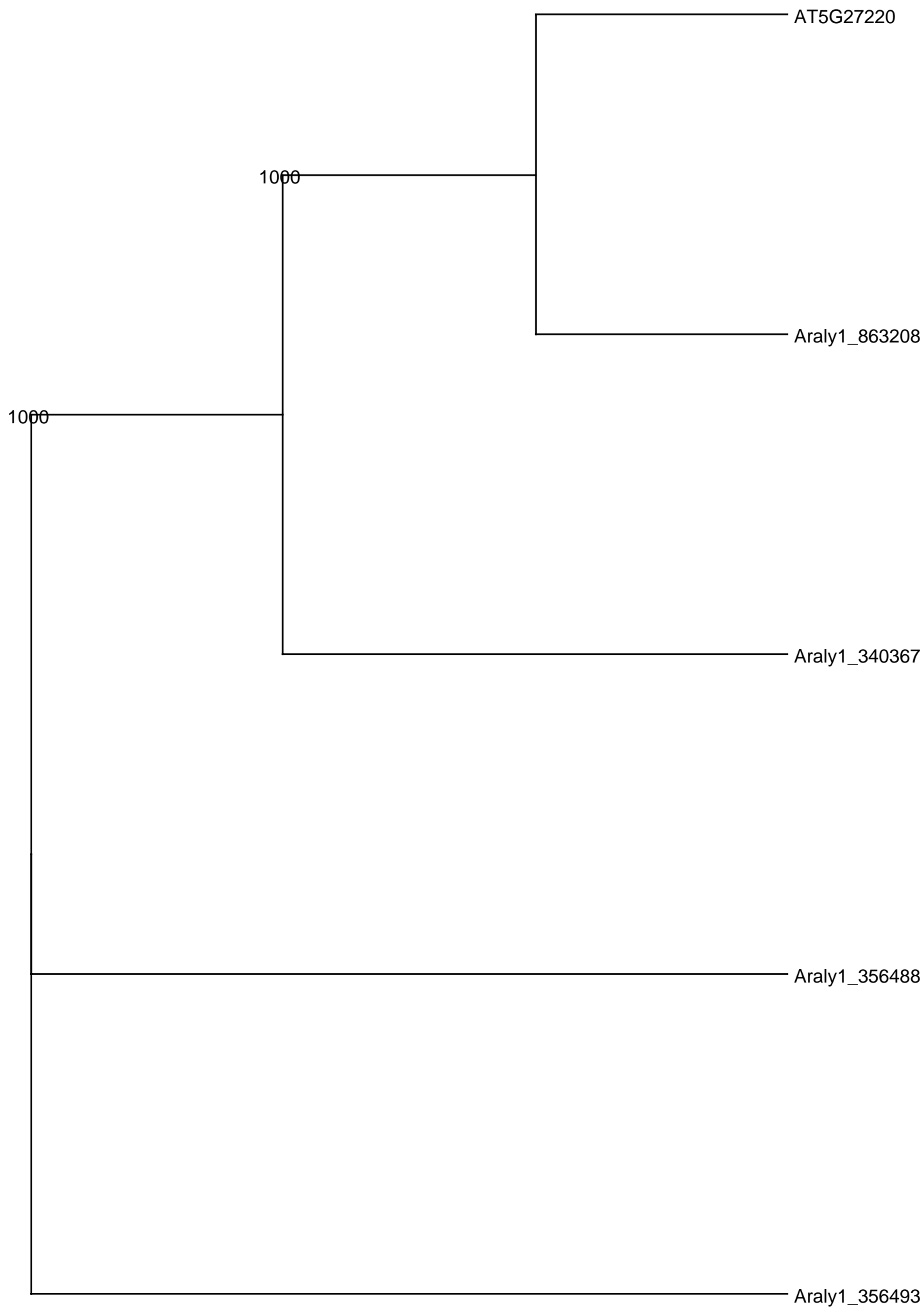

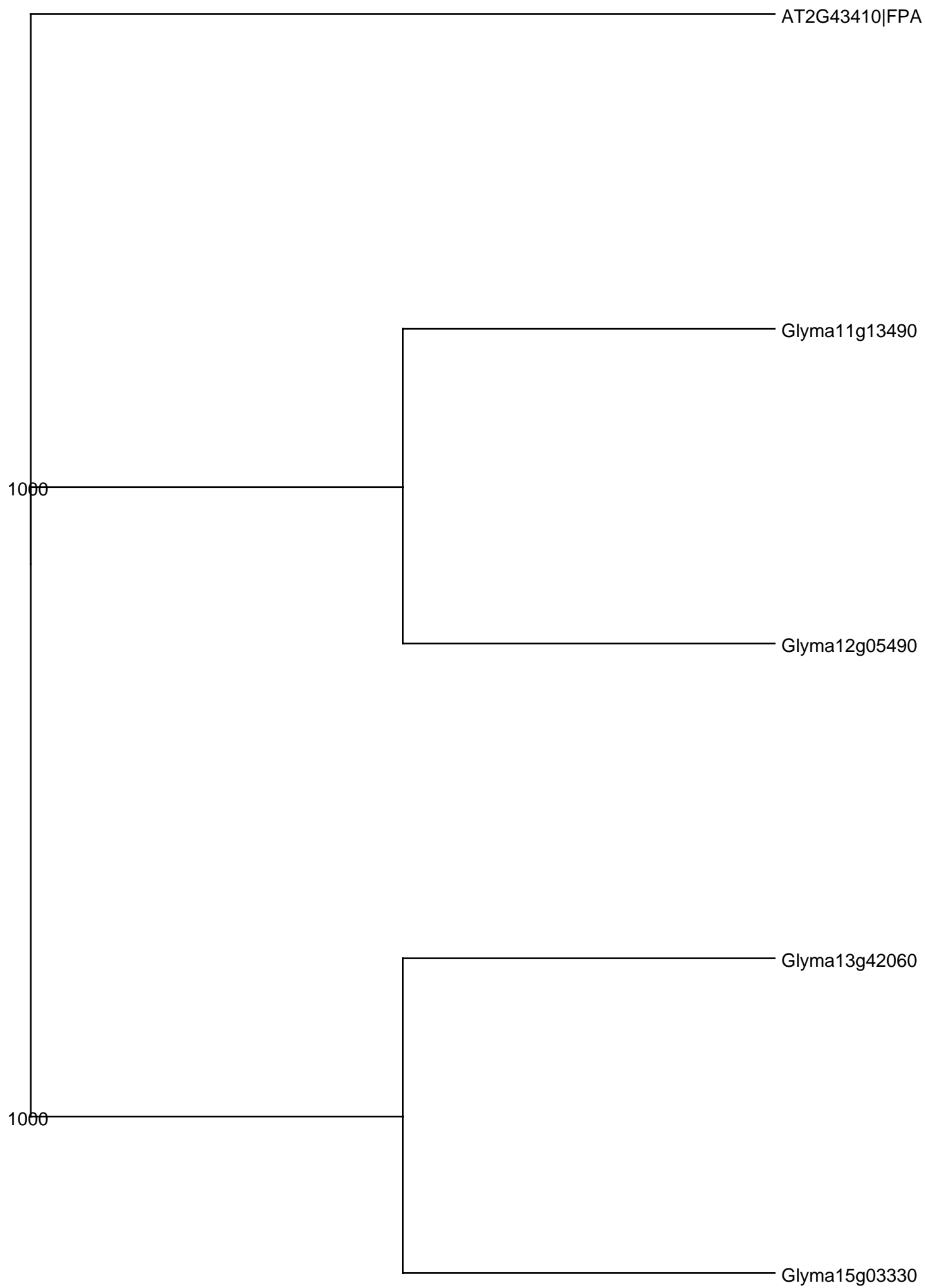

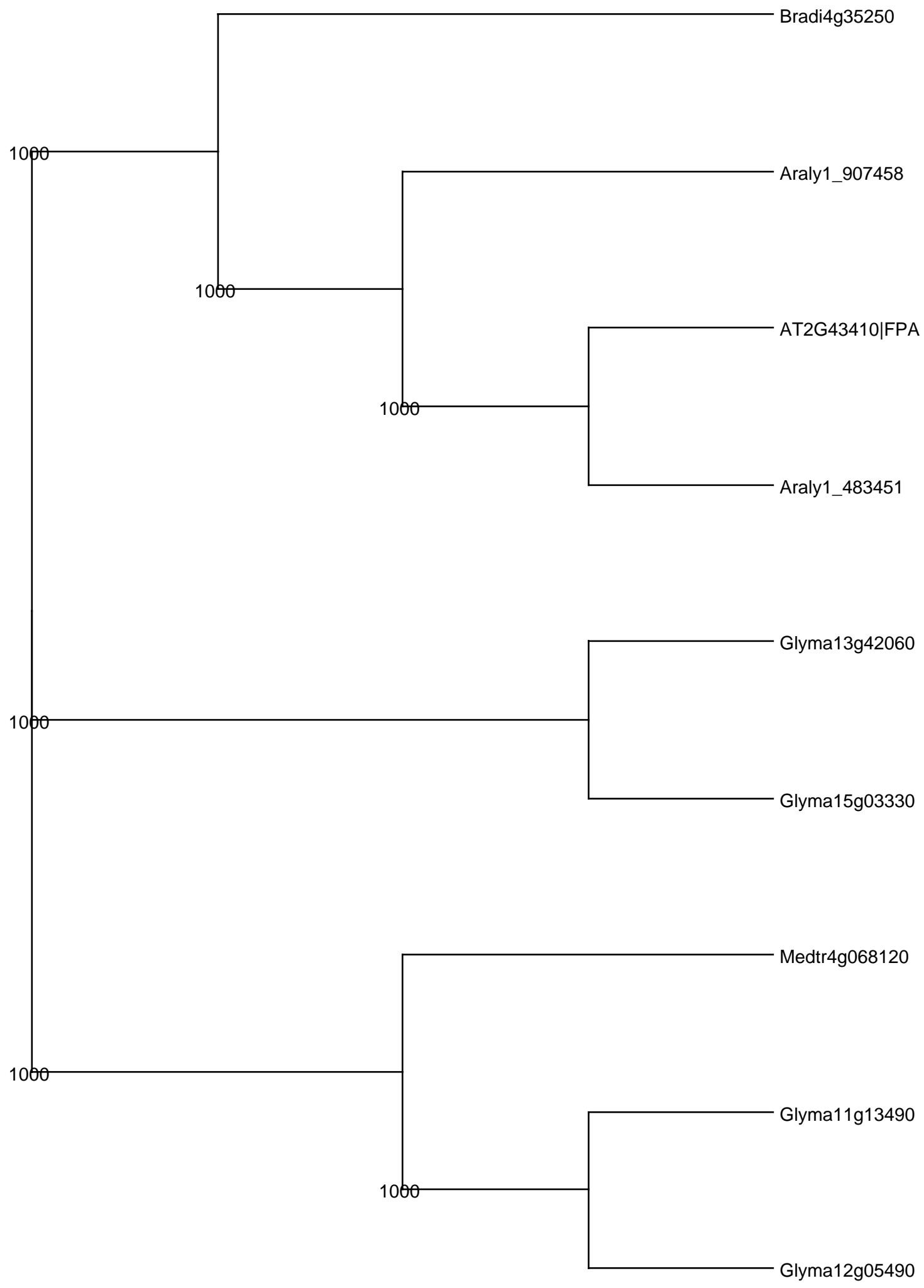

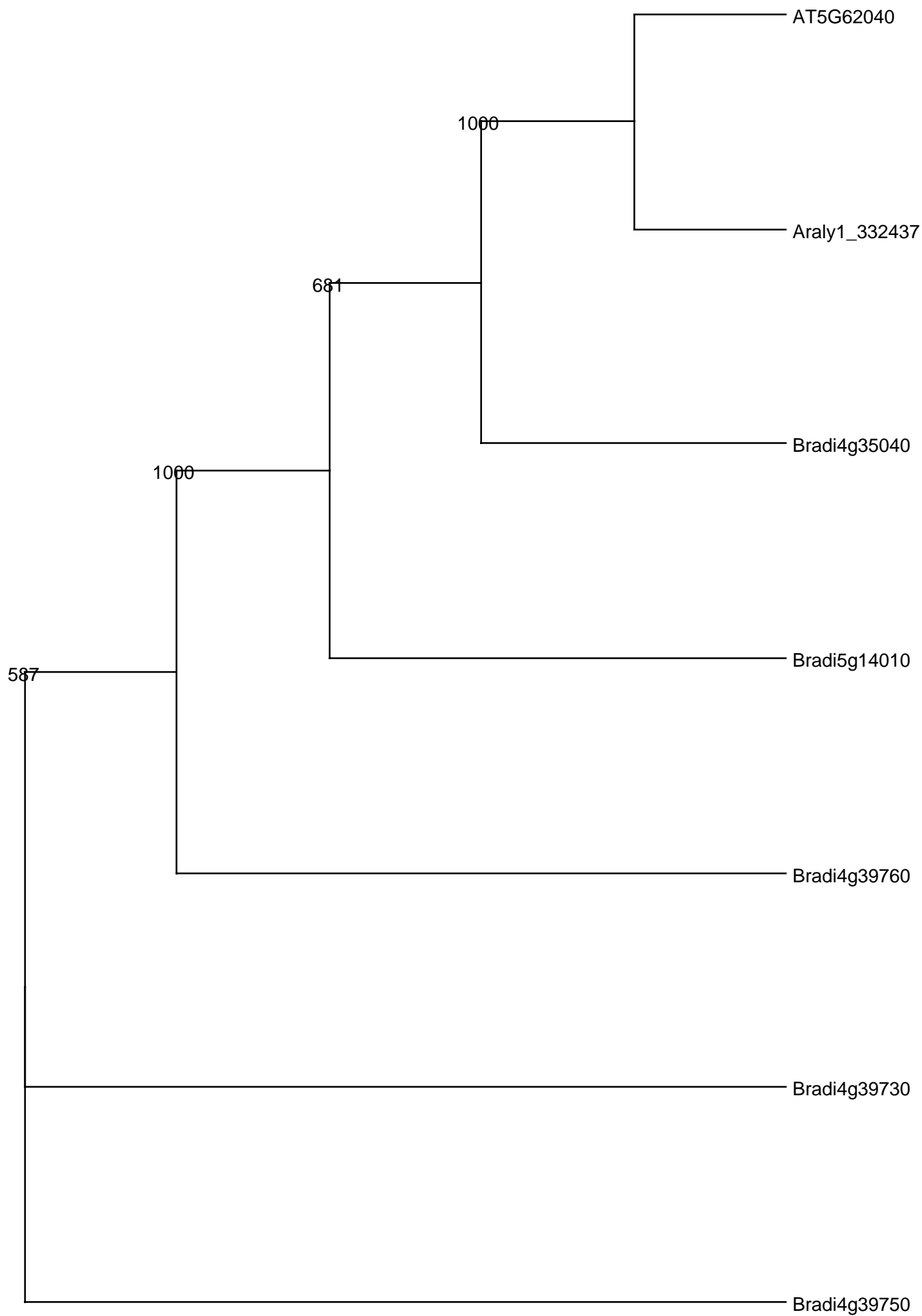

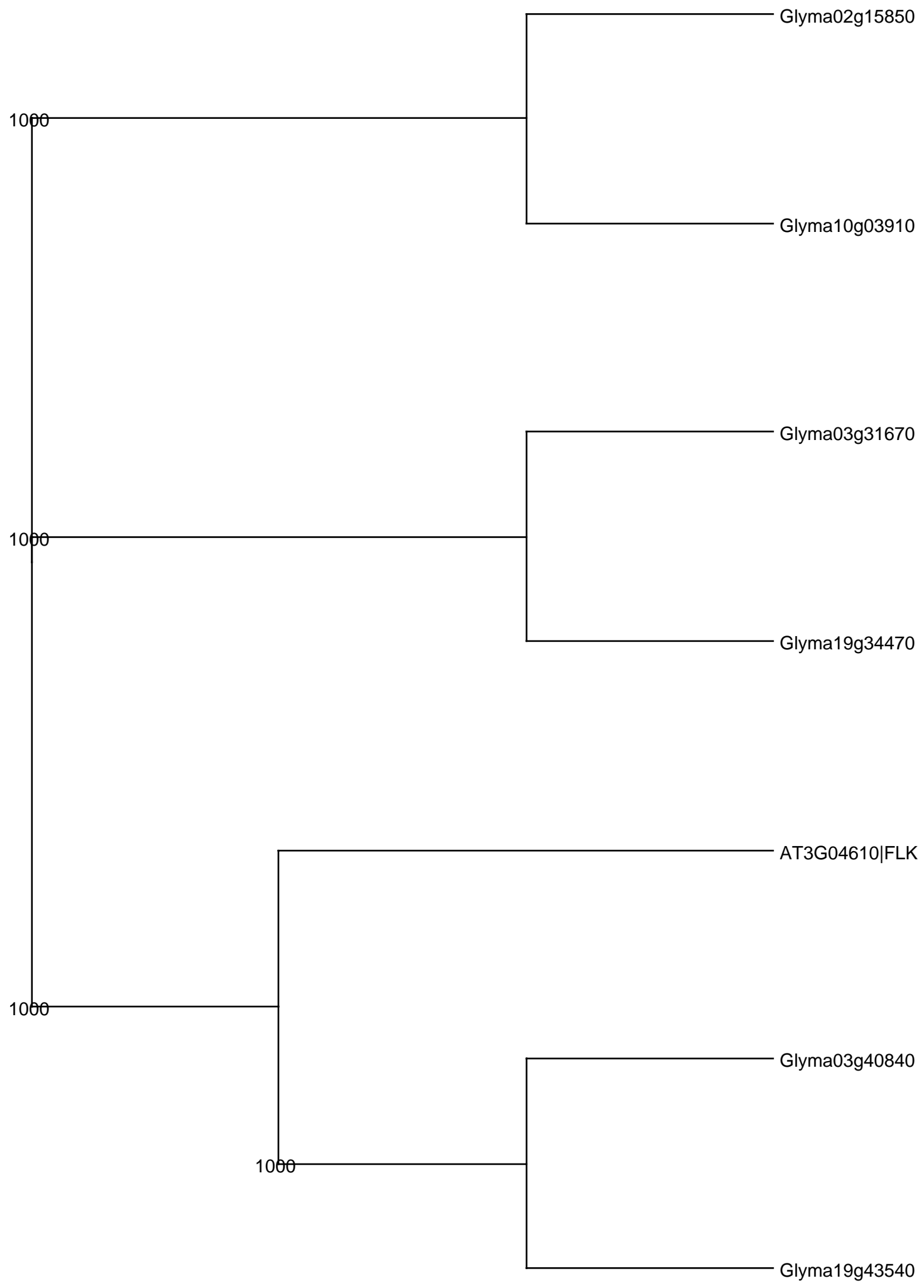

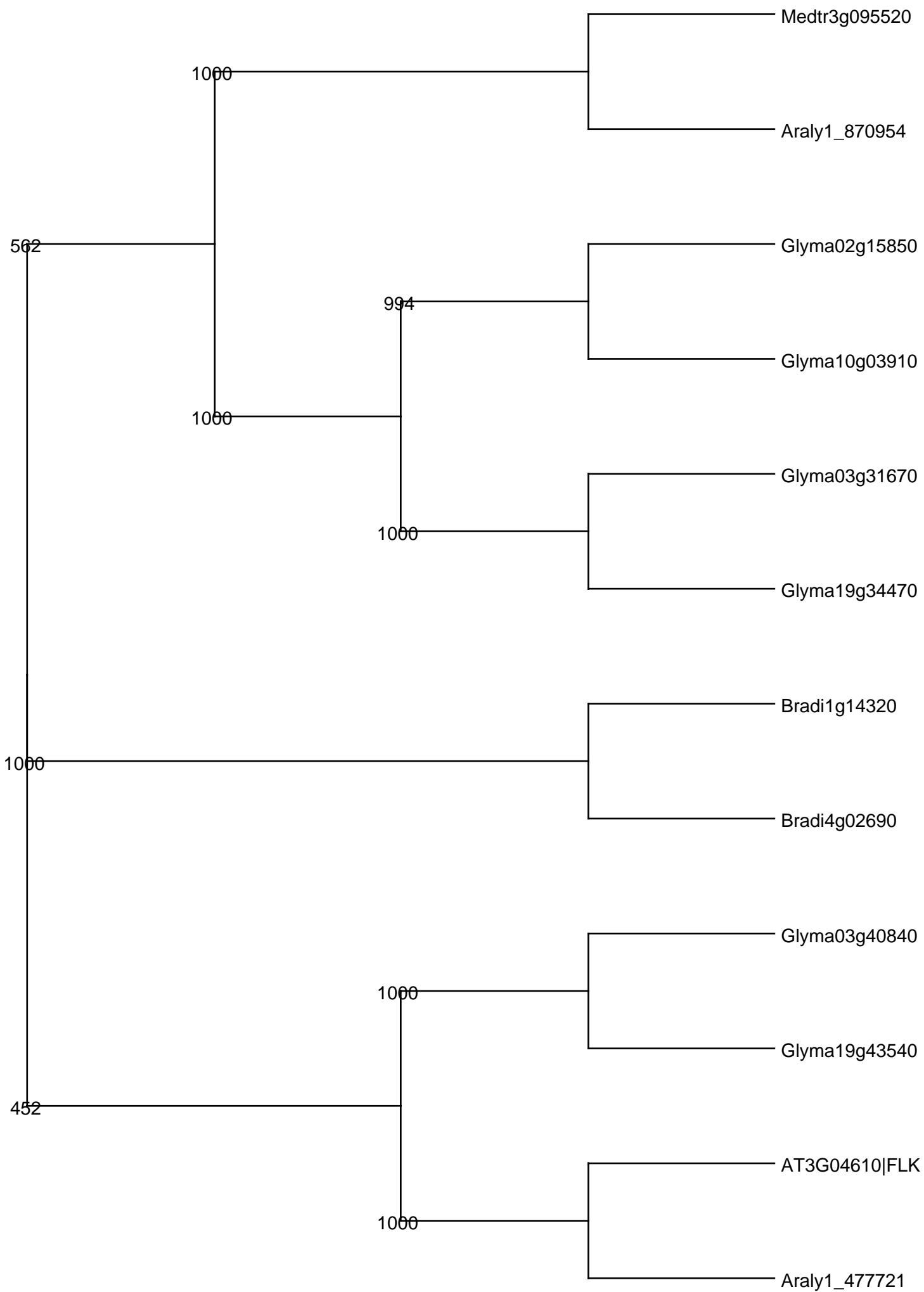

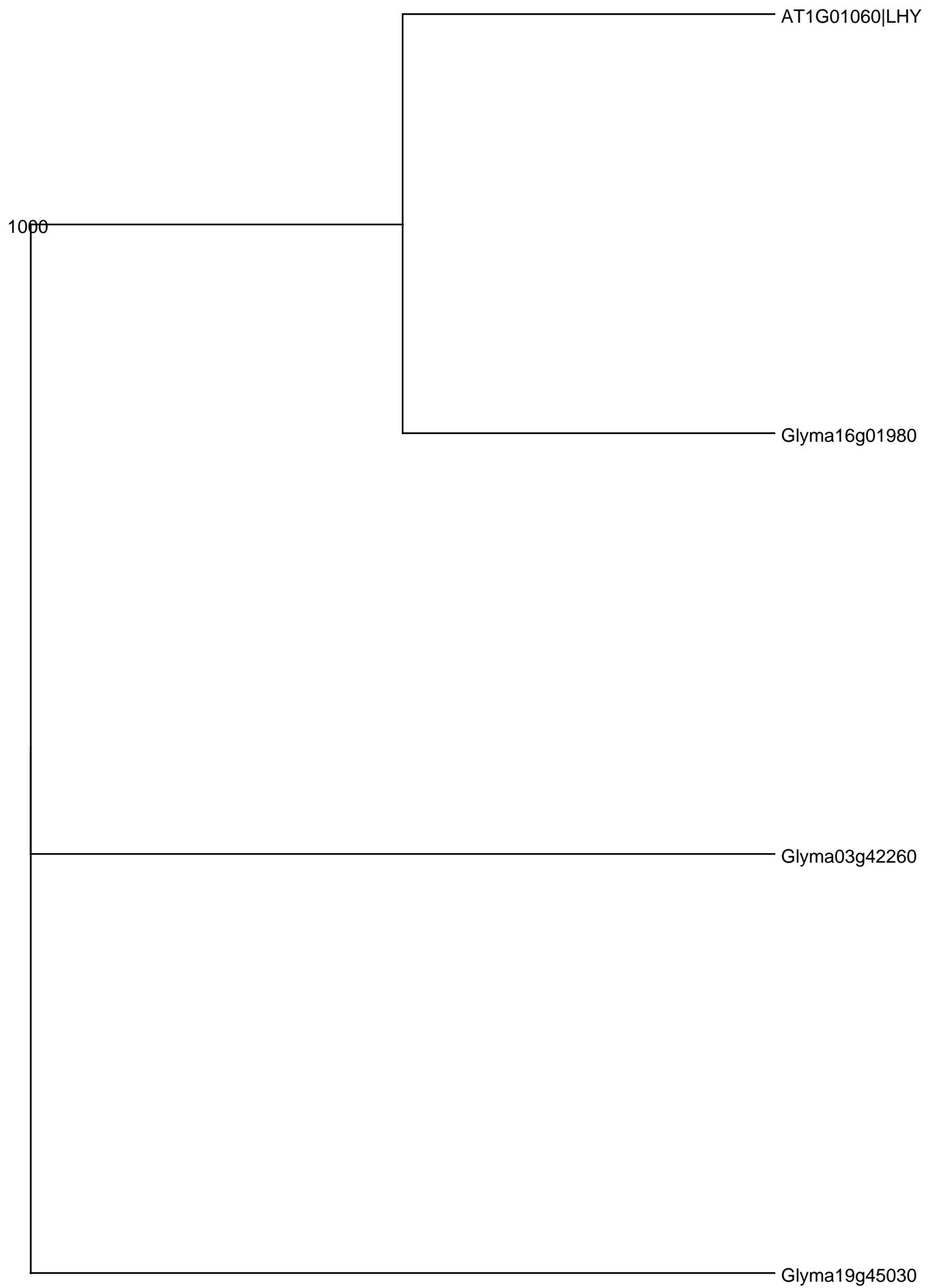

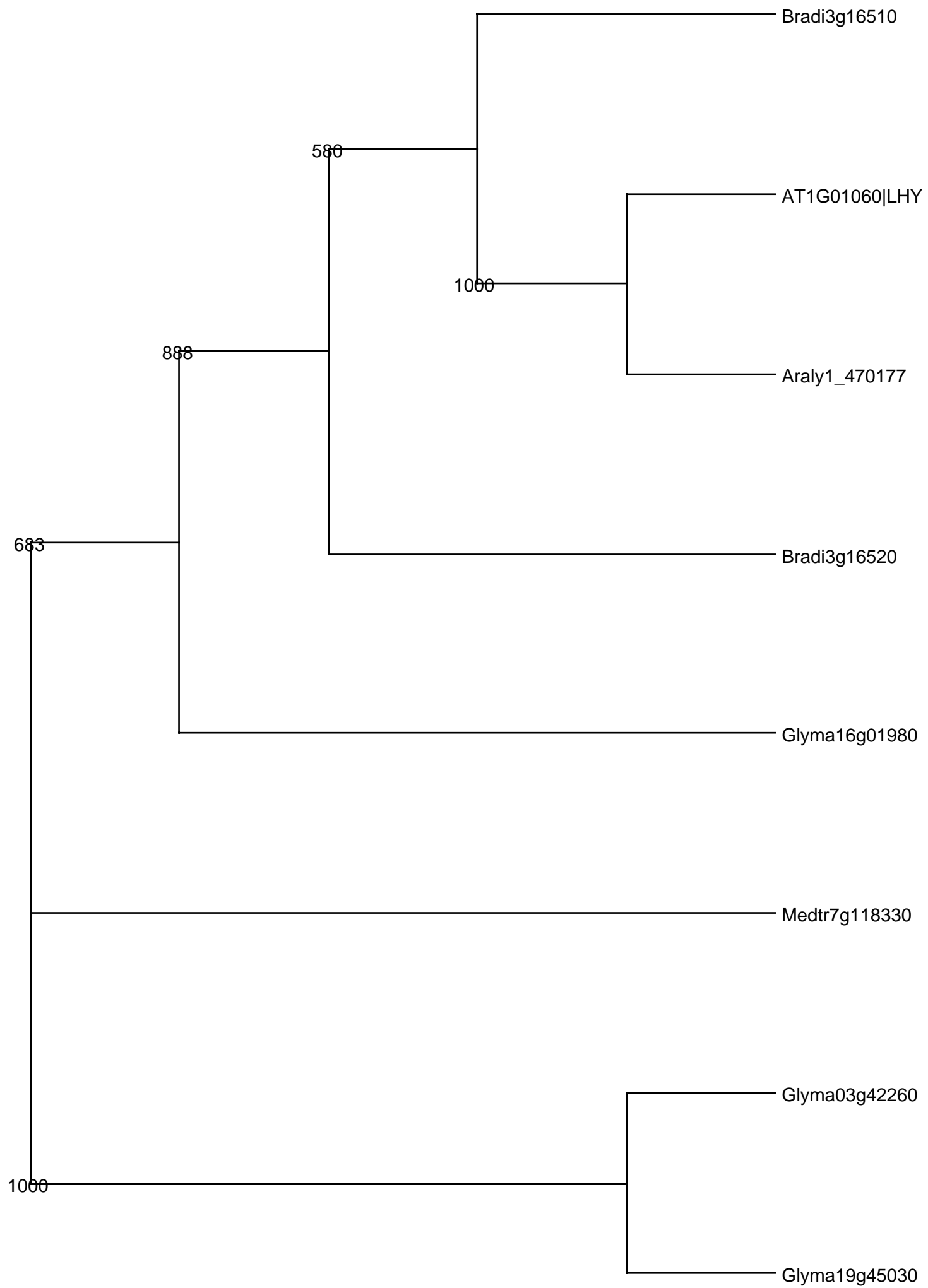

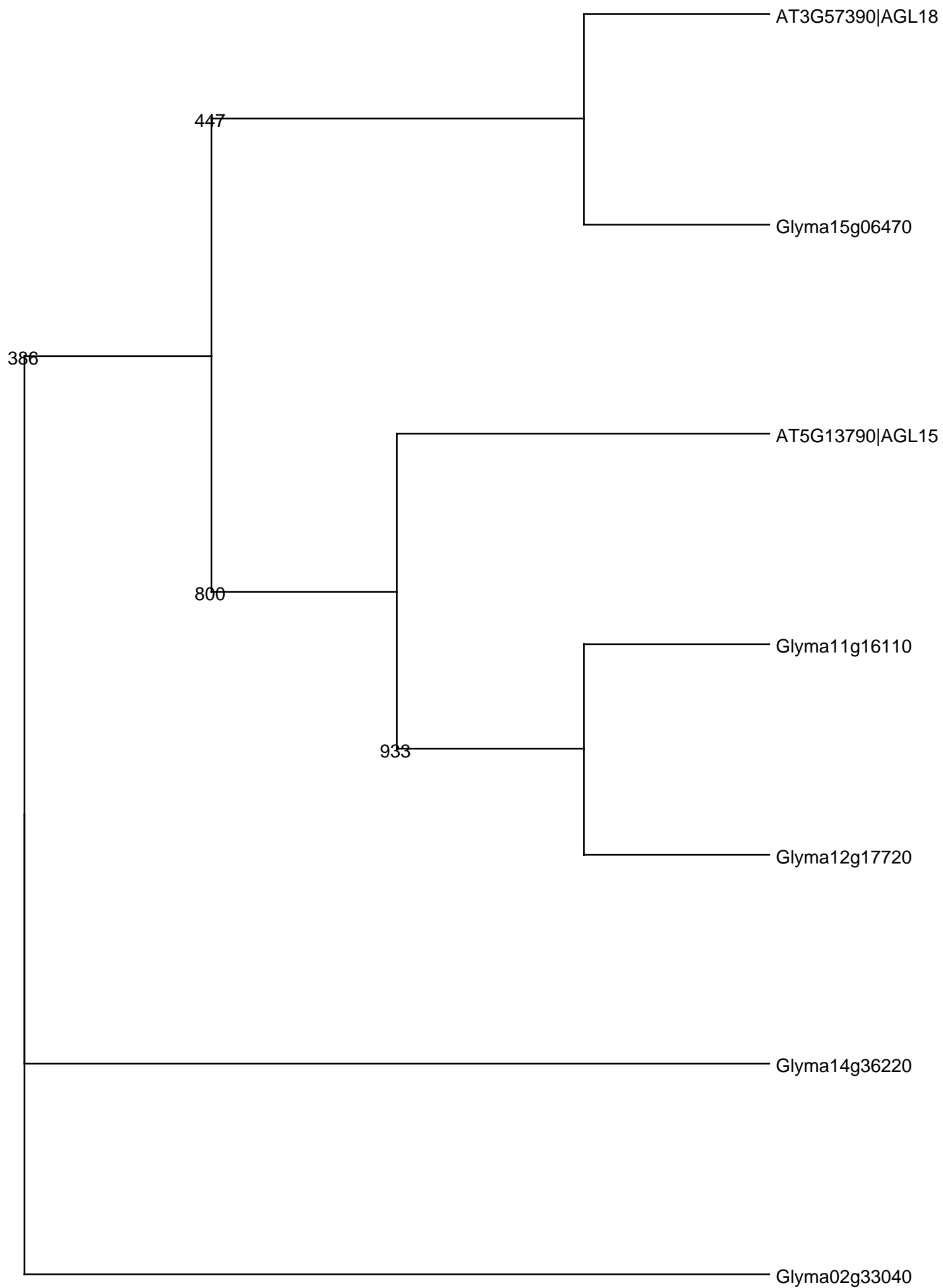

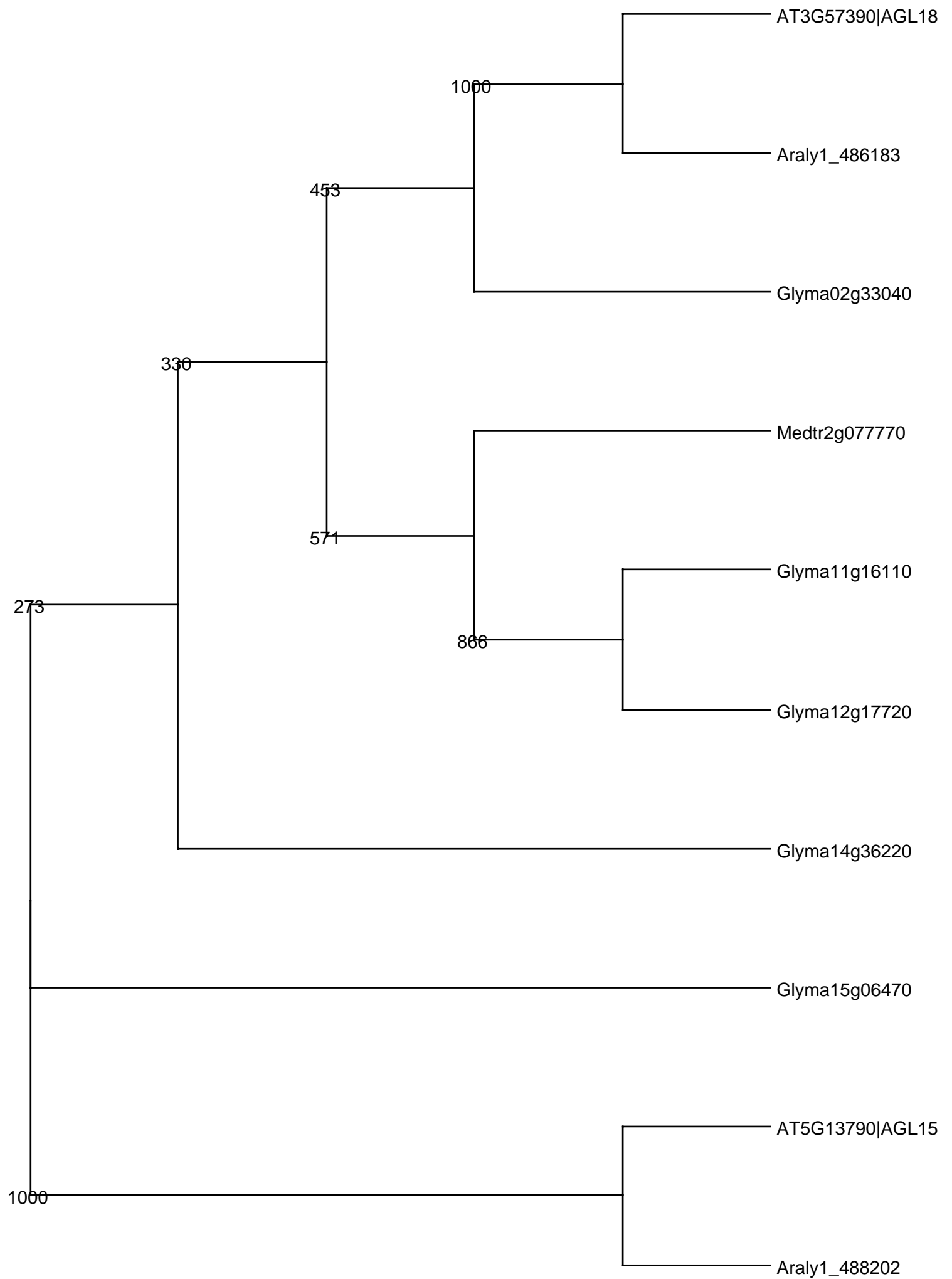

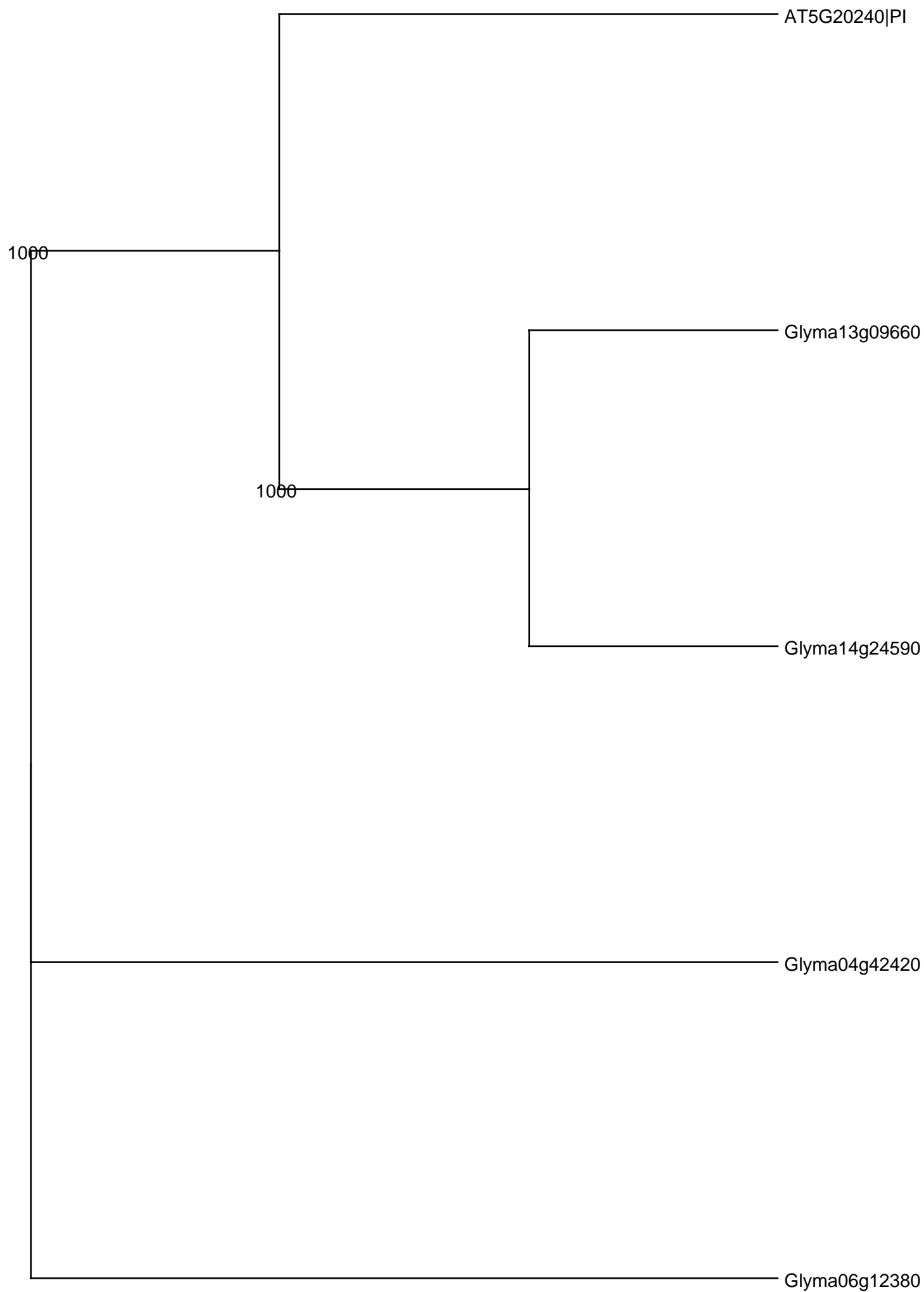

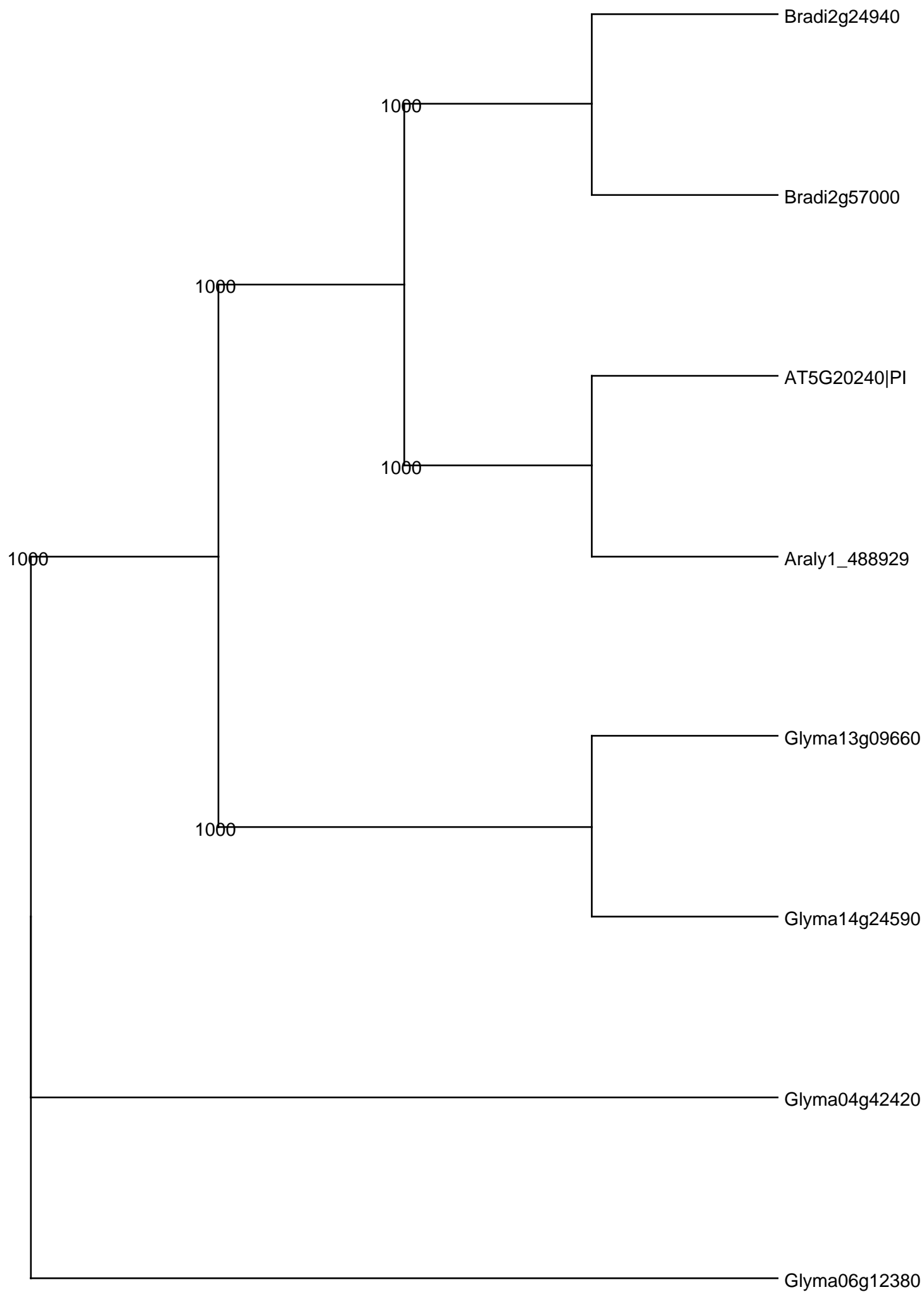

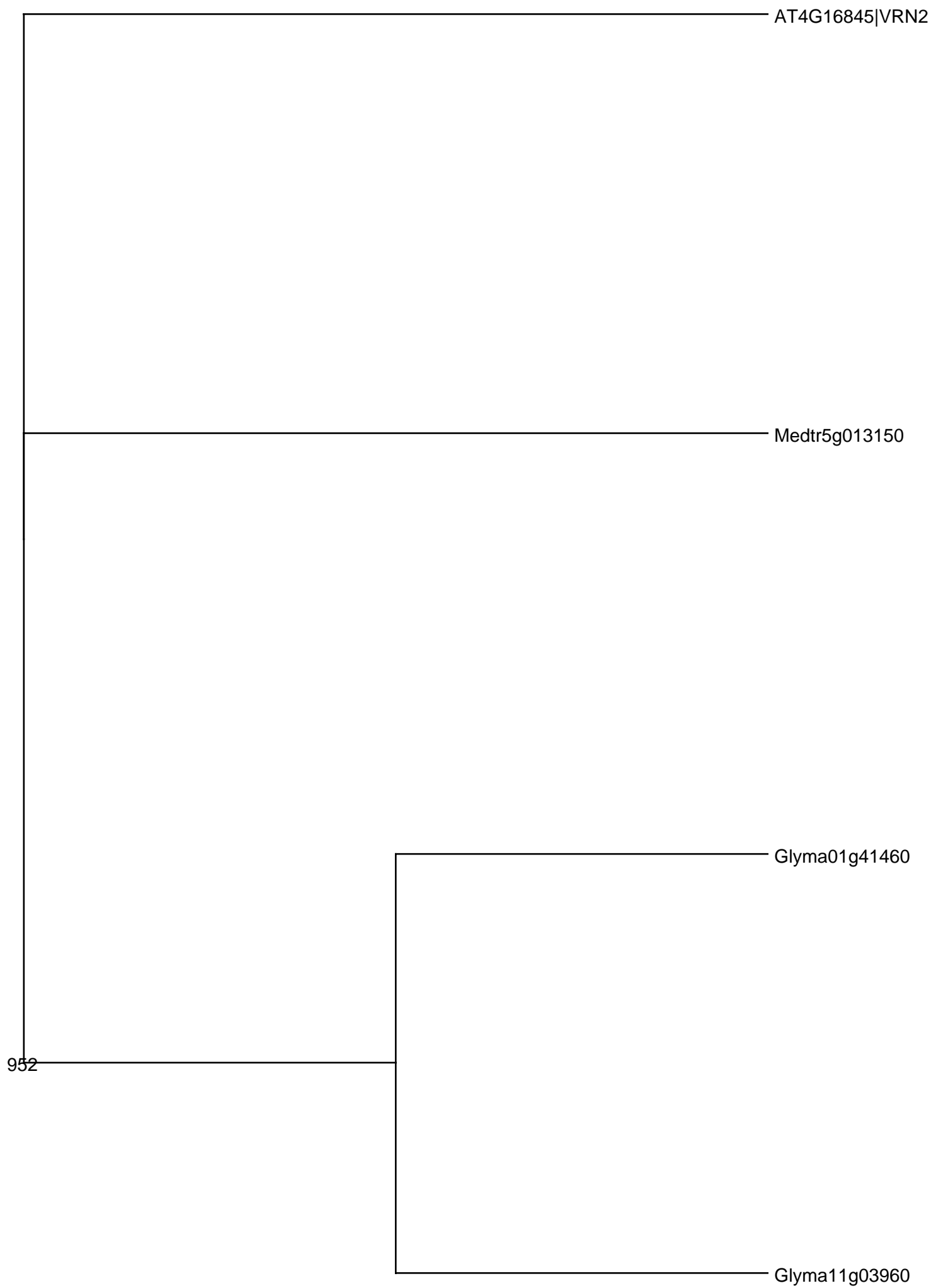

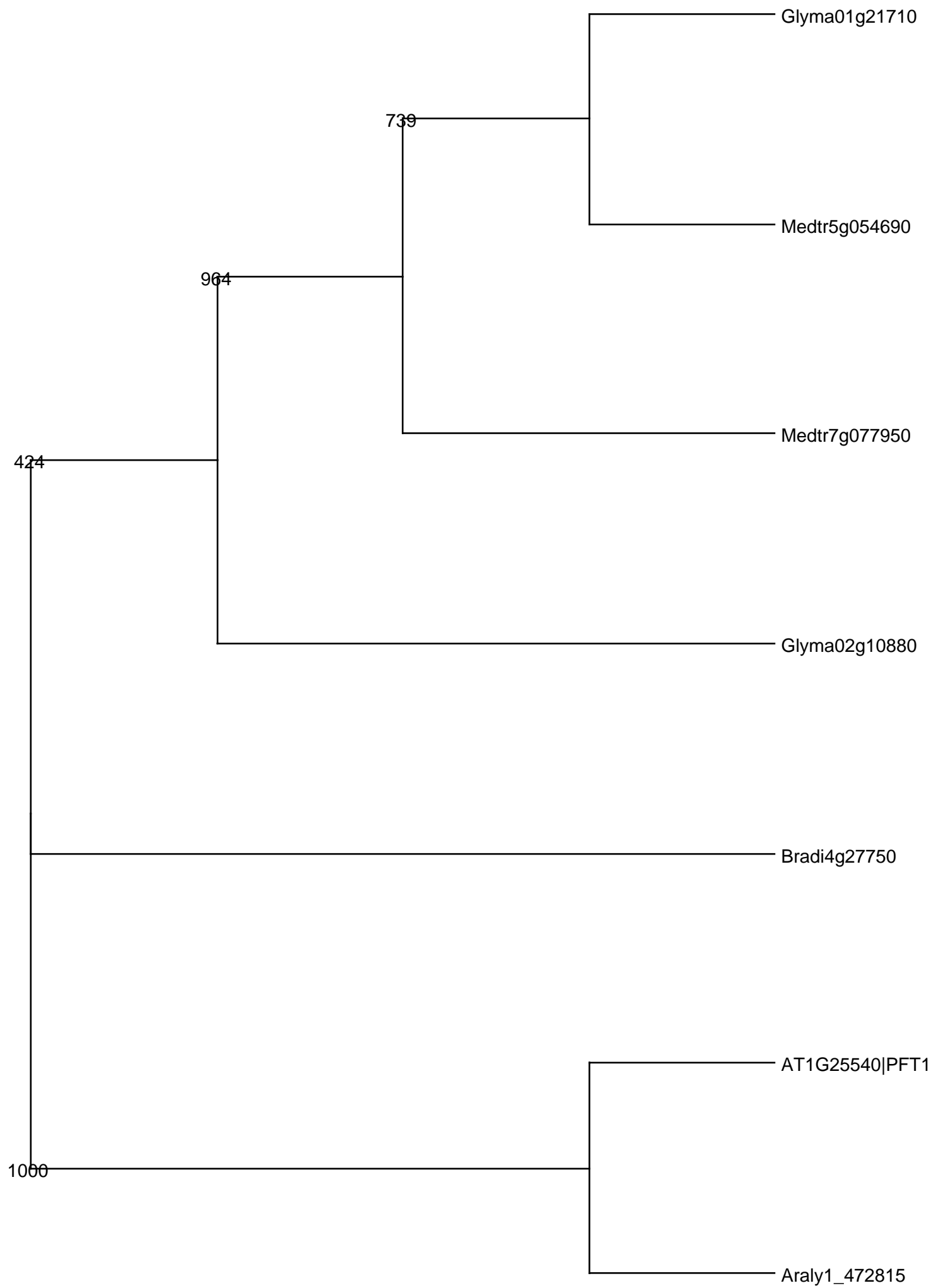

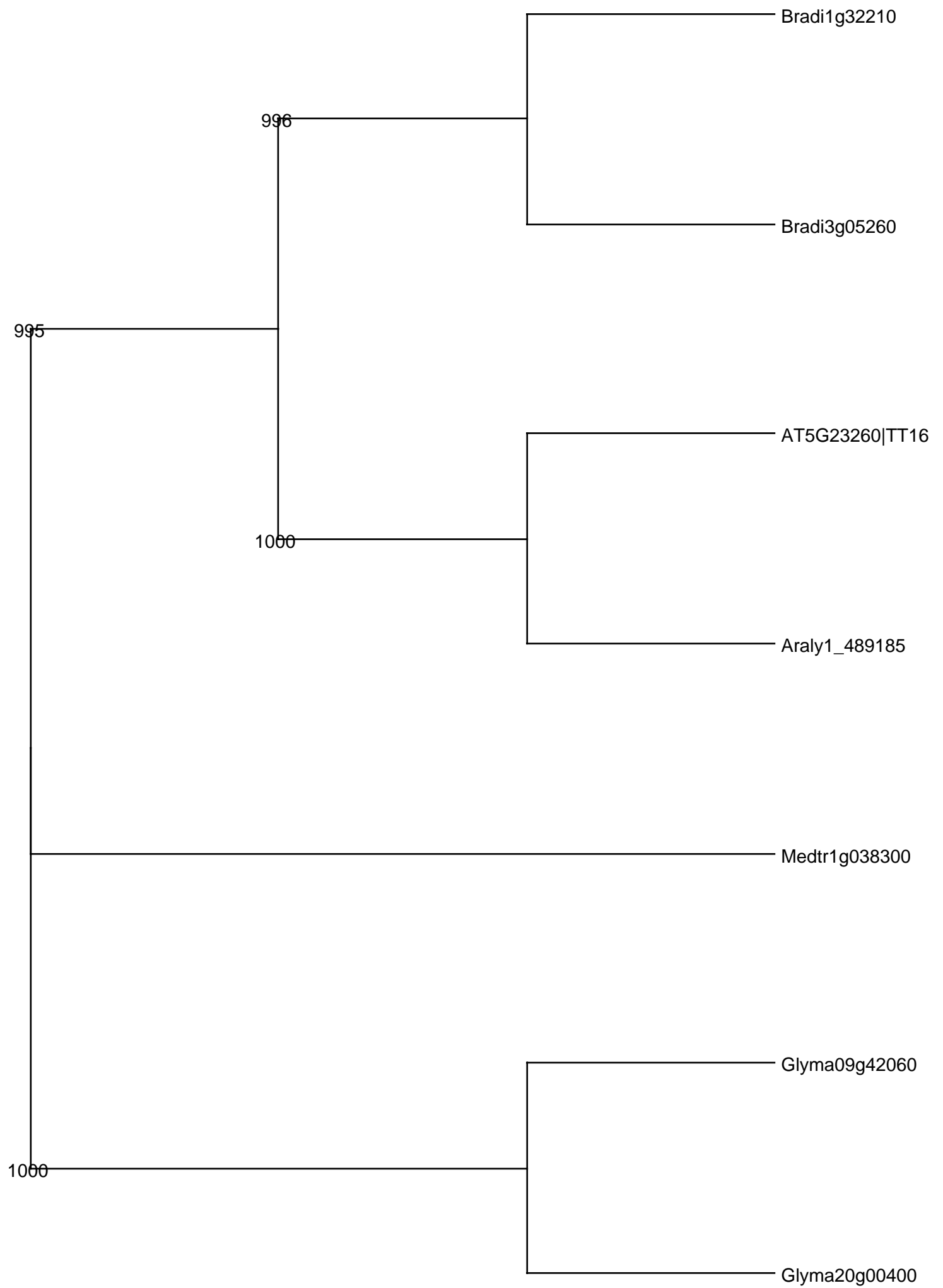

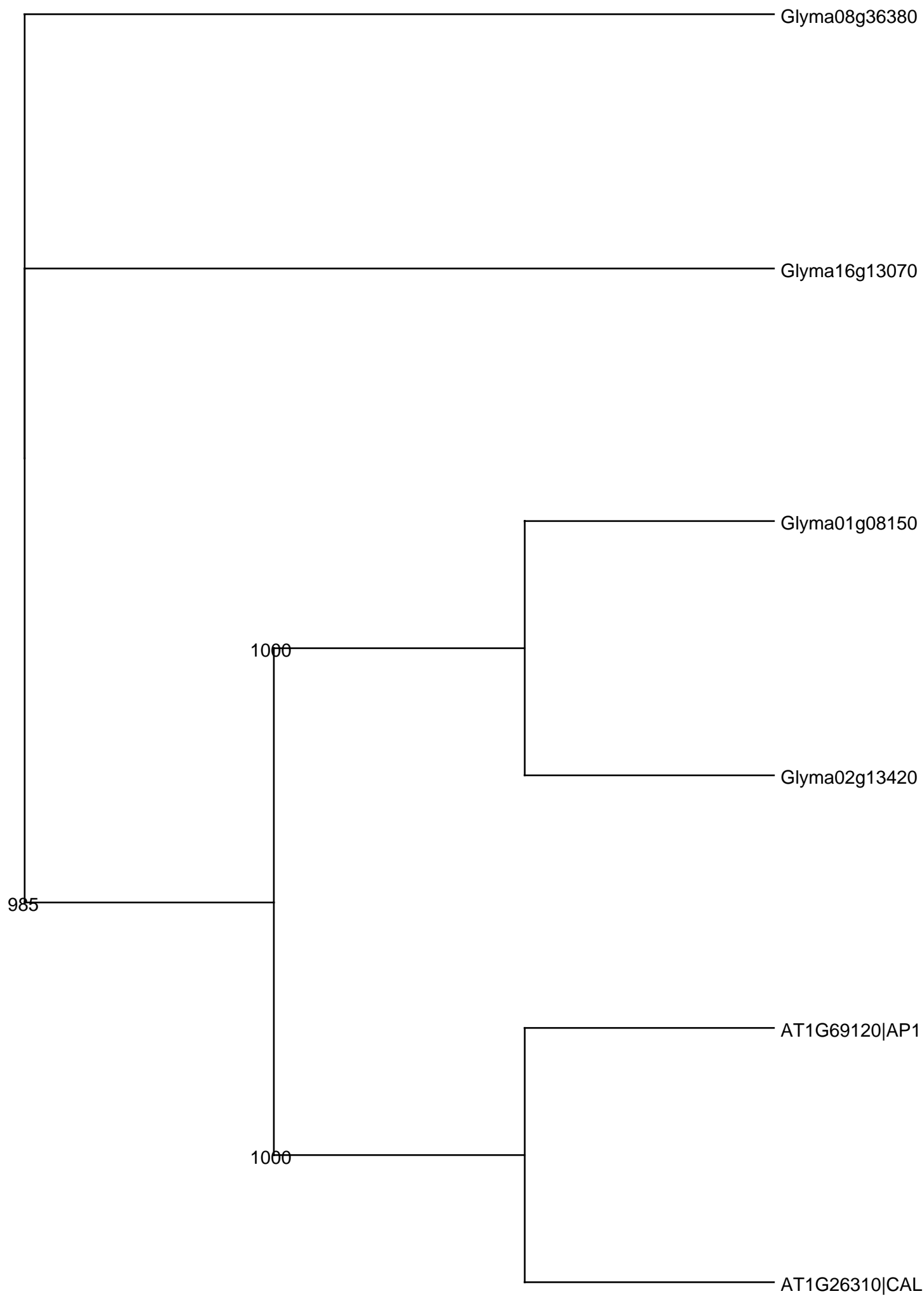

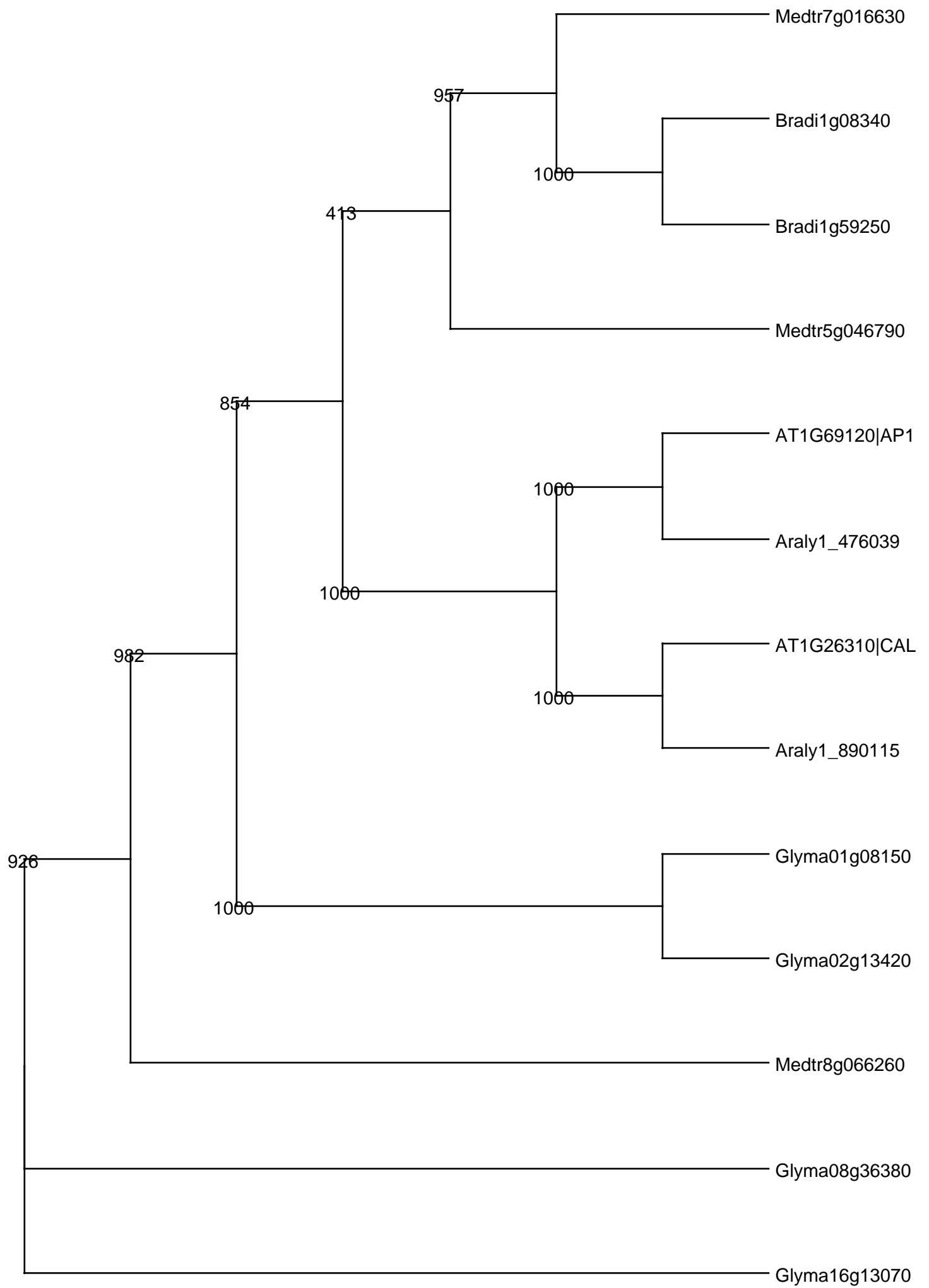

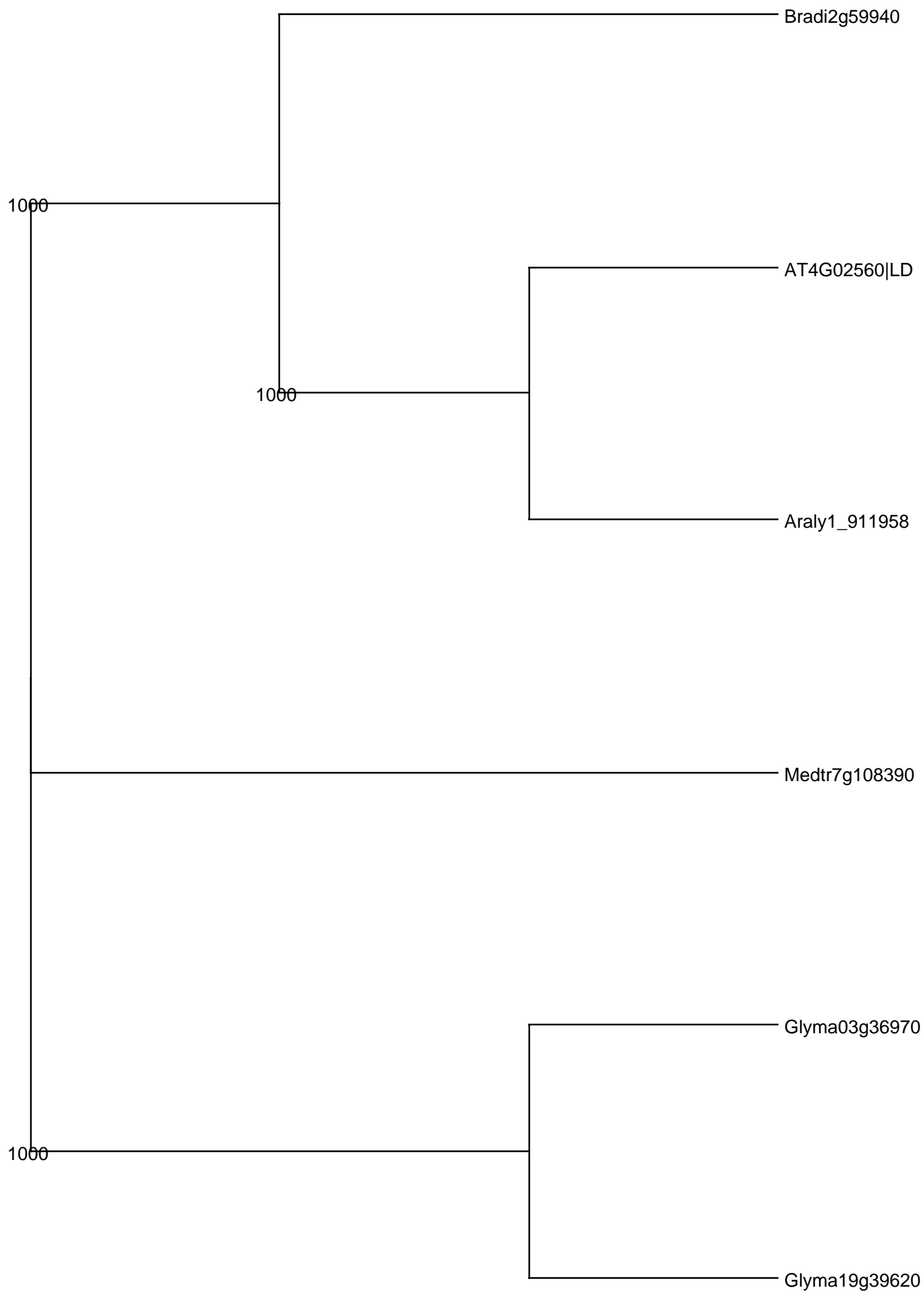

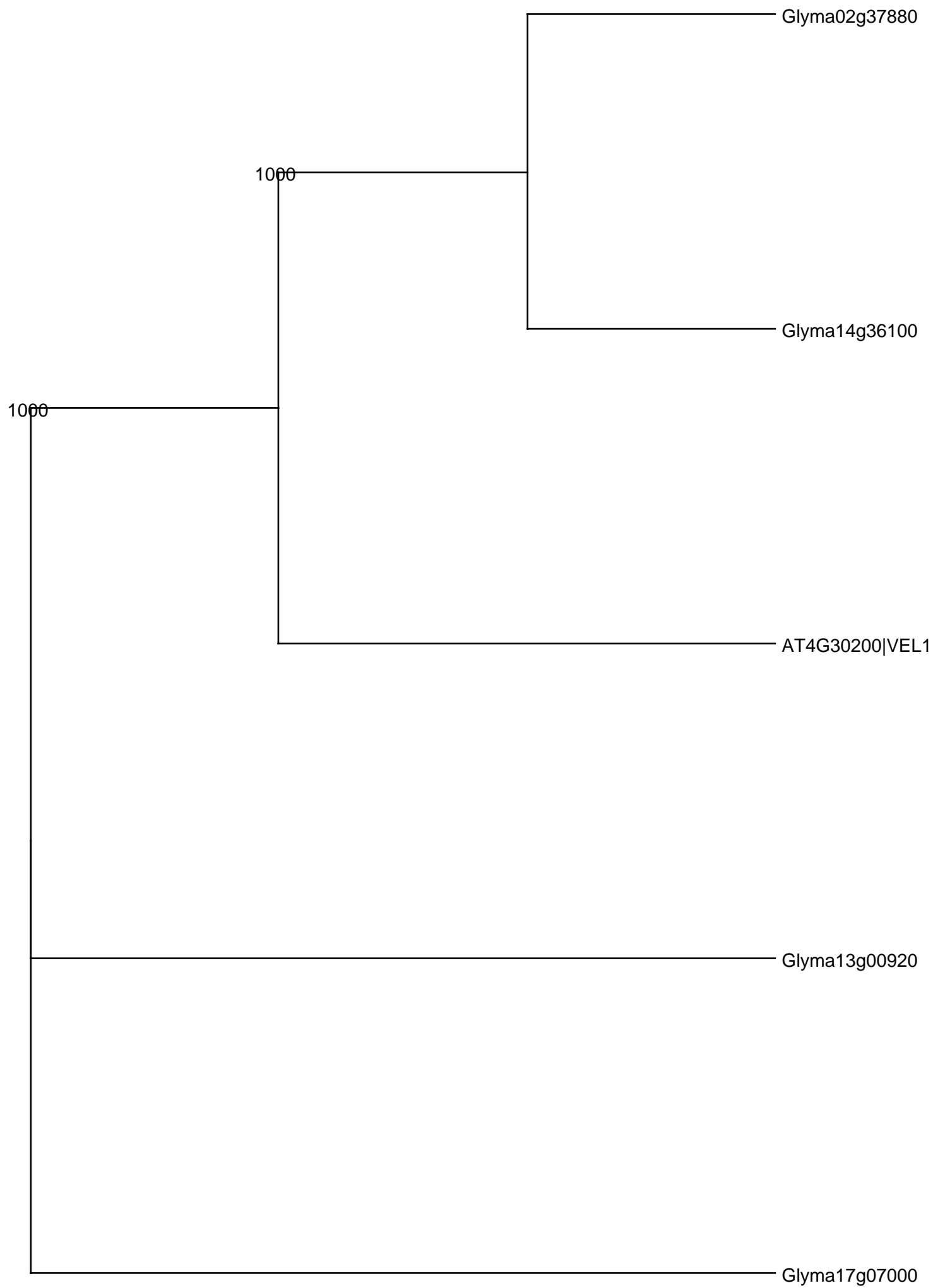



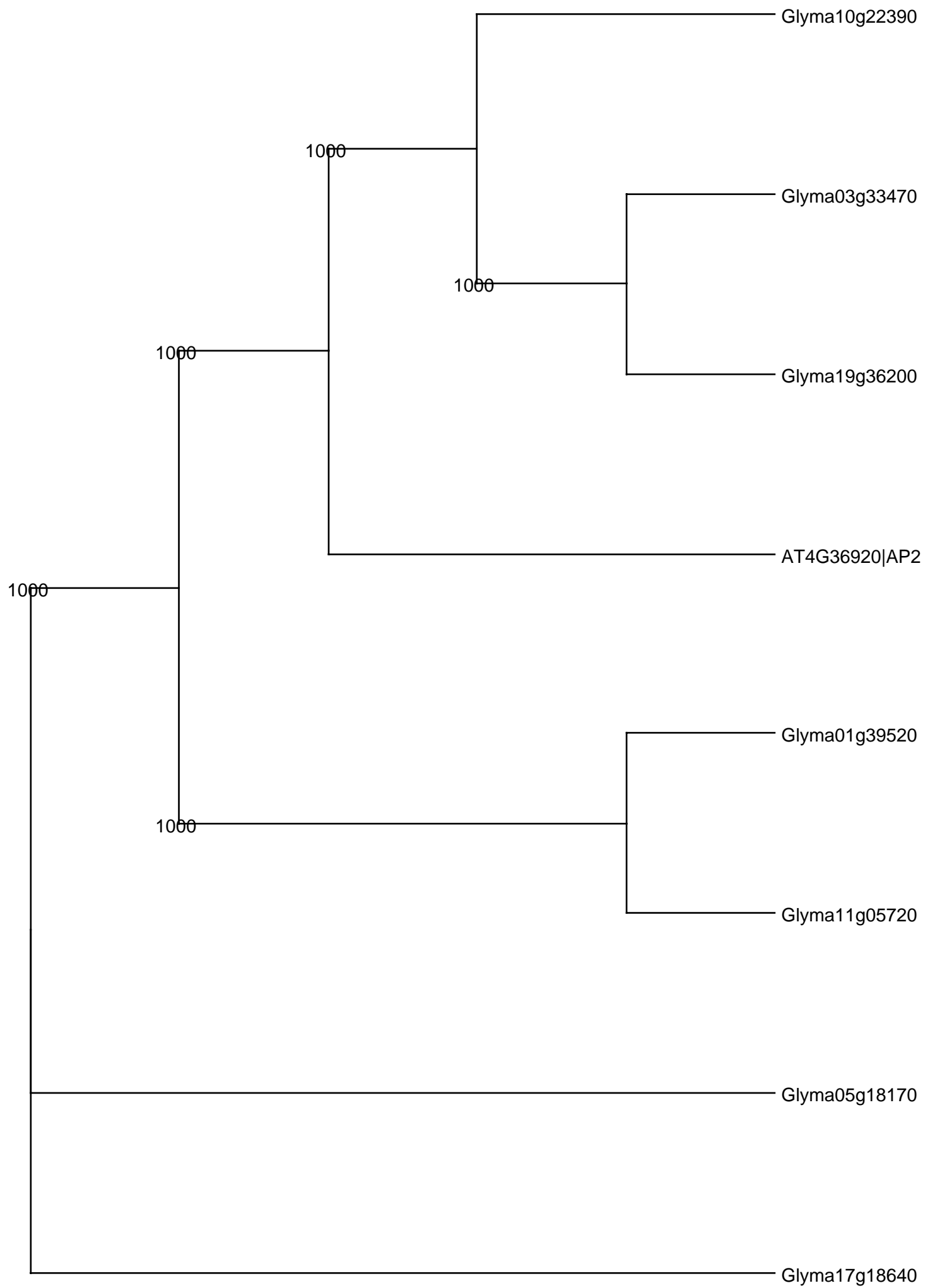

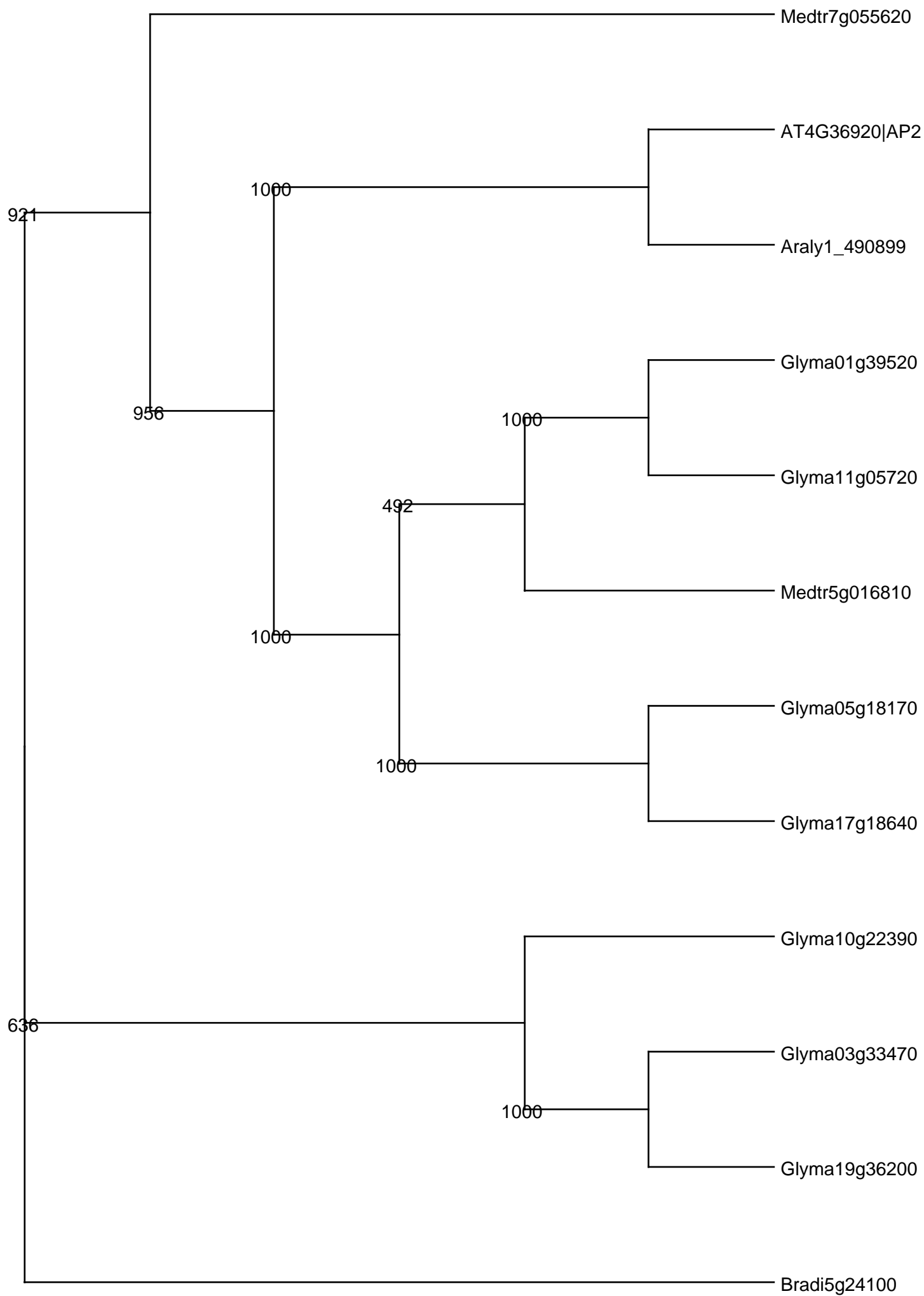

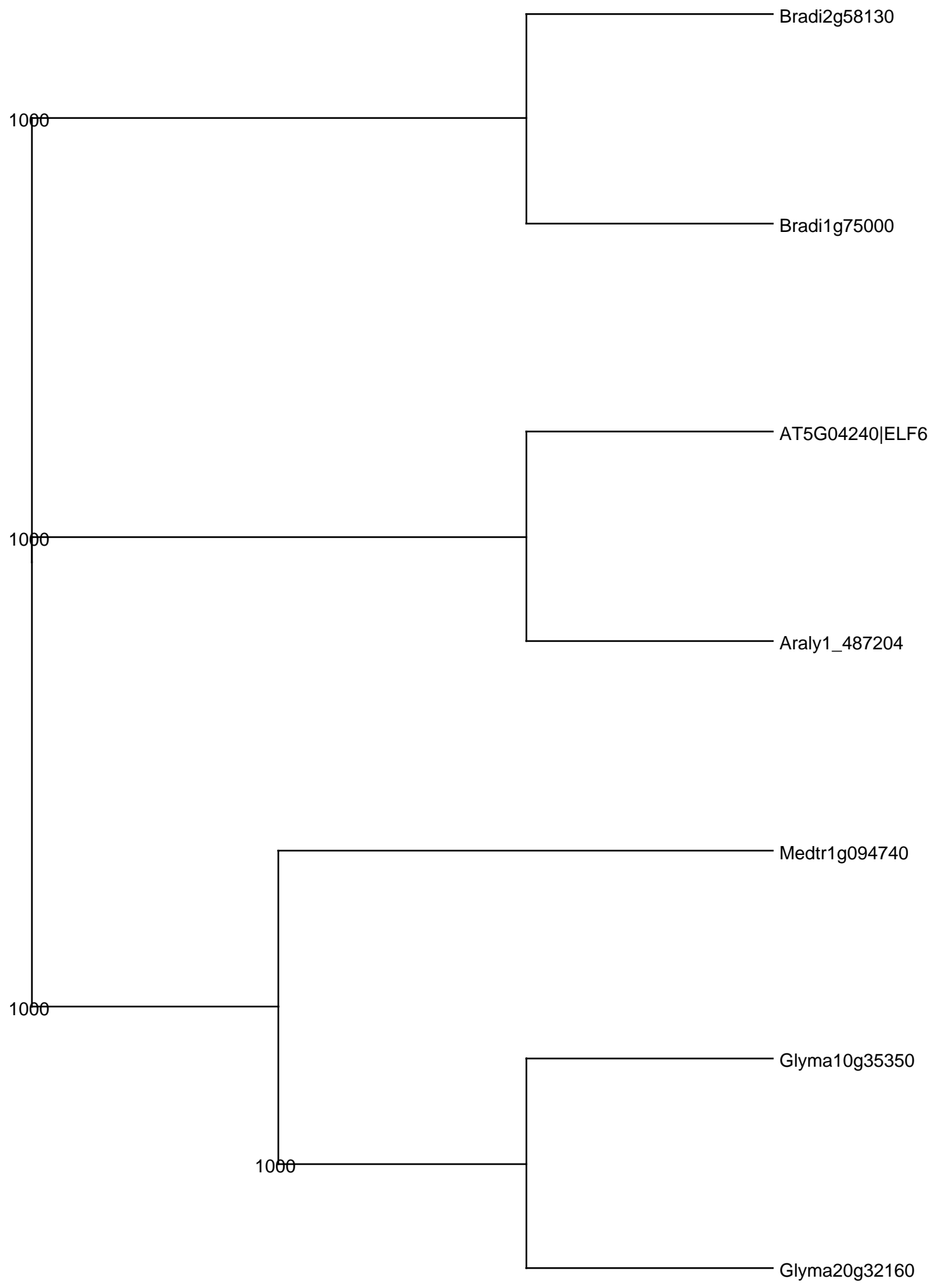

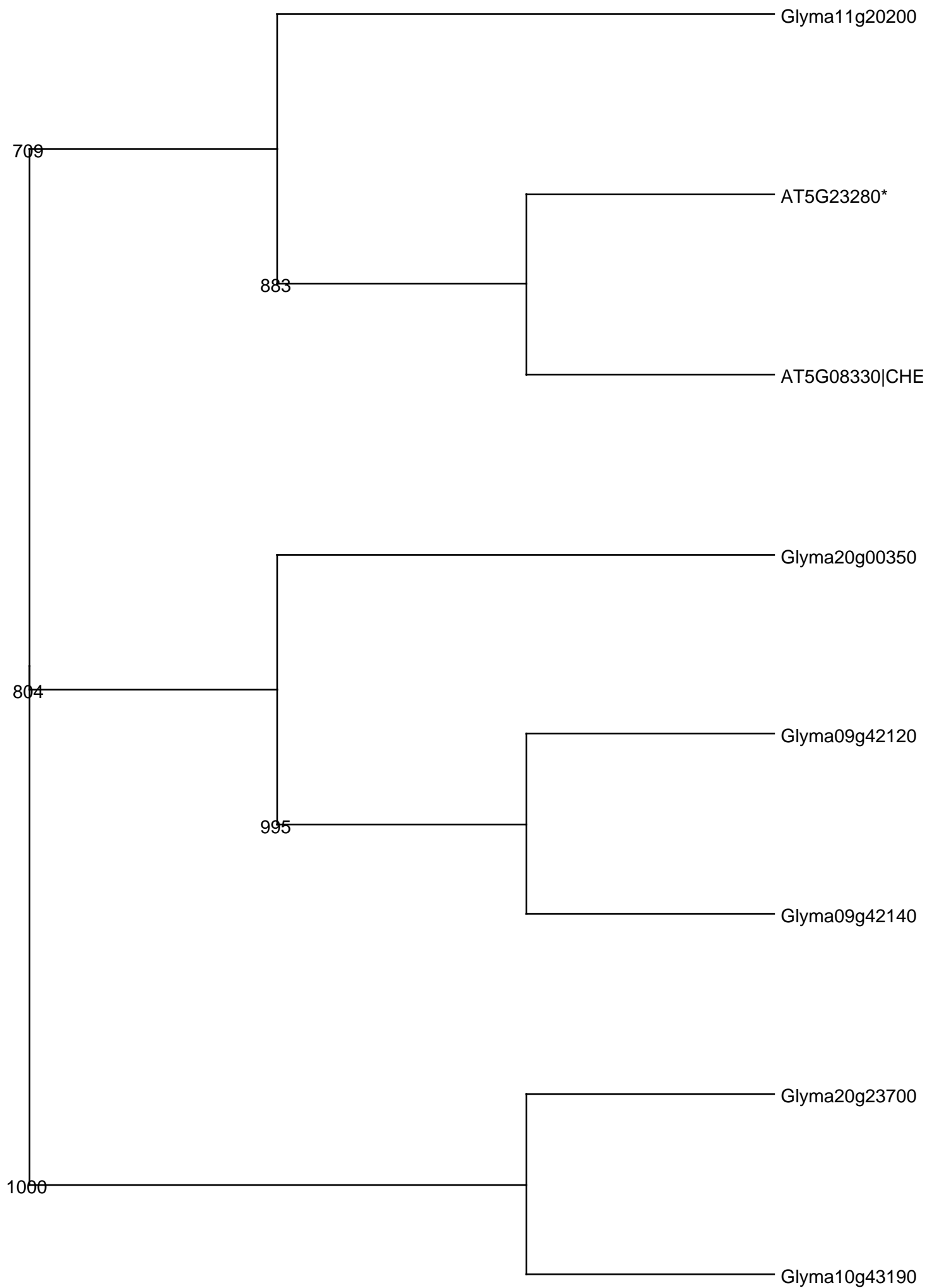

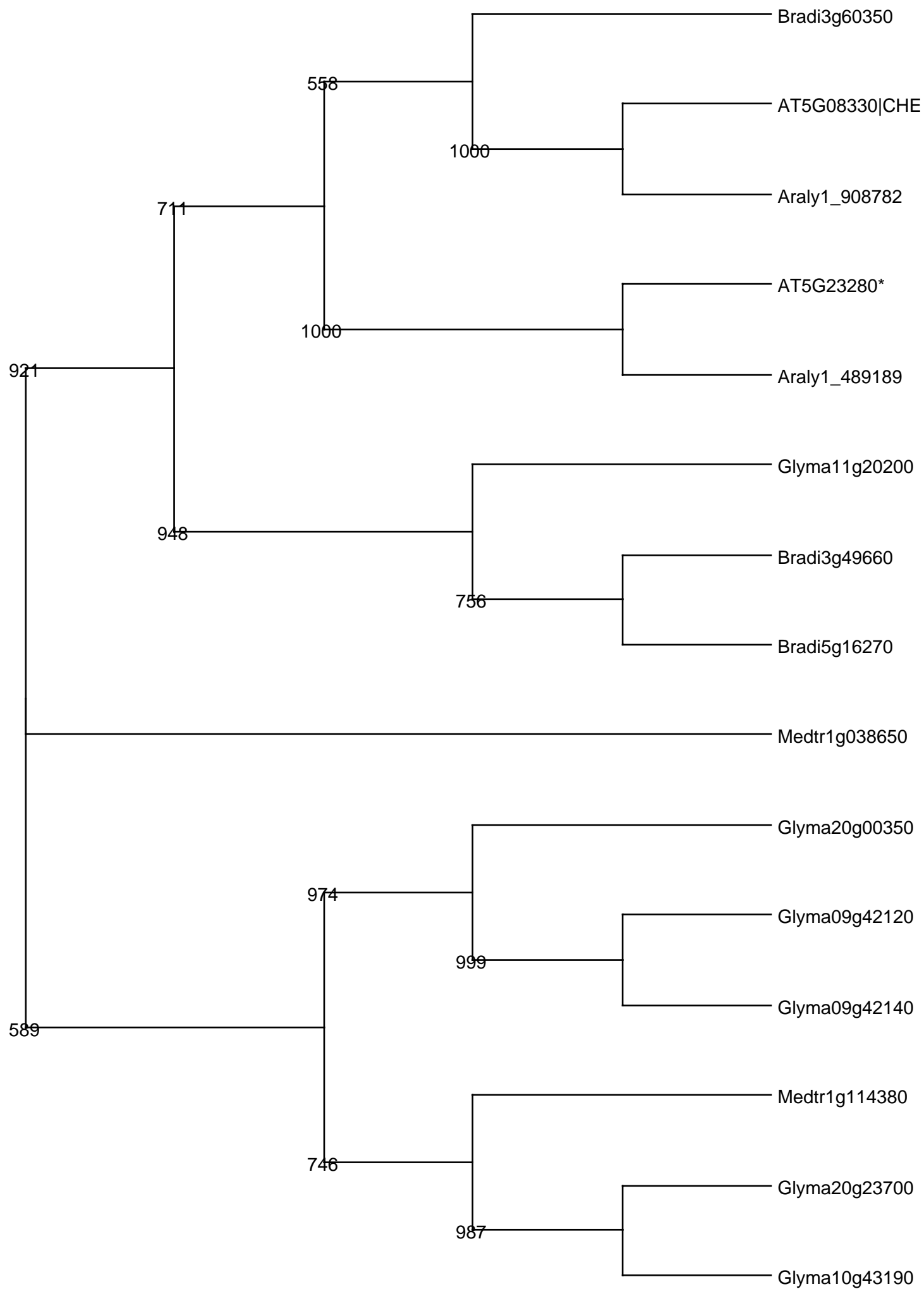

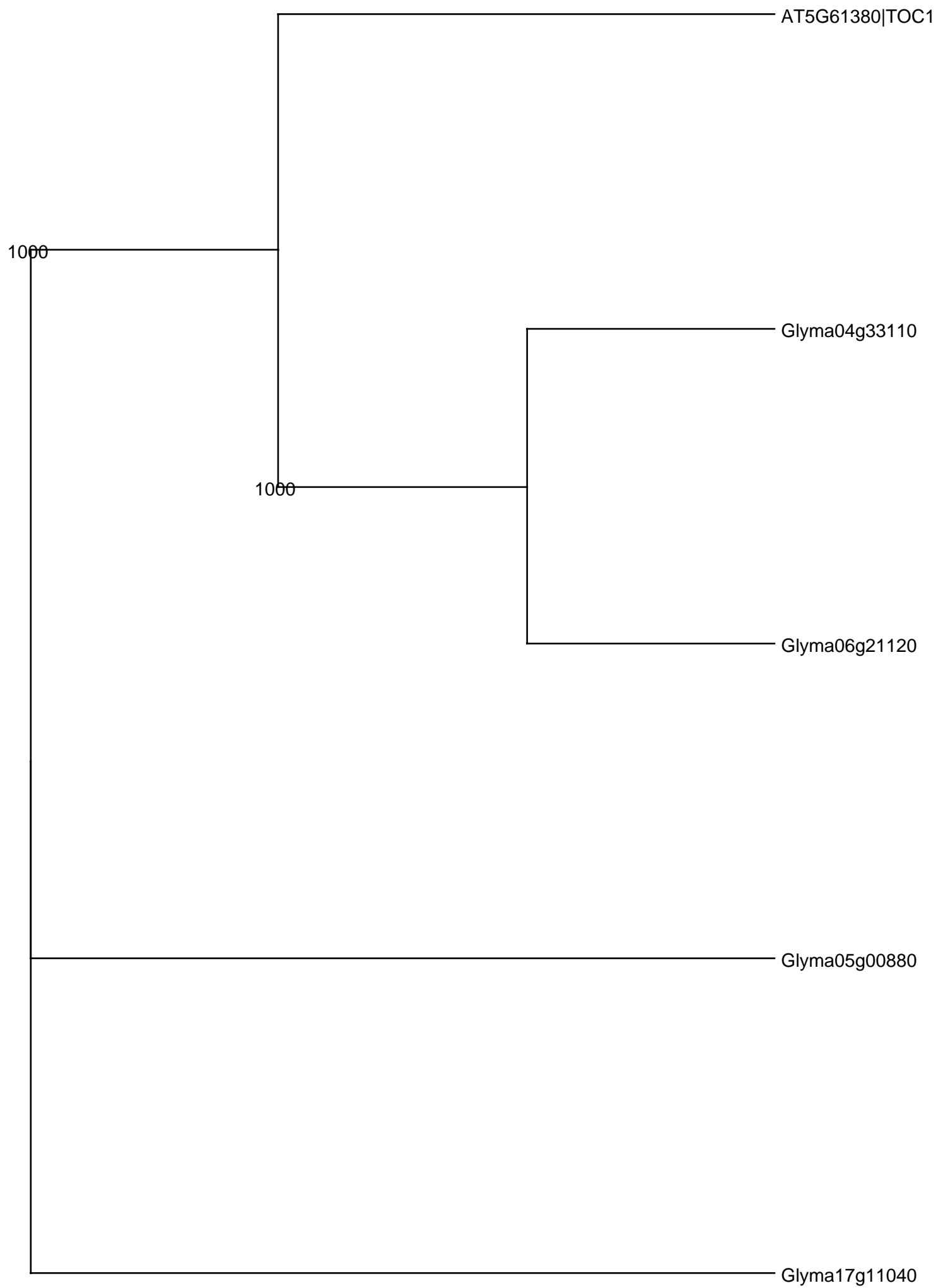

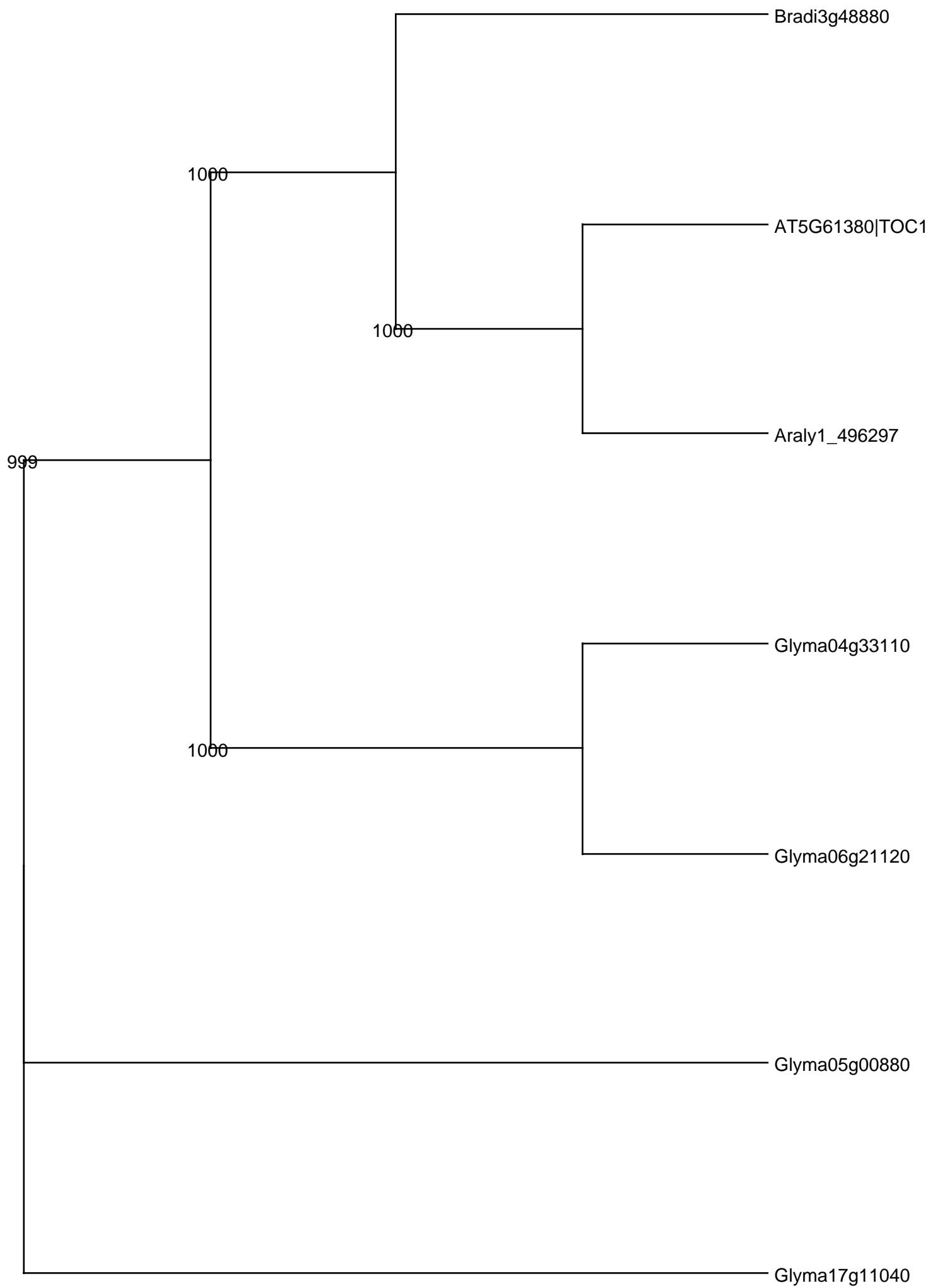

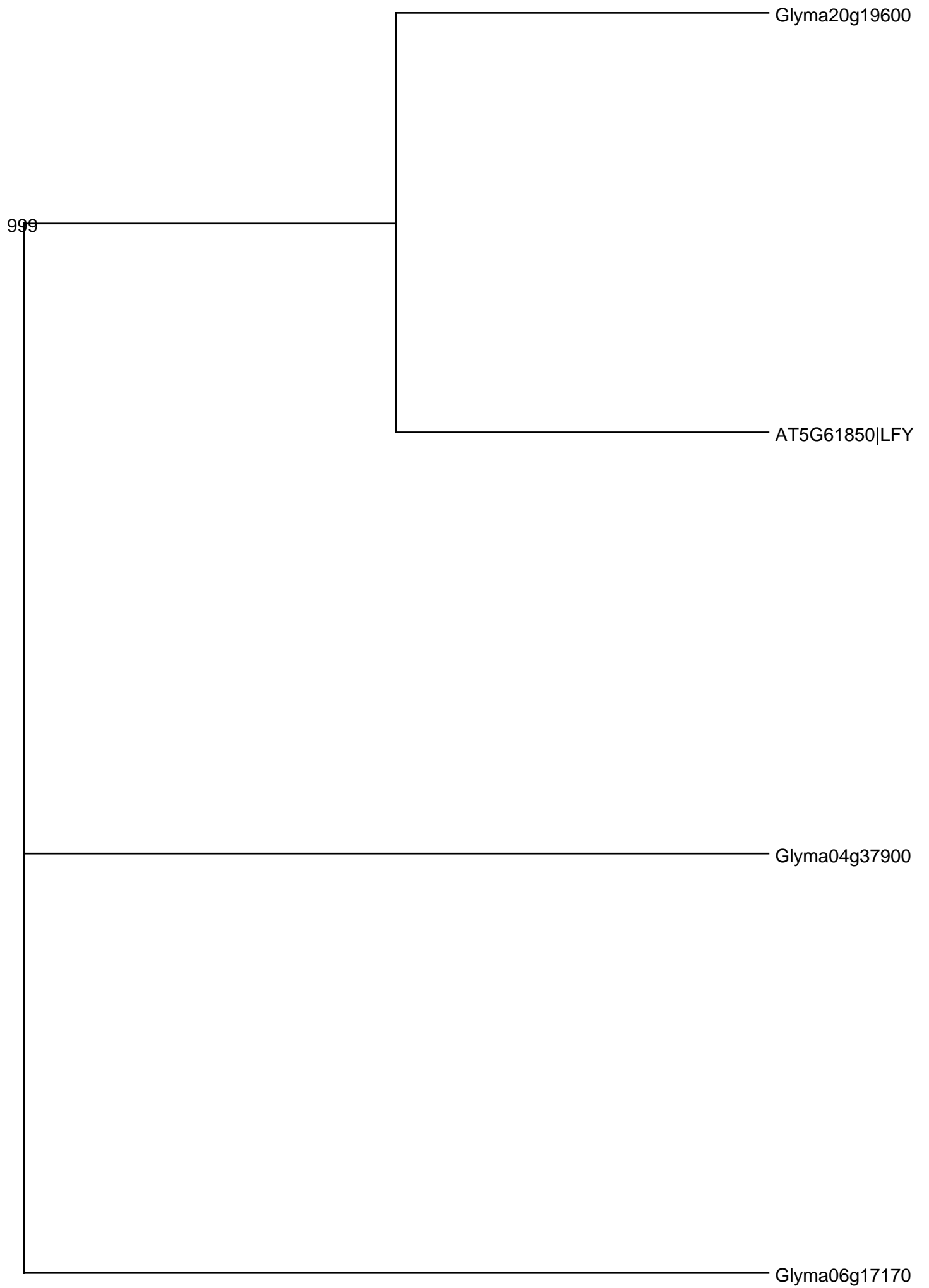

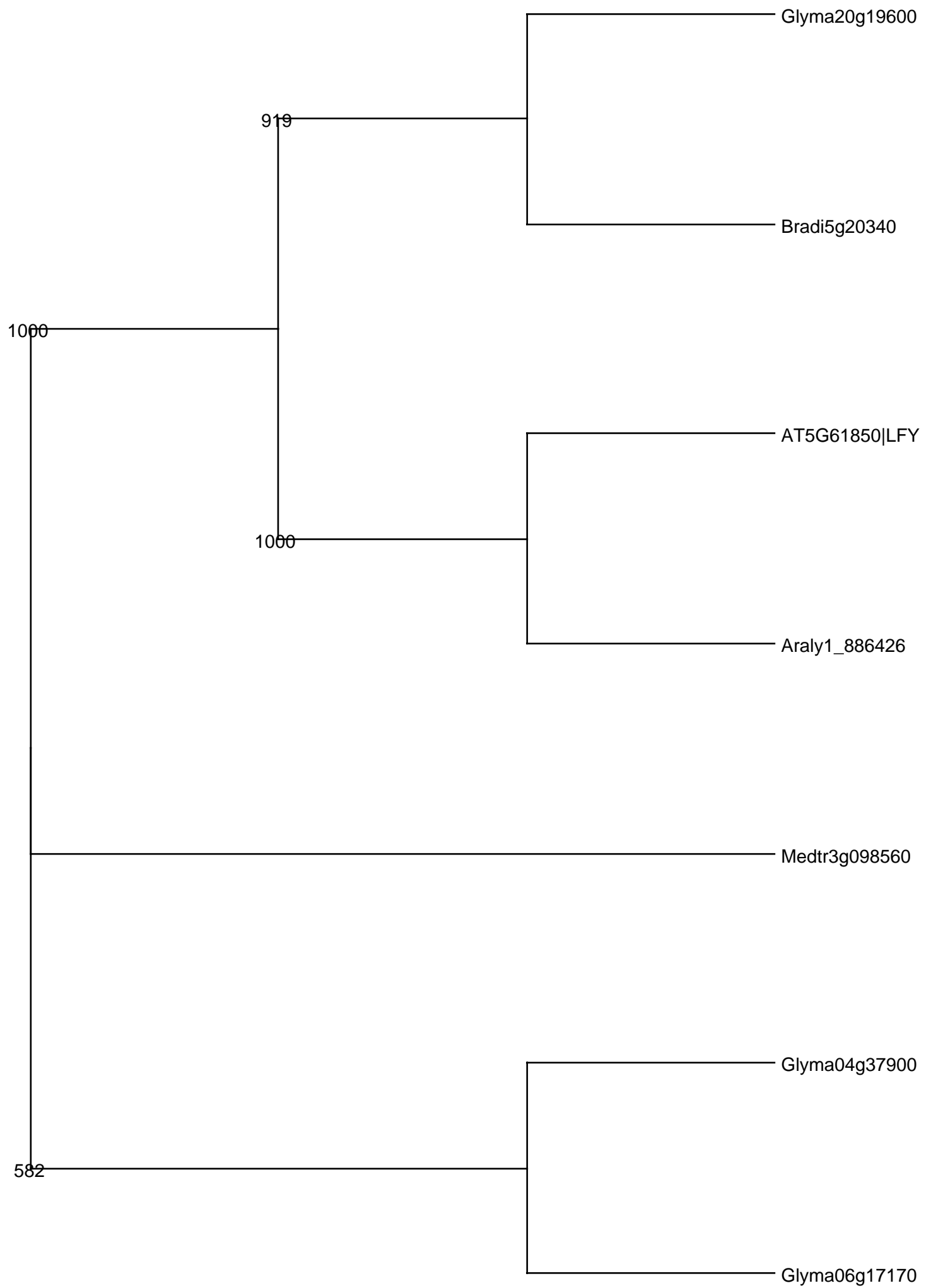

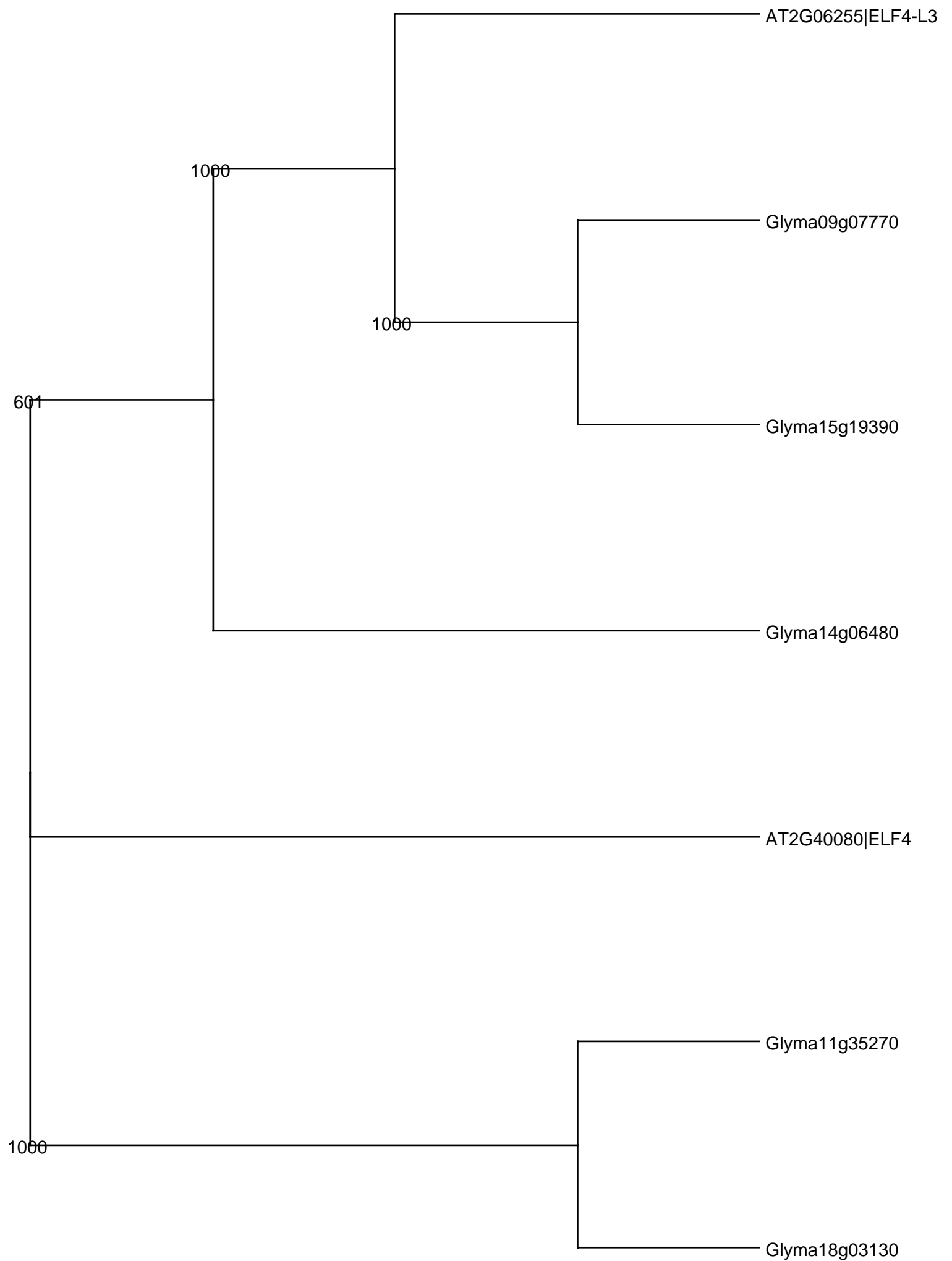

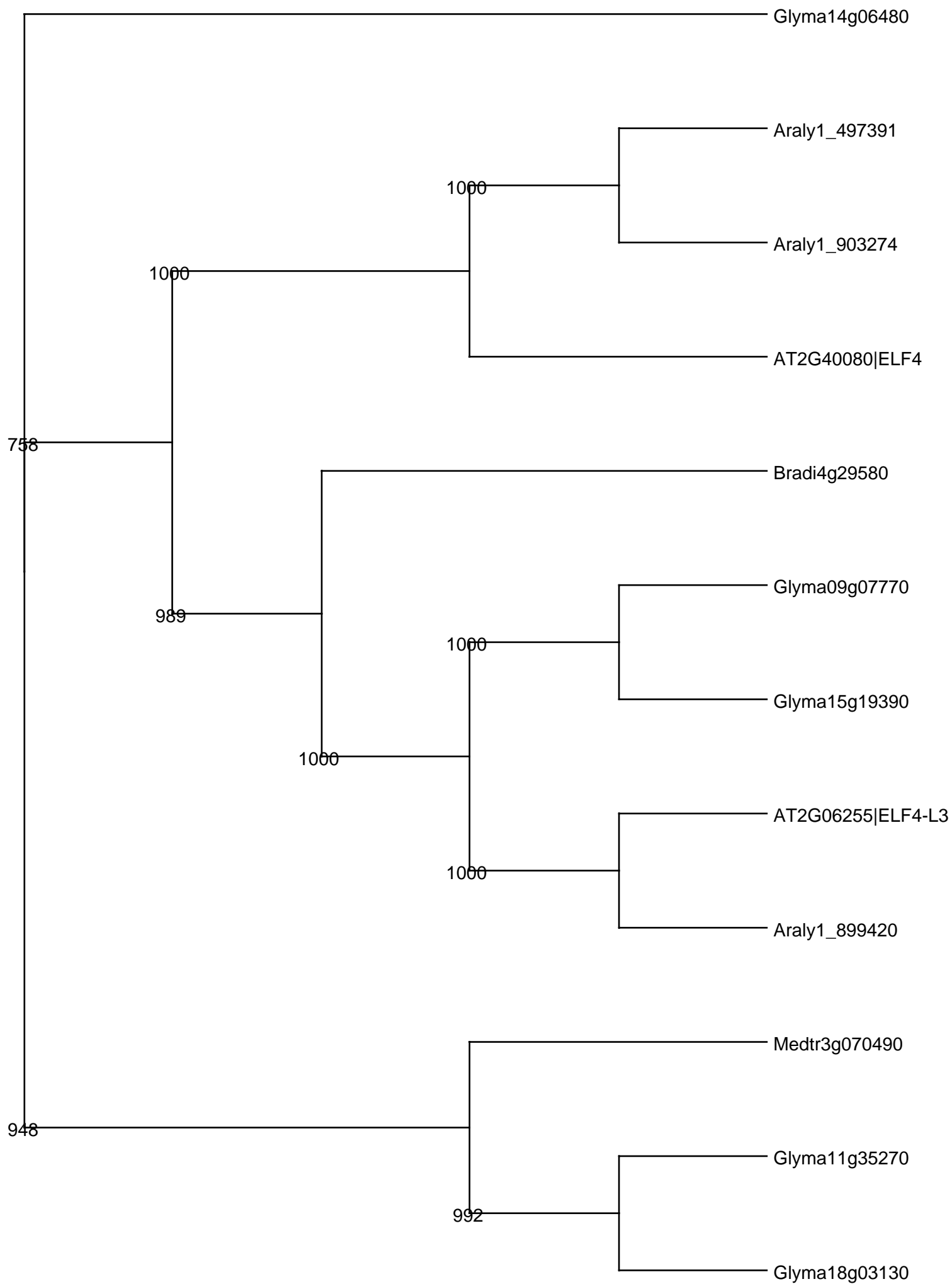

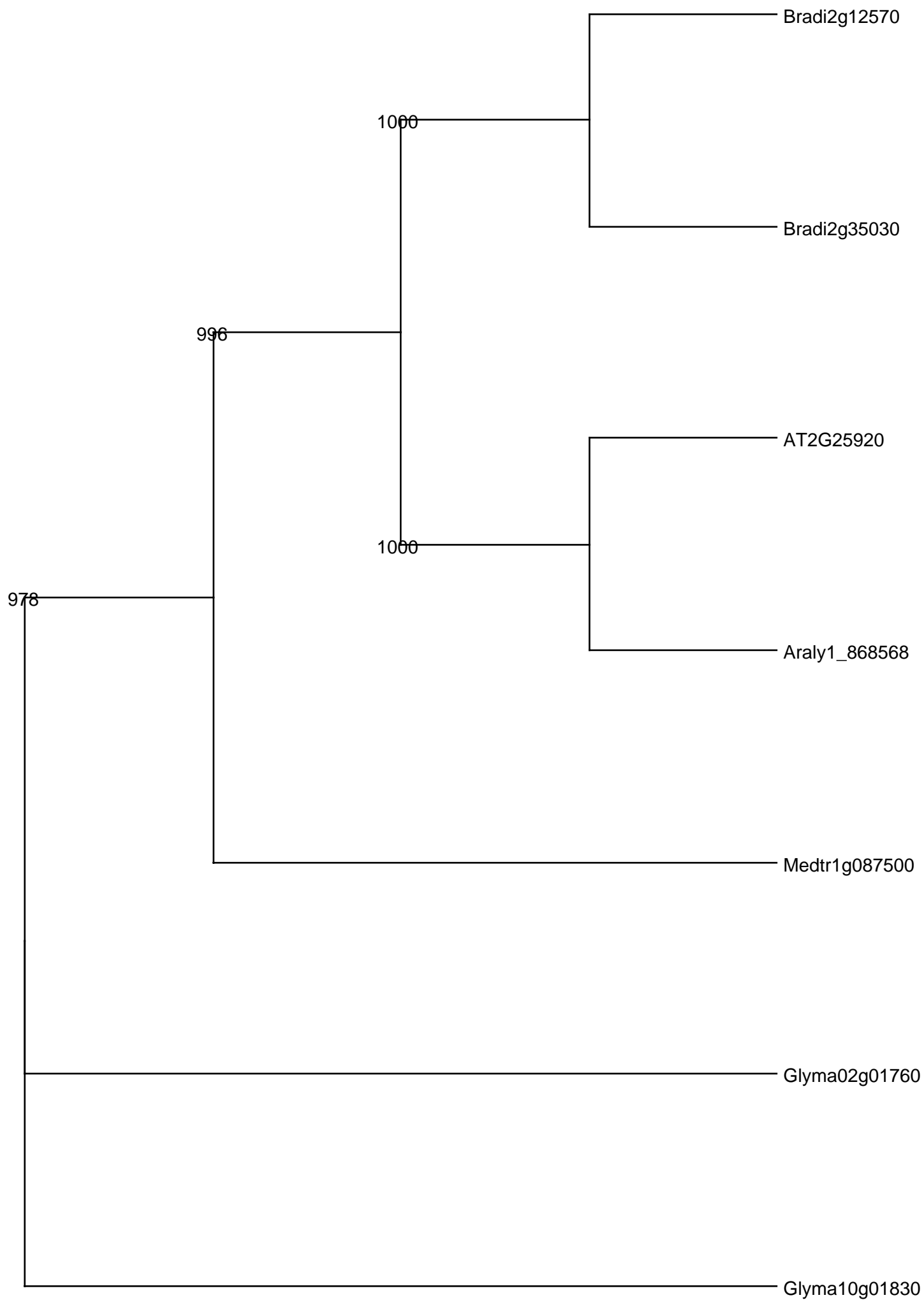

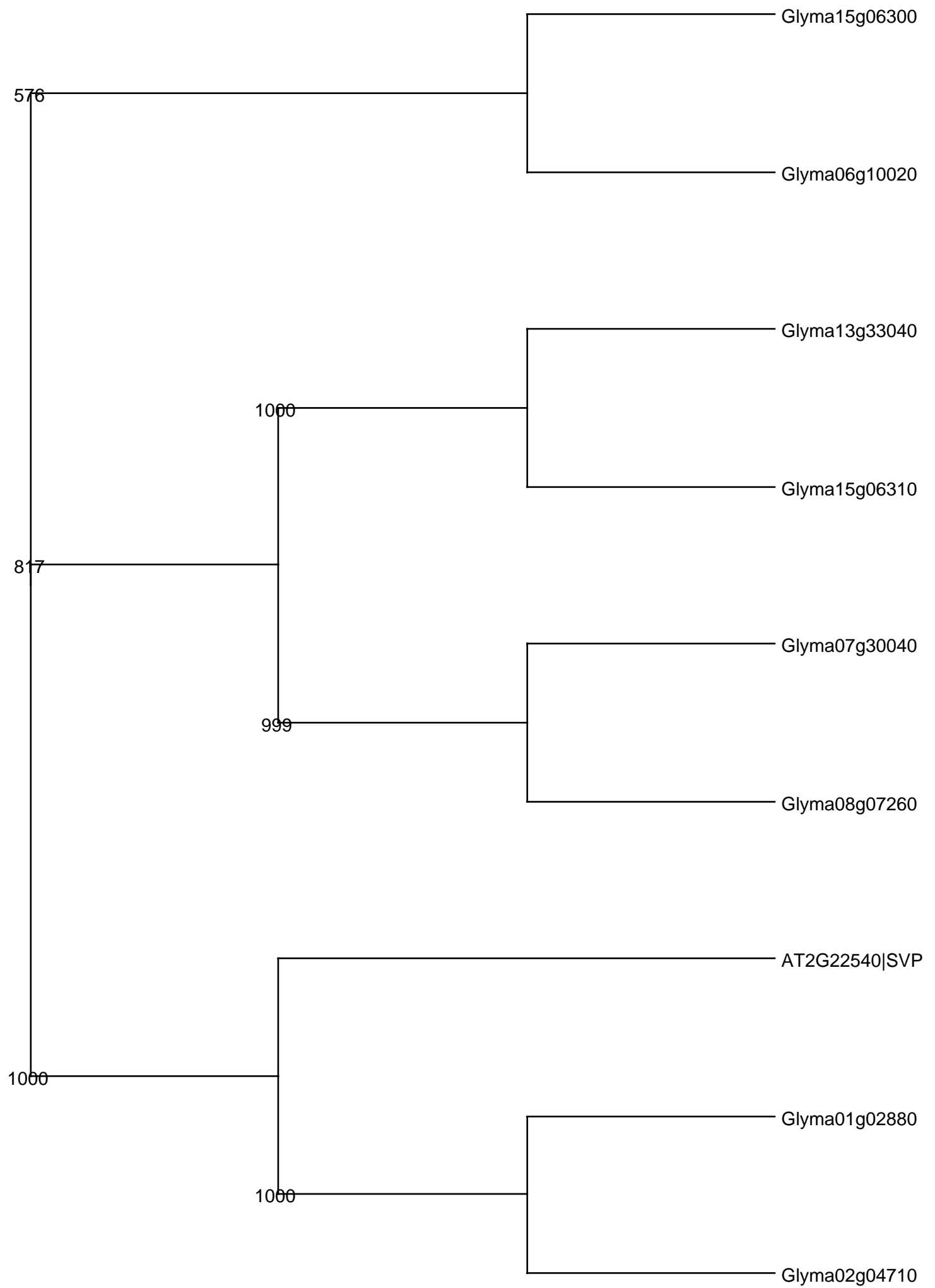

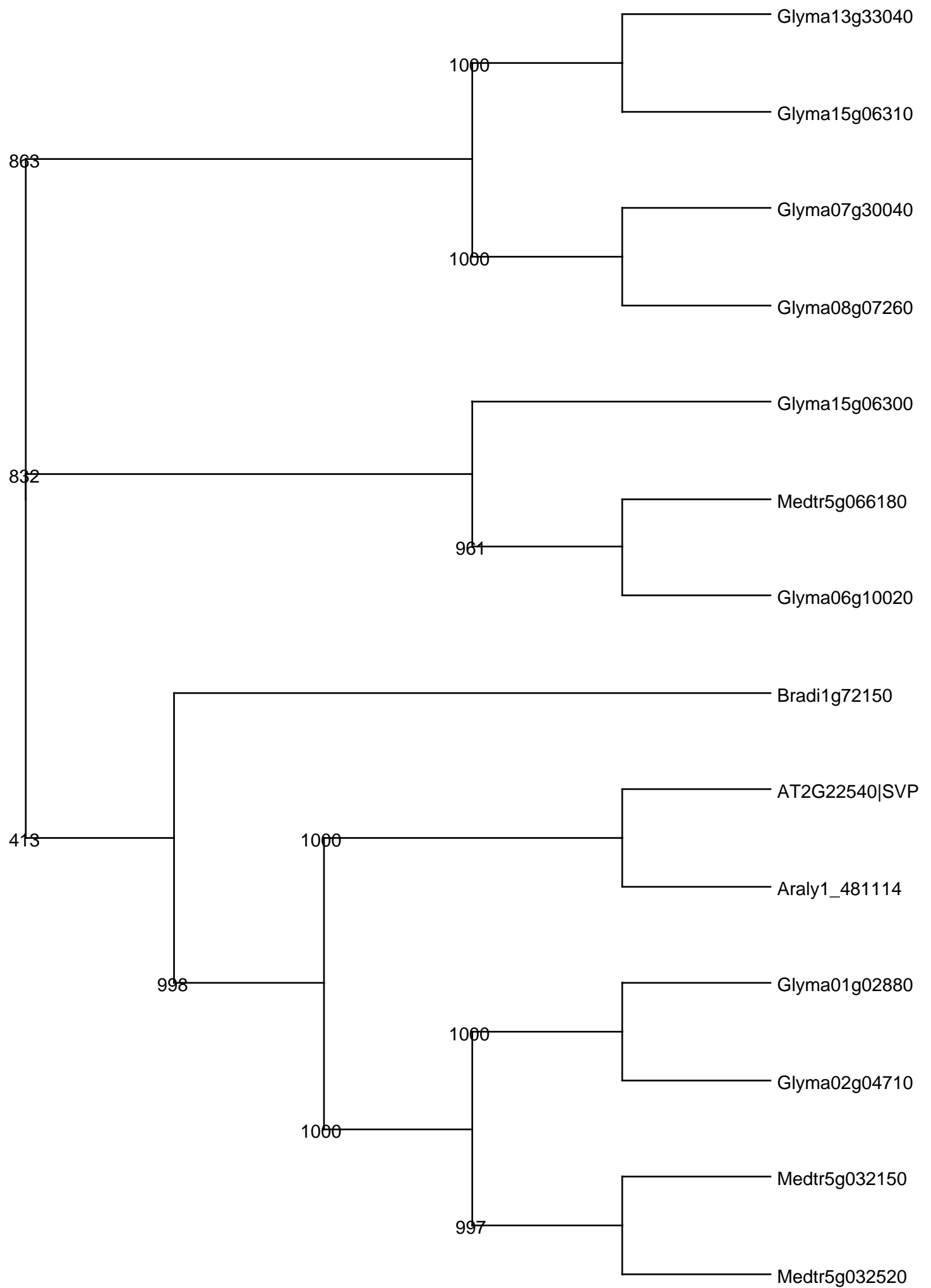

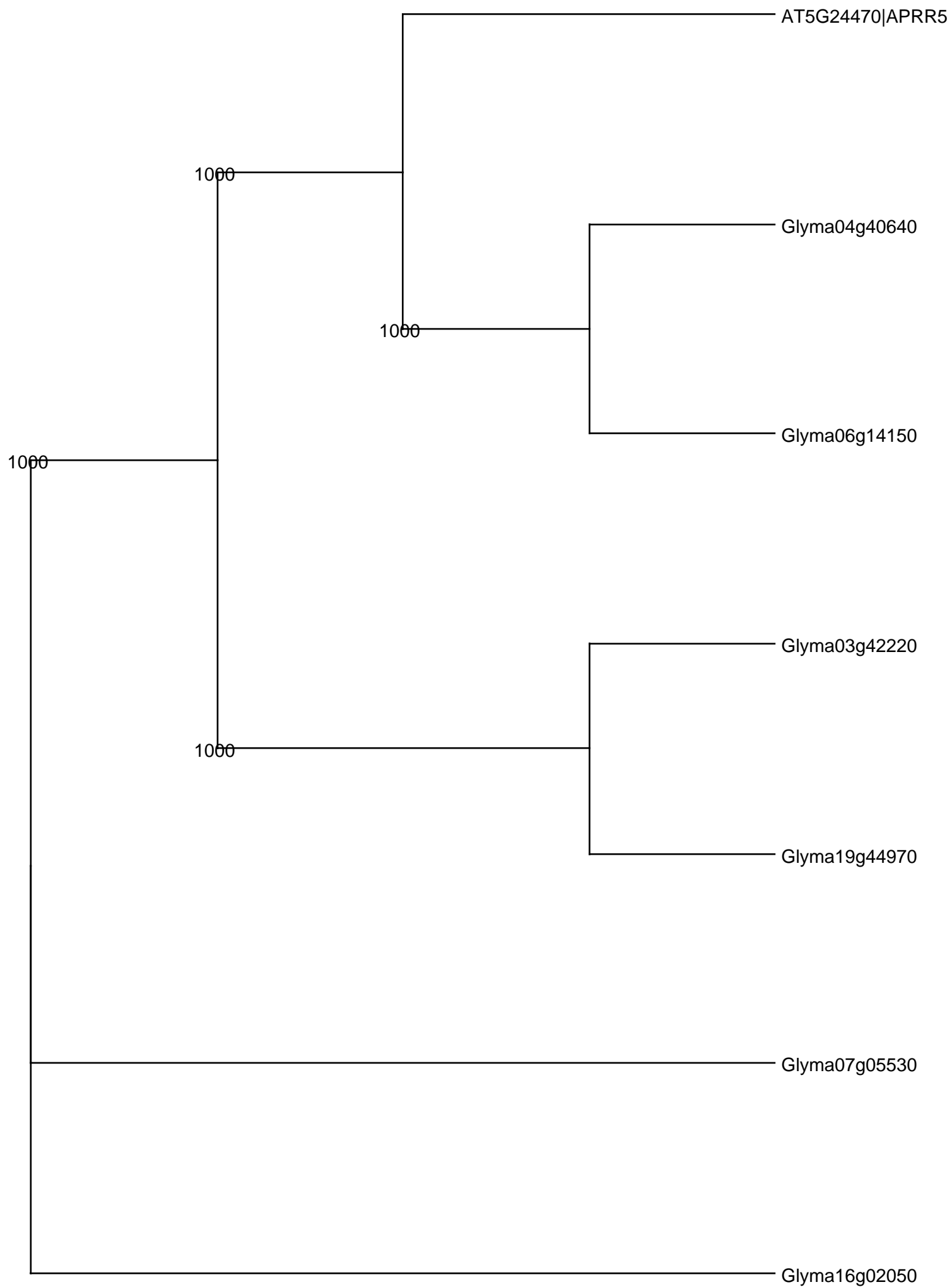

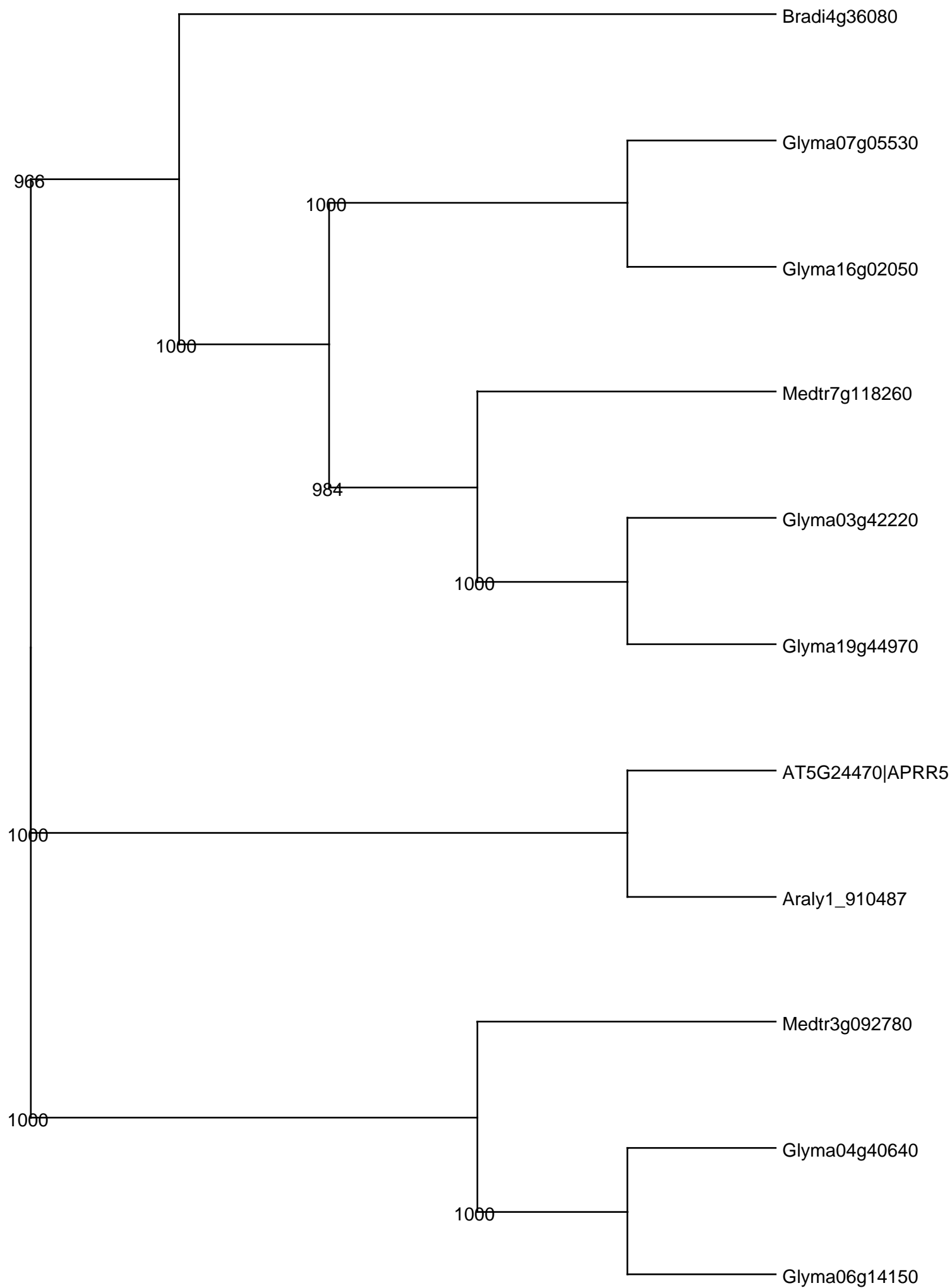

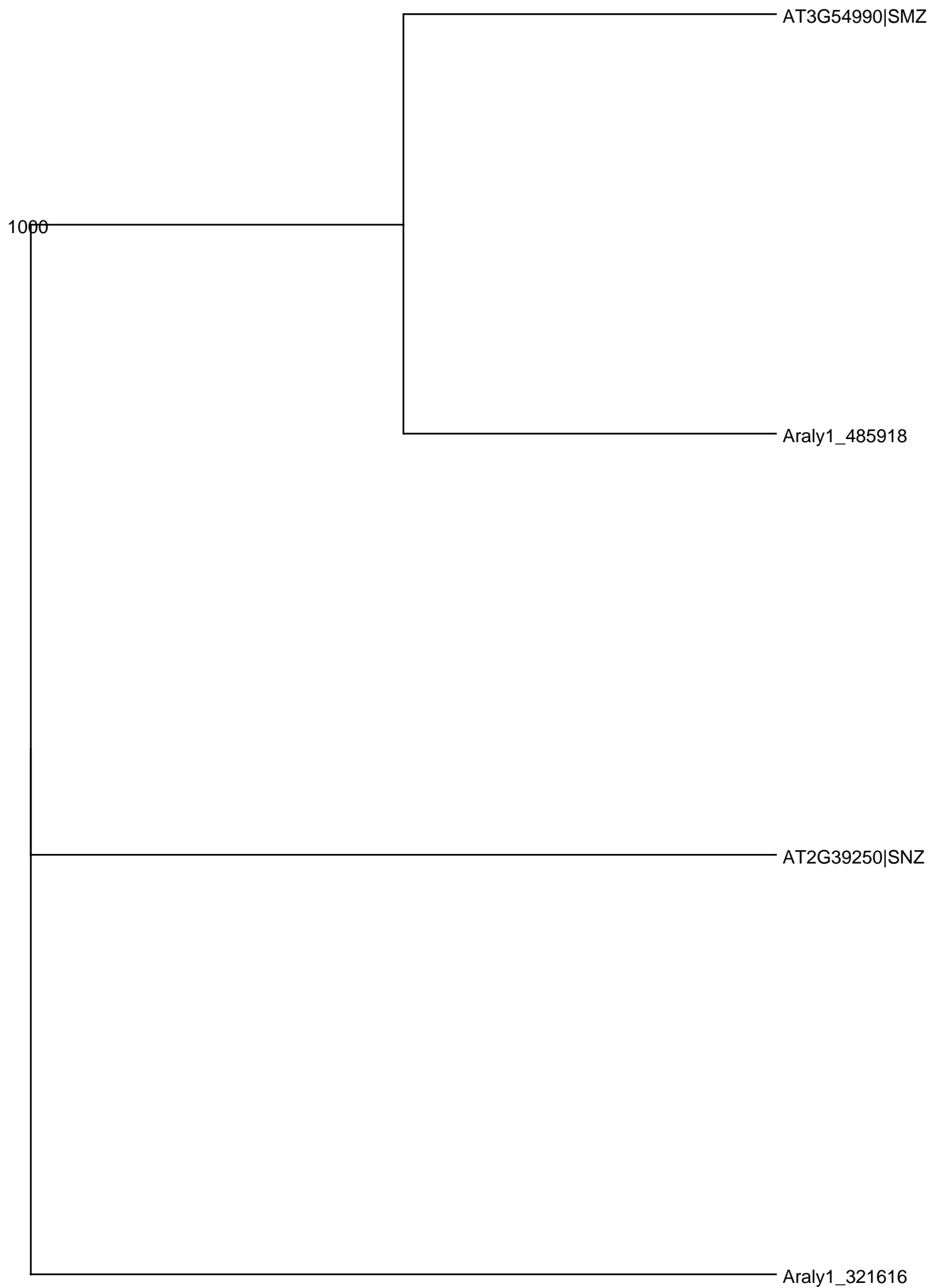

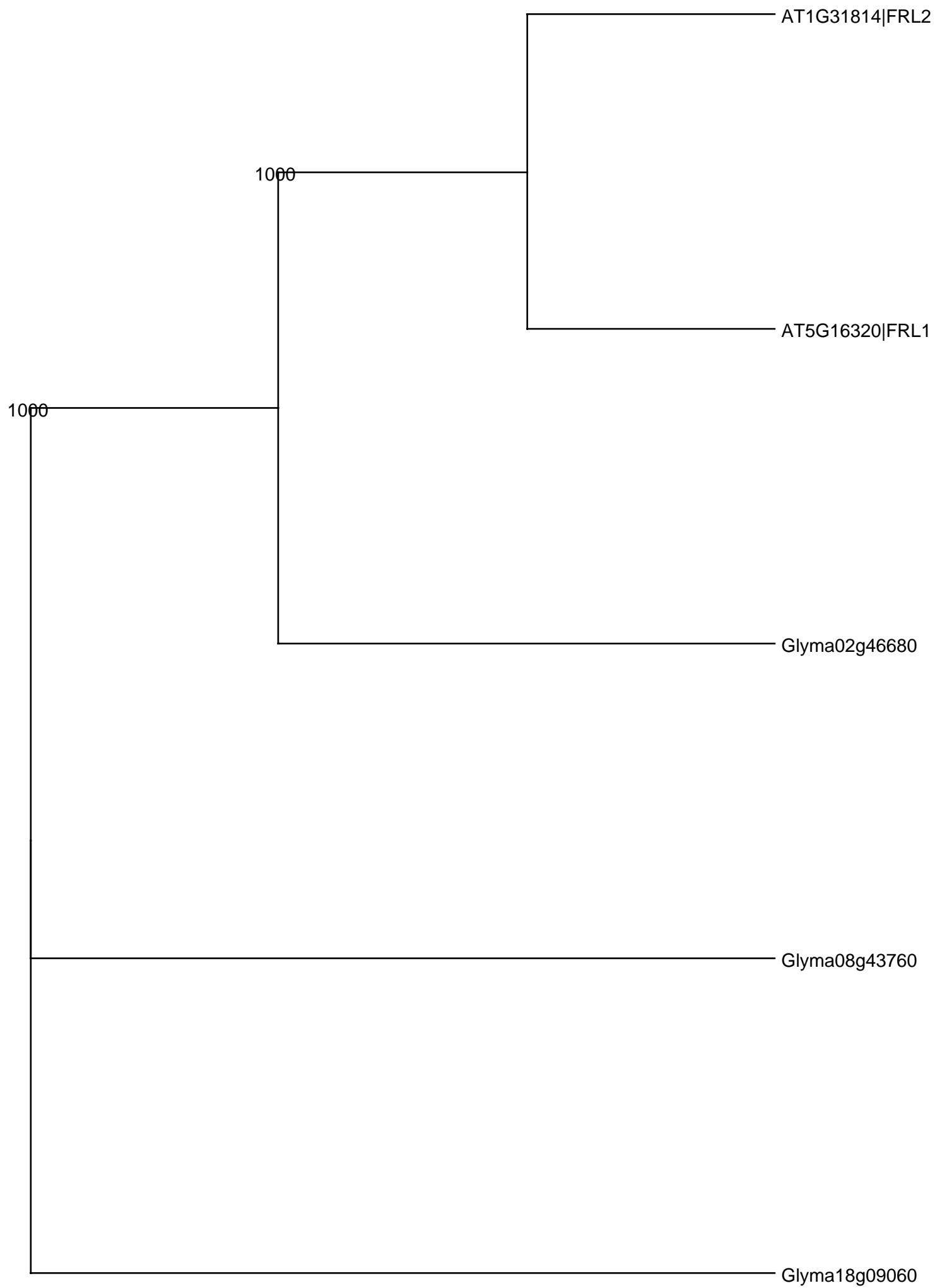

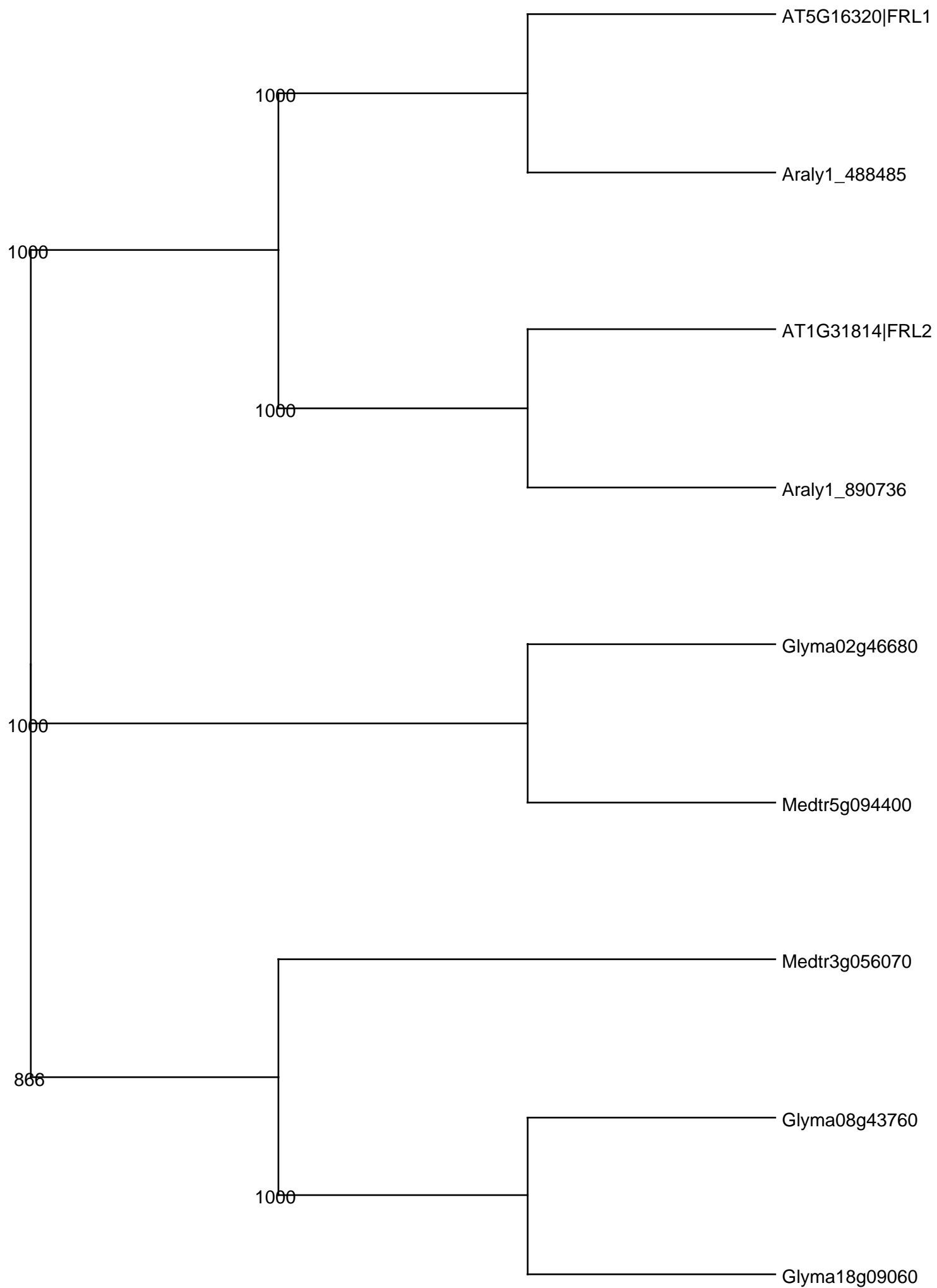

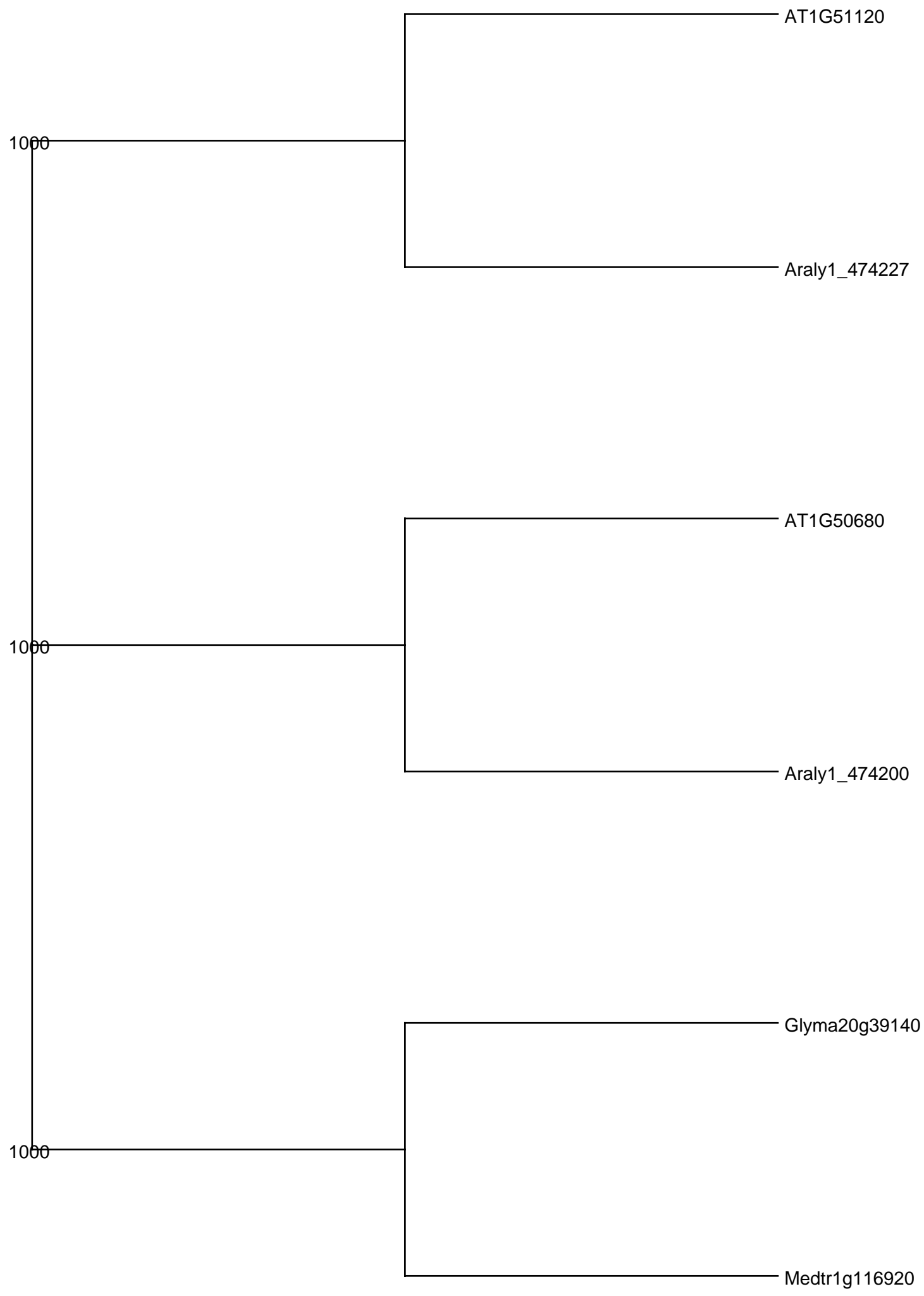

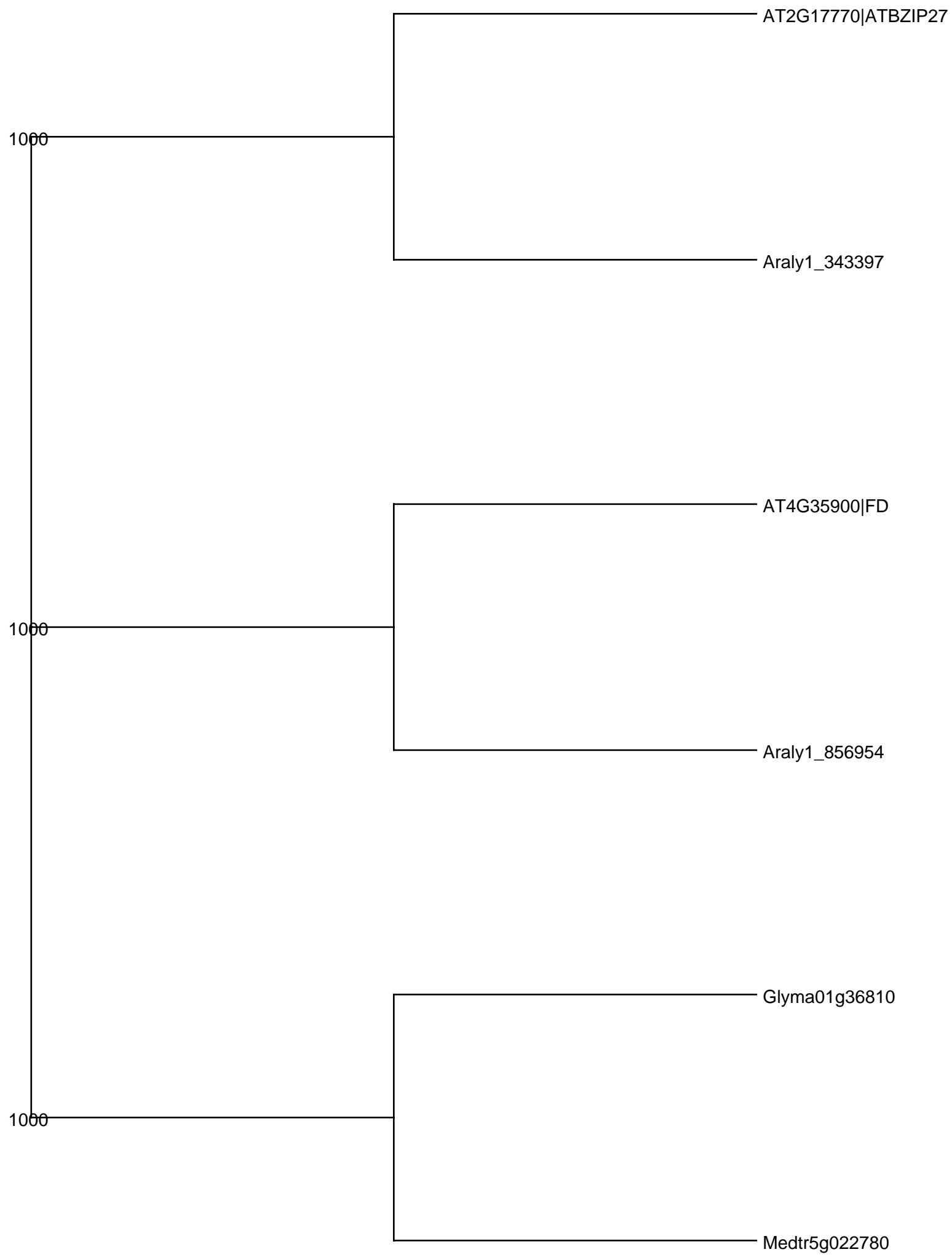

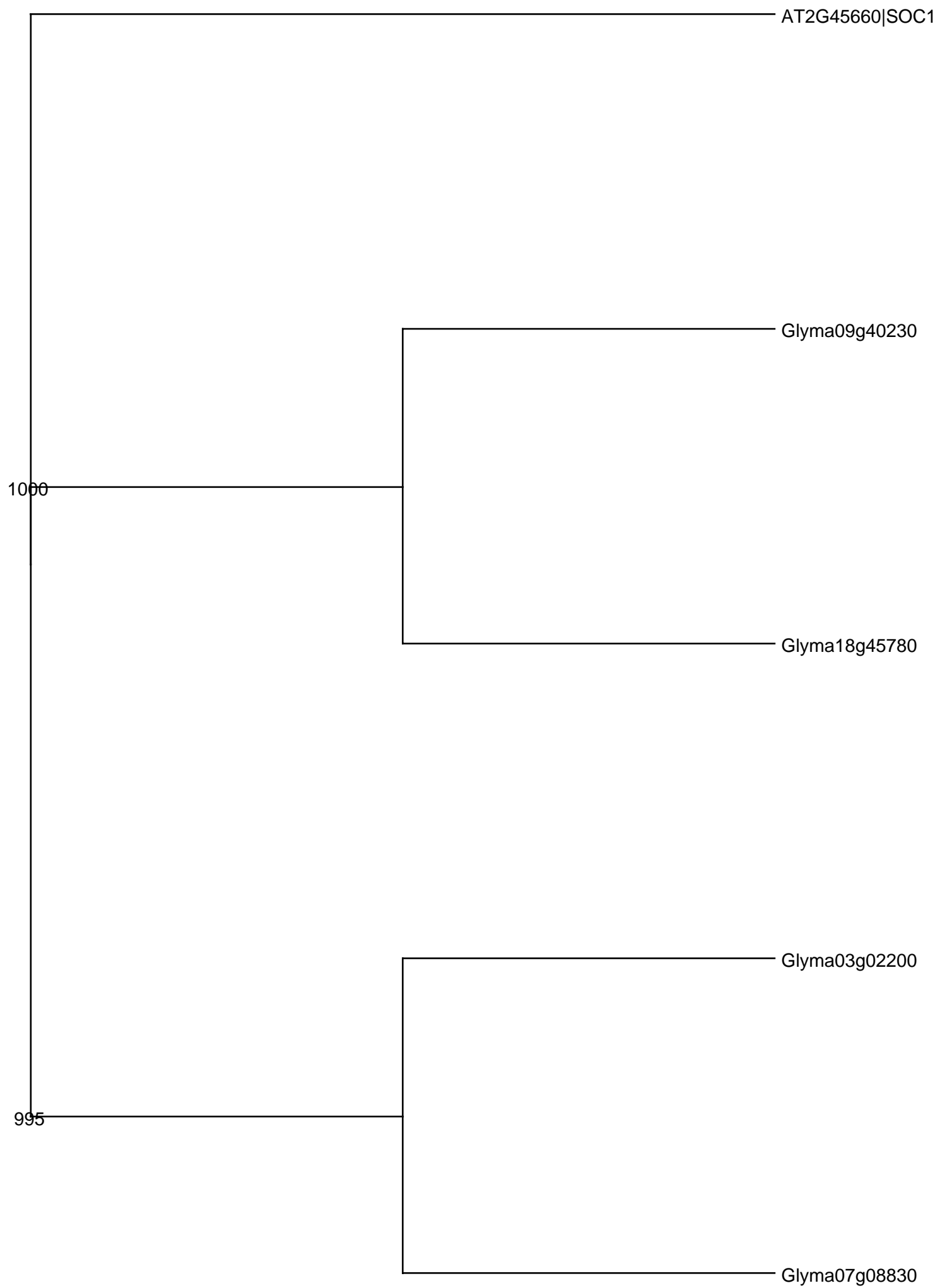

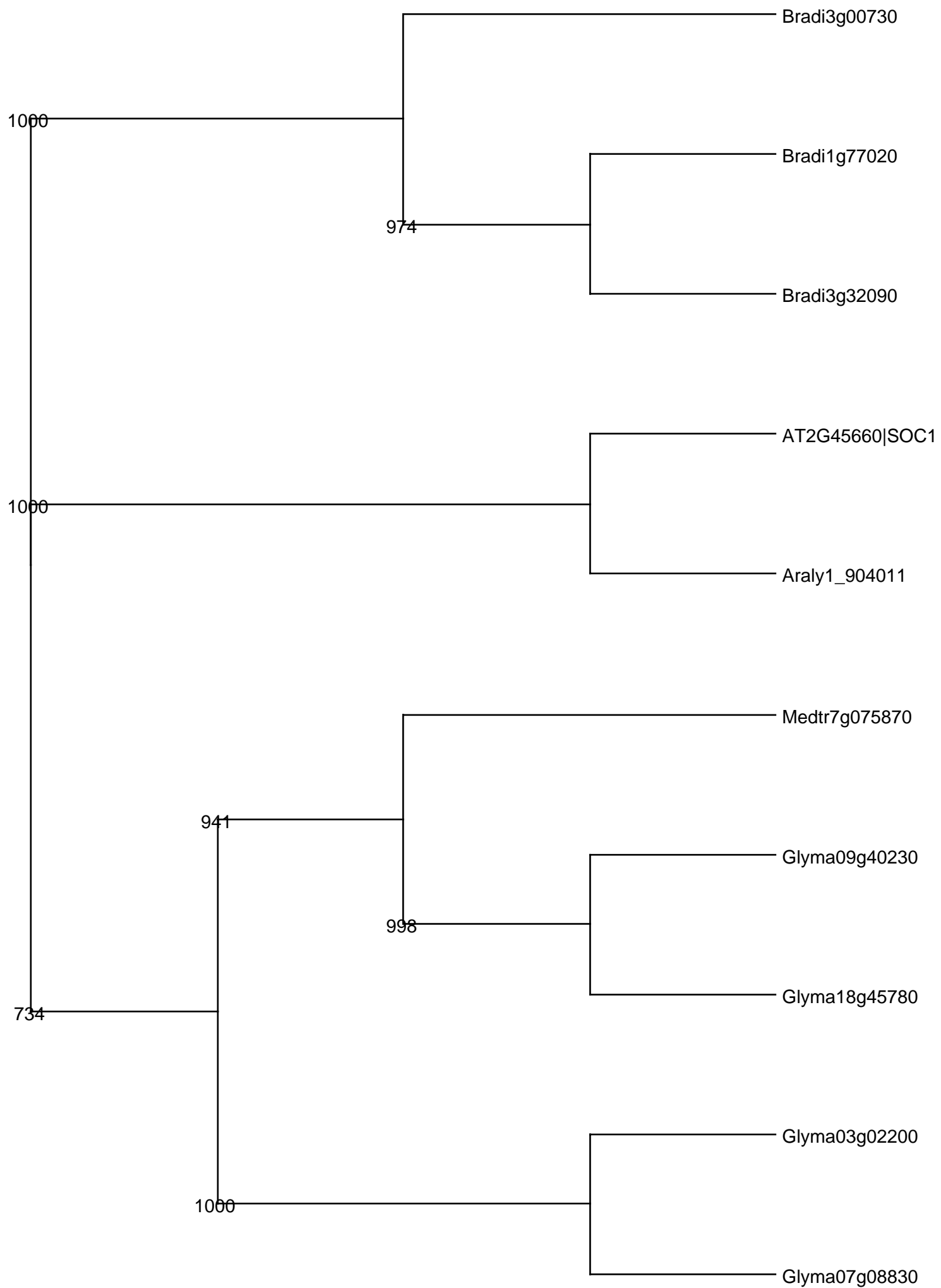

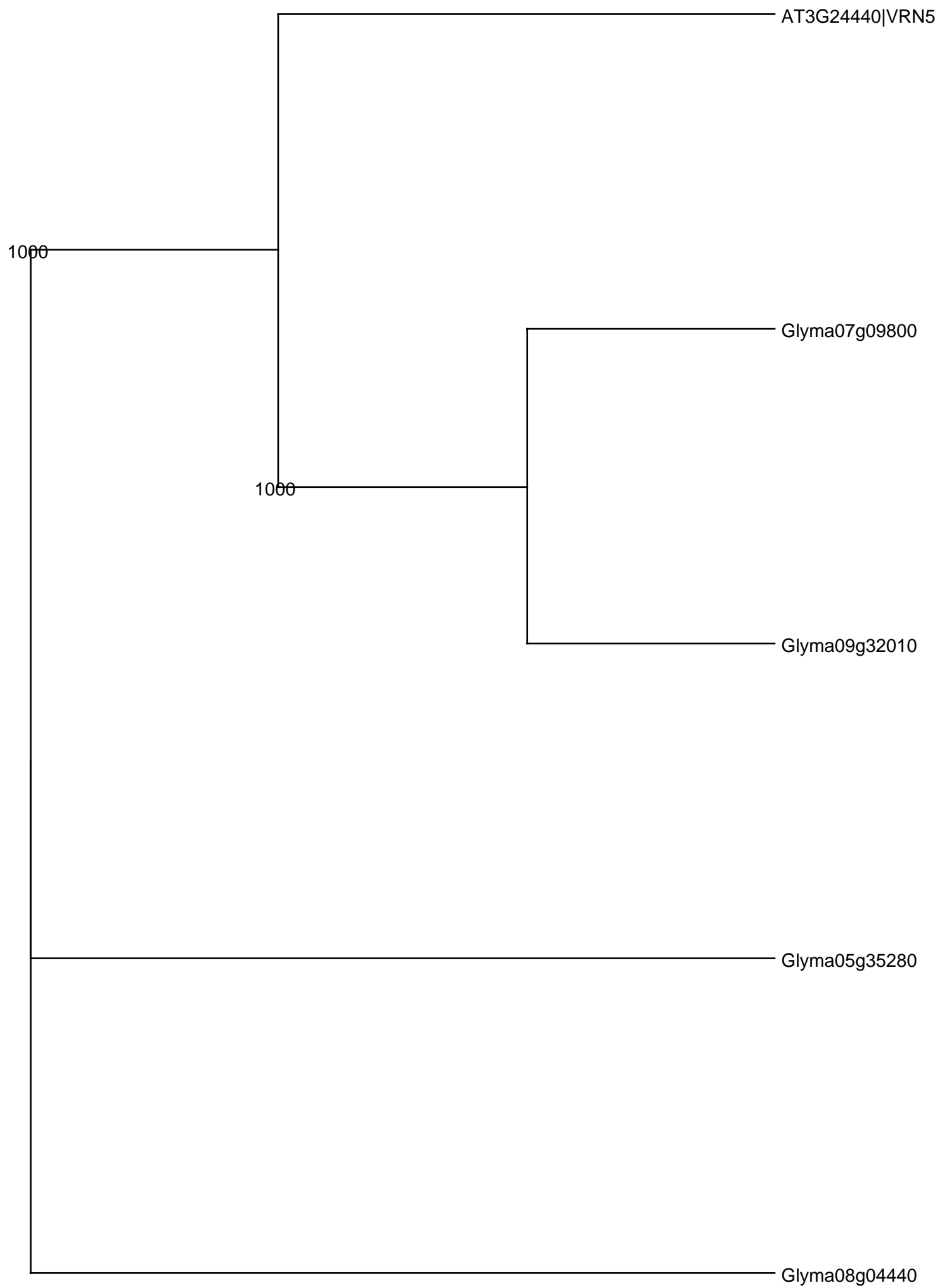

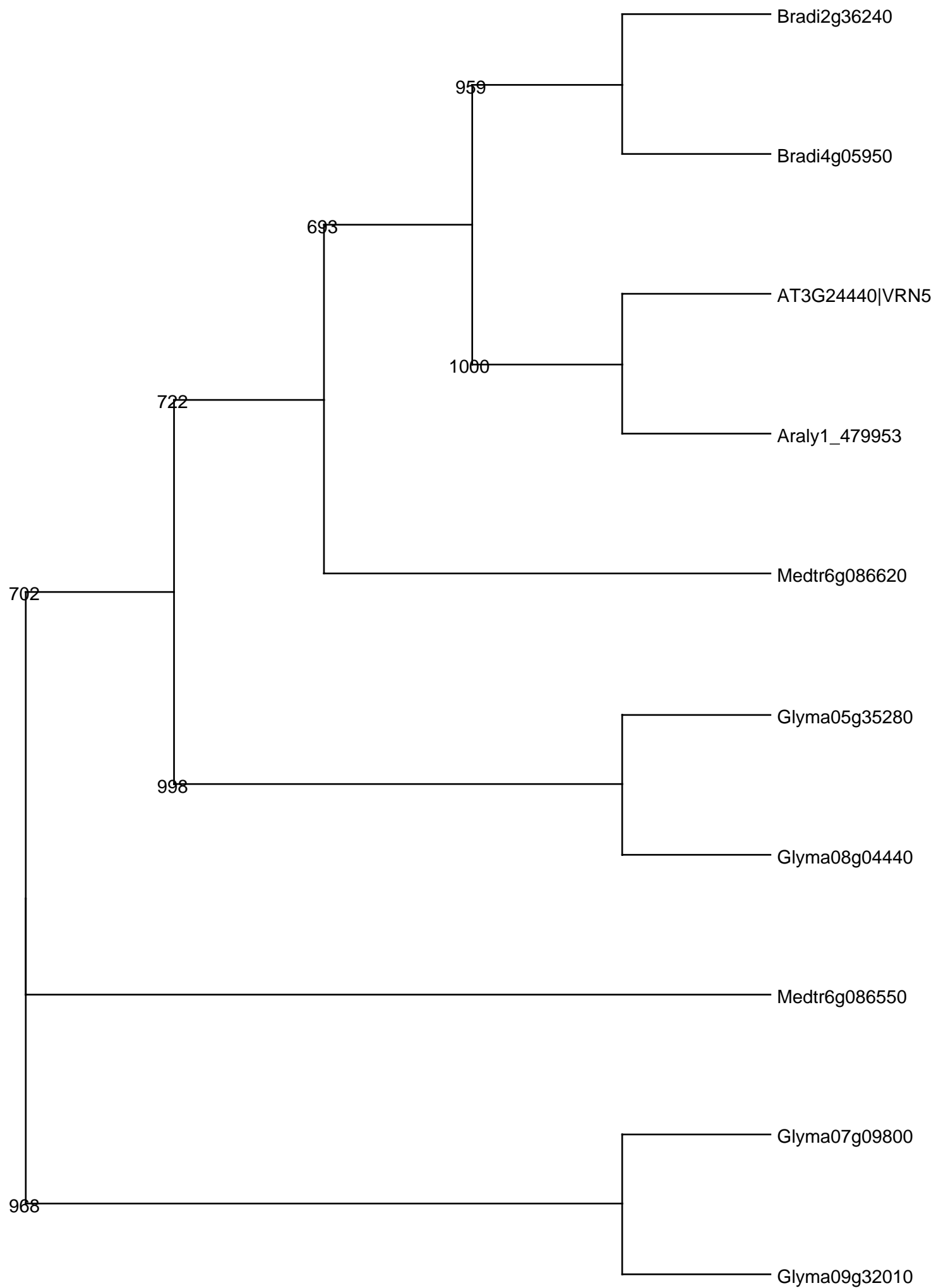

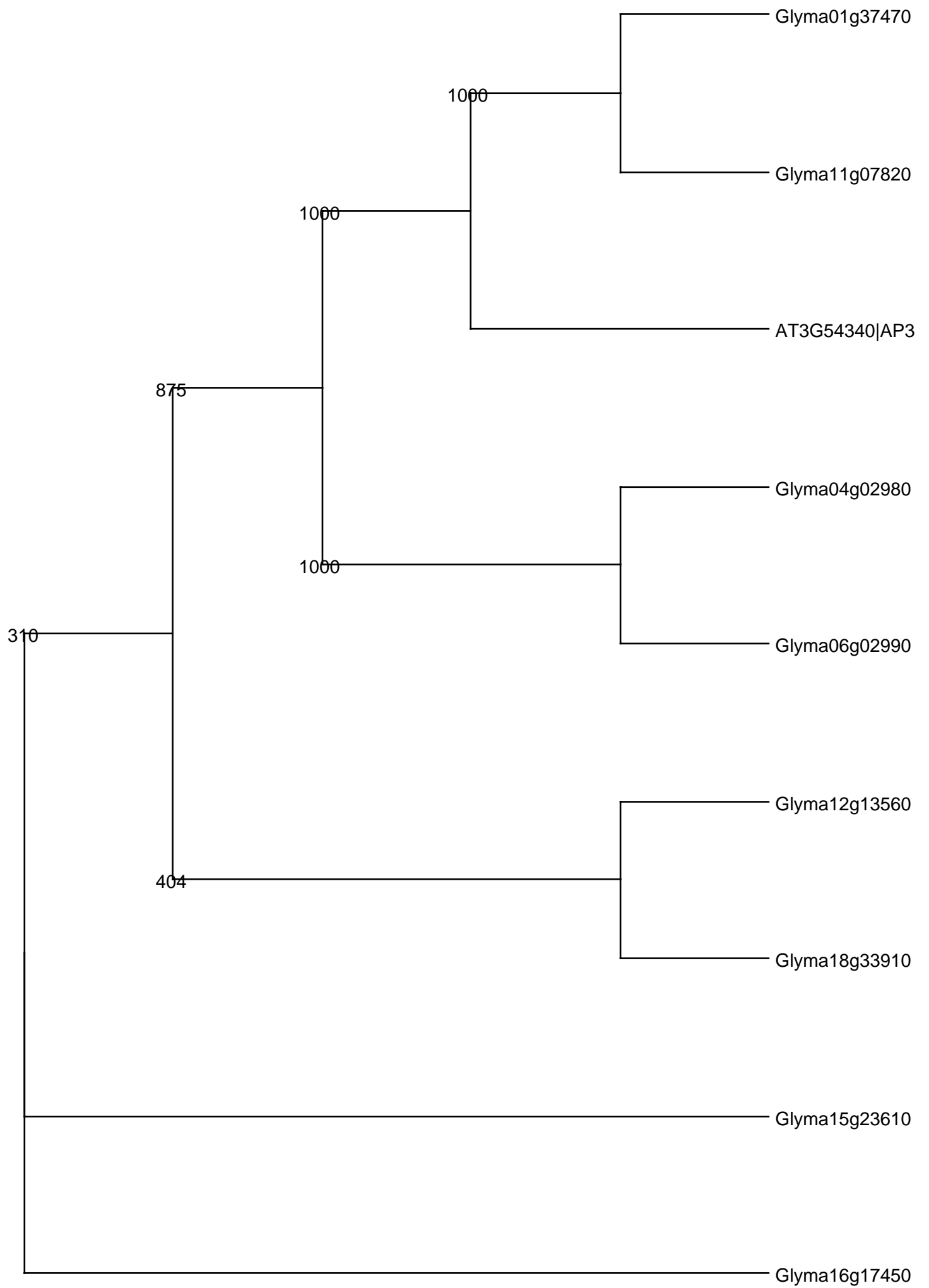

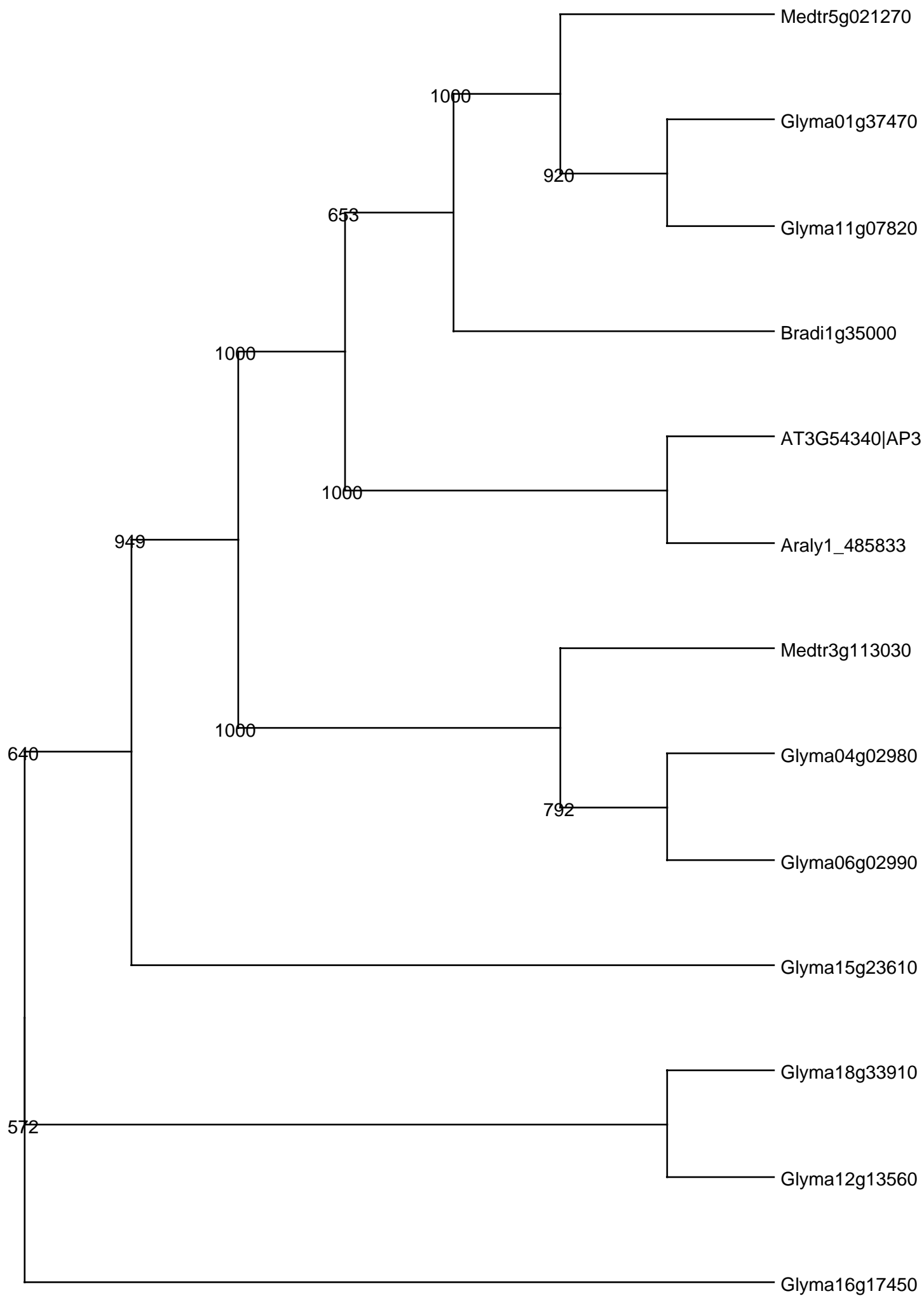

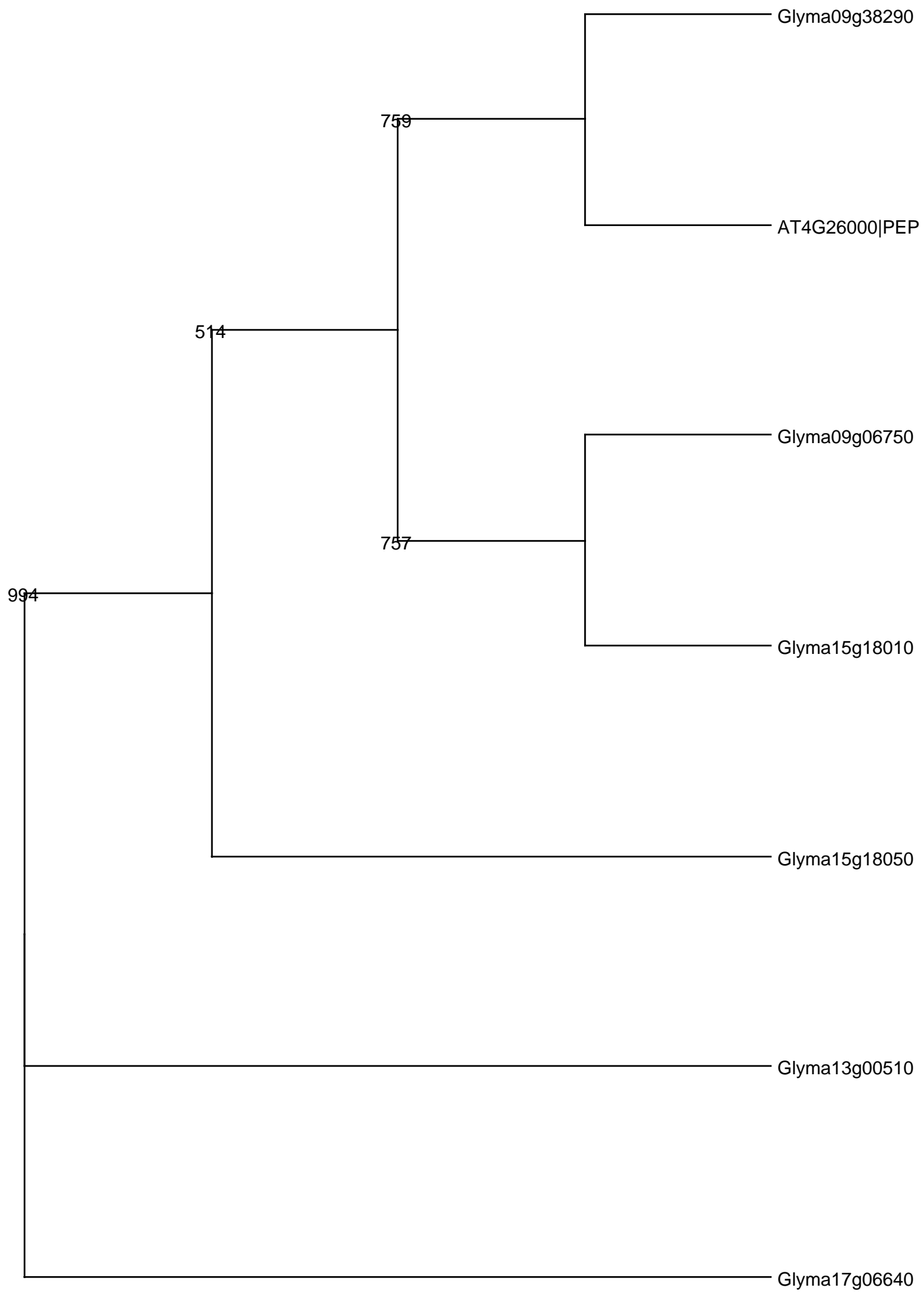

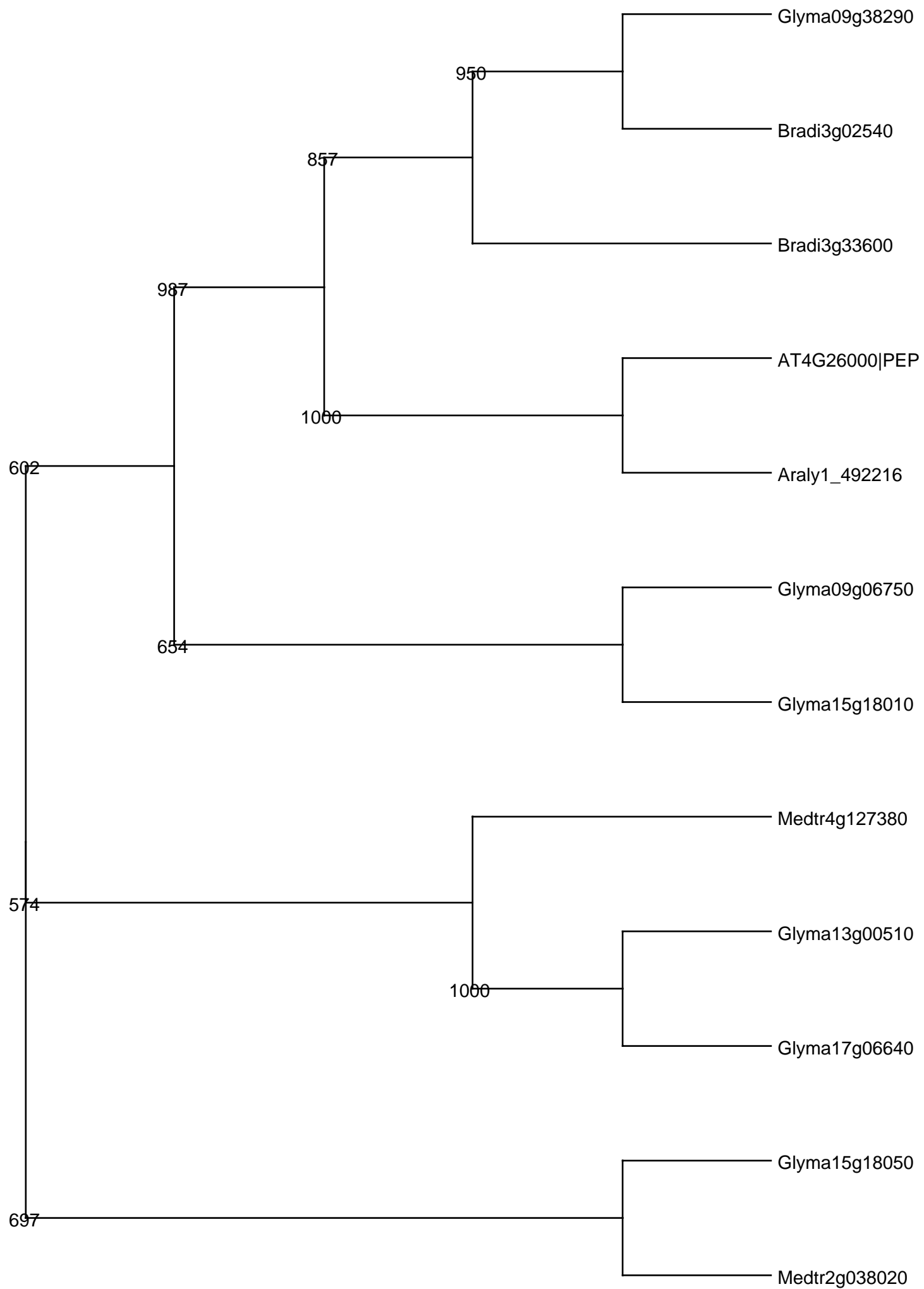

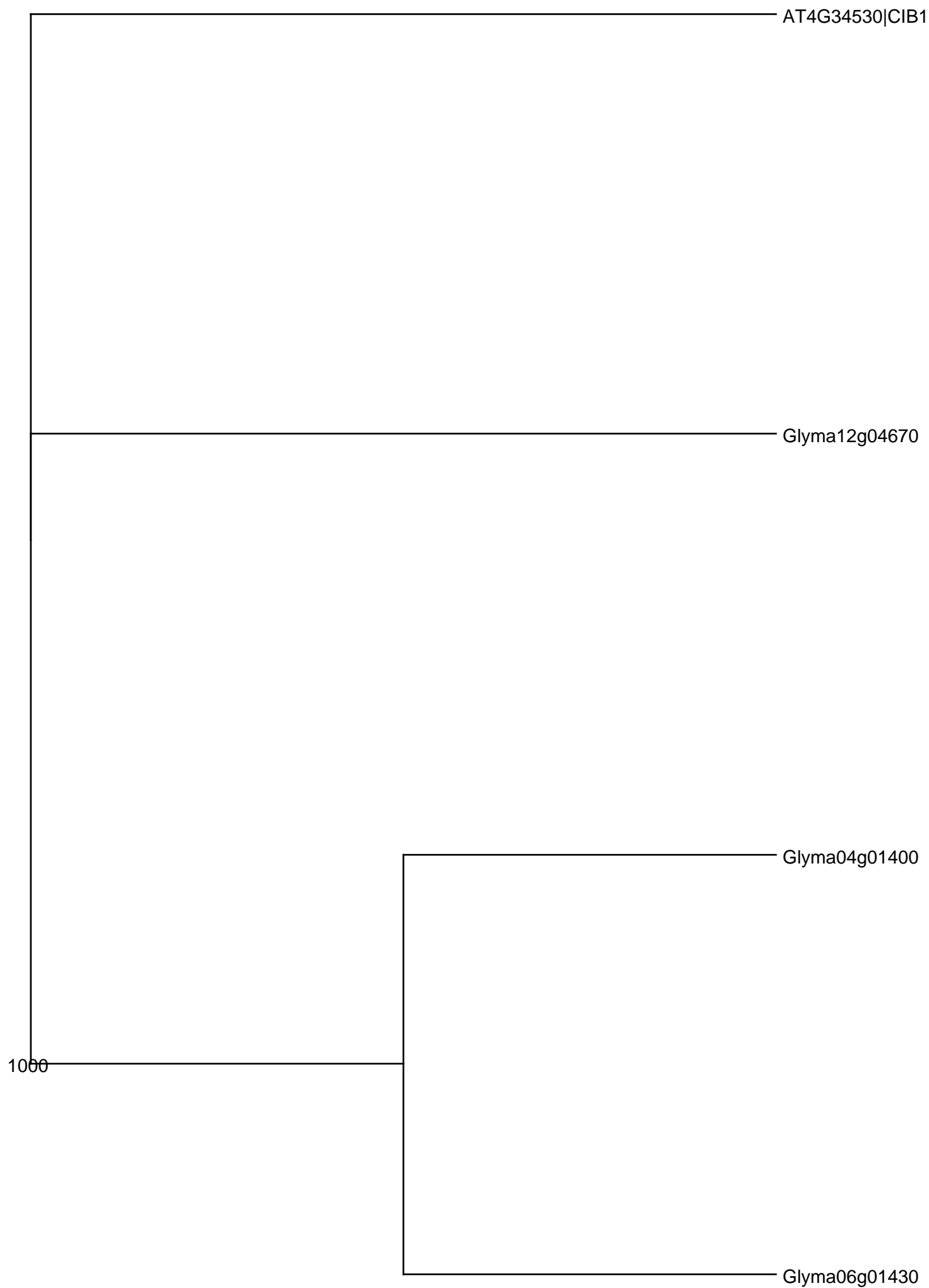

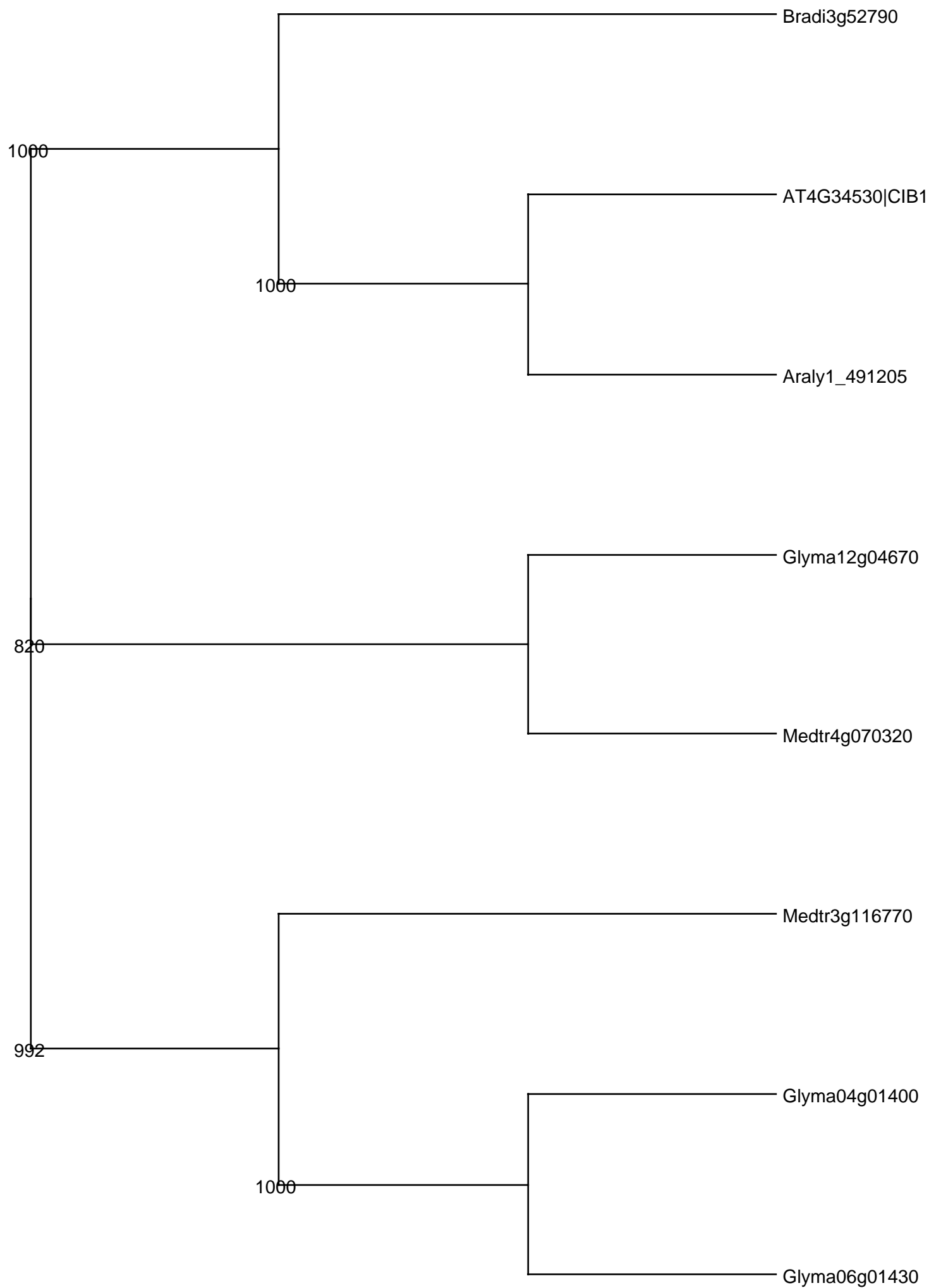

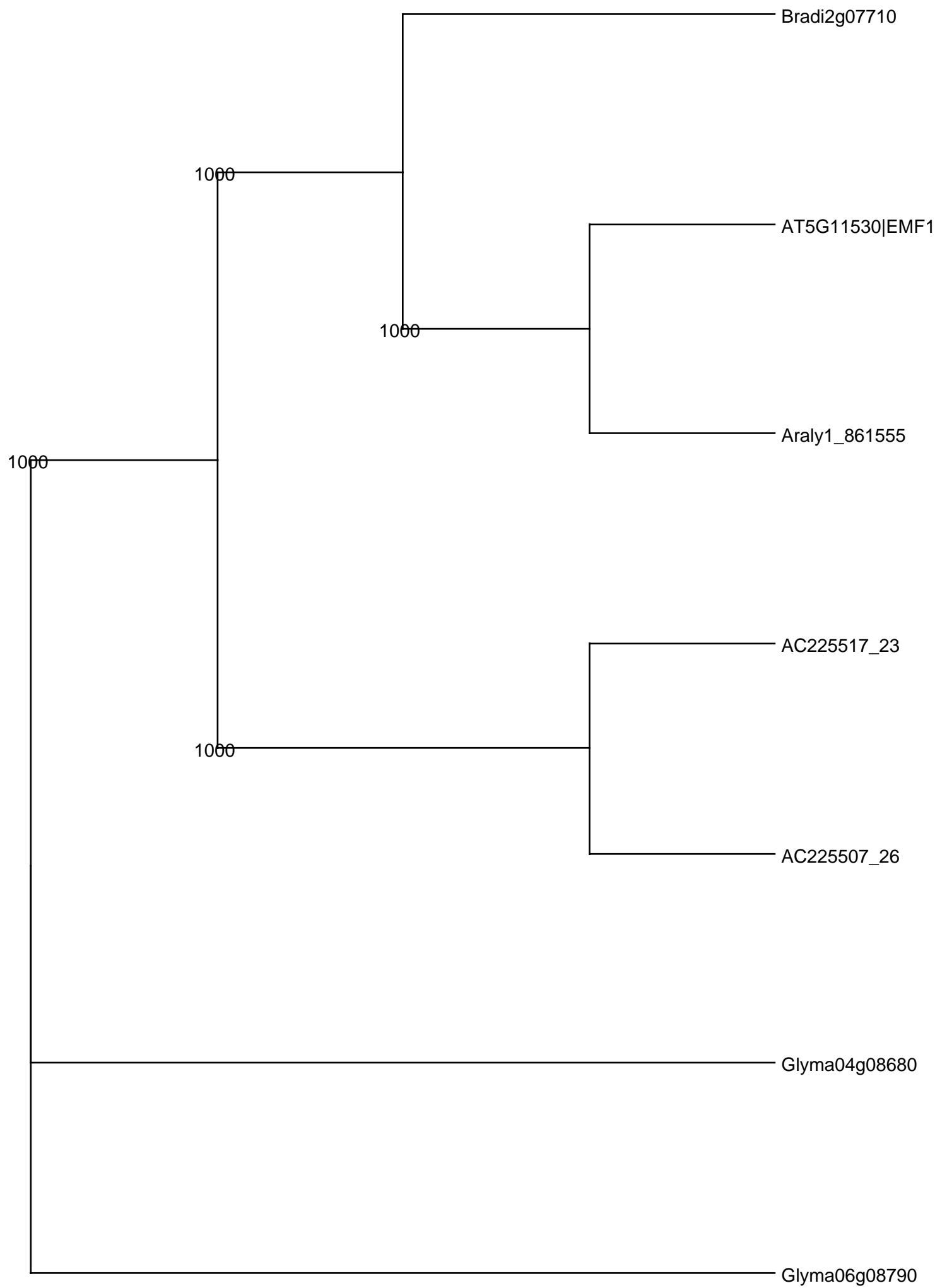

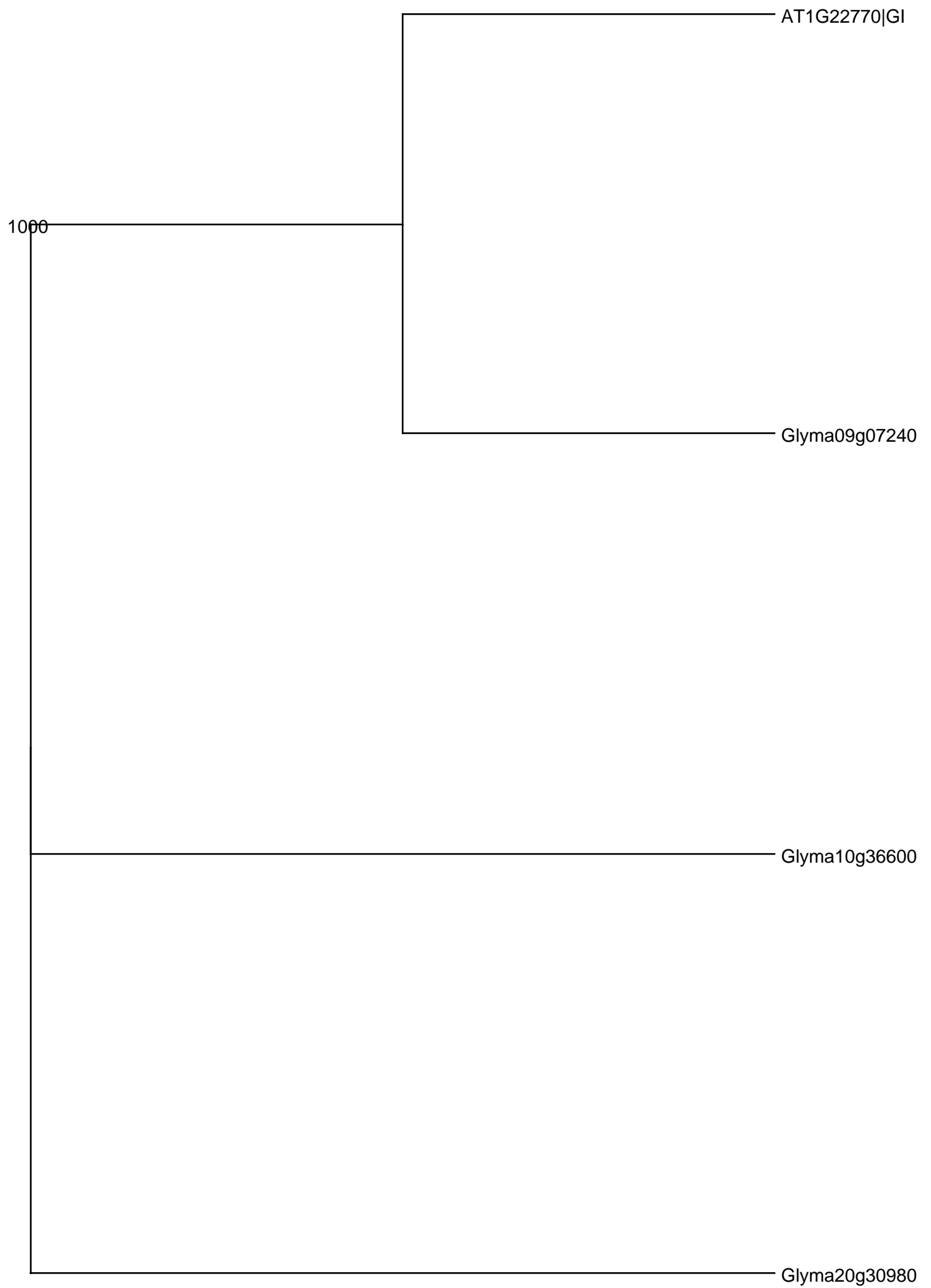

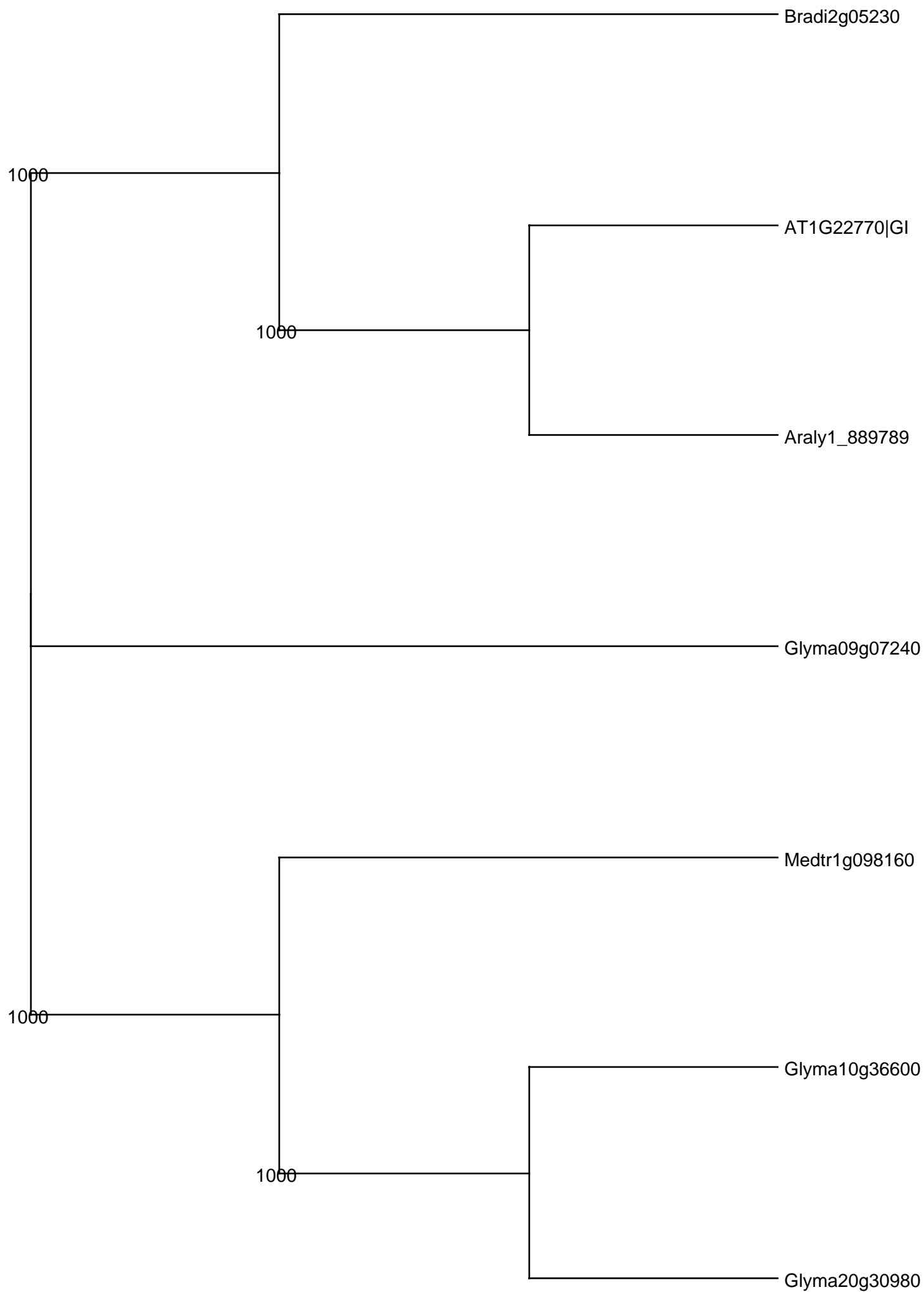

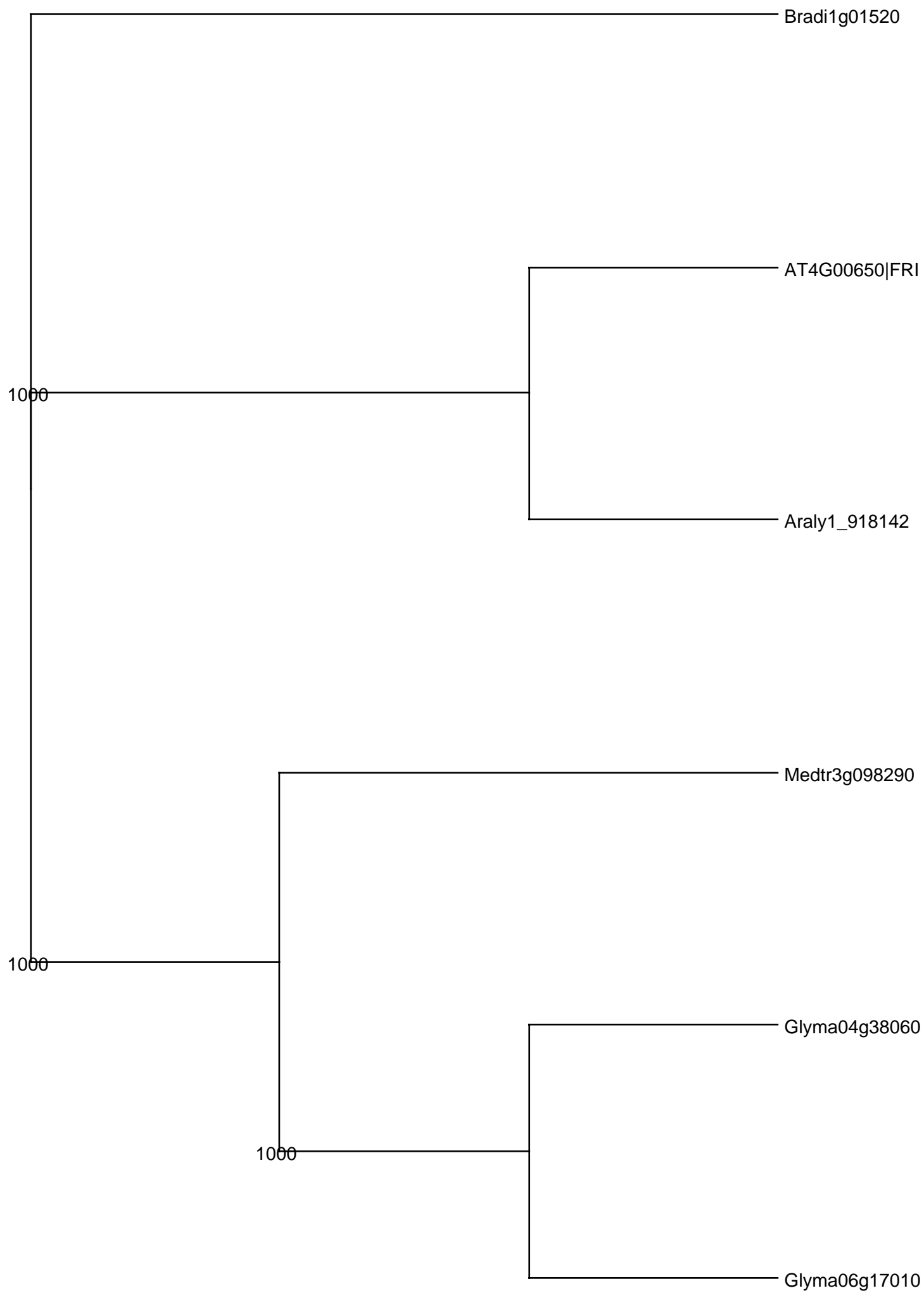

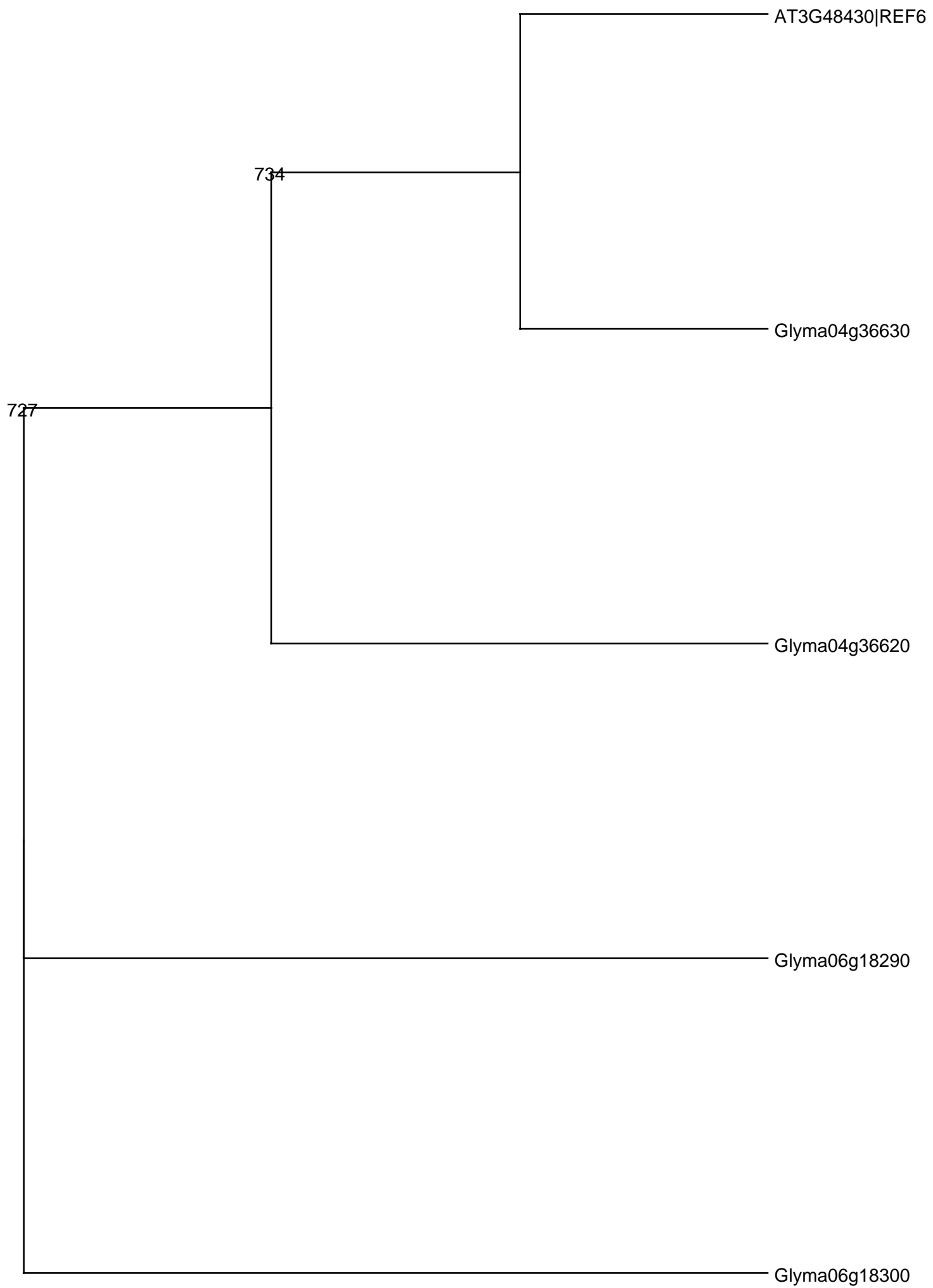

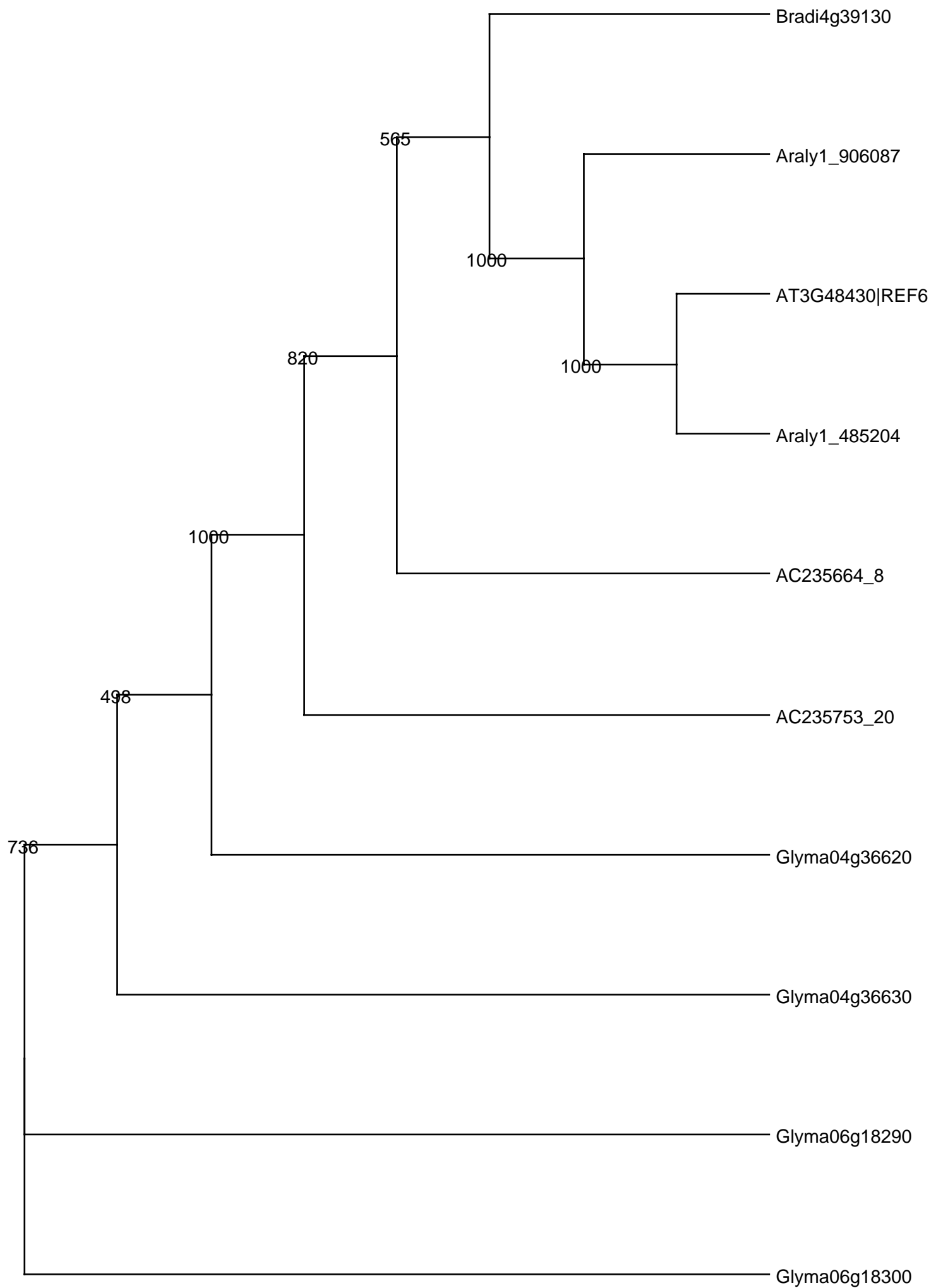

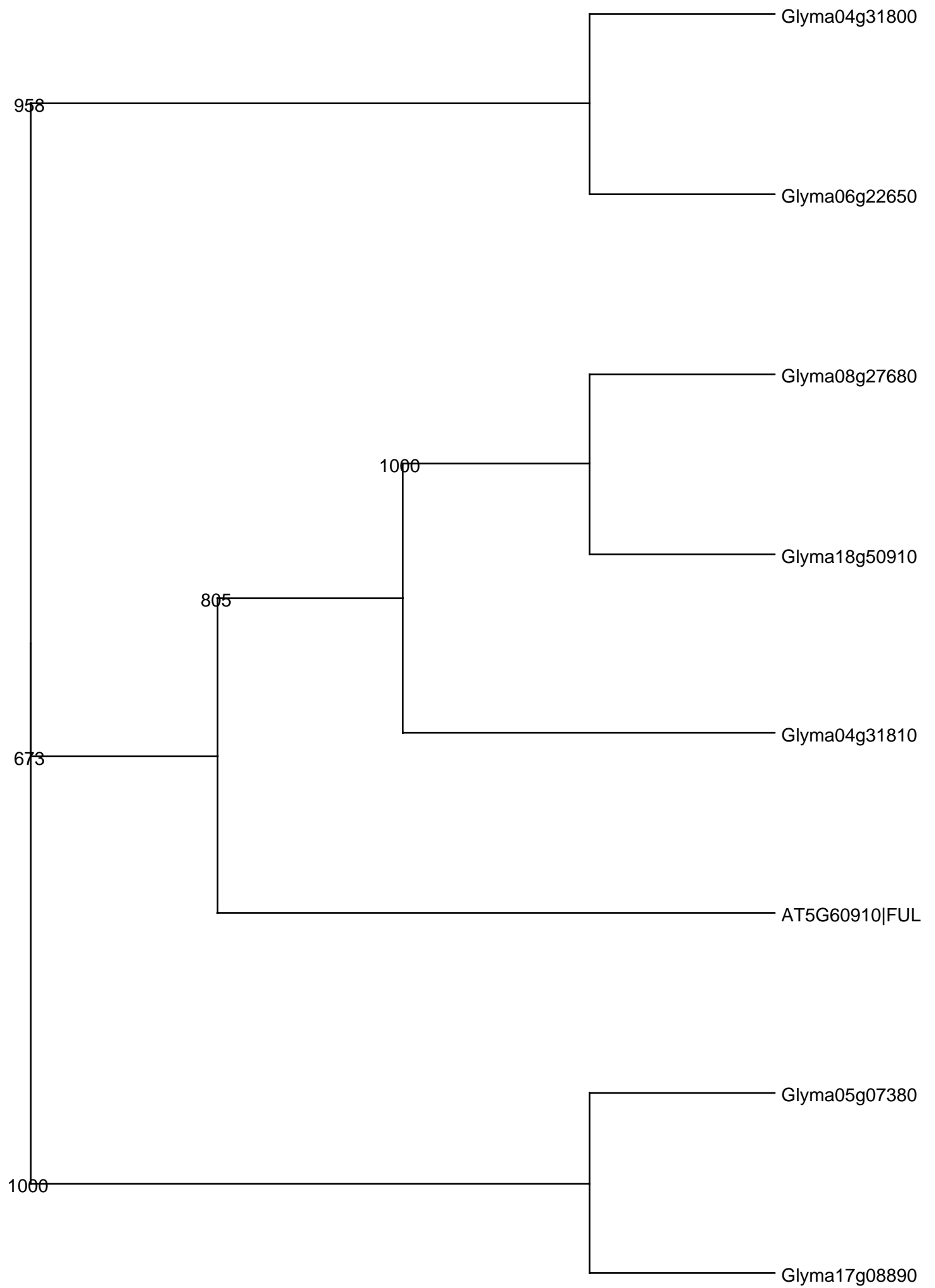

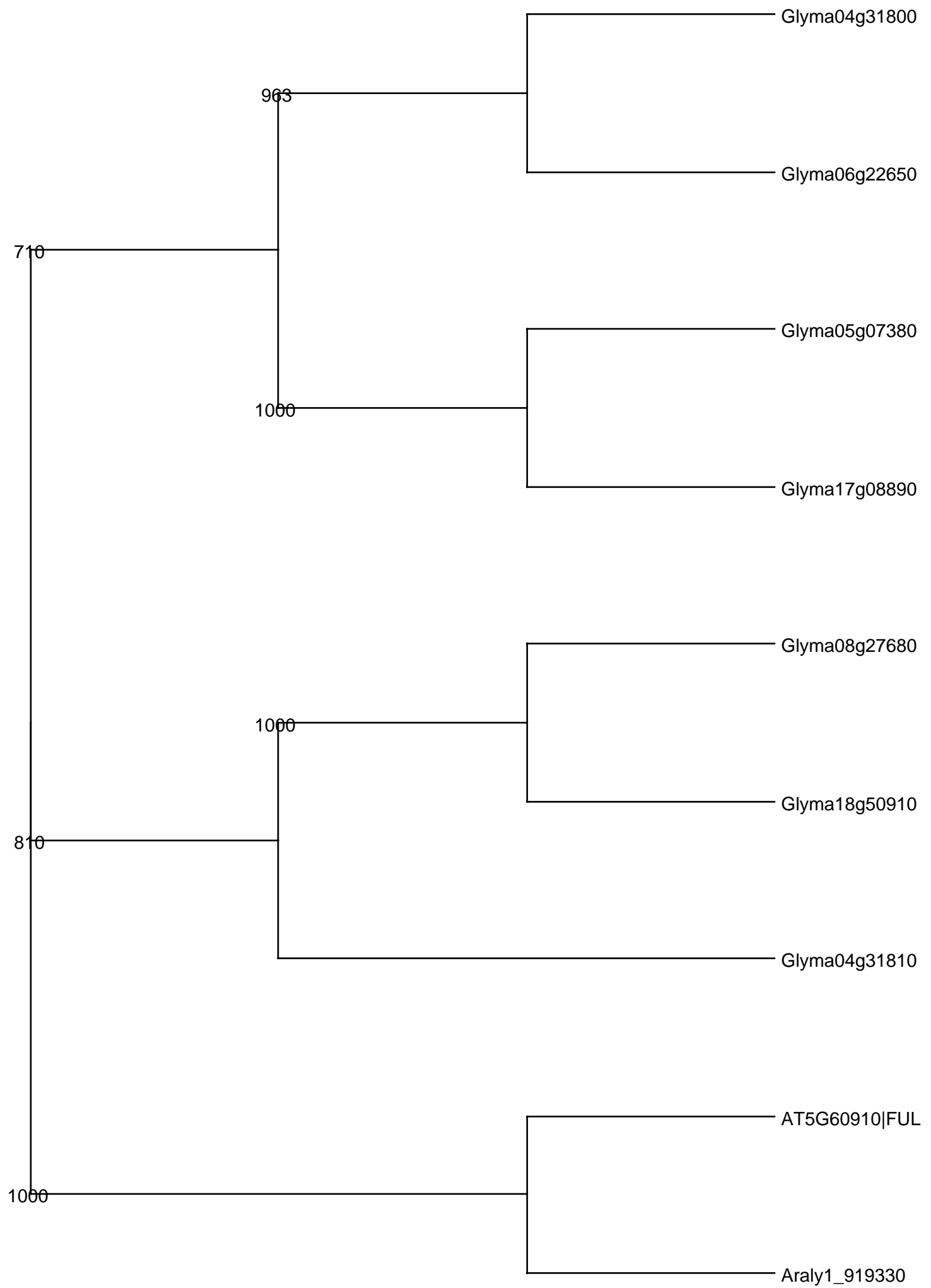

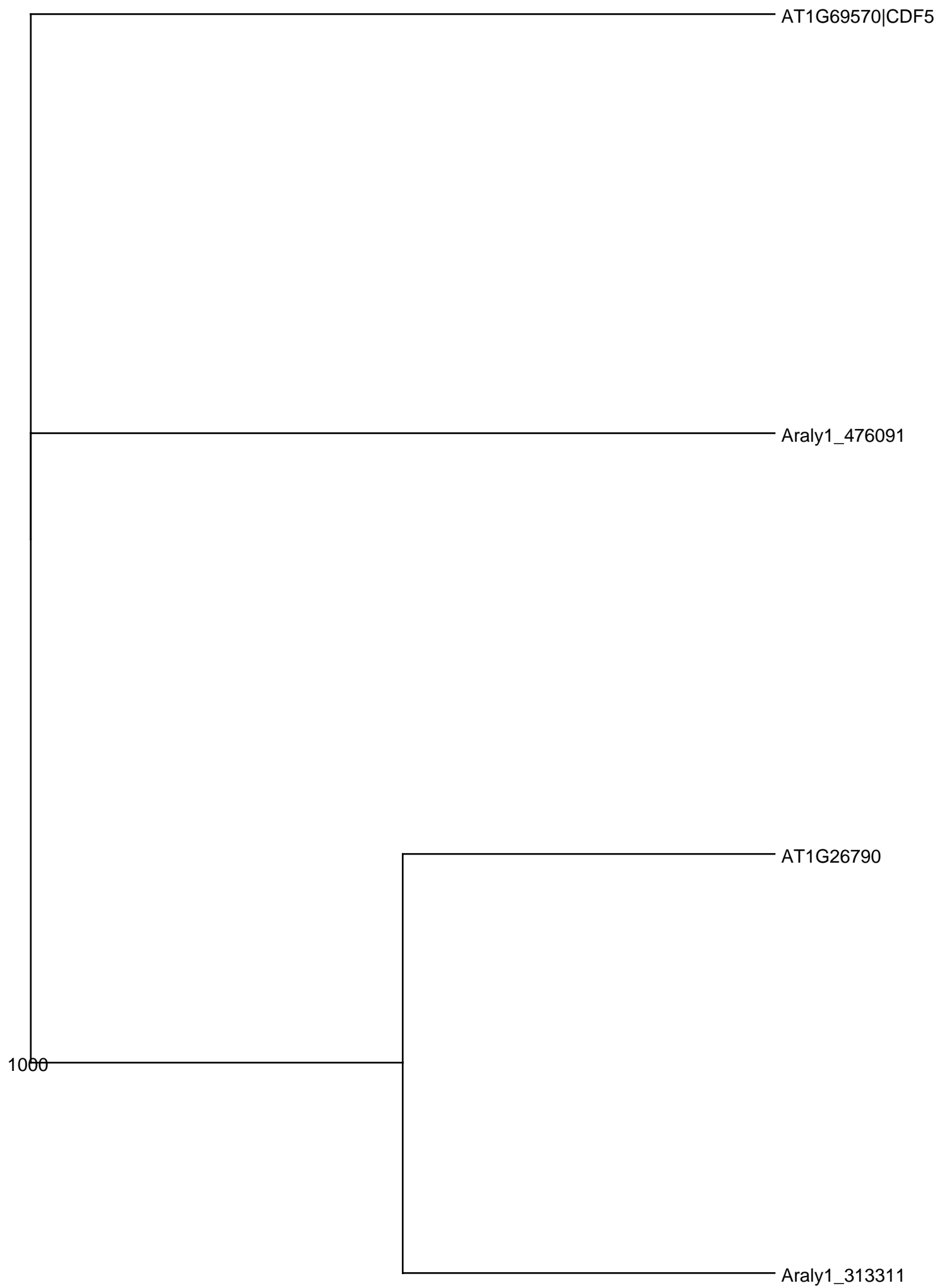

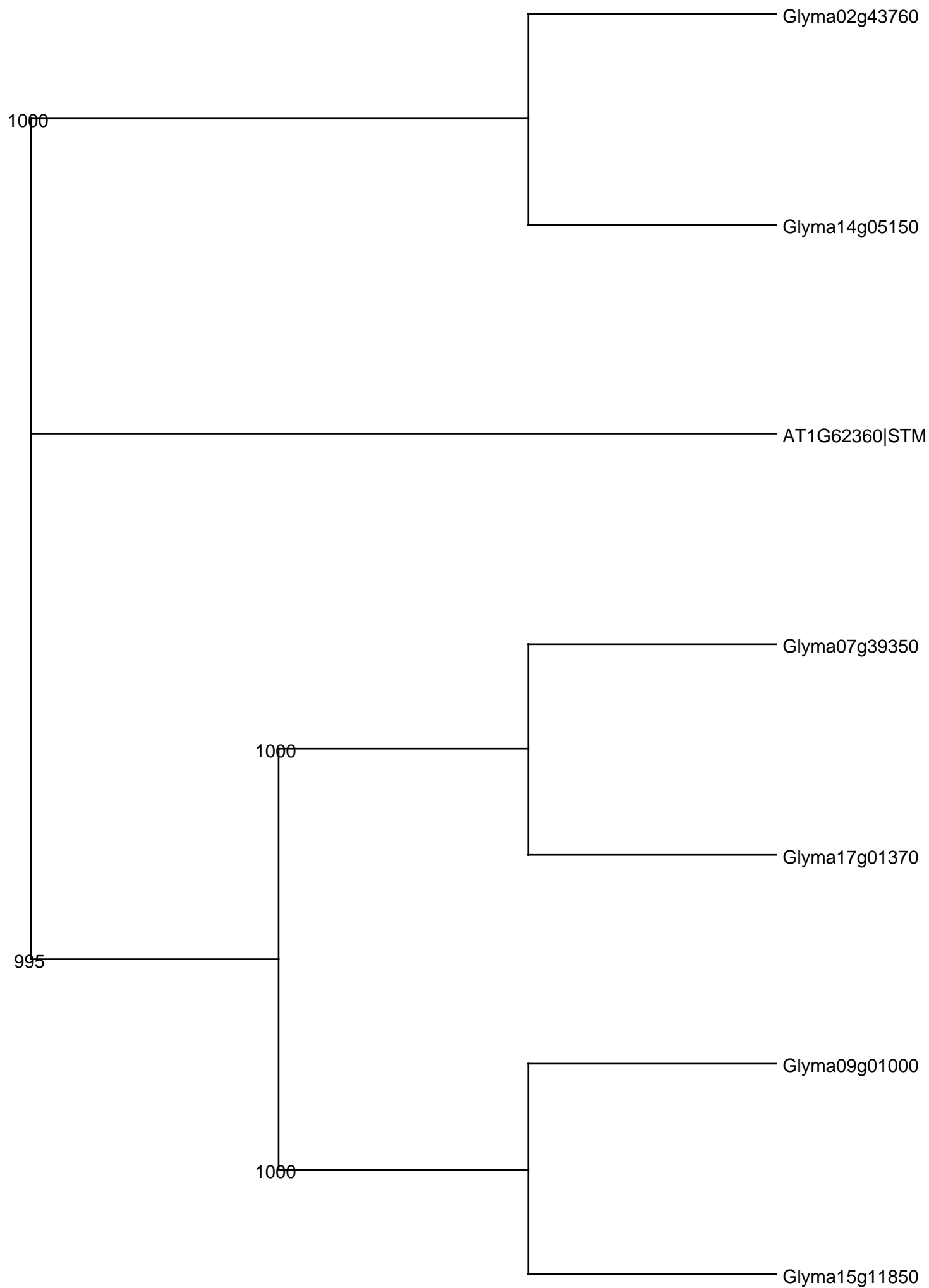

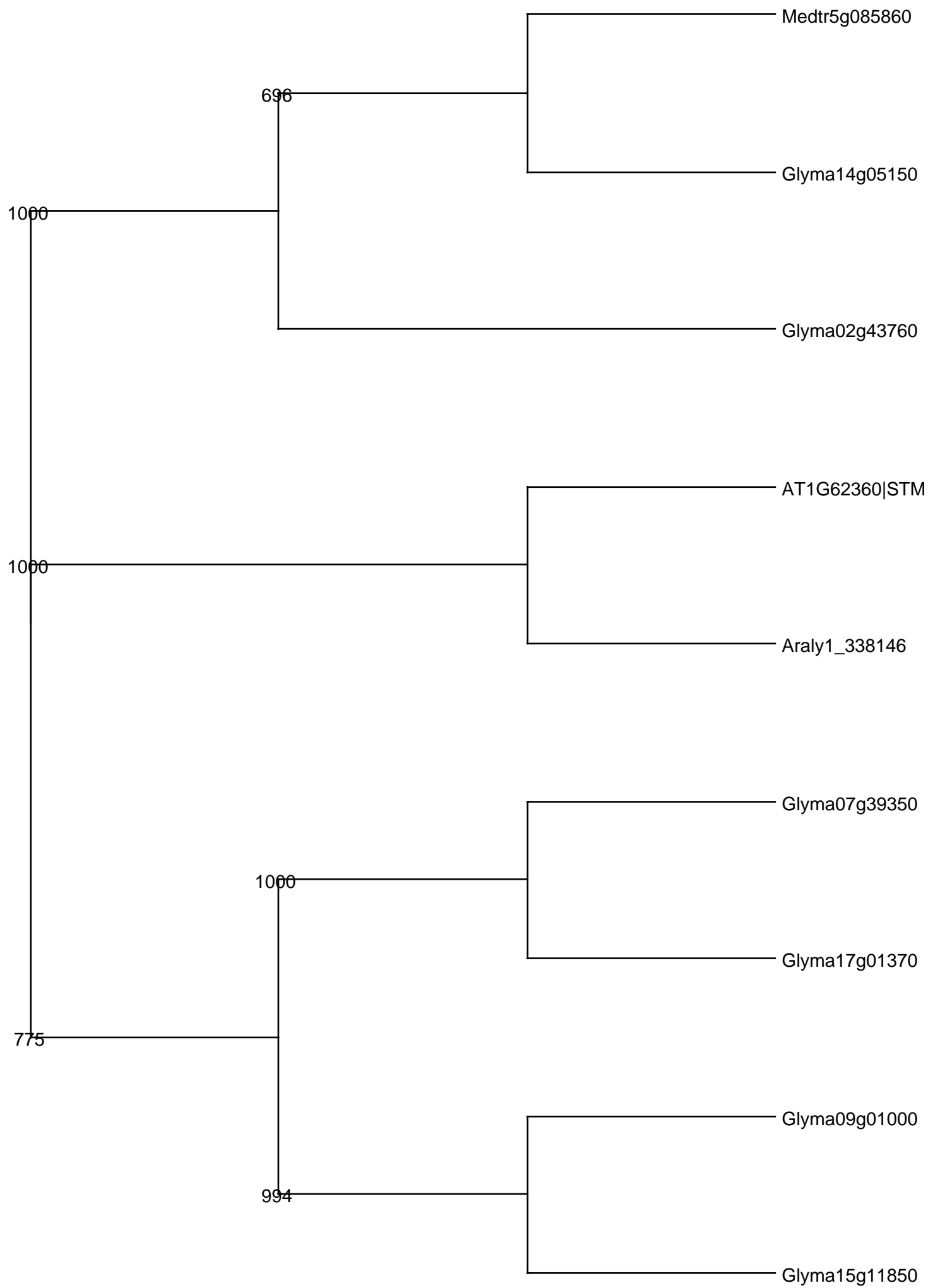

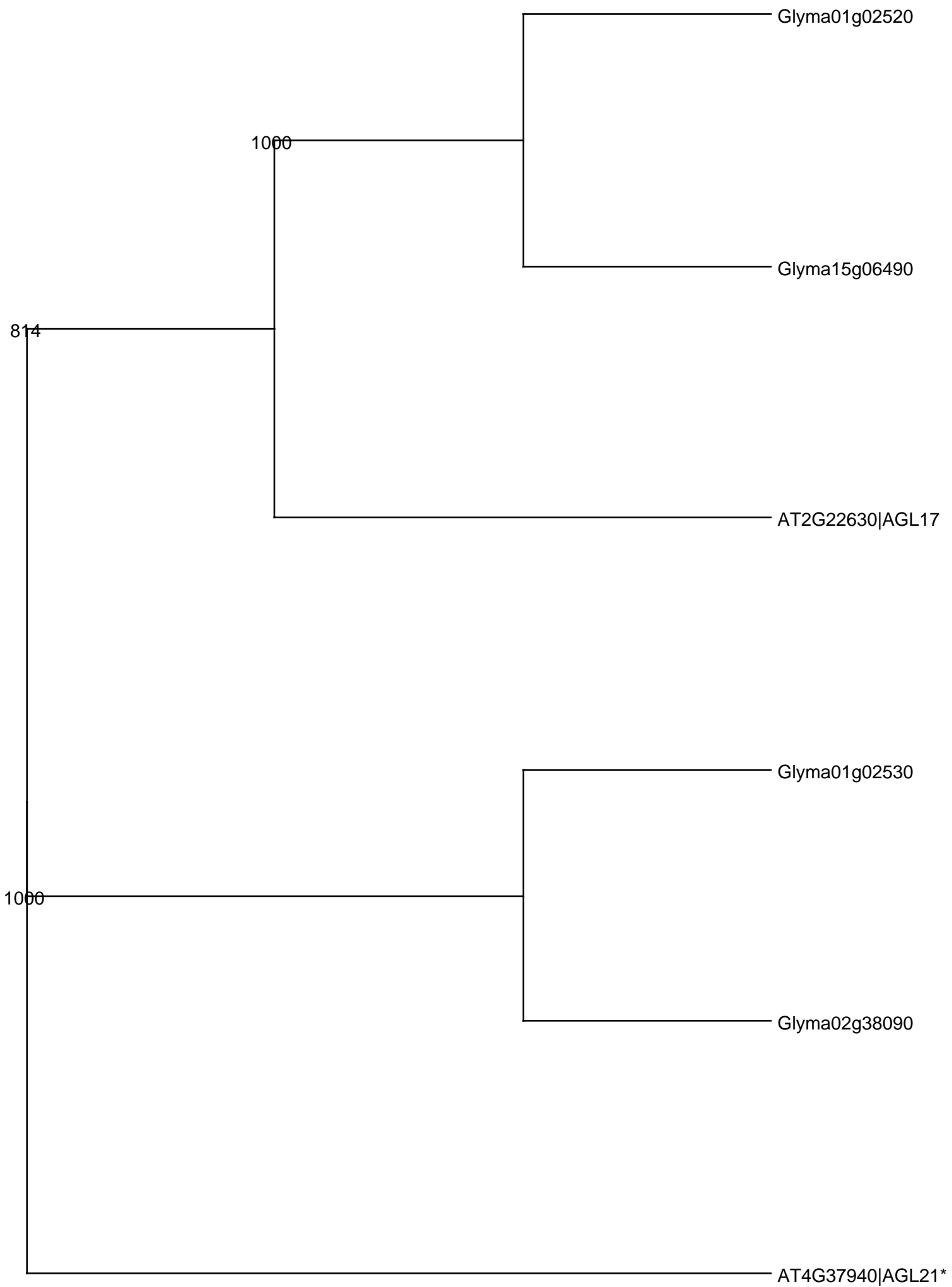

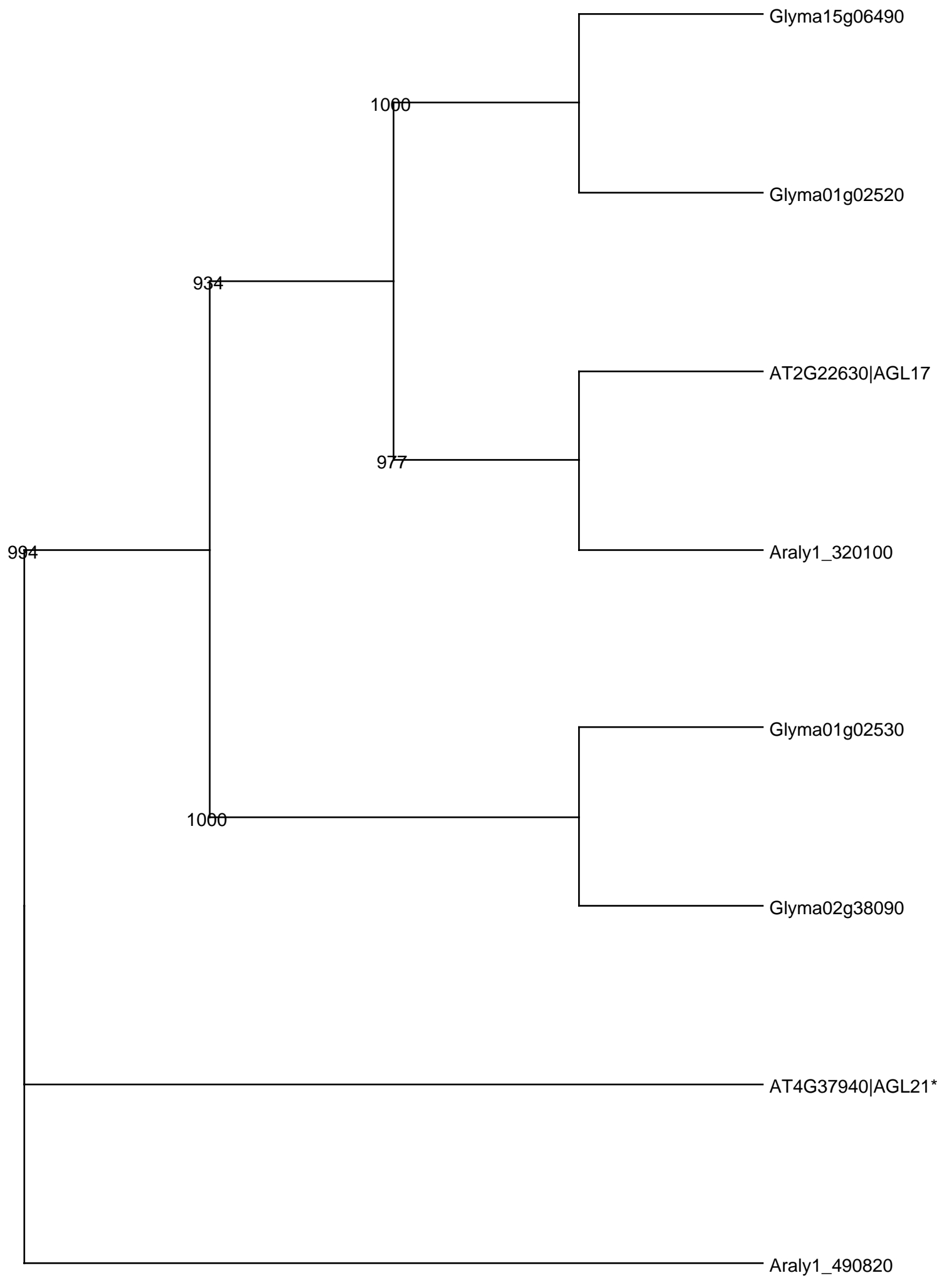

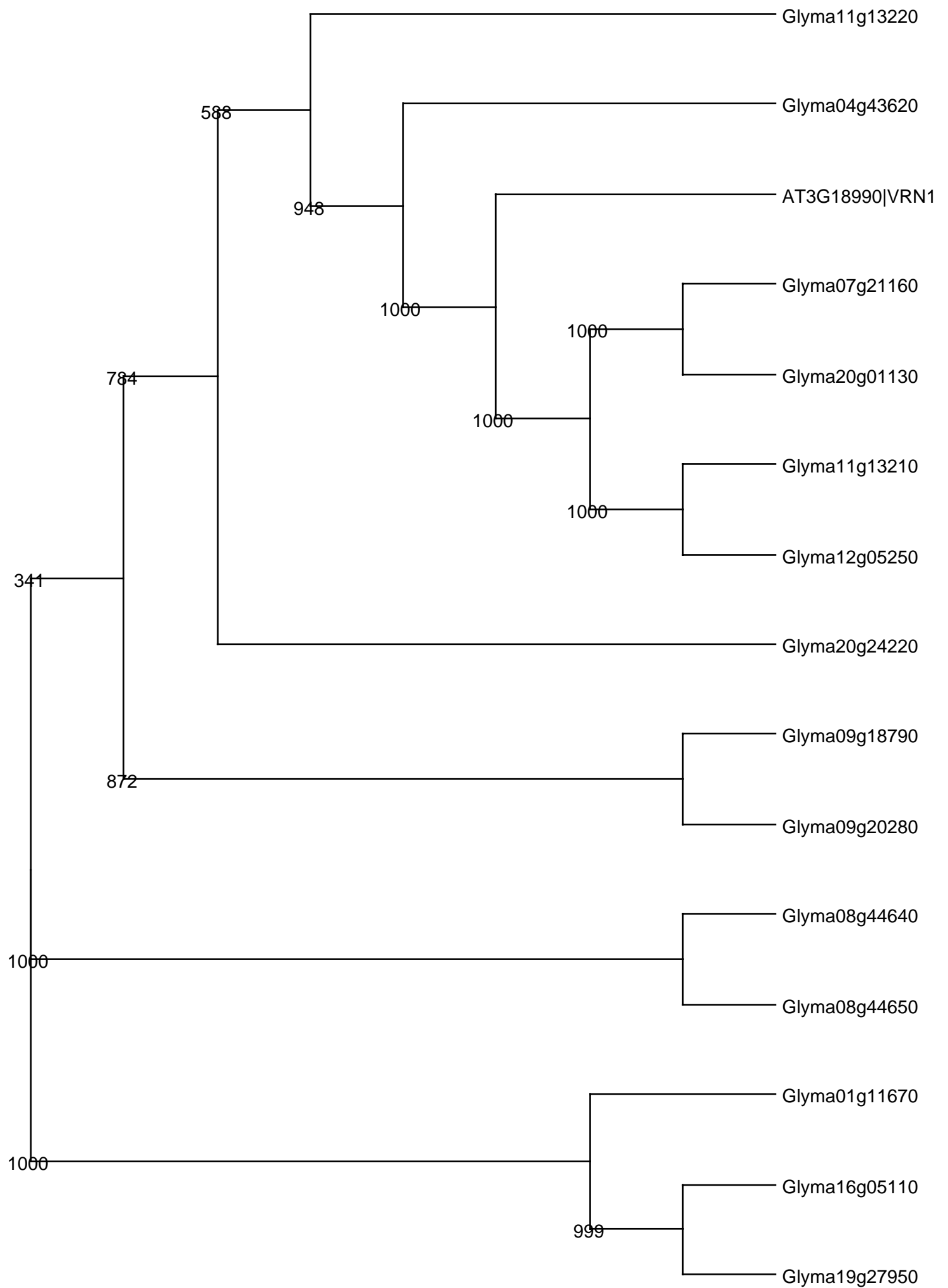

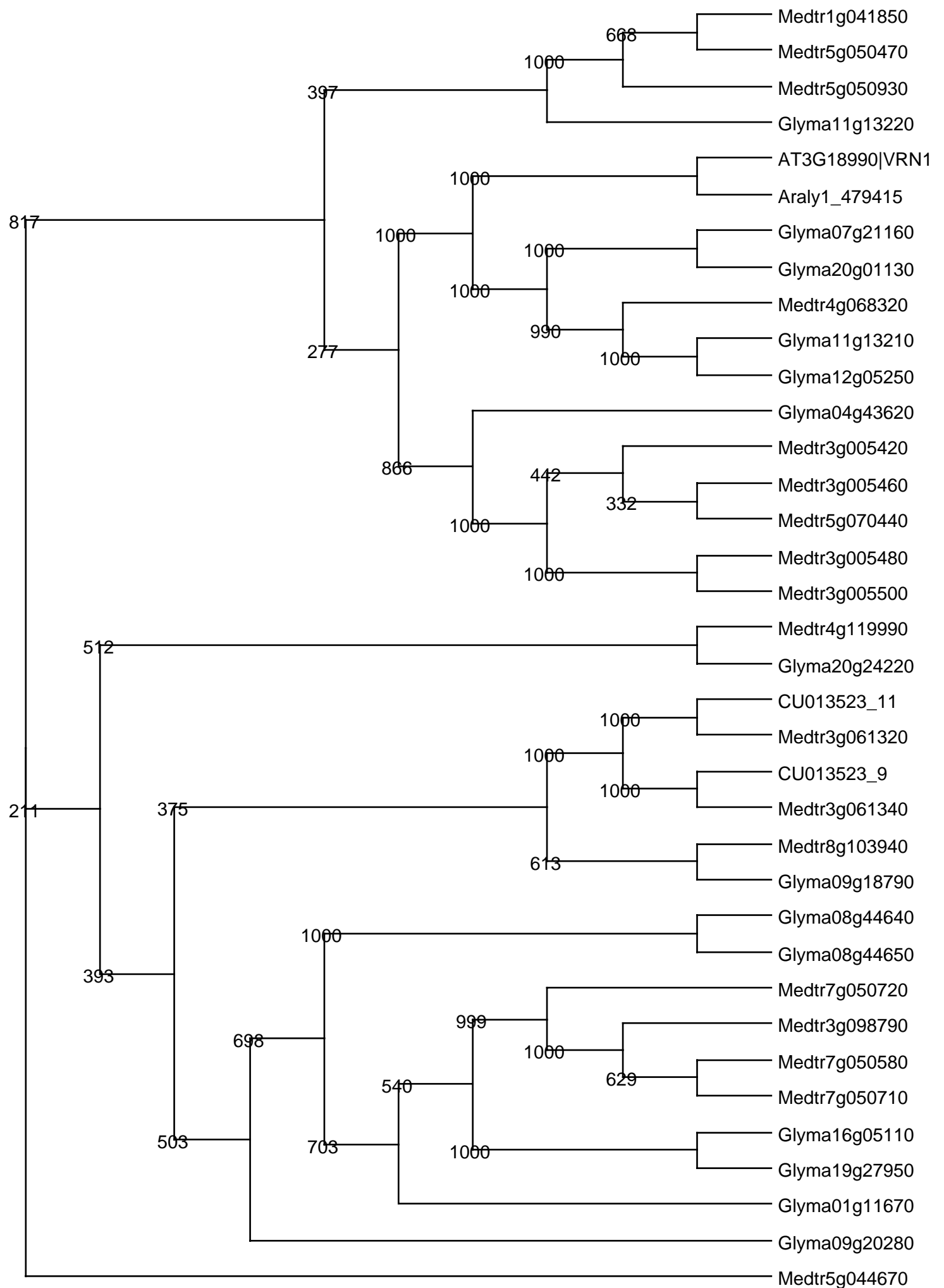

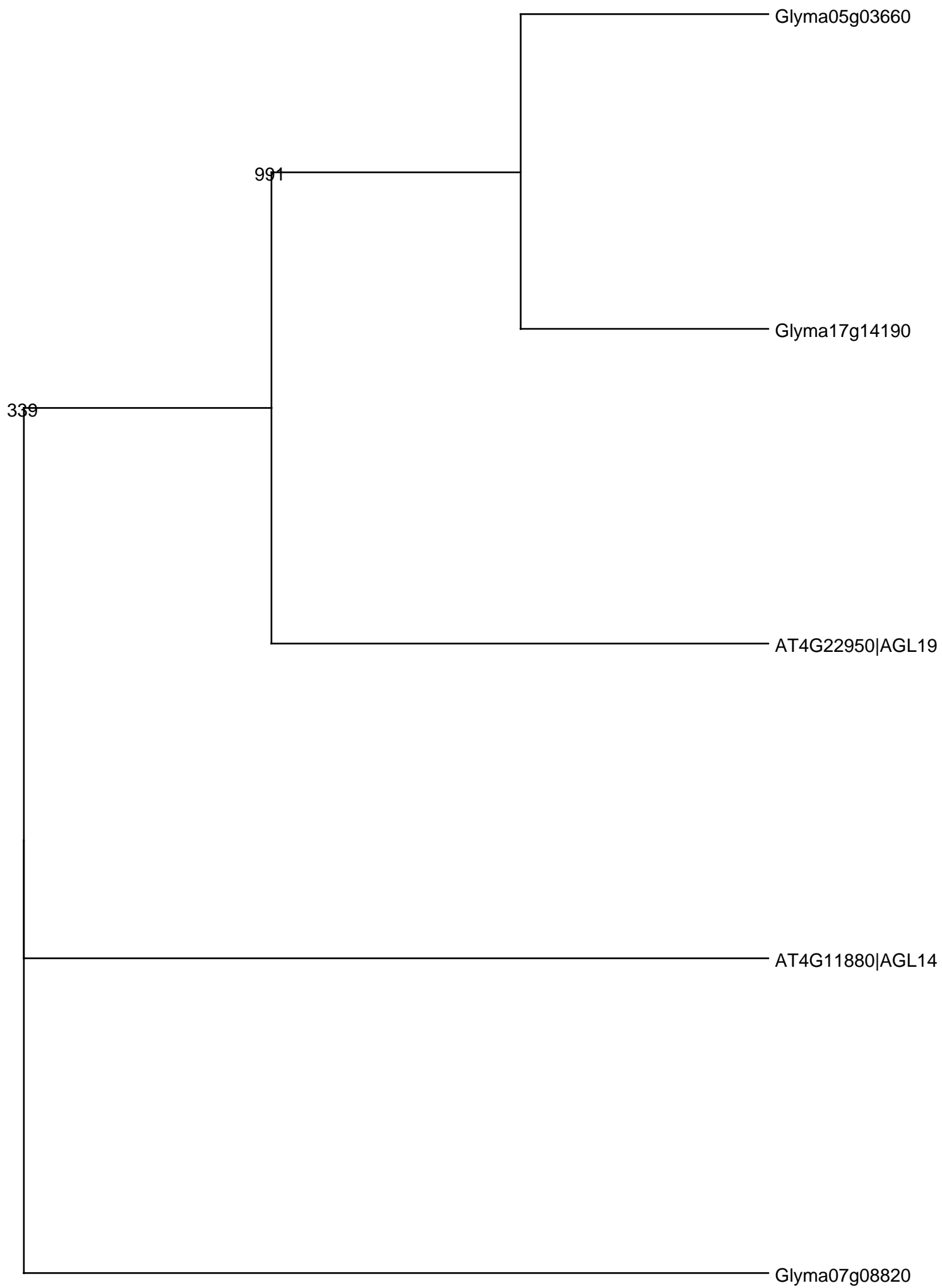

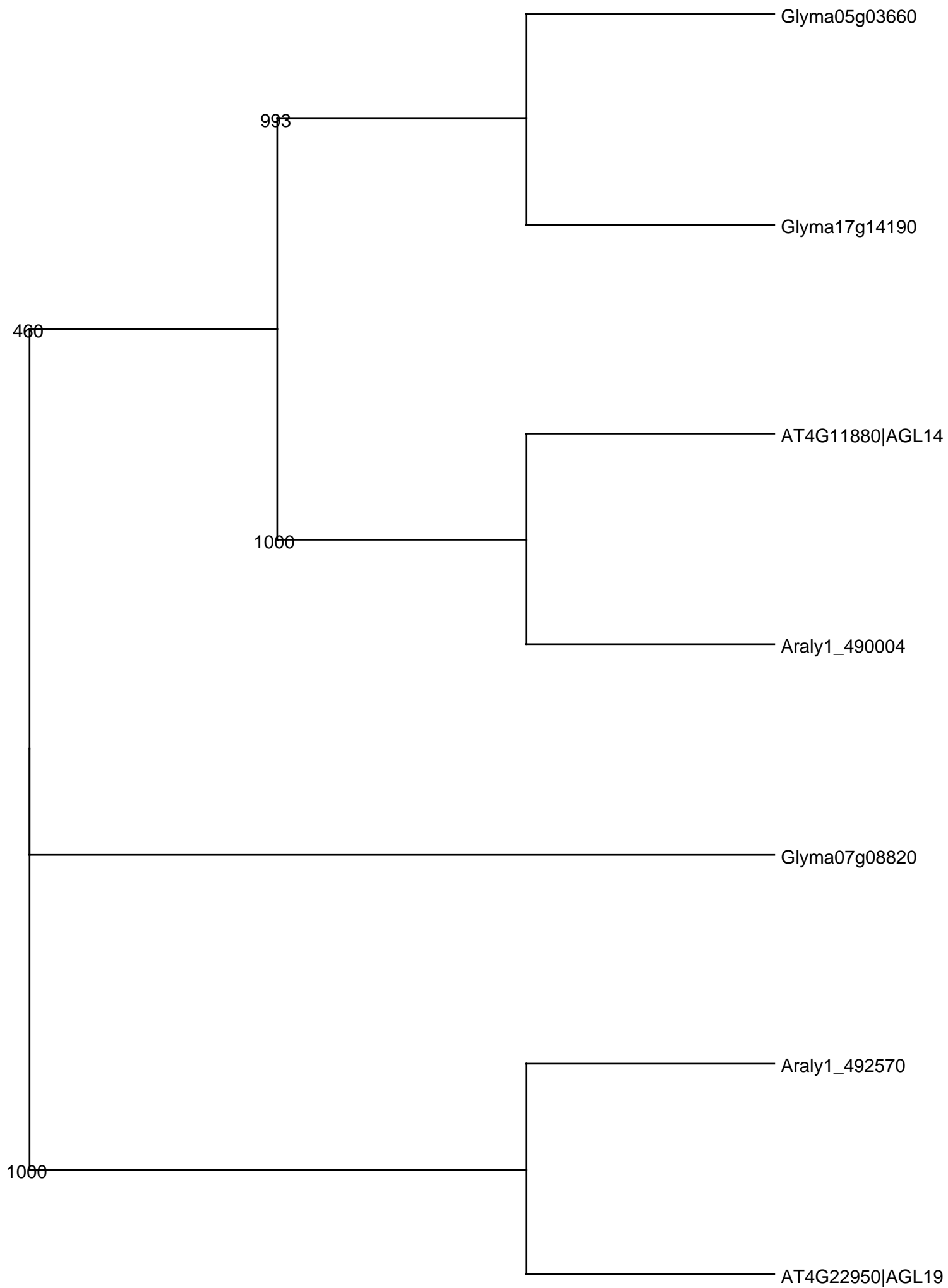

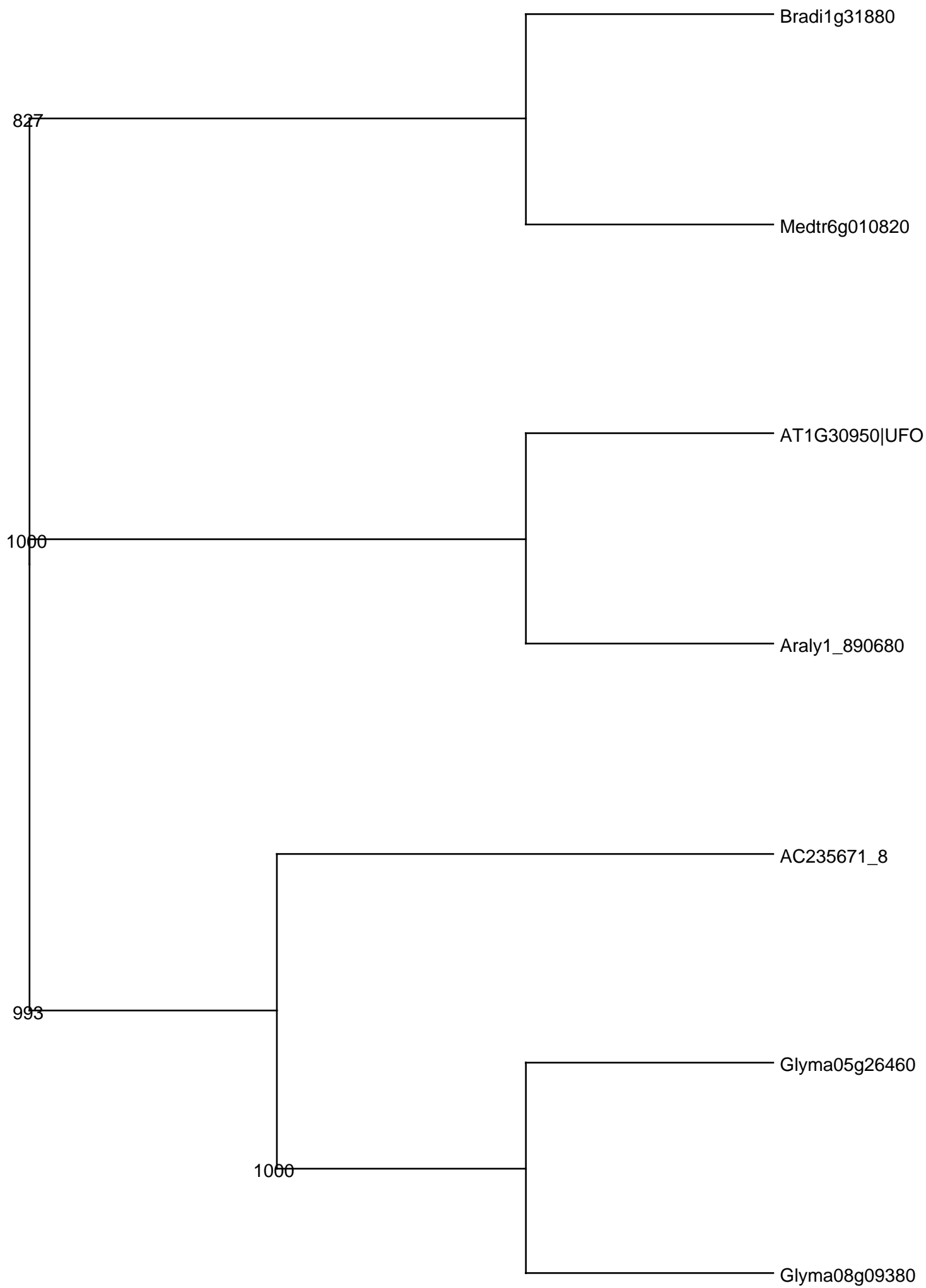

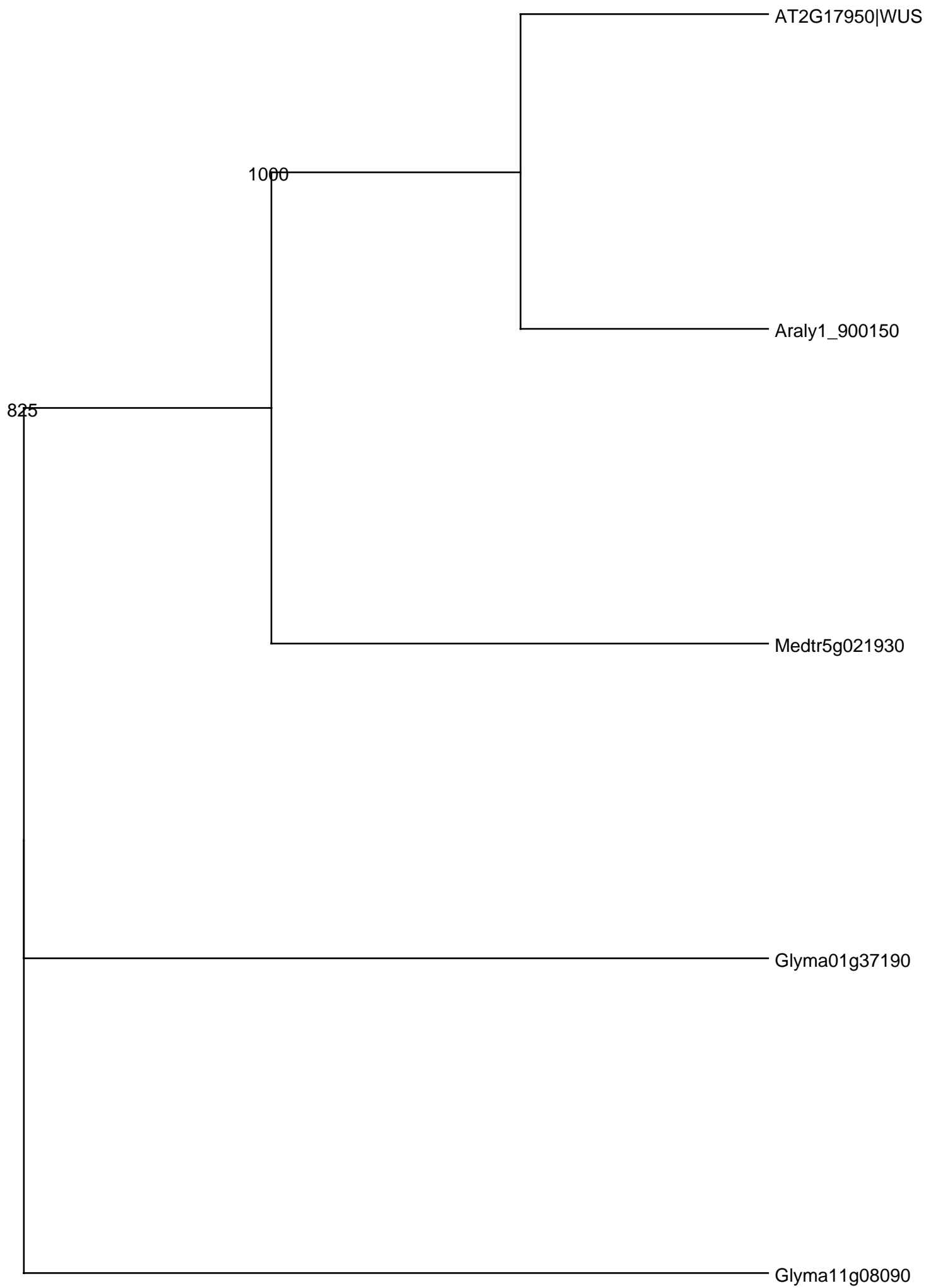

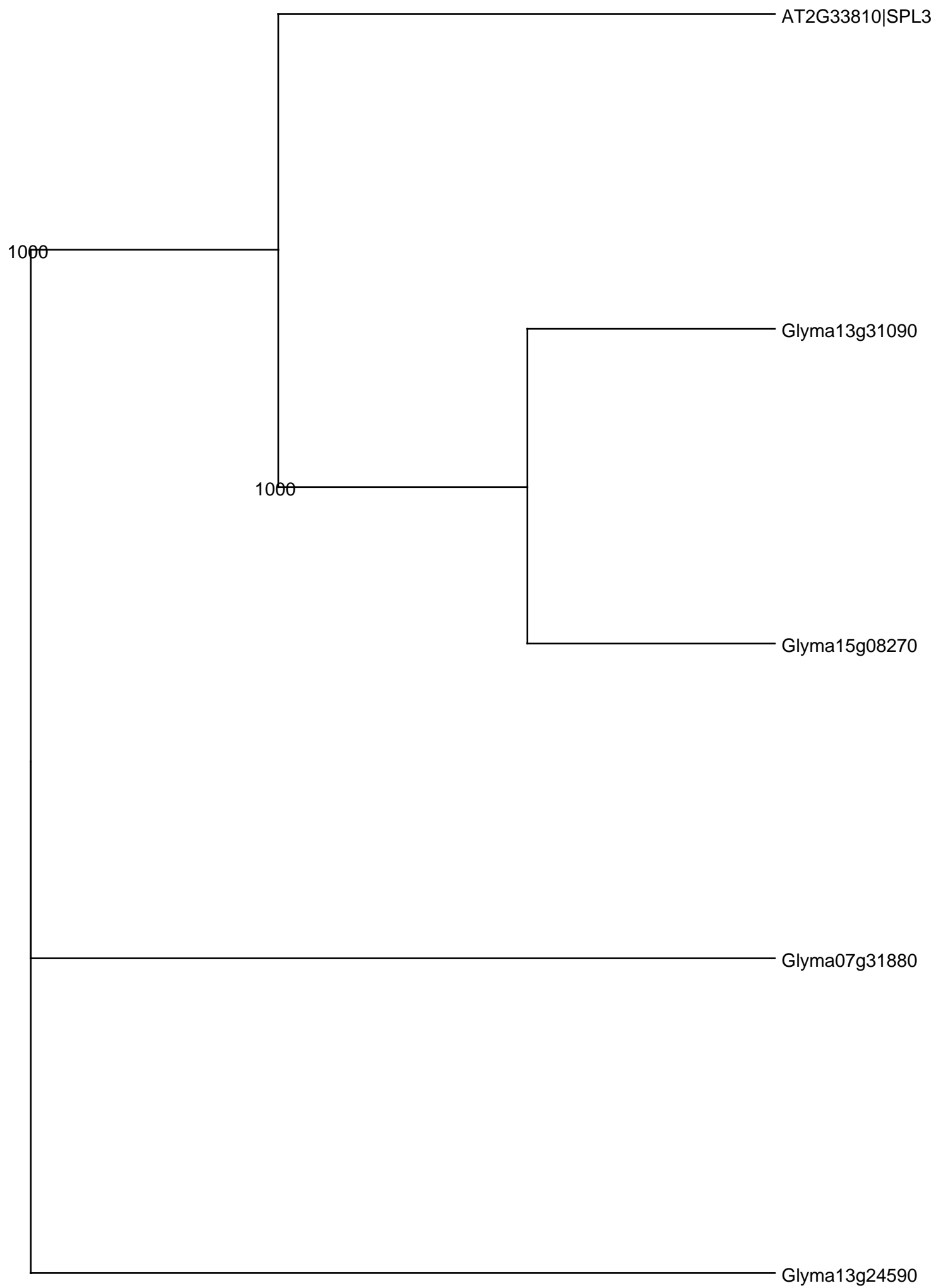

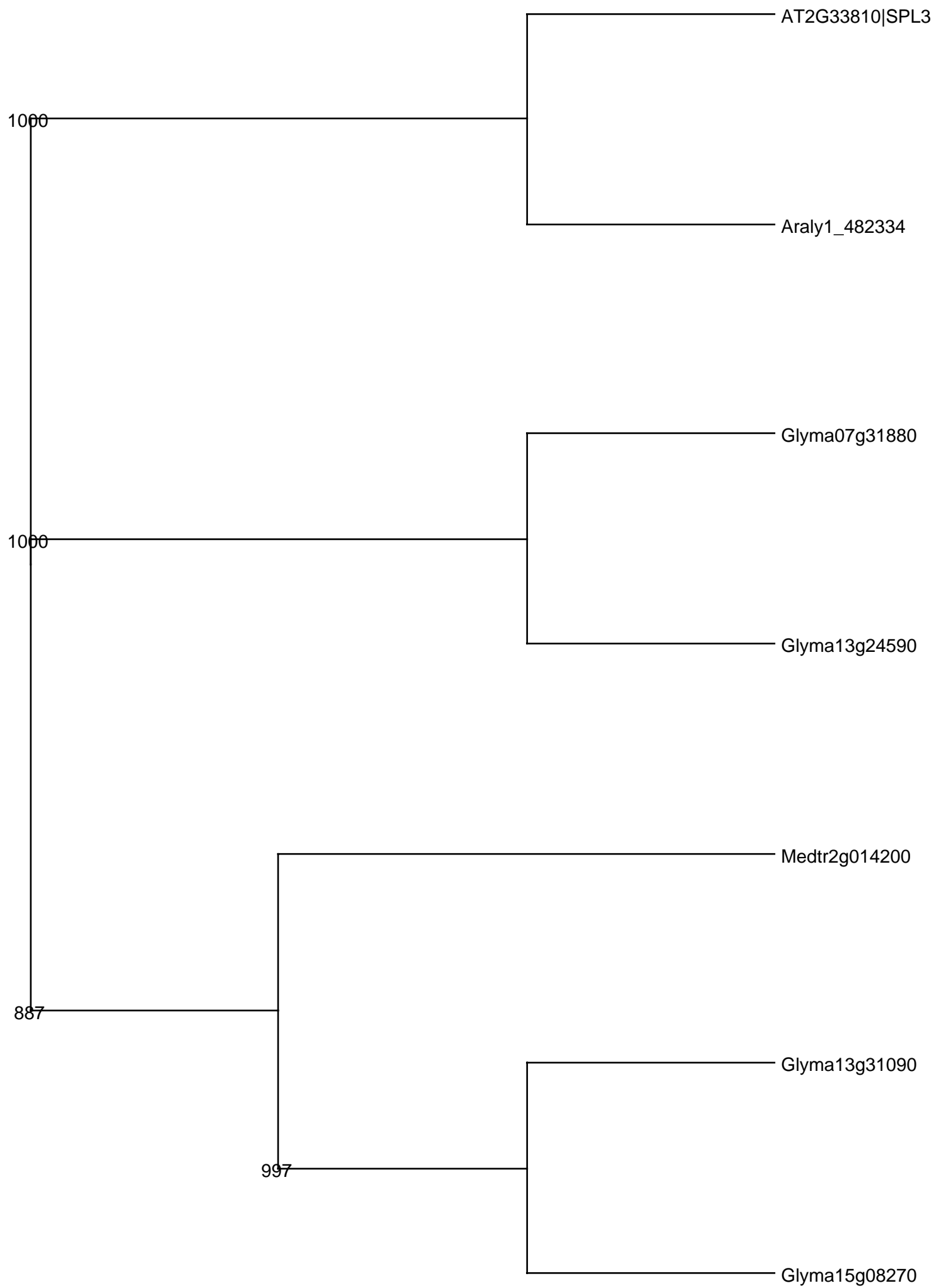

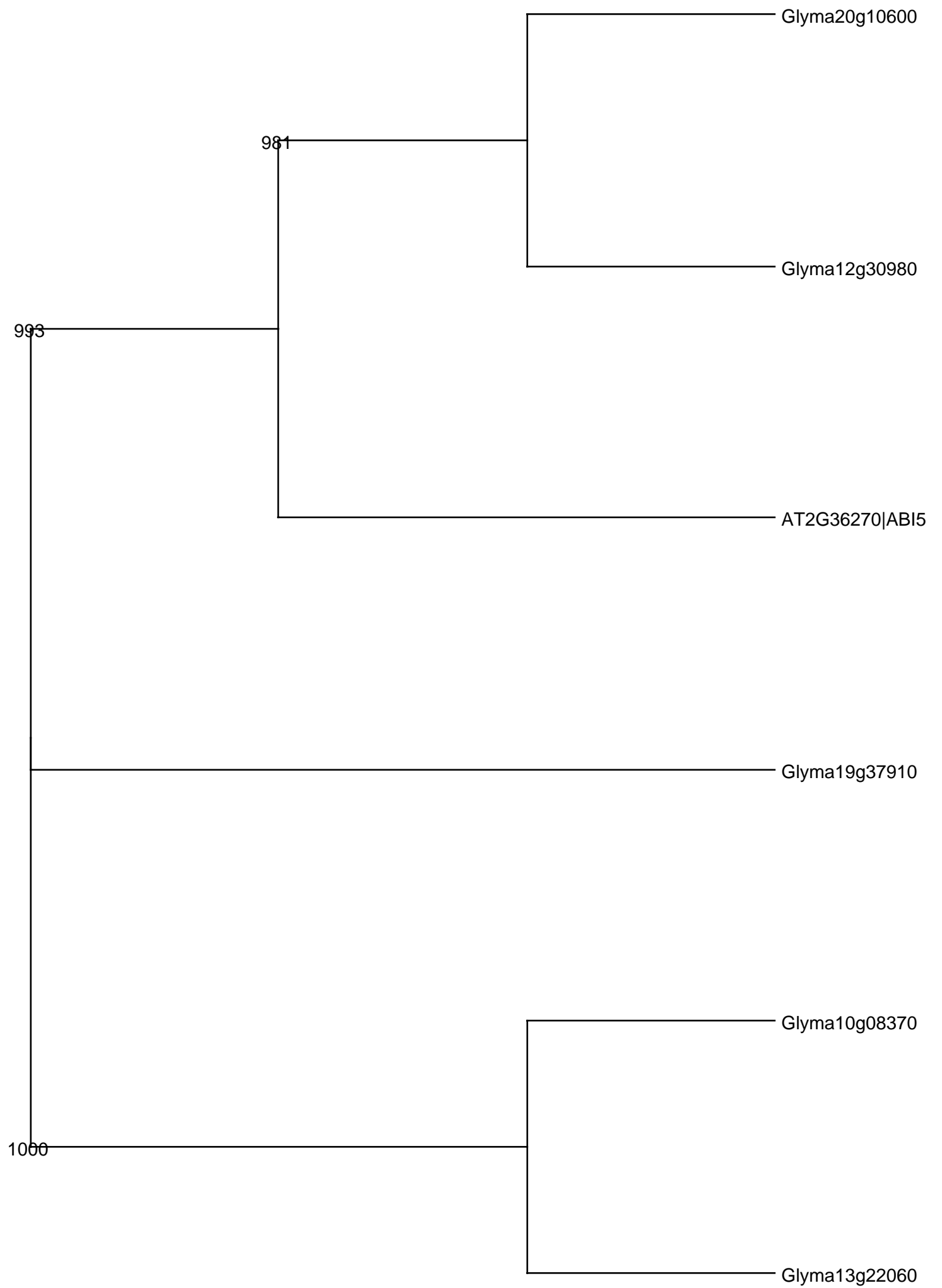

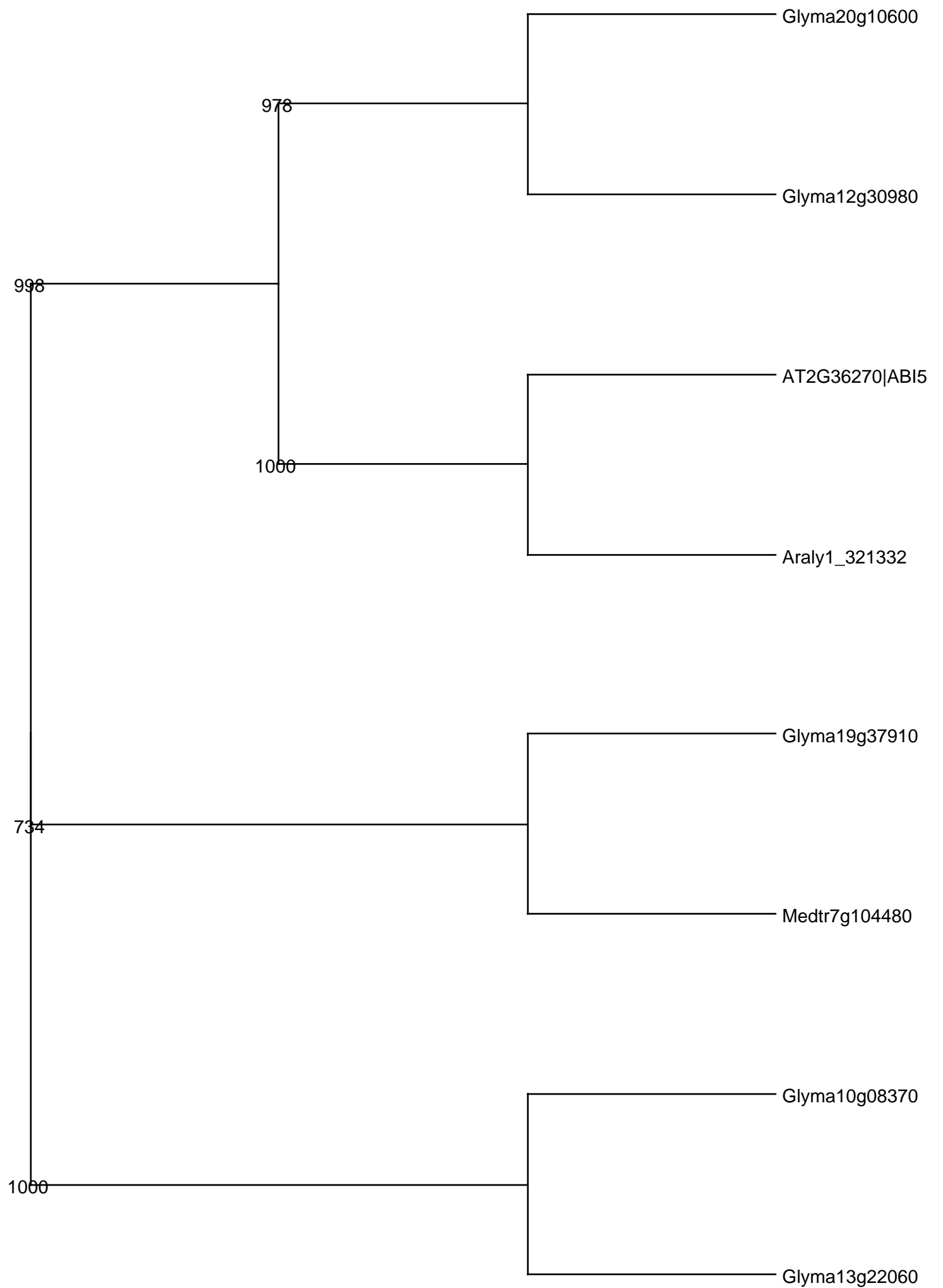

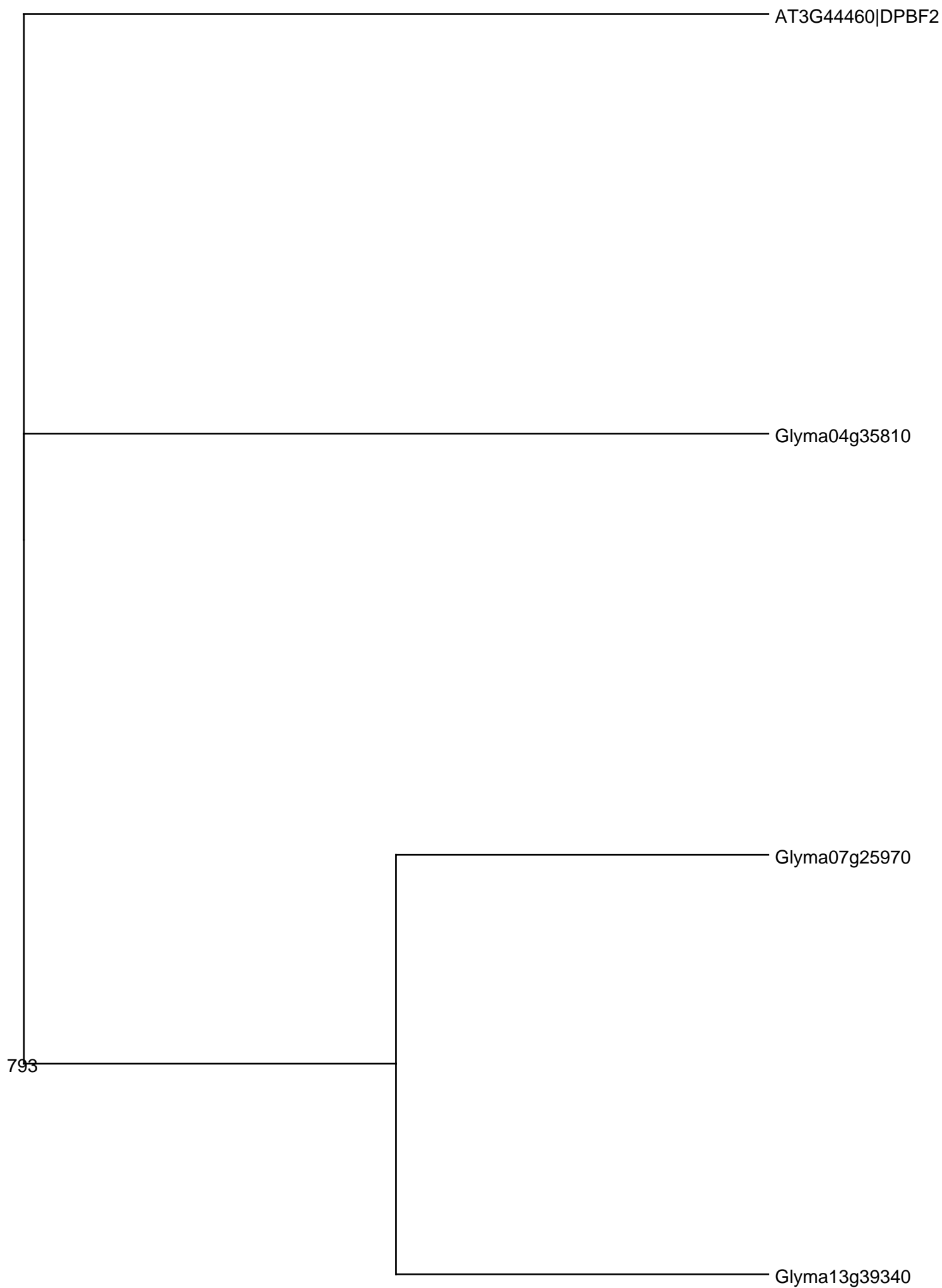

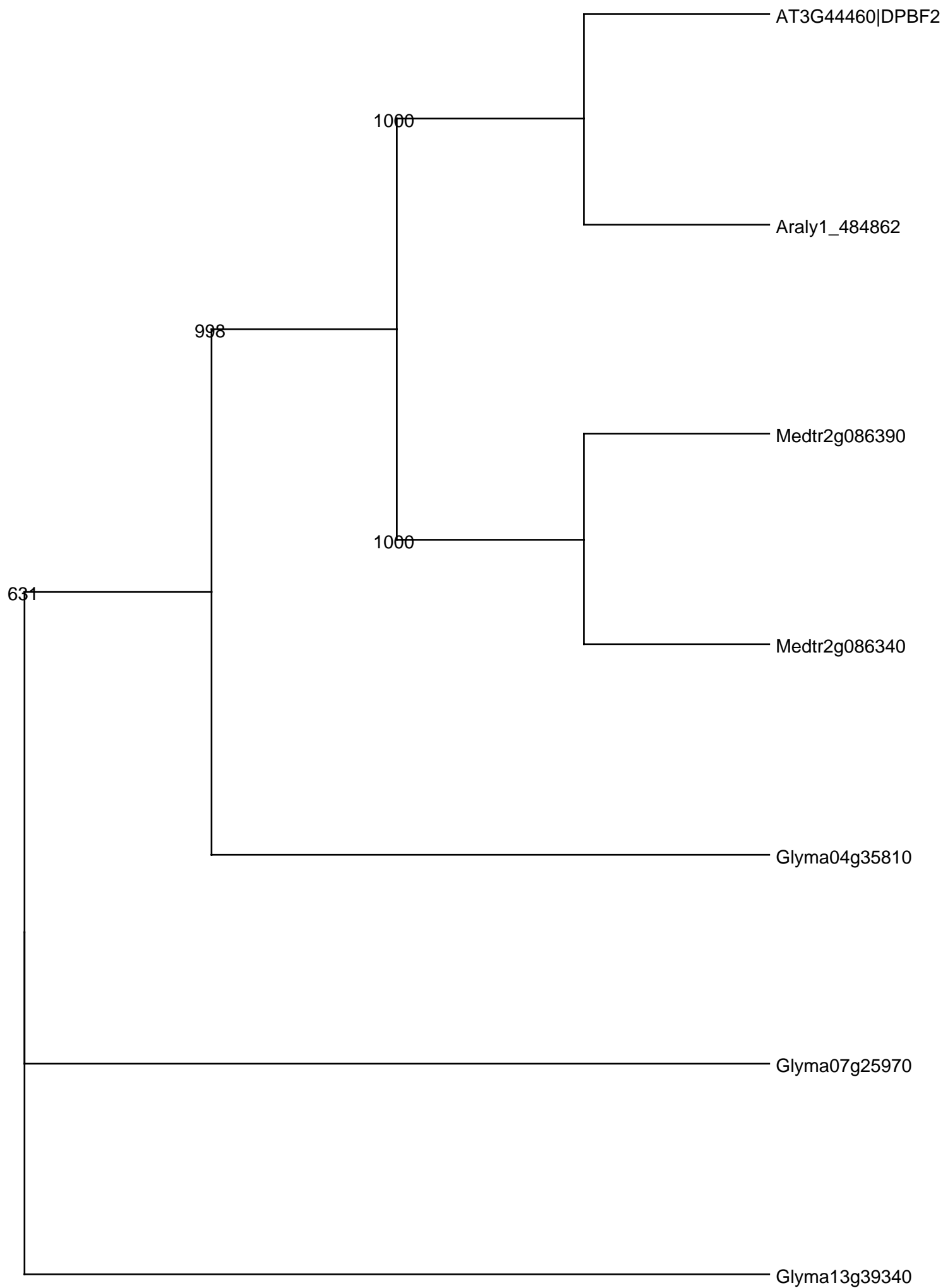

Supplement: Dataset S3 — Phylogenetic trees for OGs that contain four or more sequences including Arabidopsis flowering genes. (PDF) [file pone.0038250.s008.pdf]
