# Supplementary material for: Comparative Genomic Analysis of Soybean Flowering Genes
Source: PLoS One. 2012 Jun 5;7(6):e38250. doi: 10.1371/journal.pone.0038250 (PMC3367986; doi:10.1371/journal.pone.0038250)
Supplement: Table S1 — List of OGs containing flowering pathways genes in Arabidopsis. (PDF) [file pone.0038250.s002.pdf]

Table S1. List of OGs containing flowering pathways genes in Arabidopsis.

OG ID	Arabidopsis genes	soybean homologues	Pathway
OG5_126706	***GRF2; GRF4; GRF7; GRF1; GRF5; GRF6; GRF3; GRF8;*** *GRF10*; GRF12*; GRF11**	Glyma02g37120; Glyma04g10820; Glyma06g10650; Glyma14g35410; Glyma01g07070; Glyma02g12930; Glyma07g35240; Glyma20g03120; Glyma08g47900; Glyma18g53610; Glyma04g09820; Glyma06g09890	FPI
OG5_126901	**AT2G23070; AT2G23080;** ***CKA2; CKA1***	Glyma05g22320; Glyma17g17520; Glyma01g39950; Glyma05g22250; Glyma11g05340; Glyma17g17790	-
OG5_127148	***MSI1;*** *MSI2*; MSI3**	Glyma11g09700; Glyma12g03700; Glyma05g26150; Glyma08g09090	Au,V
OG5_127186	***CRY2; CRY1;*** *UVR3**	Glyma02g00830; Glyma10g32390; Glyma18g07770; Glyma20g35220; Glyma08g22400; Glyma04g11010; Glyma06g10830; Glyma13g01810; Glyma14g35020	L
OG5_127266	***SPY;*** *SEC**	Glyma10g31190; Glyma20g36330; Glyma02g36210; Glyma03g35610; Glyma10g08710; Glyma19g38230	-
OG5_127568	***PIE1***	Glyma02g29380; Glyma09g17220	V
OG5_127594	***NF-YB7; HAP3A; NF-YB8; NF-YB10; NF-YB3; HAP3B***	Glyma04g38860; Glyma05g32680; Glyma08g00330; Glyma10g33550; Glyma20g34050; Glyma03g33490; Glyma10g05610; Glyma11g29860; Glyma13g10690; Glyma19g36220; Glyma03g22710; Glyma05g31680; Glyma08g14930; Glyma09g01650; Glyma09g28670; Glyma11g18190; Glyma15g12570; Glyma02g46970; Glyma08g44140; Glyma18g08620	FPI
OG5_127642	***E12A11***	Glyma05g34030; Glyma08g05650	-
OG5_127884	***PAF2; PAF1***	Glyma13g24530; Glyma09g29030; Glyma14g07960; Glyma17g37050	V
OG5_127954	***ESD4;*** *ULP1A*; ULP1B**	Glyma06g37220; Glyma07g37640; Glyma09g04970; Glyma15g15890; Glyma17g03010	-
OG5_128052	***FY***	Glyma13g26820; Glyma15g37830	Au
OG5_128092	***HAP5B; HAP5A; NF-YC4***	Glyma08g17630; Glyma13g27790; Glyma02g09860; Glyma04g37290; Glyma06g17780; Glyma12g34510; Glyma13g35980	FPI
OG5_128830	***EFS***	Glyma04g42410; Glyma06g12390	V
OG5_128874	***ELF9***	Glyma03g27010; Glyma10g31450; Glyma20g36110	FPI
OG5_129164	***CLF; SWN;*** *MEA**	Glyma01g39490; Glyma03g37840; Glyma11g05760; Glyma02g01540; Glyma03g38320; Glyma10g01580; Glyma11g07150; Glyma19g40430	Au,V
OG5_129325	***SUF4***	Glyma05g34280; Glyma08g05390	V
OG5_129431	***VIP4***	Glyma04g32540; Glyma06g21900	-

OG5_129545	***LHP1***	Glyma03g23260; Glyma04g00340; Glyma06g00400; Glyma16g08860	-
OG5_129661	***ATARP6***	Glyma04g07540; Glyma06g26590	Am,V
OG5_130358	***ATSWC6***	Glyma11g29610; Glyma18g06460	V
OG5_130364	***FIE1***	Glyma02g17110; Glyma10g02690; Glyma12g34240; Glyma13g36310	Au,V
OG5_131003	***ELF5***	Glyma05g01510; Glyma17g10370	-
OG5_131018	***VIP3***	Glyma13g16700; Glyma17g05990	-
OG5_131236	***FLD;*** *LDL1*; LDL2**	Glyma07g09990; Glyma09g31770; Glyma02g18610; Glyma06g38600	Am,Au
OG5_132181	***COP1***	Glyma02g43540; Glyma14g05430	L
OG5_135817	***AGL6; AG;*** *SHP2*; SHP1*; STK**	Glyma02g45730;Glyma14g03100;Glyma03g02210;Glyma05g07350;Glyma07g08890;Glyma09g27450;Glyma16g32540;Glyma08g42300;Glyma18g12590;Glyma04g43640;Glyma06g48270;Glyma08g06980;Glyma05g29590;Glyma08g12730;Glyma13g29510;Glyma15g09500	-
OG5_136555	***PHYA; PHYB; PHYE; PHYD; PHYC***	Glyma03g38620;Glyma10g28170;Glyma19g41210;Glyma20g22160;Glyma09g03990;Glyma15g14980;Glyma09g11600;Glyma15g23400	L
OG5_138708	***LUX;*** AT5G59570*	Glyma01g36730;Glyma11g14490;Glyma12g06410	L
OG5_139246	***PRR7; APRR3***	Glyma10g05520;Glyma12g07860;Glyma13g19870;Glyma11g15560;Glyma11g15580	L
OG5_139532	***MAF1; FLC; MAF4; MAF3; MAF5; AGL31***	Glyma05g28130	Am,FPI, V
OG5_143093	***SPA4; SPA1; SPA3; SPA2***	Glyma07g06420; Glyma16g03030; Glyma06g37080; Glyma12g25240; Glyma12g35320; Glyma13g35190; Glyma01g43360; Glyma05g37070; Glyma08g02490; Glyma11g02110	L
OG5_144912	***SEP3; SEP4; SEP2; SEP1***	Glyma05g28140; Glyma08g11120; Glyma10g38580; Glyma11g36890; Glyma18g00800; Glyma20g29250; Glyma08g11110; Glyma01g08130; Glyma02g13400; Glyma08g27670; Glyma13g06730; Glyma18g50900; Glyma19g04320	-
OG5_144915	***ABF1; ABF2; ABF4***	Glyma04g04170; Glyma06g04350; Glyma02g14880; Glyma07g33600	-
OG5_144994	***ATCOL5; ATCOL4***	Glyma13g01290; Glyma17g07420; Glyma04g06240; Glyma06g06300; Glyma07g08920; Glyma08g24550; Glyma12g10320; Glyma14g21260; Glyma18g11180; Glyma18g11400	L
OG5_146511	***PIF3***	Glyma02g00980; Glyma03g38390; Glyma03g38670; Glyma10g28290; Glyma19g40980; Glyma19g41260; Glyma20g22280	-

OG5_146543	***FT; TSF; TFL1***	Glyma02g07650; Glyma08g28470; Glyma08g47810; Glyma08g47820; Glyma16g04830; Glyma16g04840; Glyma16g26660; Glyma16g26690; Glyma18g53670; Glyma19g28390; Glyma19g28400; Glyma18g53680; Glyma18g53690; Glyma03g35250; Glyma09g26550; Glyma16g32080; Glyma19g37890	Am,FPI
OG5_147254	***FVE;*** *NFC5**	Glyma09g07120; Glyma13g42660; Glyma15g02770; Glyma15g18450	Am,Au
OG5_147255	***FPF1;*** *FLP1*;* AT5G10625*	Glyma07g37770; Glyma13g41140; Glyma15g04260; Glyma17g02860; Glyma04g07900; Glyma09g05080; Glyma09g05090; Glyma14g17260; Glyma15g15730; Glyma17g29720	-
OG5_150020	***EMF2***	Glyma10g23370; Glyma10g23420; Glyma11g03950; Glyma20g16880	-
OG5_150244	***FKF1; LKP2; ZTL***	Glyma05g34530; Glyma08g05130; Glyma09g06220; Glyma13g00860; Glyma15g17480; Glyma17g06950	L
OG5_150261	**AT1G29160; AT2G34140*; CDF1***	Glyma05g29090; Glyma13g30330; Glyma15g08860; Glyma08g12230	L
OG5_150191	***COL9*; AT5G48250***	Glyma13g11590; Glyma20g07050; Glyma02g38870; Glyma14g36930	L
OG5_150317	***CDF3; CDF2***	Glyma01g05960; Glyma03g01030; Glyma04g33410; Glyma05g00970; Glyma06g20950; Glyma08g24590; Glyma15g29870; Glyma17g10920; Glyma19g29610; Glyma02g12080; Glyma07g35690; Glyma09g37170; Glyma13g05480; Glyma18g49520; Glyma19g02710; Glyma20g04600	L
OG5_152404	***HAP5C; NF-YC3***	Glyma03g39910; Glyma08g15700; Glyma10g29690; Glyma13g27780; Glyma19g42460; Glyma20g37620; Glyma13g27770	FPI
OG5_153242	***TEM2; RAV1; TEM1;* AT3G25730**	Glyma01g22260; Glyma02g11060; Glyma10g34760; Glyma20g32730	L
OG5_153294	***ELF3;* AT3G21320**	Glyma04g05280; Glyma07g01600; Glyma08g21110; Glyma14g10530; Glyma17g34980	L
OG5_153332	***EEL; AREB3***	Glyma05g25200; Glyma13g02310; Glyma13g03880; Glyma14g33810; Glyma02g05100; Glyma03g00580; Glyma04g02420; Glyma04g14840; Glyma05g13890; Glyma06g02470; Glyma06g47220; Glyma08g24340; Glyma15g35080; Glyma19g20090; Glyma19g30230	-
OG5_153389	***TOE1***	Glyma02g09600; Glyma11g15650; Glyma12g07800; Glyma13g40470; Glyma15g04930	-
OG5_153420	***CCA1***	Glyma07g05410	L
OG5_155119	***FCA***	Glyma17g03960	Am,Au
OG5_156252	***HUA2;*** AT5G08230*	Glyma11g10670; Glyma12g02980	V
OG5_156319	***COL2; CO; COL1***	Glyma08g28370; Glyma13g07030; Glyma18g51320; Glyma19g05170	FPI,L
OG5_156377	***ELF4-L4; ELF4-L2***	Glyma07g04180; Glyma13g16850; Glyma16g00890; Glyma17g05860	-

OG5_156386	***GBF4;* AT5G44080**	Glyma08g08220; Glyma08g19590; Glyma10g36820; Glyma15g05440	-
OG5_158796	***ATC***	Glyma10g08340; Glyma12g30940; Glyma13g22030; Glyma13g39360	-
OG5_160037	***FPA***	Glyma11g13490; Glyma12g05490; Glyma13g42060; Glyma15g03330	Au
OG5_163423	***FLK***	Glyma02g15850; Glyma03g31670; Glyma03g40840; Glyma10g03910; Glyma19g34470; Glyma19g43540	Au
OG5_164233	***LHY***	Glyma03g42260; Glyma16g01980; Glyma19g45030	L
OG5_164556	***AGL18; AGL15***	Glyma02g33040; Glyma14g36220; Glyma15g06470; Glyma11g16110; Glyma12g17720	-
OG5_169532	***PI***	Glyma04g42420; Glyma06g12380; Glyma13g09660; Glyma14g24590	-
OG5_169591	***VRN2***	Glyma01g41460; Glyma11g03960	Au,V
OG5_170135	***PFT1***	Glyma01g21710; Glyma02g10880	-
OG5_170227	***TT16***	Glyma09g42060; Glyma20g00400	-
OG5_170388	***AP1; CAL***	Glyma01g08150; Glyma02g13420; Glyma08g36380; Glyma16g13070	FPI,MI
OG5_170601	***LD***	Glyma03g36970; Glyma19g39620	Au
OG5_170648	***VEL1***	Glyma02g37880; Glyma13g00920; Glyma14g36100; Glyma17g07000	Au,V
OG5_170666	***AP2***	Glyma01g39520; Glyma03g33470; Glyma05g18170; Glyma10g22390; Glyma11g05720; Glyma17g18640; Glyma19g36200	-
OG5_170678	***ELF6***	Glyma10g35350; Glyma20g32160	-
OG5_170687	***CHE;*** AT5G23280*	Glyma11g20200; Glyma09g42120; Glyma09g42140; Glyma10g43190; Glyma20g00350; Glyma20g23700	L
OG5_170758	***TOC1***	Glyma04g33110; Glyma05g00880; Glyma06g21120; Glyma17g11040	L
OG5_170760	***LFY***	Glyma04g37900; Glyma06g17170; Glyma20g19600	FPI,MI
OG5_170827	***ELF4; ELF4-L3***	Glyma11g35270; Glyma14g06480; Glyma18g03130; Glyma09g07770; Glyma15g19390	L
OG5_170837	**AT2G25920**	Glyma02g01760; Glyma10g01830	-
OG5_177438	***SVP***	Glyma01g02880; Glyma02g04710; Glyma06g10020; Glyma07g30040; Glyma08g07260; Glyma13g33040; Glyma15g06300; Glyma15g06310	Am,V
OG5_178368	***APRR5***	Glyma03g42220; Glyma04g40640; Glyma06g14150; Glyma07g05530; Glyma16g02050; Glyma19g44970	L
OG5_189809	***FRL2; FRL1***	Glyma02g46680; Glyma08g43760; Glyma18g09060	V
OG5_189849	**AT1G51120; AT1G50680**	Glyma20g39140	-
OG5_190004	***ATBZIP27; FD***	Glyma01g36810	Am,MI

OG5_190130	***SOC1***	Glyma03g02200; Glyma07g08830; Glyma09g40230; Glyma18g45780	FPI
OG5_190237	***VRN5***	Glyma05g35280; Glyma07g09800; Glyma08g04440; Glyma09g32010	Au,V
OG5_190319	***AP3***	Glyma01g37470; Glyma04g02980; Glyma06g02990; Glyma11g07820; Glyma12g13560; Glyma15g23610; Glyma16g17450; Glyma18g33910	-
OG5_190441	***PEP***	Glyma09g06750; Glyma09g38290; Glyma13g00510; Glyma15g18010; Glyma15g18050; Glyma17g06640	V
OG5_190478	***CIB1***	Glyma04g01400; Glyma06g01430; Glyma12g04670	L
OG5_190563	***EMF1***	Glyma04g08680; Glyma06g08790	-
OG5_190821	***GI***	Glyma09g07240; Glyma10g36600; Glyma20g30980	L
OG5_190987	***FRI***	Glyma04g38060; Glyma06g17010	V
OG5_191085	***REF6***	Glyma04g36620; Glyma04g36630; Glyma06g18290; Glyma06g18300	-
OG5_211687	***FUL***	Glyma04g31800; Glyma04g31810; Glyma05g07380; Glyma06g22650; Glyma08g27680; Glyma17g08890; Glyma18g50910	MI
OG5_212080	***STM***	Glyma02g43760; Glyma07g39350; Glyma09g01000; Glyma14g05150; Glyma15g11850; Glyma17g01370	-
OG5_212214	***AGL17;*** *AGL21**	Glyma01g02520; Glyma15g06490; Glyma01g02530; Glyma02g38090	-
OG5_212244	***ELF4-L1***	Glyma14g37280	-
OG5_212406	***VRN1***	Glyma01g11670; Glyma04g43620; Glyma07g21160; Glyma08g44640; Glyma08g44650; Glyma09g18790; Glyma09g20280; Glyma11g13210; Glyma11g13220; Glyma12g05250; Glyma16g05110; Glyma19g27950; Glyma20g01130; Glyma20g24220	V
OG5_212591	***AGL19; AGL14***	Glyma05g03660; Glyma07g08820; Glyma17g14190	V
OG5_213031	***UFO***	Glyma05g26460; Glyma08g09380	-
OG5_213092	***WUS***	Glyma01g37190; Glyma11g08090	-
OG5_213105	***SPL3***	Glyma07g31880; Glyma13g24590; Glyma13g31090; Glyma15g08270	MI
OG5_213108	***ABI5***	Glyma10g08370; Glyma12g30980; Glyma13g22060; Glyma19g37910; Glyma20g10600	-
OG5_213158	***DPBF2***	Glyma04g35810; Glyma07g25970; Glyma13g39340	-
OG5_AT2G33835	***FES1***	Glyma13g31050; Glyma15g08320	V
OG5_143135	***TOE3***	-	-
OG5_149770	***FIS2***	-	-
OG5_158920	**AT5G27220**	-	-

OG5_163093	**AT5G62040**	-	-
OG5_170476	***APRR9;*** AT2G46670*	-	L
OG5_189502	***ABF3***	-	-
OG5_189564	***SNZ; SMZ***	-	-
OG5_211968	**AT1G26790*; CDF5***	-	L
OG5_212223	***COL3***	-	-
OG5_213314	***RFI2***	-	L
OG5_AT2G18870	***VEL3***	-	Au
OG5_AT2G18880	***VEL2***	-	Au
OG5_AT3G30260	***AGL79***	-	-
OG5_AT4G16810	**AT4G16810**	-	-
OG5_AT4G24540	***AGL24***	-	V
OG5_AT5G42910	**AT5G42910**	-	-
OG5_AT5G57380	***VIN3***	-	Au,V
OG5_AT5G60120	***TOE2***	-	-

* For Arabidopsis genes, those that are known as flowering genes are indicated with bold face.

** Am: Ambient temperature pathway, Au: Autonomous pathway, FPI: Flowering Pathway Intergrators, L: Light signaling pathway, MI: Meristem Identity, V: Vernalization pathway
