# Supplementary material for: Comparative Genomic Analysis of Soybean Flowering Genes
Source: PLoS One. 2012 Jun 5;7(6):e38250. doi: 10.1371/journal.pone.0038250 (PMC3367986; doi:10.1371/journal.pone.0038250)
Supplement: Table S2 — Soybean flowering genes preferentially or specifically expressed in flower. (PDF) [file pone.0038250.s003.pdf]

**Table S2. Soybean flowering genes preferentially or specifically expressed in flower**

| soybean | description | OG_ID | Arabidopsis genes |
|---|---|---|---|
| Glyma05g32680 | CCAAT-binding factor, subunit A (HAP3) | OG5_127594 | *NF-YB7;HAP3A;NF-YB8;NF-YB10;NF-YB3;HAP3B* |
| Glyma03g02210 | MADS box transcription factor | OG5_135817 | *SHP2;AGL6;SHP1;STK;AG* |
| Glyma07g08890 | MADS box transcription factor | | |
| Glyma05g28140 | - | OG5_144912 | *SEP3;SEP4;SEP2;SEP1* |
| Glyma08g11120 | MADS box transcription factor | | |
| Glyma18g00800 | - | | |
| Glyma16g32080 | - | OG5_146543 | *FT;TSF;TFL1* |
| Glyma20g07050 | - | OG5_150191 | *COL9;AT5G48250* |
| Glyma02g33040 | MADS box transcription factor | OG5_164556 | *AGL18;AGL15* |
| Glyma06g12380 | MADS box transcription factor | OG5_169532 | *PI* |
| Glyma13g09660 | MADS box transcription factor | | |
| Glyma14g24590 | MADS box transcription factor | | |
| Glyma01g08150 | MADS box transcription factor | OG5_170388 | *AP1;CAL* |
| Glyma02g13420 | MADS box transcription factor | | |
| Glyma16g13070 | MADS box transcription factor | | |
| Glyma11g35270 | - | OG5_170827 | *ELF4;ELF4-L3* |
| Glyma04g02980 | MADS box transcription factor | OG5_190319 | *AP3* |
| Glyma06g02990 | MADS box transcription factor | | |
| Glyma11g07820 | - | | |
