# Supplementary material for: Comparative Genomic Analysis of Soybean Flowering Genes
Source: PLoS One. 2012 Jun 5;7(6):e38250. doi: 10.1371/journal.pone.0038250 (PMC3367986; doi:10.1371/journal.pone.0038250)
Supplement: Table S3 — G. max genes containing large structural variation in comparison with G. soja . (PDF) [file pone.0038250.s004.pdf]

Table S3. ***G. max*** **genes containing large structural variation in comparison with** ***G. soja***

**Large deletion sites**		
G. max genes	OrthoMCL_ID	key flowering pathways involved
Glyma10g32390<br>Glyma18g07770	OG5_127186	light signaling
Glyma03g35610<br>Glyma10g08710	OG5_127266	-
Glyma14g05430	OG5_132181	light signaling
Glyma19g41210<br>Glyma15g23400	OG5_136555	light signaling
Glyma05g28130	OG5_139532	flowering pathway integrator, vernalization, ambient temperature
Glyma08g11110	OG5_144912	-
Glyma08g44650	OG5_212406	vernalization, meristem identity

**Large insertion sites**		
G. max genes	OrthoMCL_ID	key flowering pathways involved
Glyma01g39950	OG5_126901	-
Glyma10g02690	OG5_130364	autonomous, vernalization
Glyma05g01510	OG5_131003	-
Glyma03g02210<br>Glyma15g09500	OG5_135817	-
Glyma15g23400	OG5_136555	light signaling pathways
Glyma15g04260	OG5_147255	-
Glyma15g35080	OG5_153332	-
Glyma06g17010	OG5_190987	Vernalization
Glyma08g44650<br>Glyma16g05110	OG5_212406	vernalization, meristem identity
