# Supplementary material for: Comparative Genomic Analysis of Soybean Flowering Genes
Source: PLoS One. 2012 Jun 5;7(6):e38250. doi: 10.1371/journal.pone.0038250 (PMC3367986; doi:10.1371/journal.pone.0038250)
Supplement: Table S4 — Gene Ontology analysis on soybean genes of different categories. (PDF) [file pone.0038250.s005.pdf]

Table S4. Gene Ontology analysis on soybean genes of different categories

paralogue-rich		
GO Term	gene count*	p-value†
GO:0009733; response to auxin stimulus	23	4.80E-19
GO:0006952; defense response	23	3.66E-09
GO:0009611; response to wounding	6	0.000388
GO:0006869; lipid transport	8	0.002977

paralogue-less		
GO Term	gene count*	p-value†
GO:0008150; biological_process	880	1.77E-24
GO:0006952; defense response	69	2.61E-17
GO:0007242; intracellular signaling cascade	28	4.80E-15
GO:0009664; cell wall organization and biogenesis (sensu Magnoliophyta)	12	7.68E-07
GO:0006355; regulation of transcription, DNA-dependent	91	3.09E-06
GO:0006629; lipid metabolic process	29	6.90E-05
GO:0006468; protein amino acid phosphorylation	92	0.000907
GO:0008152; metabolic process	58	0.001331
GO:0006633; fatty acid biosynthetic process	10	0.017789

Gene Ontology Term enrichment analysis was carried out by GeneMerge (Castillo-Davis and Hartl, 2003, *Bioinformatics*, 19:891).

*: Arabidopsis gene counts

†: Adjusted by Bonferroni method
